# Deciphering the working mechanism of aggregation-induced emission of tetraphenylethylene derivatives by ultrafast spectroscopy†
†Electronic supplementary information (ESI) available: Synthetic procedures and characterization data (NMR and X-ray) of all newly synthesized compounds, details of the photophysical properties, the ultrafast spectroscopy studies, and the DFT calculations. CCDC 1579022 (**2**), 1586452 (**3**), 1579026 (**4**), 1579025 (**6**), 1579024 (**2-PC**), 1579027 (**3-PC**), 1579021 (**4-PC**) and 1579023 (**6-PC**). For ESI and crystallographic data in CIF or other electronic format see DOI: 10.1039/c8sc01170b


**DOI:** 10.1039/c8sc01170b

**Published:** 2018-04-24

**Authors:** Yuanjing Cai, Lili Du, Kerim Samedov, Xinggui Gu, Fei Qi, Herman H. Y. Sung, Brian O. Patrick, Zhiping Yan, Xiaofang Jiang, Haoke Zhang, Jacky W. Y. Lam, Ian D. Williams, David Lee Phillips, Anjun Qin, Ben Zhong Tang

**Affiliations:** a Department of Chemistry , Hong Kong Branch of Chinese National Engineering Research Center for Tissue Restoration and Reconstruction , The Hong Kong University of Science & Technology , Clear Water Bay , Kowloon , Hong Kong SAR , China . Email: tangbenz@ust.hk; b Center for Aggregation-Induced Emission , NSFC Center for Luminescence from Molecular Aggregates , SCUT-HKUST Joint Research Institute , State Key Laboratory of Luminescent Materials and Devices , South China University of Technology , Guangzhou 510640 , China . Email: msqinaj@scut.edu.cn; c HKUST Shenzhen Research Institute , No. 9 Yuexing 1st RD, South Area, Hitech Park Nanshan , Shenzhen 518057 , China; d Department of Chemistry , The University of Hong Kong , Pokfulam Road , Hong Kong SAR , China . Email: phillips@hku.hk; e Department of Chemistry , University of British Columbia , 2036 Main Mall , Vancouver , British Columbia , Canada V6T 1Z1; f Institute of Computational and Theoretical Studies & Department of Physics , Hong Kong Baptist University , Hong Kong SAR , China; g Institute of Life Sciences , Jiangsu University , Zhenjiang 212013 , China

## Abstract

Aggregation-induced emission (AIE) is the long-sought solution to the problem of aggregation-caused quenching that has hampered efficient application of fluorescent organic materials. An important goal on the way to fully understand the working mechanism of the AIE process was, for more than a decade, and still remains obtaining more comprehensive insights into the correlation between the ultrafast excited-state dynamics in tetraphenylethylene (TPE)-based molecules and the AIE effect in them. Here we report a number of TPE-based derivatives with varying structural rigidities and AIE properties. Using a combination of ultrafast time-resolved spectroscopy and computational studies, we observe a direct correlation between the state-dependent coupling motions and inhibited fluorescence, and prove the existence of photocyclized intermediates in them. We demonstrate that the dominant non-radiative relaxation dynamics, *i.e.* formation of intermediate or rotation around the elongated C=C bond, is responsible for the AIE effect, which is strongly structure-dependent but not related to structural rigidity.

## Abstract 2

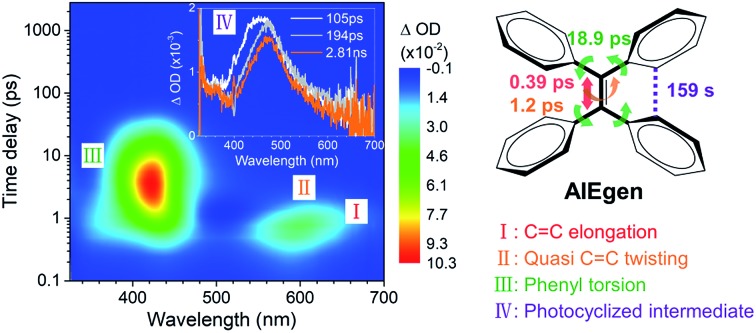
Photocyclized intermediate formation and quasi C=C twisting are the dominant processes behind the AIE.

## Introduction

In less than two decades, the seminal discovery of aggregation-induced emission (AIE)^1^,^2^ has revolutionized the research fields of bioimaging,^3^–^5^ chemo/biosensing,^6^–^8^ and optoelectronic materials sciences.^9^,^10^ AIE describes the phenomenon of molecules with low fluorescence quantum yields in solution “lighting up” with enhanced fluorescence emission upon aggregation. The AIE effect has proven to be an effective molecular-level remedy for the aggregation-caused quenching effect,^11^,^12^ which has been a major obstacle in the way of many practical applications of most fluorescent materials for decades. However, the working mechanism behind the AIE phenomenon remains a subject of much debate and intense elucidation efforts.^13^–^27^ Among several hypotheses concerning the mechanism of the AIE effect, the restriction of intramolecular rotation (RIR)^15^–^19^ and restriction of intramolecular vibration (RIV)^20^–^22^ subsumed under the overarching concept of restriction of intramolecular motion (RIM)^2^,^21^ are the most frequently cited ones, with photocyclization^23^–^25^ as well as *E*–*Z* isomerization^18^,^26^,^27^ being invoked in a number of cases. Although the discourse nowadays appears to lend much support to the general mechanism of RIM due to an extensive body of experimental evidence substantiated in part by computational studies, a clear, consistent, and deep fundamental understanding of different elemental relaxation processes associated with the basic molecular motions in non-radiative excited-state dynamics even in the simplest archetypal AIE-active compounds such as tetraphenylethylene (TPE) is yet to emerge.

The excited-state dynamics in TPE and TPE derivatives, including the role of phenyl rotation,^15^,^28^,^29^ C=C twisting^30^–^33^ and photocyclization,^24^,^25^,^34^ have been under debate and investigation for almost four decades. Certain aspects of it, such as how the non-radiative relaxation takes place, what photophysical processes are involved, what the relationships between the intramolecular motions and their respective relative contributions to the AIE mechanism are, and whether they involve photochemical reactions, have not been sufficiently, if at all, addressed neither in previous nor in contemporary subject-related reports. It is thus of fundamental importance to develop a detailed understanding of the ultrafast processes that dominate the radiative and non-radiative excited-state dynamics in TPE derivatives. It would furthermore be a significant step towards perfecting the rational molecular design of highly efficient AIE luminogens (AIEgens).^35^

**Scheme 1:**
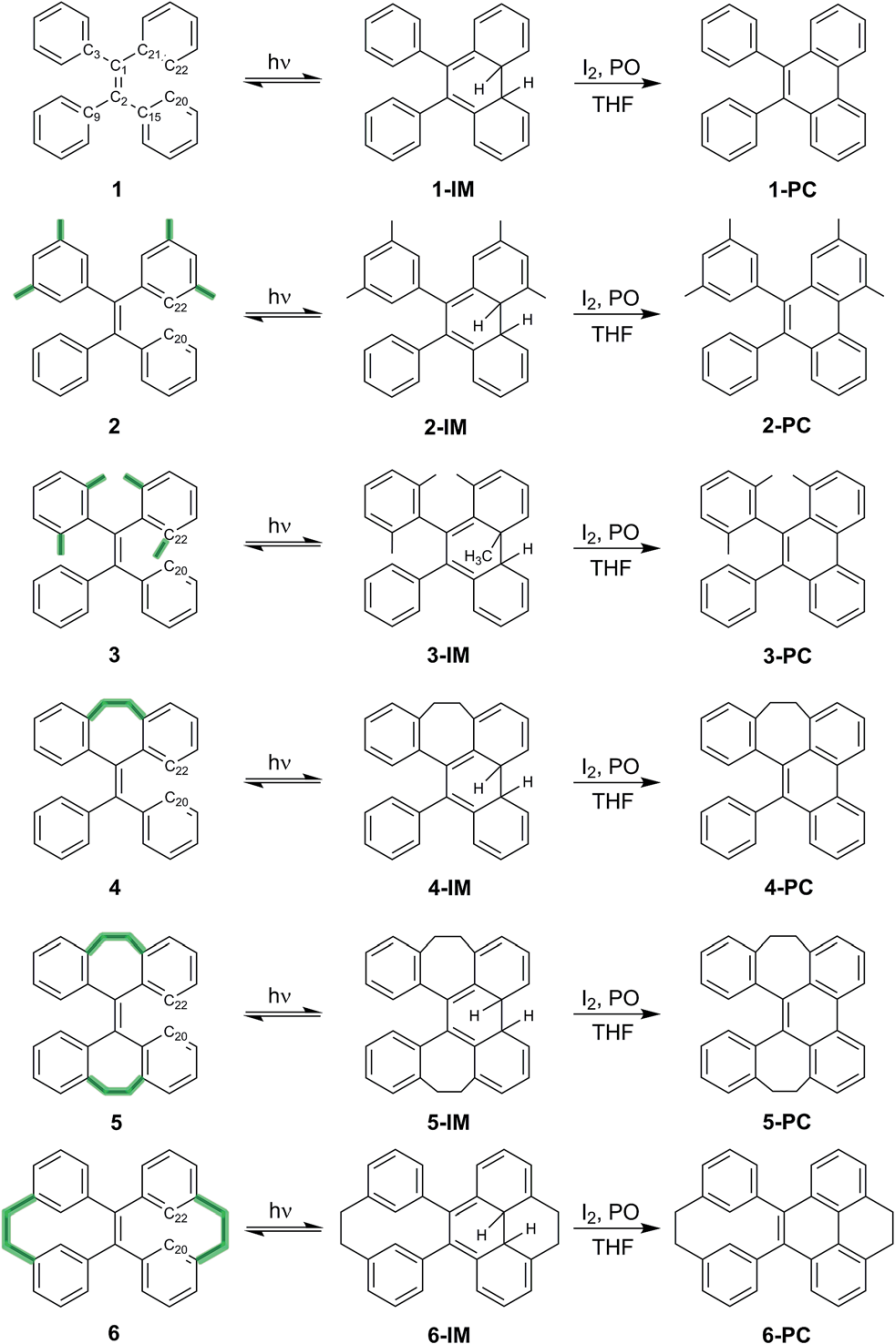
TPE derivatives **1–6** with increased structural rigidity and their transformation upon UV irradiation into the corresponding intermediates (**1-IM–6-IM**) and subsequent oxidation to isolable and fully characterized photocyclized phenanthrene derivatives **1-PC–6-PC** (PO: (±)-propylene oxide).

In our work to restrict certain relaxation channels in the excited state and investigate their role in the AIE effect, we synthesized a set of TPE-based derivatives, **1–6** (Scheme 1), with varying degrees of rigidity, studied their photophysical and AIE properties, and applied a combination of DFT calculations and ultrafast time-resolved spectroscopy to probe and resolve the excited-state dynamics and photocyclization processes in them. We have found state-dependent coupling of the two major dynamic motions (phenyl torsion and C=C twisting) in **1–6**, with the formation of a photocyclized intermediate in all compounds on different timescales, and the dominant relaxation channels responsible for the AIE effect (or its absence) in every particular case being strongly structure-dependent. The implications of these observations for the working mechanism of AIE are fully explained.

## Results and discussion

### Synthesis and characterization of TPE derivatives

TPE derivatives **1–6** with increased structural rigidity and their corresponding photocyclized phenanthrene derivatives **1-PC–6-PC** were synthesized according to the synthetic routes shown in Scheme 1. The isolated compounds were characterized by ^1^H-NMR, ^13^C-NMR and high resolution mass spectrometry. The structures were determined by single-crystal X-ray diffraction (see the ESI† for details), which confirms their correct structures.

### Structural rigidity, AIE properties and photocyclization

Scheme 1 shows the structures of TPE derivatives **1–6** in the order of increasing structural rigidity. While the molecular structures of **1–3** and **6** in their respective single crystal unit cells have two different enantiomers with different propeller-like orientations of phenyl groups, molecules of **4** and **5** with ethylene tethers (–CH_2_–CH_2_–) between geminal phenyl groups display only one type of phenyl orientation (Fig. S26†). To explore and quantify their different structural rigidities, we have investigated computationally the geometry changes^36^ in **1–6** upon transition from the ground state to the first excited state as shown in Table 1 (see ESI† Section 7.5 for more details). Upon photoexcitation in solution and in the solid state, the central ethylenic C_1_–C_2_ bond in **1–6** elongates (*i.e. d*_C_1_–C_2__), giving rise to what we will refer to from now on as a quasi C=C bond. In contrast, the bonds connecting the peripheral phenyl groups with the C_1_–C_2_ bond shorten (*e.g. d*_C_2_–C_15__) to fall into the bond length range that is between that of a typical single C–C bond and a normal C=C double bond. The twisting of the C=C bond and torsion of the phenyl rings in **1–6** are analyzed metrically in terms of the dihedral angles *τ*_C_21_–C_1_–C_2_–C_15__ and *τ*_C_1_–C_2_–C_15_–C_20__, respectively. For **1–6** in solution, each molecule has its unique set of restricted degrees of freedom with varying degrees of rigidity. The absolute change of the dihedral angle around the C_1_–C_2_ bond (|Δ*τ*_C_21_–C_1_–C_2_–C_15__|) in TPE derivatives in solution upon excitation decreases in the order **1–3** and **5–6** (Table 1), which is consistent with the increase of structural rigidity in that order. The smallest structural changes upon photoexcitation with only 0.03 Å C_1_–C_2_ bond elongation and less than 9° twisting are predicted for **6**, making it the most rigid structure, indicating that two vicinal ethylene bridges (–CH_2_–CH_2_–) do effectively restrict the motion of the C=C bond. We note that this trend persists for the changes of the dihedral angles of the phenyl torsion (|Δ*τ*_C_1_–C_2_–C_15_–C_20__|) in **1–3** after photoexcitation. Two ethylene tethers in geminally locked structure **5** and vicinally locked structure **6** have similar restriction effects on the phenyl torsion with the smallest change of the dihedral angles (*ca.* 20°).

**Table 1:** Geometry changes upon transition from the ground state to the first excited state, and the photophysical and AIE properties of TPE derivatives **1–6**

	Geometry changes between *S*_0_ and *S*_1_ in soln^*a*^									Abs^*b*^ *λ*_max_ (nm)	PL *λ*_em_ (nm)		QY (%)	
	*d*_C_1_–C_2__	*d*_C_2–_C_15__	Δ*τ*_C_21–_C_1–_C_2–_C_15__		Δ*τ*__C1–_C_2–_C_15–_C_20__		*d*_C_20–_C_22__				Soln^*b*^	Film	Soln^*b*^	Solid
	*S*_0_	*S*_1_	*S*_0_	*S*_1_	|Δ(*S*_0_ – *S*_1_)|	|Δ(*S*_0_ – *S*_1_)|	*S*_0_	*S*_1_						
**1**	1.35	1.47	1.49	1.44	56.80	29.04	3.25	3.64	**1**	239; 309	(358, 375, 396),^*c*^ 474	445	0.8	24.1
**2**	1.35	1.47	1.49	1.44	39.59	25.19	3.23	3.31	**2**	242; 311	(383),^*c*^ 472	466	0.6	30.2
**3**	1.36	1.46	1.49	1.45	33.96	20.77	3.32	3.49	**3**	244; 314	473	460	60.0	97.6
**5**	1.35	1.47	1.49	1.46	11.57	20.35	3.44	2.96	**4**	262	391	393	1.0	1.3
**6**	1.37	1.40	1.50	1.45	8.96	22.37	2.60	1.70	**5**	268	389	384	0.9	16.5
									**6**	262; 368	397	462	0.5	0.7

^*a*^Geometry changes in THF solution were calculated using DFT with the M062X functional and 6-311G (d) basis set. Bond lengths (*d*) are given in Å and angles in °. The ethylenic C_1_–C_2_ bond twisting angle is defined as the dihedral angle *τ*_C_21_–C_1_–C_2_–C_15__. The phenyl torsion angle is defined as the dihedral angle *τ*_C_1_–C_2_–C_15_–C_20__. The distance between the two carbon atoms *d*_C_20_–C_22__ is analyzed to investigate the possibility of the formation of photocyclized species. Despite multiple trials, the geometry optimization of **4** in the first excited state in solution did not converge. For more information, see ESI Section 7.5.

^*b*^All measurements of compounds **1–6** were done in THF solution (10^–5^ M) in open air. The UV/vis absorption spectra of **1**, **3** and **5** in solution, the photoluminescence (PL) spectra of **1** and **3** in the solid state, and the fluorescence quantum yields (QY) of **1** and **3** in solution have already been published elsewhere.^16^,^21^,^37^ For the sake of consistency, we repeated the measurements for these compounds on the same spectrometer. See ESI Section 5 for details.

^*c*^The extra peaks in the PL spectra of **1** and **2** upon first time excitation.

The RIM mechanism dictates a reciprocal correlation between the rigidification of the structure and possible AIE properties. For molecules **1–6**, compounds **1** and **2** are archetypal TPE-based AIEgens showing intense turn-on fluorescence upon aggregation (see the fluorescence quantum yields (QY) in Table 1), while **3** shows unexpectedly highly fluorescence in solution, and becomes even more fluorescent upon aggregation making it AEE (aggregation-enhanced emission)-active. Contrary to our expectations, the significantly more rigid structures **4–6** have low fluorescence quantum yields in solutions, among which **5** is an AIEgen, while **4** and **6** are not AIE-active. These findings clearly demonstrate that structural rigidity is not directly correlated with the AIE properties and the RIM paradigm needs to be revisited.

The UV-vis and fluorescence emission maxima of **1–6** in dilute solutions and film are shown in Table 1. While **1–3** in solution display similar UV absorption peaks at around 310 nm, the UV absorption maxima of **4** and **5** are markedly blue shifted (<270 nm), and the absorption maximum of **6** is notably red shifted (368 nm). All compounds **1–6** in dilute solutions undergo photocyclization upon UV excitation (ESI† Section 5), during which the bond formation between atoms C_20_ and C_22_ takes place (Scheme 1). Compared to the emission peaks of **1–6** in film, extra peaks at around 358, 375, and 396 nm in the PL spectrum of a dilute solution of **1** and around 383 nm for **2** as well as discernable asymmetry of the emission peaks in dilute solutions of **4–6** upon first-time photoexcitation indicate the formation of new species which could be further identified as photocyclized (diphenylphenanthrene) derivatives.^38^ In dilute solution upon prolonged excitation, **3** undergoes photocyclization, as evidenced by the emergence of similar new emission peaks at 361, 378 and 400 nm (ESI† Section 5), suggesting that the steric hindrance of the methyl groups in the *ortho*-positions of the phenyl rings cannot preclude photocyclization. The above observations reveal that photocyclization in TPE-based derivatives **1–6** is an important event in the relaxation process after photoexcitation.

### The coupling relationship of molecular motions

**Fig. 1:**
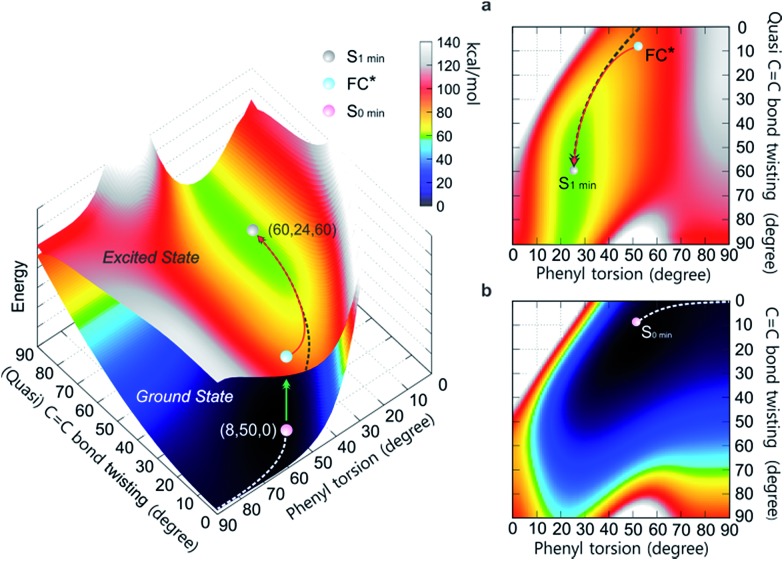
The PES of **1** in the ground state and excited state as a function of the (quasi) C=C bond twisting and phenyl torsion dihedral angles (defined in Table 1). The lower surface shows the ground state PES, and the upper one depicts the first excited state PES. (a) Top view of the first excited state PES. (b) Top view of the ground state PES. The MEPs in *S*_0_ (white dashed line) and *S*_1_ (black dashed line) are marked on the PES.

To differentiate between several possible dominant relaxation channels (*i.e.* quasi C=C bond twisting, phenyl torsion and photocyclization) in **1–6** upon excitation, and to determine their contribution to the photophysics of the AIE mechanism, probing the molecular dynamics in the excited state is necessary. Prior to this, developing a quantitative understanding of the coupling relationship between the relaxation channels directly related to the molecular motions is imperative. To that end, we have constructed the 3D potential energy surface (PES) of **1** in solution as a function of the aforementioned modes using DFT calculations (Fig. 1). In the ground state, the absolute minimum of **1** (*S*_0,min_, denoted as (8, 50, 0)) has a molecular geometry corresponding to *ca.* 8° twisting angle along the C=C bond twisting mode, *ca.* 50° torsion along the phenyl torsional coordinate, and potential energy whose value is set to 0 kcal mol^–1^. The coupling between the C=C bond twisting and phenyl torsion along the minimum energy path (MEP) of **1** on the ground state PES (Fig. 1b) is clearly exemplified by the increase of the dihedral angle of phenyl torsion from 50° to 90° actuating slight changes in the C=C bond twisting (<9°) and potential energy (<7 kcal mol^–1^), indicating that under standard conditions the torsion of the phenyl rings dominates the ground state dynamics in **1**. This is further corroborated by the thorough analysis of the intrinsic reaction coordinate (IRC) calculations (ESI† Section 7.2.1) showing that the change of the dihedral angle of phenyl torsion is linearly coupled with the small change of the C=C bond twisting (Fig. S56†).

Following the MEP along the C=C bond twisting mode in **1** in the ground state from one minimum energy geometry through the highly twisted transition state to the other minimum energy geometry (ESI† Section 7.2.2), we note that the C=C bond twisting is associated with significant changes in the C=C bond length (∼0.07 Å), and a marked increase in potential energy (∼60 kcal mol^–1^). In the excited state, the elongation (∼0.12 Å) and partial loss of the double bond character of the C=C bond in **1** triggers the C=C bond twisting as the dominant motion which is coupled with the phenyl torsion (Fig. 1a). The MEP along the C=C bond twisting mode in **1** on the first excited state PES reveals that on the way from the Frank–Condon (FC*) geometry to the minimum energy geometry (*S*_1,min_), the quasi C=C bond twists *ca.* 50°, which is accompanied by the phenyl torsion with an amplitude of less than 25°. Although their coupling relationship in the excited state is well fitted by the quadratic function Δ twisting = 0.0671 (Δ torsion)^2^ – 3.9048 Δ torsion – 2.7952, the PES around the *S*_1,min_ is rather shallow (Fig. 1a, green area at *ca.* 60 kcal mol^–1^ potential energy) and simple harmonic oscillations^39^ of the quasi C=C bond twisting may easily take place with the damping process, resulting in the relaxation to the *S*_1 min_ (ESI† Section 7.3).

### The observation of intermediates

A characteristic ultrafast transient absorption (TA) spectrum corresponds to the statistically most probable geometry of the molecules on a specific timescale in the excited state. Thus, the photoinduced excited state structural dynamics associated with molecular motions is reflected in the evolution of TA spectra.

**Fig. 2:**
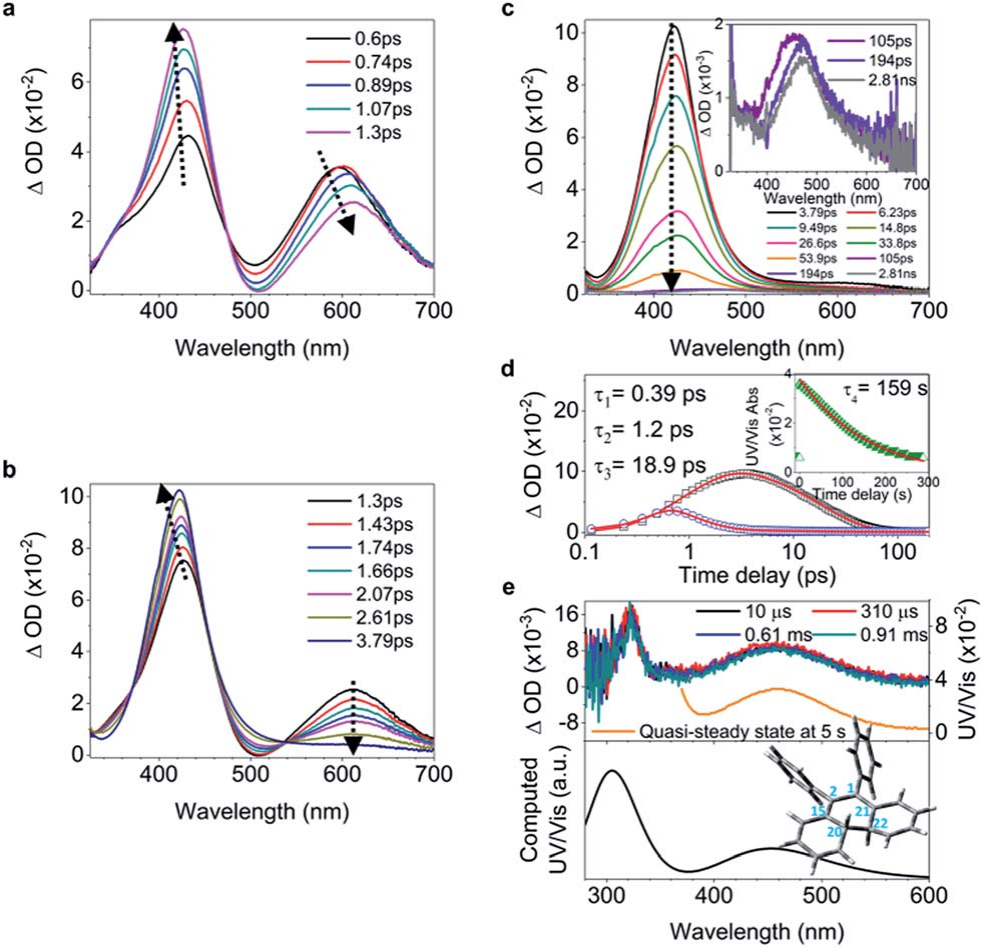
Excited state dynamics of **1** in solution observed by ultrafast time-resolved spectroscopy. (a–c) Femtosecond transient absorption (fs-TA) spectra. (d) Kinetic traces at 430 nm (black square) and 600 nm (blue circle) with the respective fits (red lines, solid) determined using a global analysis that used three exponential functions convoluted with a 120 fs (full-width at half maximum) Gaussian-shaped pulse. The inset shows the lifetime of the photocyclized intermediate **1-IM** analyzed by monoexponential fitting of the decay of UV/vis absorption (ESI† Section 6.1.7). (e) Nanosecond transient absorption (ns-TA) spectra (upper panel). UV/vis spectrum of **1-IM** in MeCN acquired after excitation of **1** in MeCN at 254 nm for 1 min with a hand-held UV lamp and then without UV/vis excitation for 5 s (upper panel, orange line). The inset shows the calculated electronic absorption spectrum (lower panel) of the optimized structure of **1-IM**.

Upon UV light irradiation, the electron density in **1–6** flows mainly from the central C=C bond to the adjacent C–C (Ph) bonds (Fig. S54†), concomitantly resulting in the elongation of the C=C bond and shortening of peripheral bonds, which is accompanied by C=C bond twisting coupled with phenyl torsion. This process in **1** is reflected in the excited state absorption spectra shown in Fig. 2a. On the sub-picosecond timescale (0.6–1.3 ps), **1** transitions from *S*_*n*_ to the emissive state *S*_1_ due to the weak stimulated emission as evidenced by the band at 500 nm with a negative amplitude at 1.3 ps.^40^,^41^ The build-up of the band at 600 nm with its red-shift to 613 nm and the growing intensity of the band at 430 nm are attributed to the central C=C bond elongation^41^ associated with the quasi C=C bond twisting. On the picosecond timescale between 1.3 ps and 3.79 ps (Fig. 2b), sequential depopulation of the band at 613 nm with the increasing band at 428 nm is attributed to the quasi C=C bond twisting,^41^,^42^ meaning that the transients in the emissive state revert back to the state in which the shortening of the elongated C=C bond took place. After 3.79 ps (Fig. 2c), the decay of the band at 422 nm assigned to phenyl torsion^43^ takes place, during which a new species forms, and the band at 465 nm is clearly observed after 105 ps. Global fitting analysis of the decay kinetics at all wavelengths yields three time constants: 0.39 ps, 1.2 ps and 18.9 ps (Fig. 2d), which correspond to the lifetimes of the initially dominant motion of C=C bond elongation (*τ*_1_), the sequential dominant motion of the quasi C=C bond twisting (*τ*_2_), and the last motion dominated by the phenyl torsion (*τ*_3_), respectively. It should be noted that both processes corresponding to *τ*_1_ and *τ*_2_ are coupled with phenyl torsion, as proved by the band at 430 nm and predicted by the calculation (Fig. 1). The monoexponential fitting of the decay of UV/vis absorption at 465 nm yields a lifetime of 159 s (Fig. 2d inset and S45†), which is attributed to the lifetime of the photocyclized intermediate **1-IM** (*τ*_4_) having a new bond connecting atoms C_20_ and C_22_ directly (Fig. 2e). The longevity of **1-IM** with two characteristic absorption bands at 320 nm and 465 nm indicates that it is in its singlet ground state. This is further confirmed by ns-TA spectroscopy, the UV/vis absorption spectrum of the quasi-steady state at 5 s and the consistent calculated UV/vis spectrum (Fig. 2e), indicating that the formation of the photocyclized intermediate is a possible non-radiative relaxation channel. Further characterization of **1-IM** was carried out by transient Raman spectroscopy which confirmed the match between the experimentally obtained and computed Raman spectra of **1-IM** (Fig. S36†).

### Fluorescence prevails as the rotation of the elongated C=C bond slows down

**Fig. 3:**
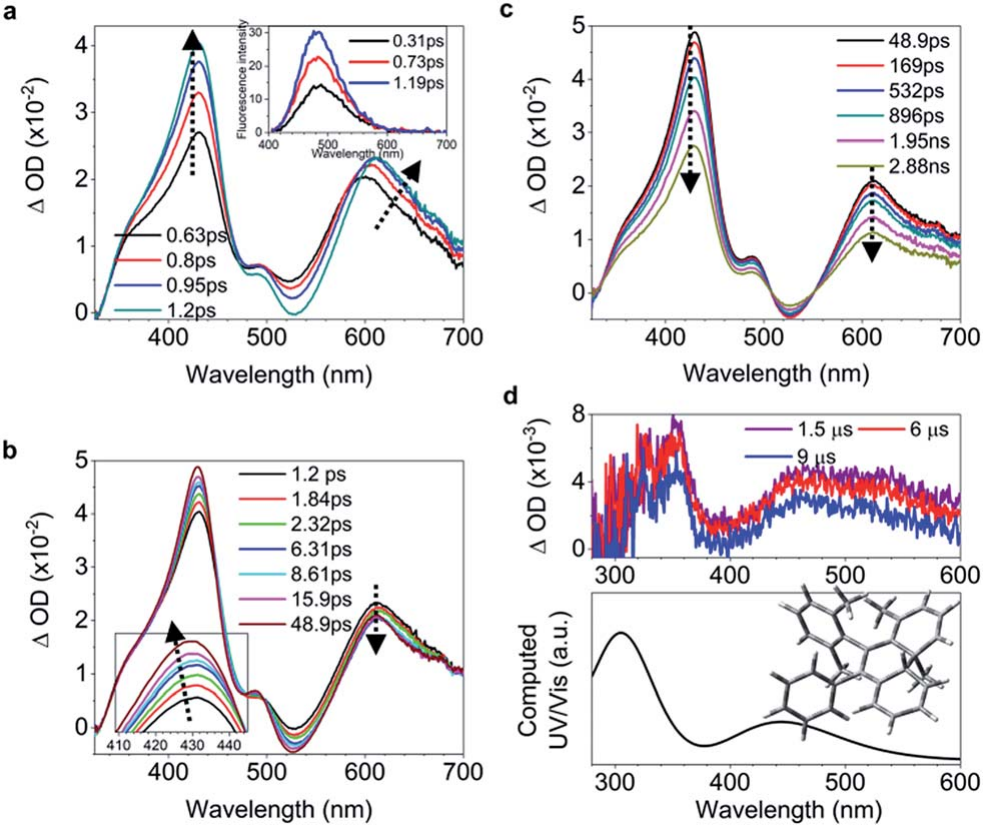
Excited state dynamics of **3** in solution observed by ultrafast time-resolved spectroscopy. (a–c) Fs-TA spectra and femtosecond time-resolved fluorescence spectra (inset in (a)). (d) Ns-TA spectra (upper panel). The inset shows the calculated electronic absorption spectrum (lower panel) of the optimized structure of the photocyclized intermediate **3-IM**.

While **2** undergoes the formation of a photocyclized intermediate and has similar ultrafast dynamic features as observed in **1** (ESI† Section 6.1.2), **3** begins to fluoresce on the subpicosecond timescale and its fluorescence intensity reaches its maximum at 1.19 ps (Fig. 3a inset and S40c†). In accordance with the transient fluorescence spectra (Fig. 3a inset, peak at around 480 nm), the dip at 477 nm in the fs-TA spectra on the timescale between 0.63 ps and 1.2 ps (Fig. 3a) originates from the stimulated emission, and the red shift of the band at 600 nm to 610 nm is attributed to the C=C bond elongation corresponding to the population of transients in the emissive state (most likely *S*_1,min_). In contrast to **1–2**, the decay of the band at 610 nm with growing intensity of the band at 430 nm accompanied by a blue shift (Fig. 3b) takes a long time (between 1.2 ps and 48.9 ps), with a time constant of 11.2 ps derived from the global fitting analysis (ESI† Section 6.1.3), indicating that the emissive state of the molecule is relatively stable and thus fluorescence prevails as the rotation (larger degree of twisting, Table 1) of the elongated C=C bond slows down. This is probably due to the steric hindrance from the *ortho*-position substituents in **3**. The depopulation of the band at 428 nm (Fig. 3c) with a time constant of 4.07 ns (Fig. S40b†) indicates that the molecules of **3** relaxing to the ground state either revert back to the original ground state structure, or form the photocyclized intermediate (Fig. 3d, upper panel). Although the stabilization of the emissive state of **3** with a twisted structure in the excited state increases the distance between atoms C_20_ and C_22_ (Table 1) and thus inhibits the formation of the intermediate, the photocyclized intermediate **3-IM** still forms as observed in the ns-TA spectra, and its structure is further confirmed by comparison with the computed UV/vis spectrum (Fig. 3d).

### Ultralong-lived intermediates

**Fig. 4:**
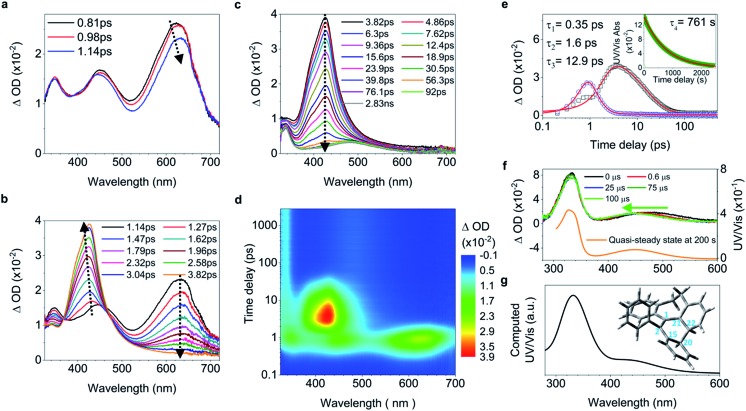
Excited state dynamics of **4** in solution observed by ultrafast time-resolved spectroscopy. (a–c) Fs-TA spectra. (d) Contour plots of the time-resolved absorption spectroscopic responses. (e) Kinetic traces at 426 nm (black square) and 630 nm (blue circle), and the corresponding fits (red lines, solid) determined using a global analysis making use of three exponential functions convoluted with a 120 fs (full-width at half maximum) Gaussian-shaped pulse. The inset shows the lifetime of the photocyclized intermediate **4-IM** analyzed by monoexponential fitting of the decay of UV/vis absorption (ESI† Section 6.1.7). (f) Ns-TA spectra (upper panel, solid lines). UV/vis spectrum of **4-IM** in MeCN acquired after excitation of **4** in MeCN at 254 nm for 30 s with a hand-held UV lamp and then without UV/vis excitation for 200 s (orange line and Fig. S31C†). (g) The calculated electronic absorption spectrum of the optimized structure of the photocyclized intermediate **4-IM**.

The sequentially dominant motions (C=C elongation and quasi C=C bond twisting) of **4** upon excitation have similar dynamics and time constants to those of **1–2** (Fig. 4a, b, and e, and ESI† Section 6.1.8). After 3.82 ps (Fig. 4c and d), the band at 485 nm emerges during the depopulation of the band at 430 nm, meaning that a new species forms during the decay process of phenyl torsion. The decay process has a shorter time constant of 12.9 ps as compared to **1–2**, indicating that the ethylene bridge in **4** restricts the freedom of torsion for the two phenyl rings and thus shortens the timescale of the bond formation between C_20_ and C_22_. On the nanosecond timescale and beyond, the band at 485 nm continues to blue-shift to 450 nm until 1.0 μs (Fig. S41a† and 4f, upper panel) due to the structural relaxation of the photocyclized species (Fig. S63†). The intermediate **4-IM** has an ultralong lifetime of 761 s as analyzed by the decay of the UV/vis absorption of its quasi-steady state at 450 nm (Fig. 4e and f, and S46†) and its structure is further confirmed by the transient Raman spectrum (Fig. S41b†).

### Ultrafast formation of intermediates

**Fig. 5:**
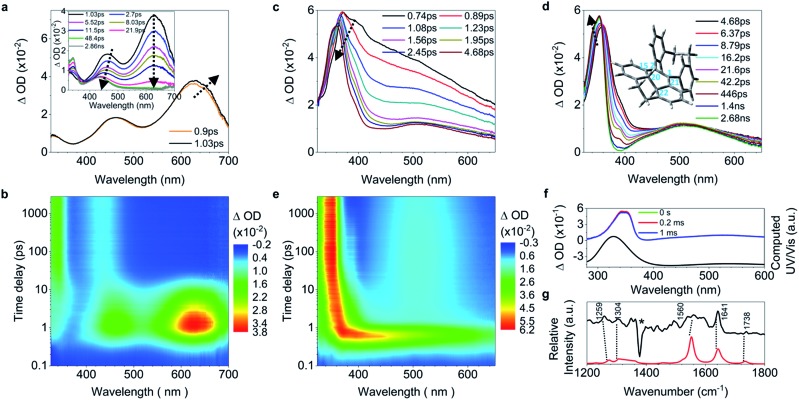
Excited state dynamics of **5** and **6** in solution observed by ultrafast time-resolved spectroscopy. (a) Fs-TA spectra of **5**. (b) Contour plots of the time-resolved absorption spectroscopic responses of **5**. (c and d) Fs-TA spectra of **6**. The optimized structure of the photocyclized intermediate (**6-IM**) is shown in the inset in (d). (e) Contour plots of the time-resolved absorption spectroscopic responses of **6**. (f) Ns-TA spectra of **6** (green, red and blue lines) and the calculated electronic absorption spectrum (black line) of **6-IM**. (g) The nanosecond transient resonance Raman (ns-TR^2^) spectrum of **6-IM** (black line) is compared to the computed Raman spectrum (red line). The star represents the bands caused by solvent subtraction artifacts.

In the ultrafast spectra, the initial C=C bond elongation in **5** upon excitation in solution is reflected by the population of the 628 nm band with its red shift before 1.03 ps (Fig. 5a). Thereafter, both bands at 462 nm and 630 nm (Fig. 5a, inset) depopulate, meaning that the decay of the quasi C=C bond twisting and phenyl torsion take place at the same time. Concomitantly, the intensified red-shifted band at 330 nm indicates that a new species is formed, which is further proved by the emergence of a band at 438 nm (Fig. 5b) attributed to the long-lived photocyclized intermediate (Fig. S42, S43 and S47†). While the process of the C=C elongation in **6** upon excitation was not observed in the fs-TA spectra because of its negligible increase of only 0.03 Å (Table 1), the formation of the photocyclized intermediate takes place directly on the subpicosecond timescale during the initial depopulation of the whole TA spectra with a time constant of 0.4 ps (Fig. 5c and S44†). This is further evidenced by the build-up of the band at 510 nm at 1.08 ps (Fig. 5c and e) and the computational prediction that **6** upon excitation has a short *d*_C_20_–C_22__ distance (1.70 Å). This means that the vicinally bi-locked structure of **6** restricts the C=C elongation and the freedom of torsion for all phenyl rings with short distances, which facilitates the ultrafast formation of the photocyclized intermediate on the subpicosecond timescale, and thus **6** barely fluoresces in solution. After 4.68 ps, the photocyclized intermediate of **6** undergoes structural relaxation (Fig. 5d), and its quasi-steady state structure, **6-IM**, is confirmed by the experimentally observed ns-TA and Raman spectra and computationally predicted spectra (Fig. 5f and g), in which bands at 345 nm and 525 nm are identified as characteristic absorption peaks of **6-IM**, with the vibrational feature at 1641 cm^–1^ mainly attributed to the stretching motion of C_20_–C_22_, and those at 1304 cm^–1^ and 1259 cm^–1^ mainly ascribed to the rocking and wagging modes of C_22_–H and C_20_–H. The identity of all intermediate species was further indirectly confirmed by isolating and characterizing their oxidized analogues (ESI† Section 2.4).

### AIE mechanism through the lens of ultrafast spectroscopy

**Fig. 6:**
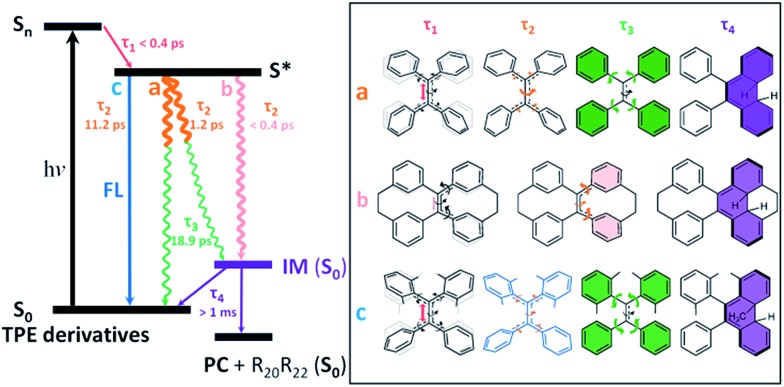
Schematic representation of ultrafast processes responsible for the AIE effect in TPE derivatives. The timescales of the dominant motions of various parts of the molecule are color-coded in red (subpicosecond), blue (subpico- to nanosecond), pink (subpico- to picosecond), brown (picosecond), green (pico- to nanosecond) and purple (millisecond to second/minute). Three different pathways for the decay of S* are represented by non-radiative decay channels (a) and (b), and a fluorescence decay channel (c). Examples of the molecules with a dominant decay channel are shown in the box on the right. R_20_R_22_ represents substituents on atoms C_20_ and C_22_ in TPE derivatives. *τ*_1_ is the time constant of the motion dominated by the elongation of the C=C bond associated with quasi C=C bond twisting. *τ*_2_ is the time constant of the motion dominated by the quasi C=C bond twisting coupled with phenyl torsion. *τ*_3_ is the time constant of the motion dominated by the phenyl torsion. *τ*_4_ is the lifetime of IM.

After photoexcitation, **1–6** in solution reach the hot emissive excited state S* (most likely *S*_1,min_, Fig. 6) upon relaxation *via* the dominant motion of the C=C bond elongation with a time constant of *τ*_1_ < 0.4 ps (Table S6†). Due to the structural rigidity, the *τ*_1_ components in **1–6** undergo relaxation *via* three possible channels with one of them being the dominant relaxation pathway (Fig. 6). They either do not fluoresce due to (a) the quasi C=C bond twisting with a time constant of 1–2 ps (*e.g.* flexible structures **1**, **2**, and **4**), or (b) the ultrafast formation of the photocyclized intermediate (IM) on the sub-picosecond timescale (*e.g.* rigid structures **5**, **6**), or they do fluoresce (c) due to the structural stability of the emissive state of the molecule on a relatively longer timescale (**3**). Thus, the dynamics of the quasi C=C bond twisting (*τ*_2_) with the possibility of intermediate formation in S* is the key factor that determines the fluorescence properties of TPE derivatives in solution. When the non-radiative relaxation pathways for TPE derivatives in solution are blocked in the solid state (ESI† Section 6.2), and no other non-radiative relaxation channels open up, enhanced fluorescence and thus the AIE properties will emerge. After *τ*_2_, transients of all compounds finally revert back to the ground state through the phenyl torsion (*τ*_3_) *via* two different routes: relaxation to the original ground state structure and formation of a singlet ground state IM with a long-lived lifetime (*τ*_4_) that either (**1**) reverts back to the original compound or (**2**) is oxidized to form the corresponding photocyclized product (PC) (ESI† Section 7.4). Intermediate formation from **1–6** upon excitation in solution was experimentally confirmed by the outcome of the photochemically induced reactions (ESI† Section 2.4).

## Experimental

### Synthesis, photophysics and computational calculations

Detailed information on the synthesis and characterization of **1–6** and their photoinduced cyclization products **1-PC–6-PC** is given in ESI† Sections 1–4. The UV/vis absorption spectra were measured on a UV/vis spectrometer (Shimadzu, UV-2600, Japan). The steady state PL spectra were collected on a Horiba Fluoromax-4 spectrofluorometer. The absolute fluorescence quantum yields were measured by using a Hamamatsu quantum yield spectrometer, C11347 Quantaurus_QY. ESI† Section 5 gives more details on the photophysical properties. The MO calculations were carried out using the Gaussian 16 software package; all other computational calculations, including the electron density difference between *S*_1-FC_ and *S*_0_, the intrinsic reaction coordinates through the transition states of phenyl torsion and ethylenic C=C bond twisting, the potential energy hypersurface, the Gibbs free energy, the geometry optimization of TPE derivatives in solution and in the solid state (both in the ground state (*S*_0_) and excited state (*S*_1_)), and the UV/vis and Raman spectra of photocyclized intermediates **1-IM–6-IM**, were performed at the DFT level of theory using the M062X functional^44^ and 6-311G (d) basis set^45^ as implemented in the D0.1 version of the Gaussian 09 software package. The scaling factor for all UV/vis and Raman calculations was 1.000. DFT computational details are given in ESI† Sections 6 and 7.

### The ultrafast time-resolved spectroscopy

Femtosecond transient absorption (fs-TA), nanosecond transient absorption (ns-TA), femtosecond time-resolved fluorescence (fs-TRF), nanosecond transient resonance Raman (ns-TR^2^) and time-resolved resonance Raman (ns-TR^3^) experiments were carried out using the same experimental setups and methods as described previously.^46^ The pump wavelength employed for the fs-TA and fs-TRF measurements was 267 nm, and the pump wavelength employed for the ns-TA and ns-TR^3^ measurements was 266 nm. Two probe wavelengths (355 nm and 309.1 nm) were used for the ns-TR^3^ experiment. The ns-TR^2^ data were obtained by using the difference between the Raman spectra obtained at different power values of the 266 nm pump laser. Compounds **1–6** in MeCN solution were studied in a flow-through 2 mm path-length cuvette with an absorbance of 0.5 at 267 nm throughout the data acquisition. More details are available in ESI† Section 6.

## Conclusions

TPE-based derivatives **1–6** with varying rigidities and photophysical properties have been synthesized and their ultrafast excited-state relaxation dynamics and its role in the AIE process were studied. We found that C=C bond elongation, quasi C=C bond twisting, phenyl torsion and photocyclization are the sequentially dominant processes in TPE derivatives upon photoexcitation in solution. Their state-dependent coupling motions derived from ultrafast time-resolved spectroscopy show that (a) the lifetime of the species with electronic configuration conducive to fluorescence (mostly likely the lowest vibrational state of *S*_1_) is the determining factor for the observed fluorescence quantum yields, (b) in less rigid structures, the rotation of the elongated C=C bond is the dominant motion that eventually leads to the non-radiative decay (*e.g.* AIE-active TPE), and (c) in the more rigid structures, the torsion of phenyl rings will dominate the relaxation dynamics and, if possible, lead to the formation of singlet ground state photocyclized intermediates (*e.g.* AIE-active or AIE-inactive bi-locked TPE derivatives). Thus, while the dominant intramolecular motion that serves as a non-radiative relaxation channel being restricted upon aggregation in every particular case is strongly structure-dependent, counter-intuitively, the AIE effect (or absence thereof) is not directly related to rigidity as seemingly implied by the RIM paradigm.

## Conflicts of interest

There are no conflicts to declare.

## Supplementary Material

Supplementary information

Crystal structure data
