# Supplementary material for: Deciphering the working mechanism of aggregation-induced emission of tetraphenylethylene derivatives by ultrafast spectroscopy
Source: Chem Sci. 2018 Apr 24;9(20):4662–70. doi: 10.1039/c8sc01170b (PMC5969501; doi:10.1039/c8sc01170b)
Supplement: Supplementary file 1 [file SC-009-C8SC01170B-s001.pdf]

Electronic Supplementary Material (ESI) for Chemical Science.
This journal is © The Royal Society of Chemistry 2018

Electronic Supplementary Information

Deciphering the working mechanism of aggregation-induced emission of tetraphenylethylene derivatives by ultrafast spectroscopy

Yuanjing Cai,[‡abc] Lili Du,[‡dg] Kerim Samedov,[‡e] Xinggui Gu,[‡a] Fei Qi,[‡f] Herman H. Y. Sung,[a] Brian O. Patrick,[e] Zhiping Yan,[d] Xiaofang Jiang,[b] Haoke Zhang,[a] Jacky W. Y. Lam,[a] Ian D. Williams,[a] David Lee Phillips,*[d] Anjun Qin,*[b] and Ben Zhong Tang *[abc]

[a] Department of Chemistry, Hong Kong Branch of Chinese National Engineering Research Center for Tissue Restoration and Reconstruction, The Hong Kong University of Science & Technology, Clear Water Bay, Kowloon, Hong Kong SAR, China. E-mail: tangbenz@ust.hk.
[b] Center for Aggregation-Induced Emission, NSFC Center for Luminescence from Molecular Aggregates, SCUT-HKUST Joint Research Institute, State Key Laboratory of Luminescent Materials and Devices, South China University of Technology, Guangzhou 510640, China. E-mail: msqinaj@scut.edu.cn.
[c] HKUST Shenzhen Research Institute, No.9 Yuexing 1st RD, South Area, Hitech Park Nanshan, Shenzhen 518057, China
[d] Department of Chemistry, The University of Hong Kong, Pokfulam Road, Hong Kong SAR, China. E-mail: phillips@hku.hk
[e] Department of Chemistry, University of British Columbia, 2036 Main Mall, Vancouver, British Columbia, Canada V6T 1Z1.
[f] Institute of Computational and Theoretical Studies & Department of Physics, Hong Kong Baptist University, Hong Kong SAR, China.
[g] Institute of Life Sciences, Jiangsu University, Zhenjiang 212013, China.
[‡] These authors contributed equally to this work.

Table of Contents

1. TPE derivatives S3
2. Synthetic protocols of TPE derivatives S4
   - 2.1 Synthesis of **2** S4
   - 2.2 Synthesis of **4** S5
   - 2.3 Synthesis of **6** S6
   - 2.4 Photochemical reactions of **1-4** and **6** S8
3. ^{1}H NMR and ^{13}C NMR spectra S12
4. Single crystal X-ray crystallography S24
5. Photophysical properties S27
   - 5.1 Photophysical properties of **1** and **1-PC** S29
   - 5.2 Photophysical properties of **2** and **2-PC** S30
   - 5.3 Photophysical properties of **3** and **3-PC** S32
   - 5.4 Photophysical properties of **4** and **4-PC** S33
   - 5.5 Photophysical properties of **5** and **5-PC** S35
   - 5.6 Photophysical properties of **6** and **6-PC** S36
   - 5.7 The absolute quantum yields and AIE properties of TPE derivatives S37
6. Ultrafast time-resolved spectroscopy S38
   - 6.1 Ultrafast time-resolved spectroscopy of TPE derivatives in solution S40
     - 6.1.1 fs-TA and ns-TR^2 spectra of **1** S40
     - 6.1.2 fs-TA, ns-TA and ns-TR^2 spectra of **2** S42
     - 6.1.3 fs-TA spectra and time-resolved fluorescence spectra of **3** S44
     - 6.1.4 ns-TR^3 spectra of **4** S45
     - 6.1.5 ns-TA and ns-TR^3 spectra of **5** S46
     - 6.1.6 EADS spectra of **6** S48
     - 6.1.7 The lifetime of the photocyclized intermediates **1-IM** - **6-IM** S49
     - 6.1.8 The excited state dynamic processes and time constants of **1-6** S51
   - 6.2 The fs-TA spectra of **1-6** in thin film S52
7. Computational studies S54
   - 7.1 The electron density difference between $S_{1\text{-}FC}$ and S_0 of TPE derivatives S54
   - 7.2 The IRC of **1** through the transition state of Ph torsion and C=C twisting in ground state S55
   - 7.3 The potential energy hypersurface of **1** in ground state and excited state S62
   - 7.4 The Gibbs free energy and MOs of the TPE derivatives S64
     - 7.4.1 The Gibbs free energy of the TPE derivatives S64
     - 7.4.2 The MOs of the TPE derivatives S70
   - 7.5 The geometry changes of TPE derivatives at S_0 and S_1 in solution and in solid state S75
   - 7.6 The optimized geometry of TPE derivatives S86
8. The photos of TPE derivatives S134
9. References S134

1. TPE derivatives

In the ground state, the bonds drawn **in bold** have lengths between 1.440-1.475 Å, which is longer than a normal C=C bond but shorter than a normal C-C bond. Metric parameters of the intermediates (IM) are derived from the theoretical calculations, while geometries of the photo-cyclized compounds (PC) were obtained from the X-ray single crystal structure analysis. Compounds in blue are newly made and are reported in this manuscript for the first time.

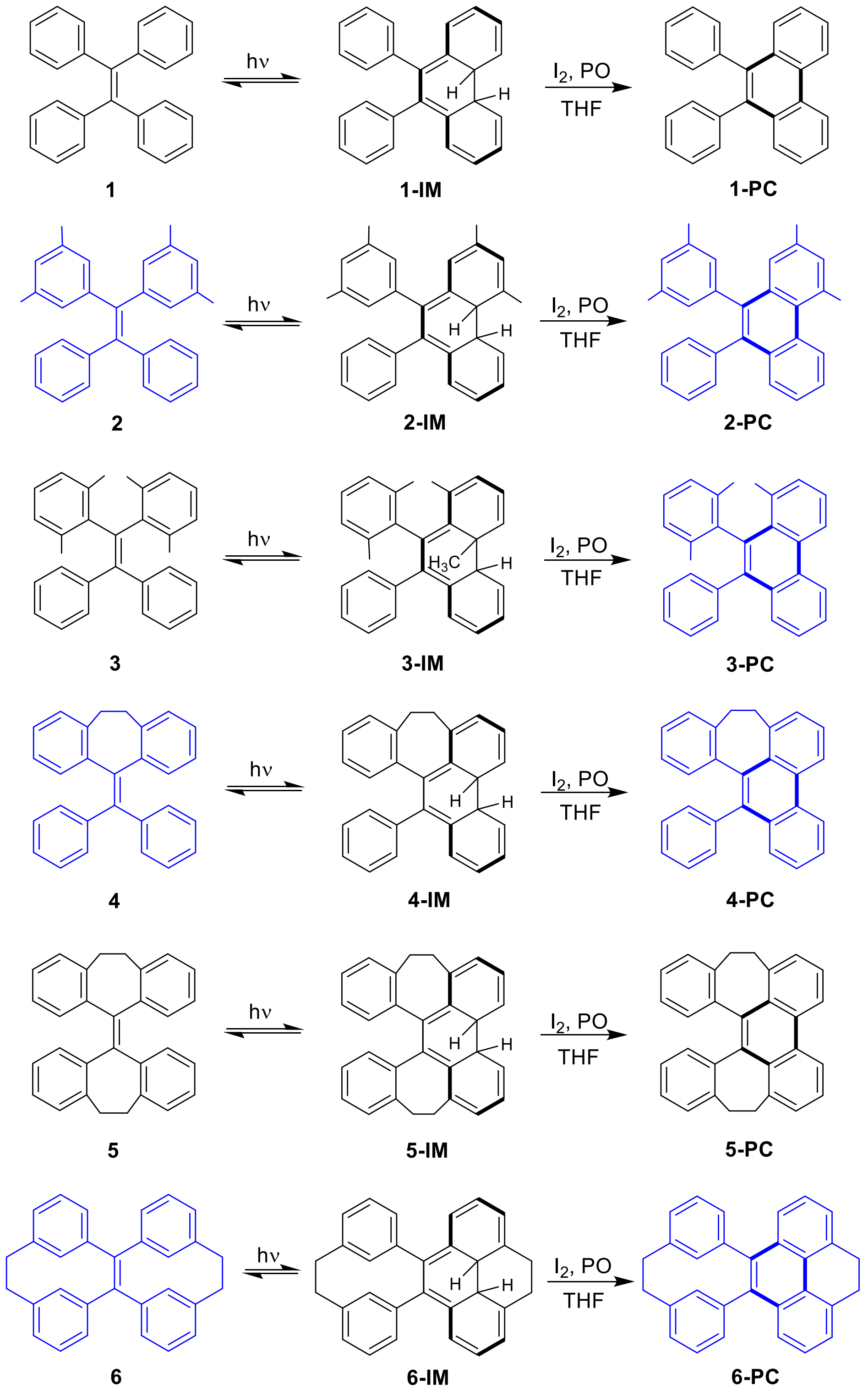

Figure S1. TPE derivatives **1-6** and their transformations upon photoexcitation.

2. Synthetic protocols of TPE derivatives

The compounds **1**, **3**, **5**, **1-PC** and **5-PC** are literature known compounds and were synthesized according to procedures published elsewhere.[1-3] The compound **1-PC** published in literatures is carried out by chemically-induced cyclization reaction using DDQ (2,3-dichloro-5,6-dicyano-p-benzoquinone) as an oxidizing agent, in which the intermediary cyclized TPE (**1-IM**) is formed by using methanesulfonic acid as a catalyst.[1-2]

All the chemical reagents were purchased from Sigma Aldrich and used as received without further purification. The ^{1}H and ^{13}C NMR spectra were measured at room temperature on a Bruker ARX 400 NMR spectrometer using $CDCl_3$ as solvent and tetramethylsilane (TMS: δ=0 ppm) as internal standard. High-resolution mass spectra (HRMS) were recorded on a Finnigan MAT TSQ 7000 Mass Spectrometer System.

2.1 Synthesis of 2

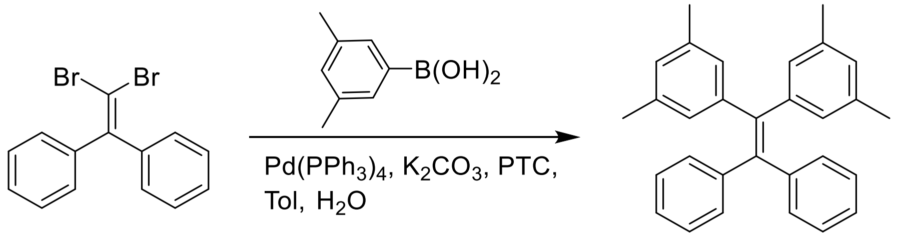

2a **2**

The compound **2a** was synthesized according to a literature procedure and purified by column chromatography.[3] A mixture of **2a** (1.370 g, 4.053 mmol), 3,5-dimethylphenylboronic acid (0.757 g, 5.047 mmol), $Pd(PPh_3)_4$ (0.235 g, 0.203 mmol), tetrabutylammonium hydrogen sulfate (phase transfer catalyst, PTC, 0.139 g, 0.409 mmol) and K_2CO_3 (1.693 g, 12.250 mmol) in toluene (80 mL) and water (40 mL) were added in a 250 mL two-neck flask. The reaction mixture was heated up to 90°C and stirred under N_2 overnight. On the next day, the two-layered reaction mixture was cooled down to r.t., the organic layer was separated and the water layer was extracted with dichloromethane (3×50 mL). The concentrated residue was purified *via* silica gel flash chromatography (dichloromethane/hexane = 1:30). Colorless block single crystals of **2** were grown from hexane by slow evaporation. 0.967 g of **2** as white solid was obtained. Yield: 55%. The ^{1}H NMR spectrum of **2** is shown in Figure S2. ^{1}H NMR (400.132 MHz, r.t., $CDCl_3$), δ_H (TMS, ppm): 7.16-7.04 (m, 6H, Ar-*H*), 7.04-6.94 (m, 4H, Ar-*H*), 6.76-6.68 (m, 2H, Ar-*H*), 6.67-6.59 (m, 4H, Ar-*H*), 2.11 (s, 12H, C*H*$_2$). The $^{13}C\{^{1}H\}$ NMR spectrum of **2** is shown in Figure S3. ^{13}C NMR (100.632 MHz, r.t., $CDCl_3$), δ_C (TMS, ppm): 144.21, 143.57, 141.52, 140.49, 136.84, 131.33, 129.27, 128.11, 127.59, 126.23, 21.31. MALDI-TOF-HRMS: m/z calcd. for $[C_{30}H_{28}]$: 388.2191; found 388.2200.

2.2 Synthesis of 4

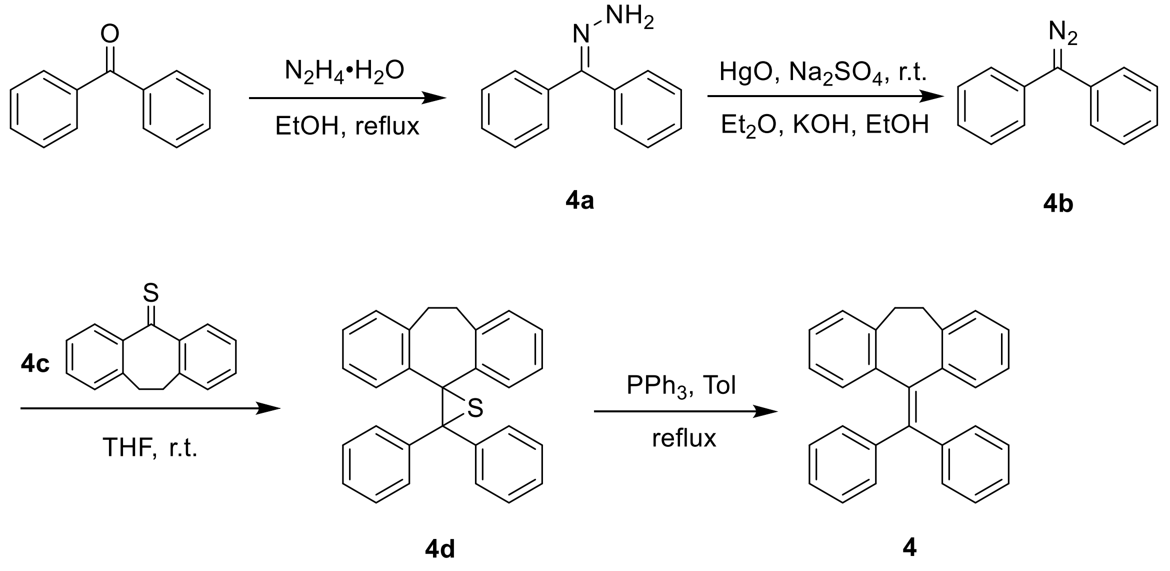

The compound **4a** was synthesized according to a literature procedure and purified by crystallization. [4] The compound **4c** was synthesized according to a literature procedure and purified by column chromatography. [5] In a 500 mL one-neck round-bottom flask compound **4a** (5.600 g, 28.535 mmol) was dissolved in dry Et_2O (250 mL) and anhydrous Na_2SO_4 (3.780 g, 26.612 mmol), HgO (12.360 g, 57.066 mmol) were added. A freshly prepared saturated solution of KOH in EtOH (18 mL) was then added, whereupon the reaction mixture was stirred at r.t. overnight. The resulting suspension was then filtered and the solid residue was washed several times with Et_2O, filtrates were combined and concentrated under reduced pressure yielding crude **4b** (as identified by its NMR spectra reported elsewhere)[6] (5.500 g, 28.315 mmol) as solid which was used in the next step without further purification. In a 500 mL two-neck round-bottom flask under nitrogen compound **4c** (4.700 g, 20.952 mmol) was dissolved in dry THF (150 mL), then the solution of compound **4b** (5.500 g, 28.315 mmol) in dry THF (200 mL) was added. The reaction mixture was stirred at r.t. overnight. The resulting reaction mixture was concentrated under reduced pressure until a solid precipitated. The solid was washed with EtOH to obtain crude product of **4d** (as identified by its NMR spectra reported elsewhere) [7] (7.811 g, 20.000 mmol) as a white solid. A mixture of compound **4d** (7.811 g, 20.000 mmol) and triphenylphosphine PPh_3 (9.000 g, 34.313 mmol) in anhydrous toluene (250 mL) was heated to reflux overnight. On the next day, the resulting reaction mixture was concentrated under reduced pressure until a solid precipitated. The solid was washed with EtOH to remove the unreacted PPh_3 to obtain crude product of **4.** The compound **4** was purified *via* silica gel flash chromatography (dichloromethane/hexane = 1:4). Colorless needle-like single crystals of **4** were grown from hexane by slow evaporation. 5.807 g of **4** as a white solid were obtained. Yield: 81%. The ^1H NMR spectrum of **4** is shown in Figure S4. ^1H NMR (400.132 MHz, r.t., $CDCl_3$), δ_H (TMS, ppm): 7.25-7.21 (m, 4H, Ar-*H*), 7.19-7.10 (m, 4H, Ar-*H*), 7.09-7.03 (m, 4H, Ar-*H*), 7.03-6.96 (m, 4H, Ar-*H*), 6.91-6.80 (m, 2H, Ar-*H*), 3.75-3.55 (m, 2H, C*H*$_2$), 3.05-2.84 (m, 2H, C*H*$_2$). The $^{13}C\{^1H\}$ NMR spectrum of **4** is shown in Figure S5. ^{13}C NMR (100.632 MHz, r.t., $CDCl_3$), δ_C (TMS, ppm): 142.07, 141.46, 141.16, 140.38, 137.83, 129.74, 129.71, 128.93, 127.83, 126.91, 126.37, 125.46, 32.76. MALDI-TOF-HRMS: m/z calcd. for $[C_{28}H_{22}]$: 358.1722; found 358.1702.

2.3 Synthesis of 6

The synthesis of side-locked TPE **6** is rather challenging. Up till now, side-locked TPE **6** has not been successfully synthesized and isolated by others, [8] although other kinds of locked structures have been studied. [9-13]

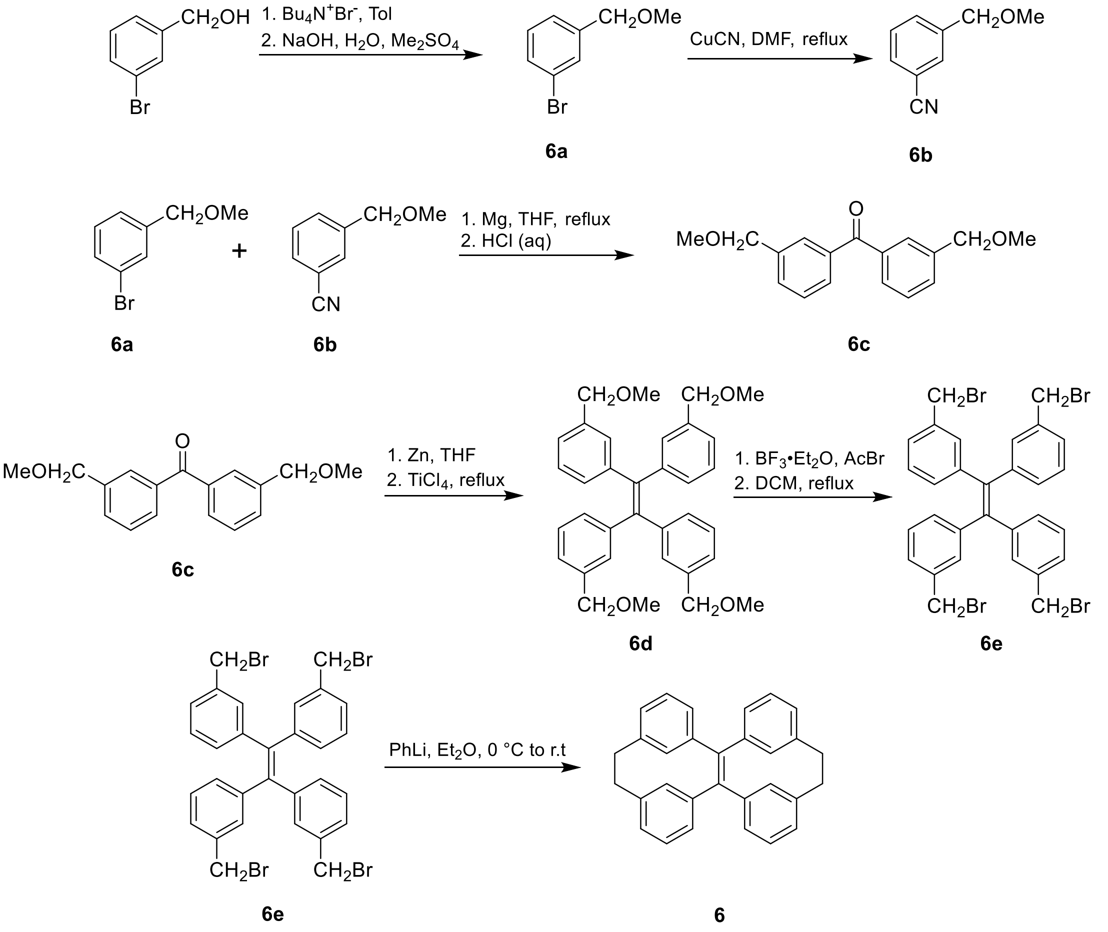

2.3.1 Synthesis of 6a: An aqueous solution of NaOH (20.000g, 50 wt%) was added at r.t. to a 250 mL round-bottom flask containing 3-bromobenzyl alcohol (2.000 g, 10.693 mmol), tetrabutylammonium bromide, $^{n}Bu_4NBr$, (0.100 g, 0.310 mmol) and toluene (50 mL). After stirring the solution for 30 min, the reaction mixture was cooled down to 0°C and kept at this temperature using an ice bath, while dimethyl sulfate, Me_2SO_4, (8 mL, 84.357 mmol) was added dropwise. Upon completion of addition the ice bath was removed and the reaction solution was warmed up to and kept at 40°C using oil bath for 3-4 hours. After that, the reaction mixture was cooled down to r.t. and ammonium hydroxide NH_4OH (40 mL, 28 wt%) was added very slowly to quench the excess of Me_2SO_4, whereupon the reaction mixture was allowed to stir for an additional hour. The upper layer of toluene solution was separated using a separatory funnel. The remaining layer was washed with H_2O (30 mL) and extracted with toluene (3×30 mL). Toluene layers were combined and the solvent was removed. Compound **1** was purified *via* silica gel flash chromatography (ethyl acetate/hexane = 1:9). 0.887 g of **1** as a pale yellow oil was obtained. Yield: 41%. The ^{1}H NMR spectrum of **6a** is shown in Figure S6. ^{1}H NMR (400.132 MHz, r.t., $CDCl_3$), δ_H (TMS, ppm): 7.53-7.48 (m, 1H, Ar-*H*), 7.45-7.37 (m, 1H, Ar-*H*), 7.25-7.17 (m, 2H, Ar-*H*), 4.41 (s, 2H, Ph-CH_2), 3.39 (s, 3H, Me-*H*). The $^{13}C\{^{1}H\}$ NMR spectrum of **6a** is shown in Figure S7. $^{13}C\{^{1}H\}$ NMR (100.632 MHz, r.t.,

$CDCl_3$), δ_C (TMS, ppm): 140.69, 130.66, 130.55, 129.98, 126.05, 122.56, 73.79, 58.29. CI-HRMS (NH_3 negative ion mode): *m/z* calcd. for $[C_8H_9BrO]^-$: 198.9837; found 198.9450.

2.3.2 Synthesis of 6b: Copper cyanide CuCN (0.593 g, 6.621 mmol) was added at r.t. to a 100 mL round-bottom flask containing **6a** (0.887 g, 4.412 mmol) and dimethylformamide, DMF, (10 mL). The color of the suspension solution turned to green after stirring for a few seconds. Then the reaction mixture was refluxed overnight. On the next day, the reaction mixture, while still hot, was dumped into cold water (0°C) and large amount of drab suspension formed immediately. The suspension was filtered and a dull light-brownish solid was obtained. The isolated precipitate was added to a chilled (0°C) mixture of H_2O (20 mL) and ethylenediamine (8 mL), and the suspension was allowed to warm up to r.t. and stirred for 20 min. Dichloromethane, DCM, (60 mL) was added and precipitate dissolved. The solution was stirred for further 30 min. It was then washed with H_2O (20 mL) and extracted with dichloromethane (3×20 mL). DCM layers were combined and the solvent was removed. Compound **6b** was purified *via* silica gel flash chromatography (ethyl acetate/hexane = 1:9). 0.539 g of **6b** as a yellow oil was obtained. Yield: 83%. The ^{1}H NMR spectrum of **6b** is shown in Figure S8. ^{1}H NMR (400.132 MHz, r.t, $CDCl_3$), δ_H (TMS, ppm): 7.63 (m, 1H, Ar-*H*), 7.59-7.52 (m, 2H, Ar-*H*), 7.49-7.40 (m, 1H, Ar-*H*), 4.47 (s, 2H, Ph-C*H*$_2$), 3.41 (s, 3H, Me-*H*). The ^{13}C{^{1}H} NMR spectrum of **6b** is shown in Figure S9. ^{13}C NMR (100.632 MHz, r.t., $CDCl_3$), δ_C (TMS, ppm): 139.99, 131.77, 131.33, 130.95, 129.28, 118.86, 112.59, 73.49, 58.60. CI-HRMS (CH_4 positive ion mode): m/z calcd. for $[C_9H_9NO]^+$: 148.0684; found 148.0767.

2.3.3 Synthesis of 6c: In a 50 mL two-neck round-bottom flask under nitrogen compound **6a** (0.202 g, 1.005 mmol) was dissolved in dry THF (2 mL) and magnesium turnings (0.025 g, 1.028 mmol) were added. The solution was refluxed under nitrogen for 2 hours until all magnesium turnings disappeared and the reaction mixture turned yellowish brown. The reaction mixture was then cooled down to 0°C using an ice bath. A solution of compound **6b** (0.144 g, 0.978 mmol) in dry THF (2 mL) was added, and the resulting reaction mixture was refluxed overnight. On the next day, the reaction mixture was cooled down to 0°C and hydrochloric acid (37wt%, 1 mL, 12.076 mmol) and H_2O (1 mL) were added. The resulting solution was stirred at r.t. for 10 min and then refluxed overnight. The solvent and water were removed *in vacuo*, and compound **6c** was purified *via* silica gel flash chromatography (ethyl acetate/hexane = 1:9). 0.164 g of **6c** as a colorless oil was obtained. Yield: 62%. The ^{1}H NMR spectrum of **6c** is shown in Figure S10. ^{1}H NMR (400.132 MHz, r.t, $CDCl_3$), δ_H (TMS, ppm): 7.77 (m, 1H, Ar-*H*), 7.74-7.67 (m, 1H, Ar-*H*), 7.61-7.55(m, 1H, Ar-*H*), 7.51-7.43(m, 1H, Ar-*H*), 4.52 (s, 2H, Ph-C*H*$_2$), 3.41 (s, 3H, Me-*H*). The ^{13}C{^{1}H} NMR spectrum of **6c** is shown in Figure S11. ^{13}C NMR (100.632 MHz, r.t., $CDCl_3$), δ_C (TMS, ppm): 196.72, 138.79, 137.88, 131.76, 129.54, 129.20, 128.53, 74.30, 58.46. MALDI-TOF-HRMS: m/z calcd. for $[C_{17}H_{18}O_3]$: 270.1256; found 270.1275.

2.3.4 Synthesis of 6d: In a 100 mL two-neck round-bottom flask under nitrogen compound **6c** (1.613 g, 5.967 mmol) was dissolved in dry THF (35 mL) and zinc powder (0.936 g, 14.314 mmol) was added. The solution was kept at -78°C using a dry ice/acetone bath. Titanium tetrachloride, $TiCl_4$, (0.9 mL, 8.190 mmol) was added dropwise. Upon completion of addition the dry ice/acetone bath was removed and the reaction solution was refluxed overnight. On the next day, the reaction mixture was cooled down to r.t. and the crude product was taken up in ethyl acetate and filtered through a short plug of silica gel. All volatiles were then removed, and compound **6d** was purified *via* silica gel flash chromatography (ethyl acetate/hexane = 1:6). 1.193 g of **6d** as a colorless oil was obtained. Yield: 79%. The ^{1}H NMR spectrum of **6d** is shown in Figure S12. ^{1}H NMR (400.132 MHz, r.t, $CDCl_3$), δ_H (TMS, ppm): 7.12-7.03 (m, 8H, Ar-*H*), 7.01-6.93 (m, 8H, Ar-*H*), 4.24 (s, 8H, Ph-C*H*$_2$), 3.15 (s, 12H, Me-

H). The $^{13}C\{^1H\}$ NMR spectrum of **6d** is shown in Figure S13. ^{13}C NMR (100.632 MHz, r.t., $CDCl_3$), δ_C (TMS, ppm): 143.70, 141.16, 137.60, 130.94, 130.80, 127.95, 126.15, 74.55, 57.71. MALDI-TOF-HRMS: m/z calcd. for [$C_{34}H_{36}O_4$]: 508.2614; found 508.2597.

2.3.5 Synthesis of 6e: In a 100 mL two-neck round-bottom flask under nitrogen compound **6d** (1.193 g, 2.345 mmol) was dissolved in dry dichloromethane (70 mL). The solution was kept at 0°C using an ice bath. Boron trifluoride diethyl etherate BF_3•Et_2O (7.2 mL, 58.339 mmol) and acetyl bromide, AcBr, (4.8 mL, 64.924 mmol) were added. The reaction mixture was stirred for 10 min, after which the ice bath was removed and the reaction mixture was refluxed overnight. On the next day, the reaction mixture was cooled down to 0°C and the aqueous $NaHCO_3$ solution (36 mL, 10 wt%) was slowly added. The reaction mixture was stirred at 0°C for 10 min and then warmed up to r.t. The crude product was extracted with dichloromethane (3×20 mL). DCM layers were combined and the solvent was removed. Compound **6e** was purified *via* silica gel flash chromatography (ethyl acetate/hexane = 1:20). 0.864 g of **6e** as a yellow solid was obtained. Yield: 57%. The ^1H NMR spectrum of **6e** is shown in Figure S14. ^1H NMR (400.132 MHz, r.t, $CDCl_3$), δ_H (TMS, ppm): 7.20-7.08 (m, 8H, Ar-*H*), 7.08-7.02 (m, 4H, Ar-*H*), 7.00-6.89 (m, 4H, Ar-*H*), 4.28 (s, 8H, Ph-CH_2). The $^{13}C\{^1H\}$ NMR spectrum of **6e** is shown in Figure S15. ^{13}C NMR (100.632 MHz, r.t., $CDCl_3$), δ_C (TMS, ppm): 143.23, 140.72, 137.54, 132.09, 131.19, 128.49, 127.61, 33.45. MALDI-TOF-HRMS: m/z calcd. for [$C_{30}H_{24}Br_4$]: 703.8571; found 703.8575.

2.3.6 Synthesis of 6: In a 500 mL two-neck round-bottom flask under nitrogen phenyl lithium PhLi (1.5 mL, 1M in Et_2O, 1.500 mmol) was added to dry Et_2O (30 mL). The solution was kept at 0°C using an ice bath and a dilute solution of compound **6e** (0.255 g, 0.362 mmol) in dry Et_2O (350 mL) was added dropwise (1-2 drops per second) under nitrogen. Upon completion of addition the reaction mixture was kept at 0°C for additional 3 hours and then warmed up to r.t. overnight. On the next day, the solvent was removed and the residue was taken up in a water/Et_2O mixture. The layers were separated and the water layer was extracted with Et_2O (3×60 mL). The Et_2O layers were combined and the solvent was removed. A yellow solid of crude **6** was isolated. The above procedure was repeated 5 times to obtain enough raw material for purification. Obtained raw material was then purified *via* silica gel flash chromatography which was performed twice, first using dichloromethane/hexane = 1:6 mixture as eluent to get the crude product, and then using pure hexanes to purify the crude product. Yellow needle-like single crystals of **6** were grown from acetonitrile or THF by slow evaporation. 0.139 g of **6** as yellow solid was obtained. Yield: 20%. The ^1H NMR spectrum of **6** is shown in Figure S16. ^1H NMR (400.132 MHz, r.t, $CDCl_3$), δ_H (TMS, ppm): 7.27-7.21 (m, 4H, Ar-*H*), 7.19-7.11 (m, 4H, Ar-*H*), 7.10-7.04 (m, 4H, Ar-*H*), 5.73 (m, 4H, Ar-*H*), 3.28-3.11 (m, 4H, CH_2), 2.13-1.97 (m, 4H, CH_2). The $^{13}C\{^1H\}$ NMR spectrum of **6** is shown in Figure S17. ^{13}C NMR (100.632 MHz, r.t., $CDCl_3$), δ_C (TMS, ppm): 141.88, 140.24, 139.82, 136.65, 128.68, 128.09, 127.05, 43.27. MALDI-TOF-HRMS: m/z calcd. for [$C_{30}H_{24}$]: 384.1878; found 384.1889.

2.4 Photochemical reactions of 1-4 and 6

The photocyclization reactions were carried out using high-pressure mercury lamp [5, 14]. A standard photochemical reaction vessel (1L) with a double-walled quartz immersion well was used for photo-induced cyclization reaction. A circulating chiller was used to provide cooling water (~15°C) to the quartz immersion well. Nitrogen (N_2) was bubbled through a stirred solution of TPE derivatives in dry THF for an hour. Then iodine (I_2) and (±)-propylene oxide (PO, 30 mL, 428.719 mmol) were added to the solution and the solution was purged with N_2 for another 15 min before photo-irradiation. The N_2 flow was maintained all throughout irradiation

with a 500W high-pressure mercury vapor lamp (PLS-LAM500) placed in the immersion quartz well. The progress of the reaction was monitored by TLC and ^{1}H NMR spectroscopy. After completion of the reaction, the solvent was evaporated under reduced pressure and the photocyclized TPE derivatives were purified by silica gel flash chromatography.

2.4.1 Photochemical reaction of 1 (synthesis of 1-PC):

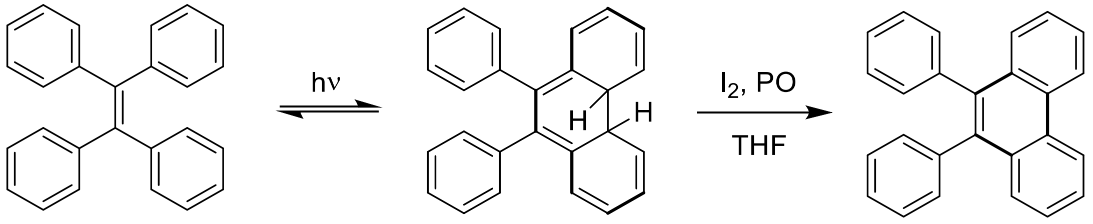

1 **1-IM** **1-PC**

1 (0.104 g, 0.313 mmol) and I_2 (0.088 g, 0.347 mmol) in dry THF (250 mL) were used in the photo-induced cyclization reaction. After 2 hours of irradiation, the resulting solution was concentrated under reduced pressure. The compound **1-PC** was purified *via* silica gel flash chromatography using hexane. 0.057 g of **1-PC** as a white solid were obtained. Yield: 55%. The spectroscopic data of the isolated pure **1-PC** (NMR and HRMS) are identical to previous literature reports for this compound. [1-2]

2.4.2 Photochemical reaction of 2 (synthesis of 2-PC):

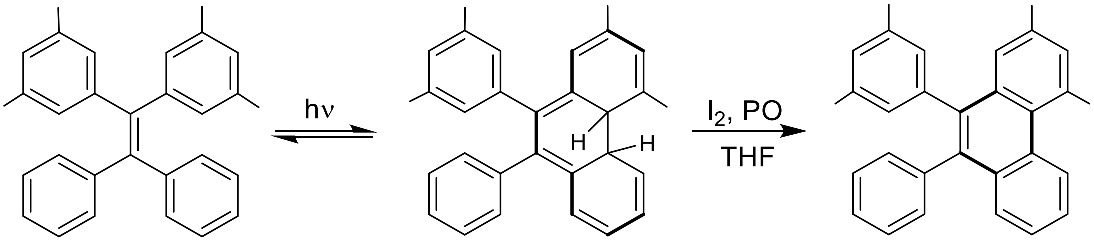

2 **2-IM** **2-PC**

2 (0.104 g, 0.268 mmol) and I_2 (0.080 g, 0.315 mmol) in dry THF (250 mL) were used in the photo-induced cyclization reaction. After an hour of irradiation, the resulting solution was concentrated under reduced pressure. The compound **2-PC** was purified *via* silica gel flash chromatography using hexane. Colorless plate-shaped single crystals of **2-PC** were grown from hexane by slow evaporation. 0.093 g of **2-PC** as a white solid were obtained. Yield: 90%. The ^{1}H NMR spectrum of **2-PC** is shown in Figure S18. ^{1}H NMR (400.132 MHz, r.t, $CDCl_3$), δ_H (TMS, ppm): 8.94-8.86 (m, 1H, Ar-*H*), 7.67-7.53 (m, 2H, Ar-*H*), 7.49-7.41 (m, 1H, Ar-*H*), 7.40-7.35 (m, 1H, Ar-*H*), 7.35-7.30 (m, 1H, Ar-*H*), 7.28-7.18 (m, 3H, Ar-*H*), 7.18-7.11 (m, 2H, Ar-*H*), 6.86-6.79 (m, 1H, Ar-*H*), 6.78-6.72 (m, 2H, Ar-*H*), 3.20 (s, 3H, Me-*H*), 2.41 (s, 3H, Me-*H*), 2.23 (s, 6H, Me-*H*). The $^{13}C\{^{1}H\}$ NMR spectrum of **2-PC** is shown in Figure S19. ^{13}C NMR (100.632 MHz, r.t., $CDCl_3$), δ_C (TMS, ppm): 140.21, 140.12, 137.92, 137.15, 136.76, 135.34, 134.91, 133.90, 133.07, 132.93, 131.24, 131.15, 129.07, 128.17, 127.90, 127.75, 127.51, 127.31, 126.37, 126.28,

125.49, 124.96, 27.47, 21.54, 21.39. MALDI-TOF-HRMS: m/z calcd. for [$C_{30}H_{26}$]: 386.2035; found 386.2040.

2.4.3 Photochemical reaction of 3 (synthesis of 3-PC):

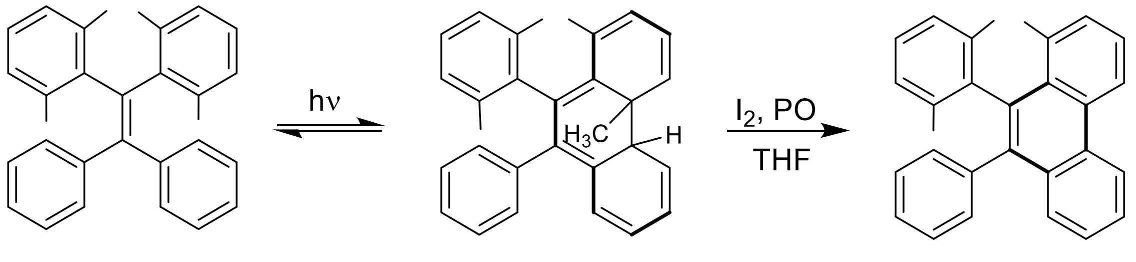

3 **3-IM** **3-PC**

3 (0.105 g, 0.270 mmol) and I_2 (0.076 g, 0.299 mmol) in dry THF (250 mL) were used in the photo-induced cyclization reaction. After an hour of irradiation, the resulting solution was concentrated under reduced pressure. The compound **3-PC** was purified *via* silica gel flash chromatography using hexane. Colorless needle-like single crystals of **3-PC** were grown from hexane by slow evaporation. 0.091 g of **3-PC** as a white solid were obtained. Yield: 90%. The ^{1}H NMR spectrum of **3-PC** is shown in Figure S20. ^{1}H NMR (400.132 MHz, r.t, $CDCl_3$), δ_H (TMS, ppm): 8.86-8.76 (m, 2H, Ar-*H*), 7.67-7.60 (m, 1H, Ar-*H*), 7.59-7.51 (m, 1H, Ar-*H*), 7.47-7.32 (m, 3H, Ar-*H*), 7.20-7.15 (m, 3H, Ar-*H*), 7.14-7.08 (m, 2H, Ar-*H*), 7.01-6.93 (m, 1H, Ar-*H*), 6.86-6.80 (m, 2H, Ar-*H*), 1.95 (s, 6H, Me-*H*), 1.90 (s, 3H, Me-*H*). The ^{13}C{^{1}H} NMR spectrum of **3-PC** is shown in Figure S21. ^{13}C NMR (100.632 MHz, r.t., $CDCl_3$), δ_C (TMS, ppm): 142.57, 139.69, 137.15, 136.66, 135.10, 132.03, 131.69, 130.83, 129.89, 129.49, 127.69, 127.22, 127.12, 126.78, 126.63, 126.45, 126.37, 126.20, 123.15, 121.99, 24.14, 21.19. MALDI-TOF-HRMS: m/z calcd. for [$C_{29}H_{24}$]: 372.1878; found 372.1882.

2.4.4 Photochemical reaction of 4 (synthesis of 4-PC):

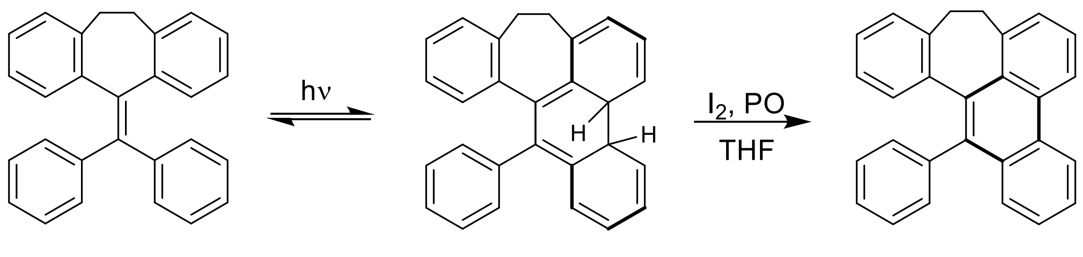

4 **4-IM** **4-PC**

4 (0.112 g, 0.312 mmol) and I_2 (0.088 g, 0.347 mmol) in dry THF (250 mL) were used in the photo-induced cyclization reaction. After 2 hours of irradiation, the resulting solution was concentrated under reduced pressure. The compound **4-PC** was purified *via* silica gel flash chromatography using hexane. Colorless needle-like single crystals of **4-PC** were grown from hexane by slow evaporation. 0.033 g of **4-PC** as a white solid were obtained. Yield: 30%. The ^{1}H NMR spectrum of **4-PC** is shown in Figure S22. ^{1}H NMR (400.132 MHz, r.t, $CDCl_3$), δ_H (TMS, ppm): 8.76-8.69 (m, 1H, Ar-*H*), 8.66-8.59 (m, 1H, Ar-*H*), 7.74-7.67 (m, 1H, Ar-*H*), 7.66-7.59 (m, 1H, Ar-*H*), 7.56-7.43 (m, 3H, Ar-*H*), 7.41-7.34 (m, 2H, Ar-*H*), 7.22-7.15 (m, 1H, Ar-*H*), 7.14-7.05 (m, 2H, Ar-*H*), 7.03-6.97 (m, 1H, Ar-*H*), 6.95-6.89 (m, 1H, Ar-*H*), 6.72-6.63 (m, 2H, Ar-*H*), 3.63-3.52 (m, 1H, CH_2), 3.46-3.36 (m, 2H, CH_2), 2.93-2.83 (m, 1H, CH_2). The ^{13}C{^{1}H} NMR

spectrum of **4-PC** is shown in Figure S23. ^{13}C NMR (100.632 MHz, r.t., $CDCl_3$), δ_C (TMS, ppm): 143.17, 140.97, 139.70, 139.05, 135.38, 135.20, 133.20, 132.09, 131.19, 130.92, 130.39, 129.74, 128.40, 127.95, 127.37, 126.98, 126.63, 126.61, 126.46, 125.59, 125.47, 125.14, 122.95, 120.47, 39.71, 33.15. MALDI-TOF-HRMS: m/z calcd. for [$C_{28}H_{20}$]: 356.1565; found 356.1563.

2.4.5 Photochemical reaction of 6 (synthesis of 6-PC):

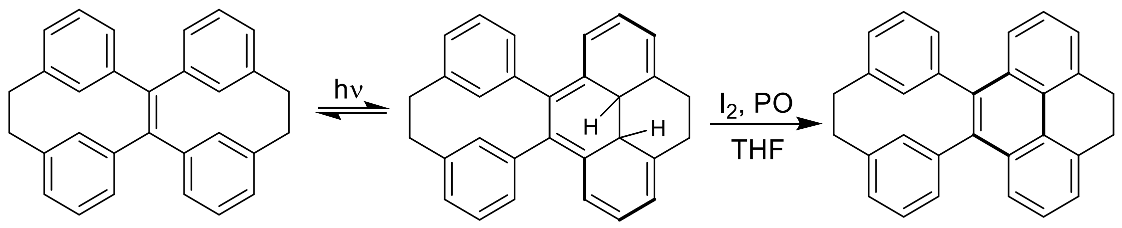

6 **6-IM** **6-PC**

6 (0.075 g, 0.195 mmol) and I_2 (0.060 g, 0.236 mmol) in dry THF (200 mL) were used in the photo-induced cyclization reaction. After an hour of irradiation, the resulting solution was concentrated under reduced pressure. The compound **6-PC** was purified *via* silica gel flash chromatography using hexane. Colorless block-shaped single crystals of **6-PC** were grown from acetonitrile or acetone by slow evaporation. 0.037 g of **6-PC** as a white solid were obtained. Yield: 50%. The ^{1}H NMR spectrum of **6-PC** is shown in Figure S24. ^{1}H NMR (400.132 MHz, r.t, $CDCl_3$), δ_H (TMS, ppm): 8.28-8.21 (m, 2H, Ar-*H*), 7.56-7.41 (m, 4H, Ar-*H*), 7.40-7.30 (m, 2H, Ar-*H*), 7.20-7.09 (m, 4H, Ar-*H*), 5.42 (m, 2H, Ar-*H*), 3.43-3.32 (m, 4H, CH_2), 3.31-3.23 (m, 2H, CH_2), 2.20-2.06 (m, 2H, CH_2). The ^{13}C{^{1}H} NMR spectrum of **6-PC** is shown in Figure S25. ^{13}C NMR (100.632 MHz, r.t., $CDCl_3$), δ_C (TMS, ppm): 140.67, 137.82, 137.37, 136.00, 135.30, 130.79, 130.02, 128.46, 128.33, 127.44, 126.45, 126.26, 125.23, 43.71, 29.21. MALDI-TOF-HRMS: m/z calcd. for [$C_{30}H_{22}$]: 382.1722; found 382.1703.

3 ^{1}H NMR and ^{13}C NMR spectra

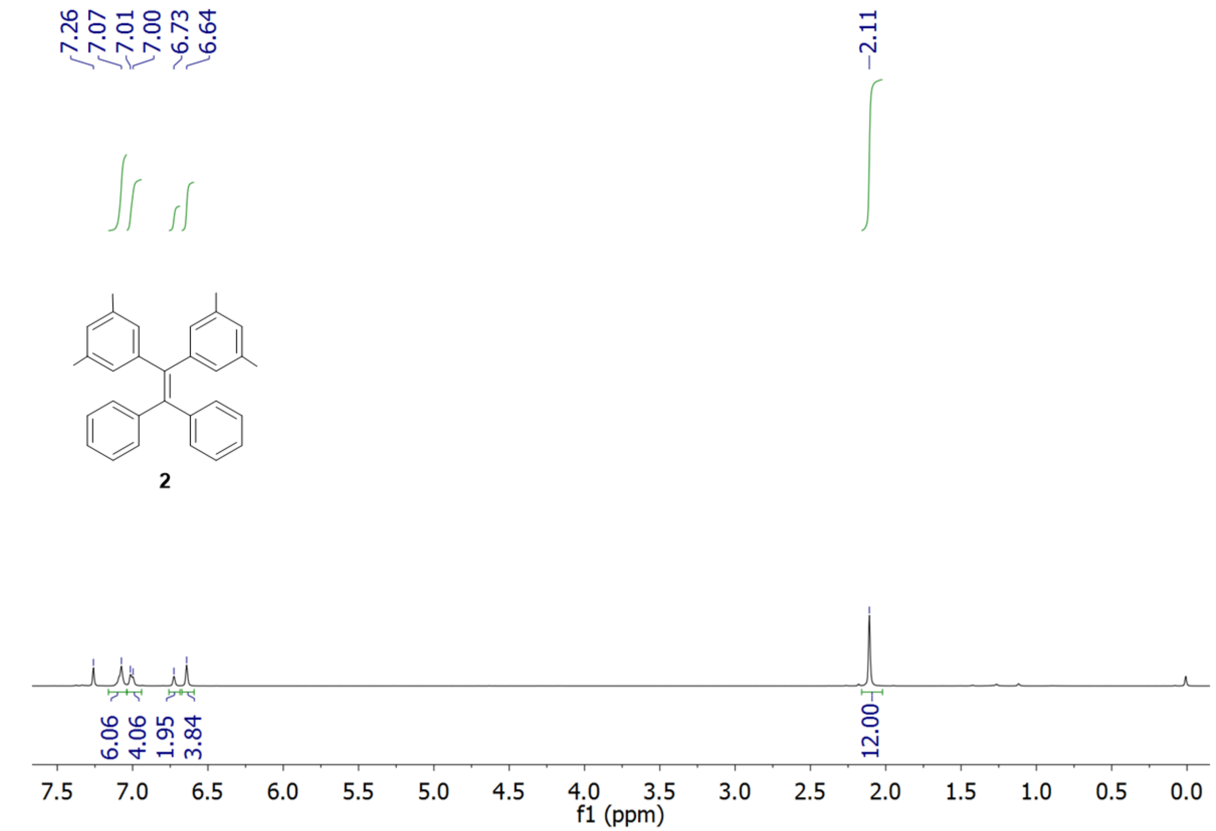

Figure S2. ^{1}H NMR spectrum of **2** (400.132 MHz, r.t., $CDCl_3$/TMS).

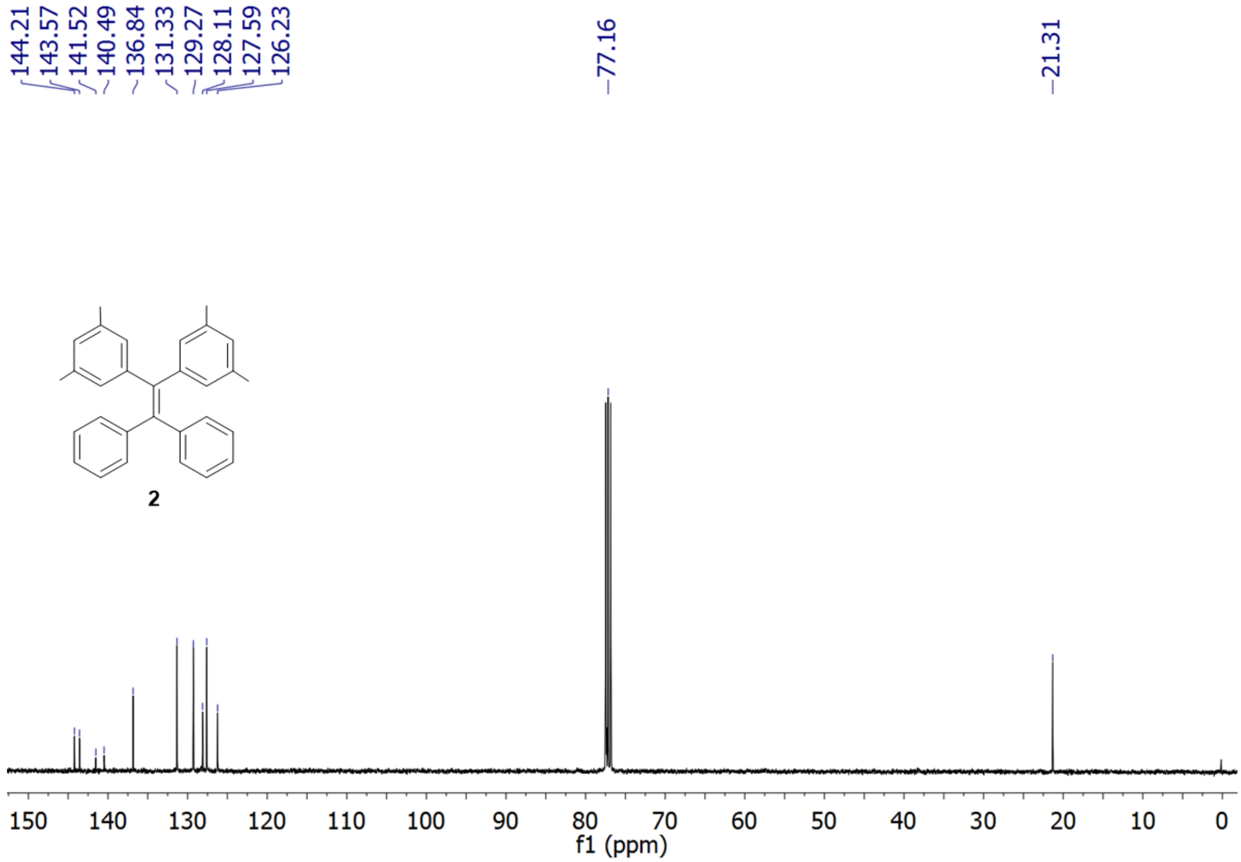

Figure S3. ^{13}C{^{1}H} NMR spectrum of **2** (100.632 MHz, r.t., $CDCl_3$/TMS).

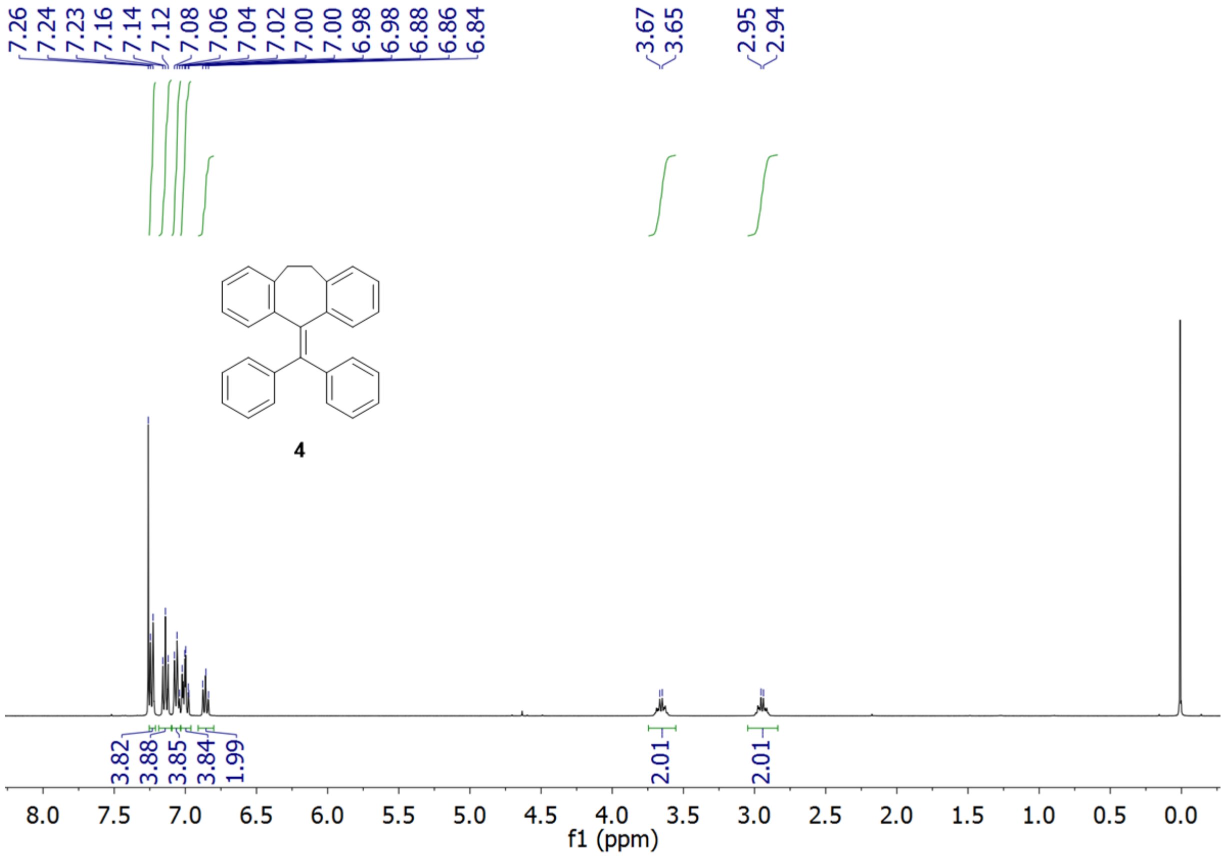

Figure S4. ^{1}H NMR spectrum of **4** (400.132 MHz, r.t., $CDCl_3$/TMS).

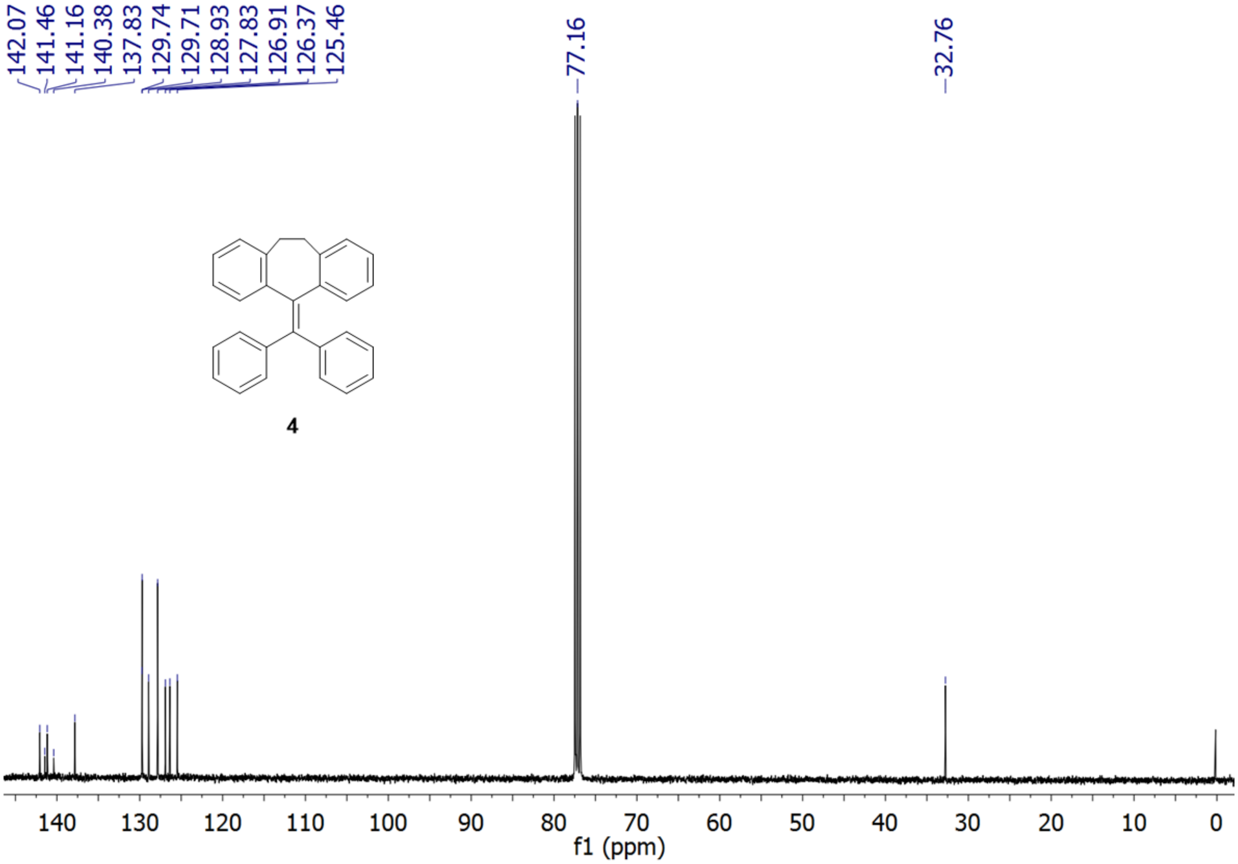

Figure S5. ^{13}C{^{1}H} NMR spectrum of **4** (100.632 MHz, r.t., $CDCl_3$/TMS).

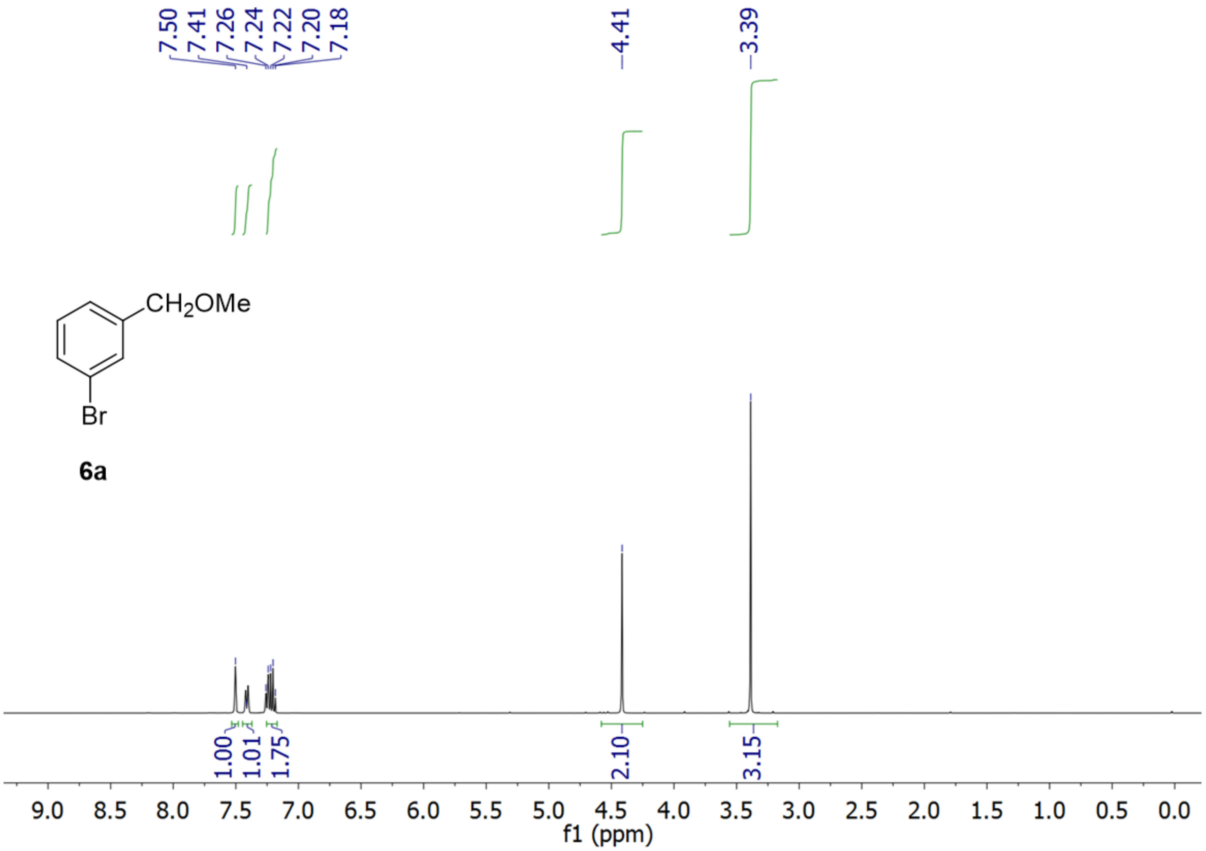

Figure S6. ^{1}H NMR spectrum of **6a** (400.132 MHz, r.t., $CDCl_3$/TMS).

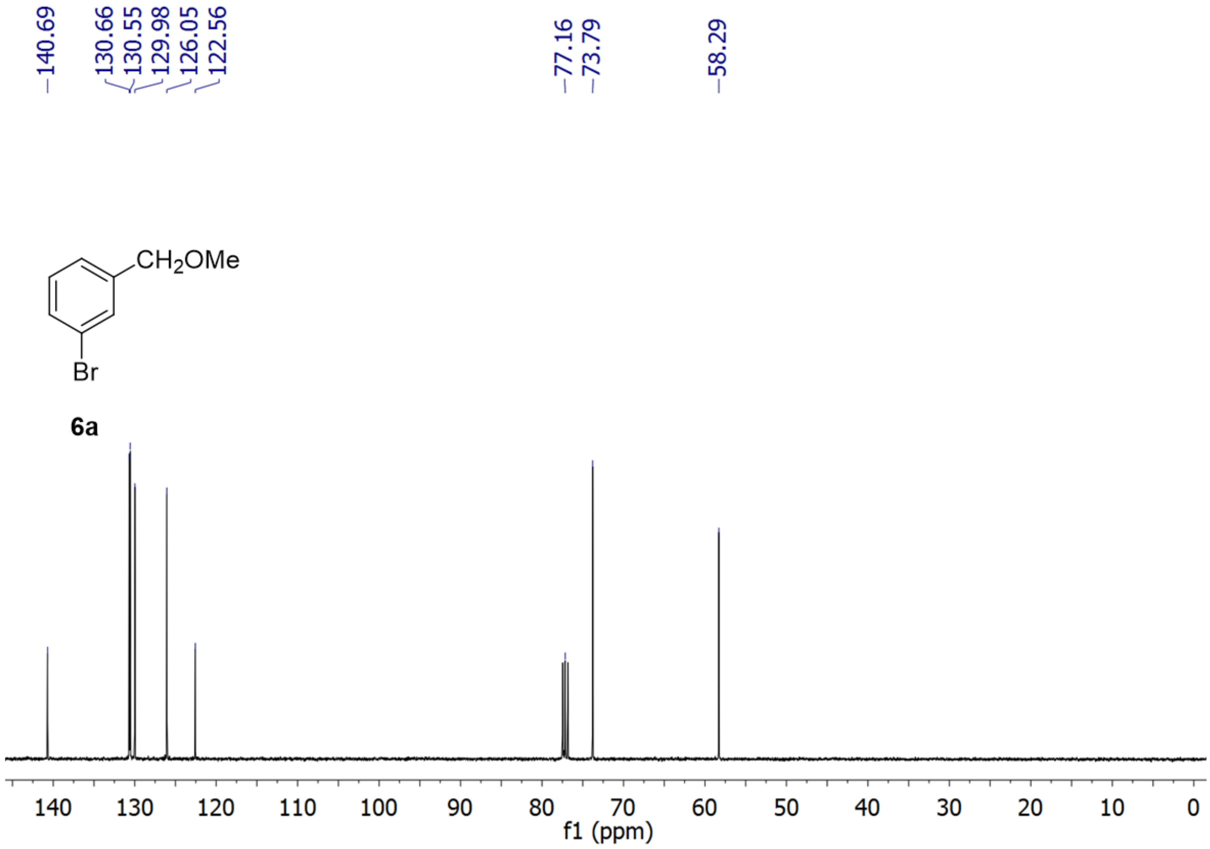

Figure S7. $^{13}C\{^1H\}$ NMR spectrum of **6a** (100.632 MHz, r.t., $CDCl_3$/TMS).

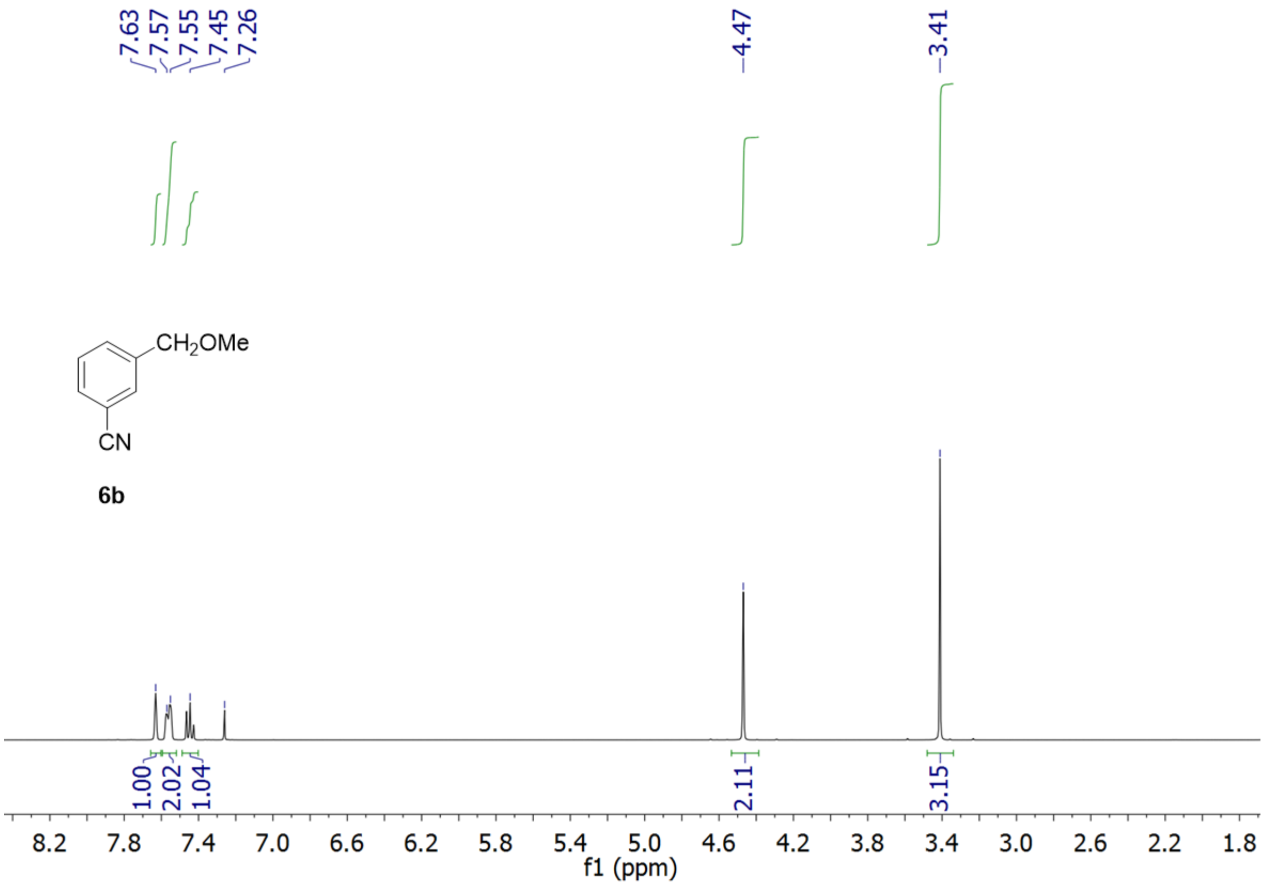

Figure S8. ^{1}H NMR spectrum of **6b** (400.132 MHz, r.t., $CDCl_3$/TMS).

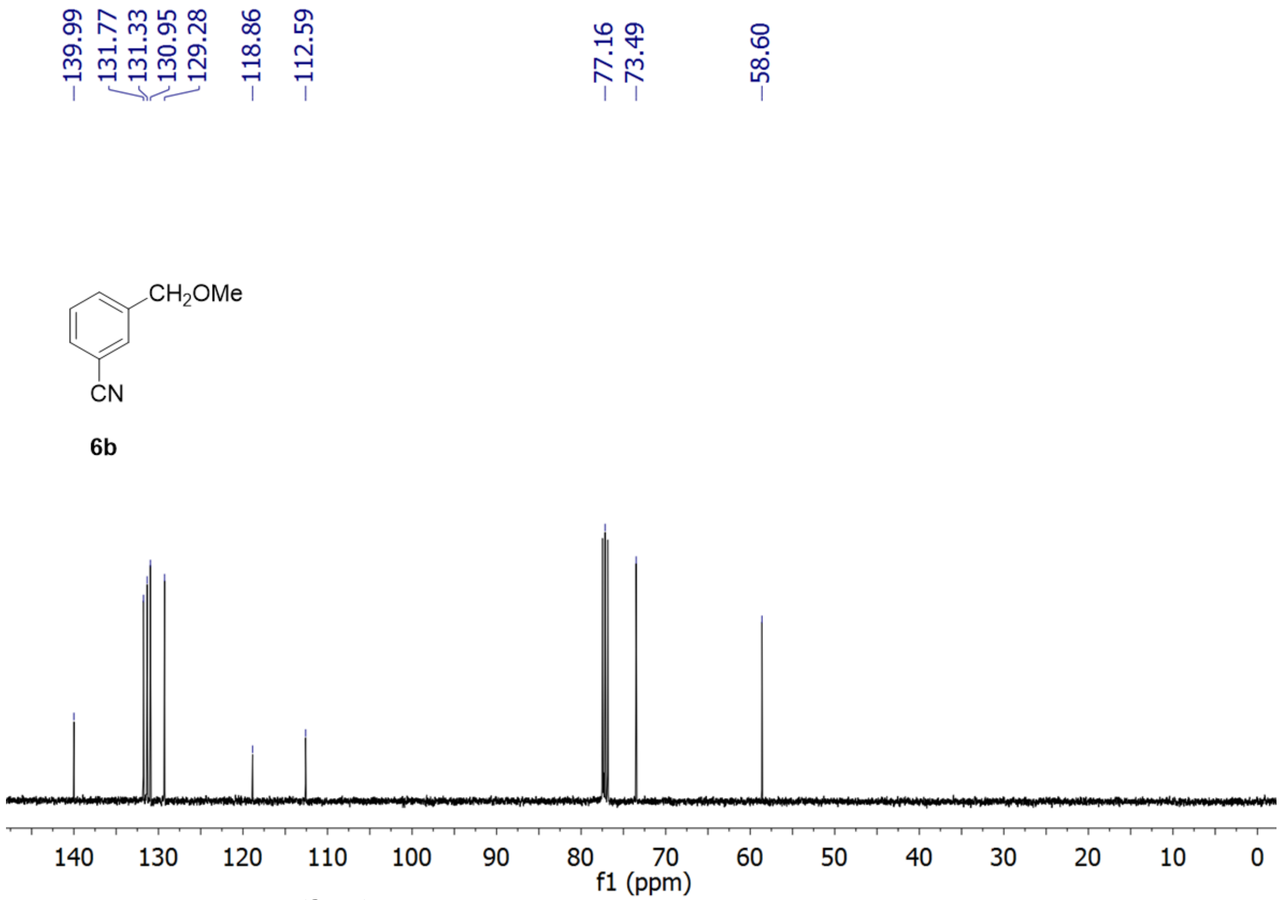

Figure S9. $^{13}C\{^{1}H\}$ NMR spectrum of **6b** (100.632 MHz, r.t., $CDCl_3$/TMS).

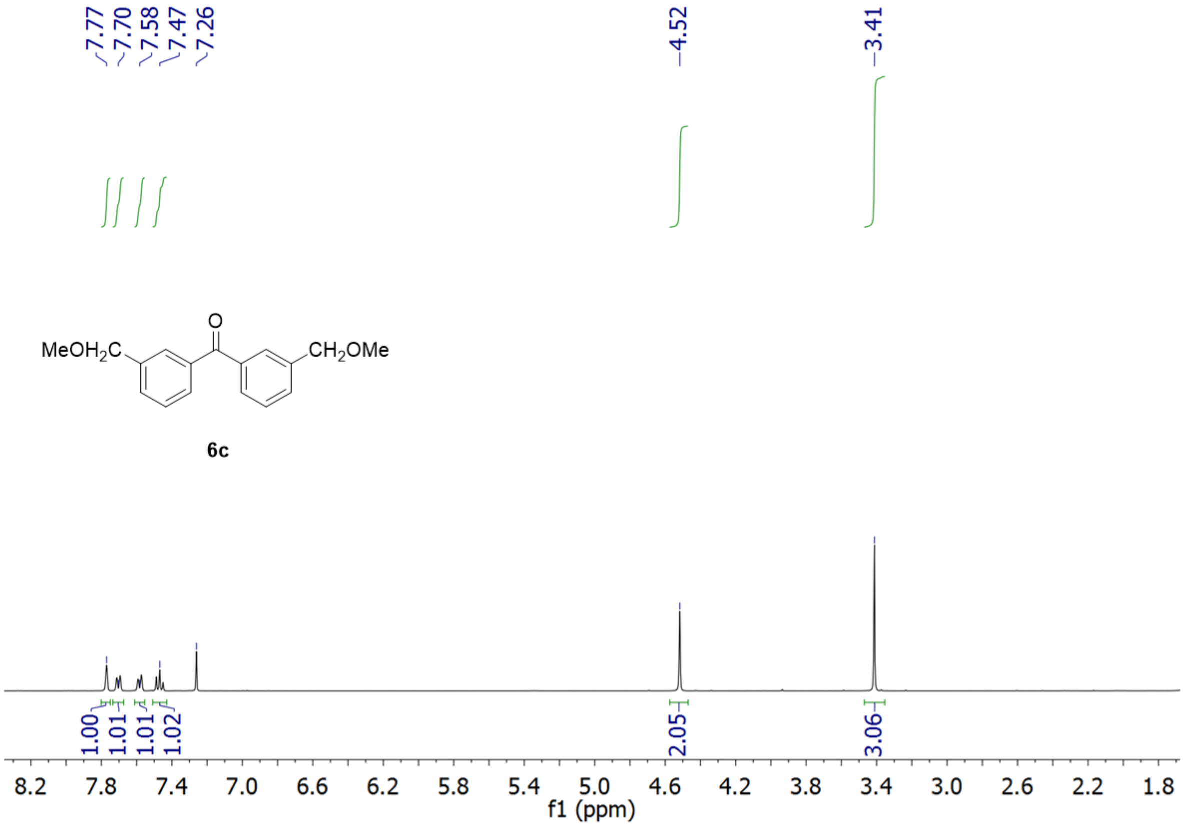

Figure S10. ^{1}H NMR spectrum of **6c** (400.132 MHz, r.t., $CDCl_3$/TMS).

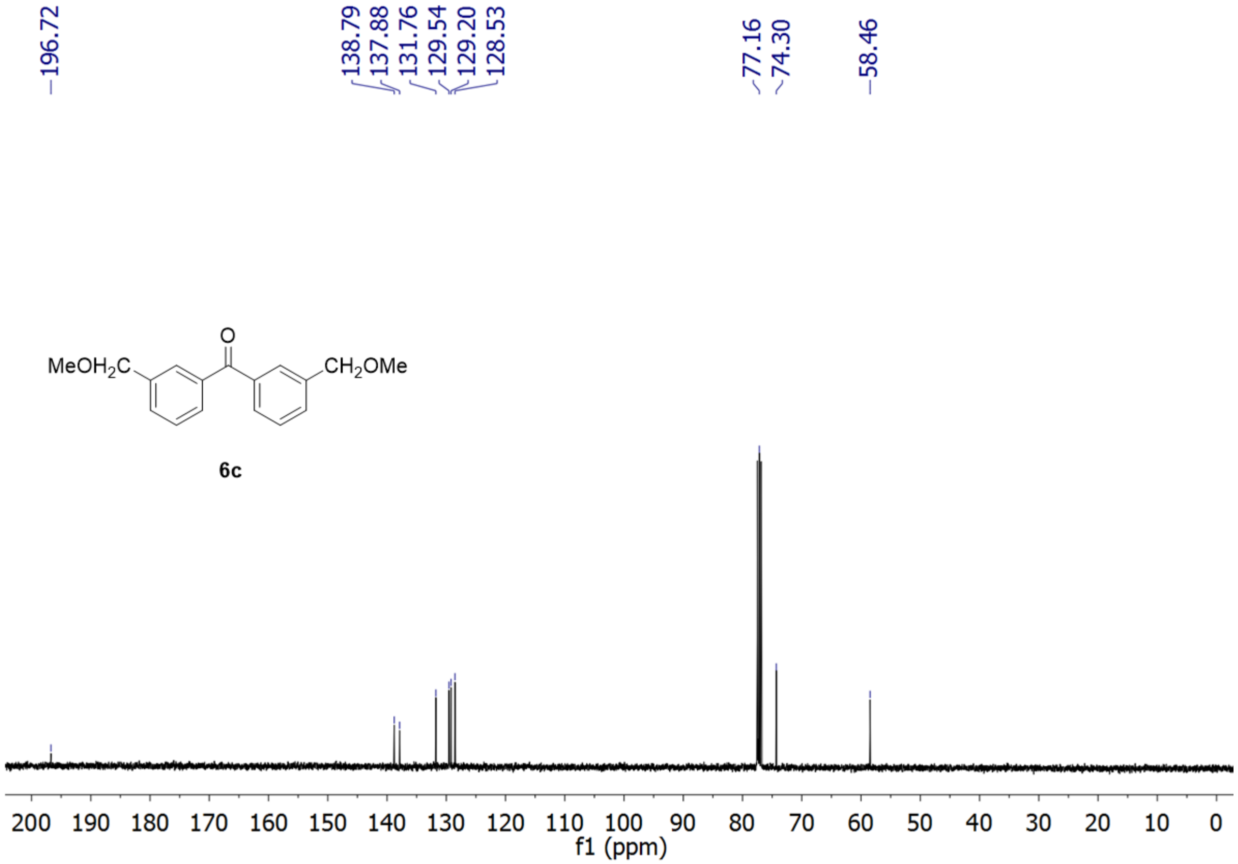

Figure S11. ^{13}C{^{1}H} NMR spectrum of **6c** (100.632 MHz, r.t., $CDCl_3$/TMS).

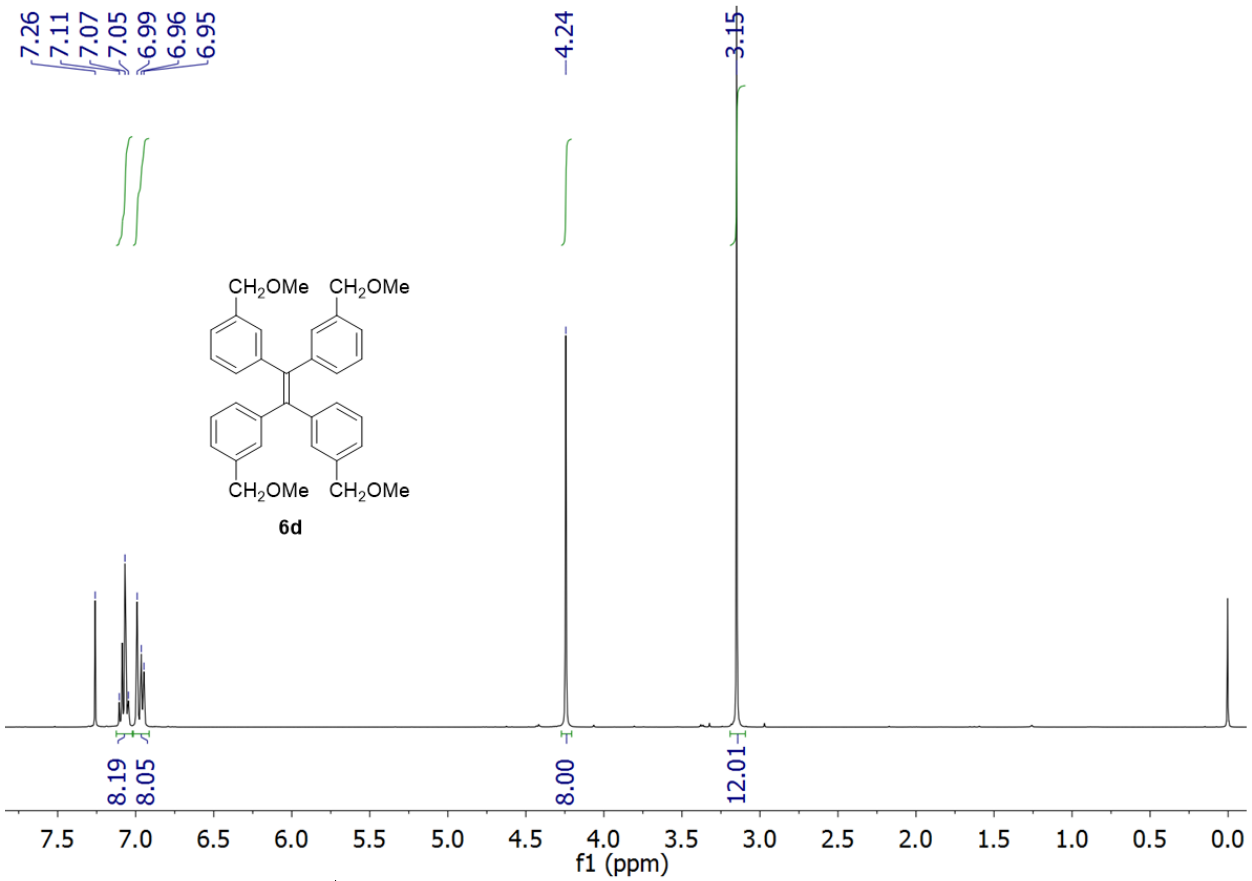

Figure S12. ^{1}H NMR spectrum of **6d** (400.132 MHz, r.t., $CDCl_3$/TMS).

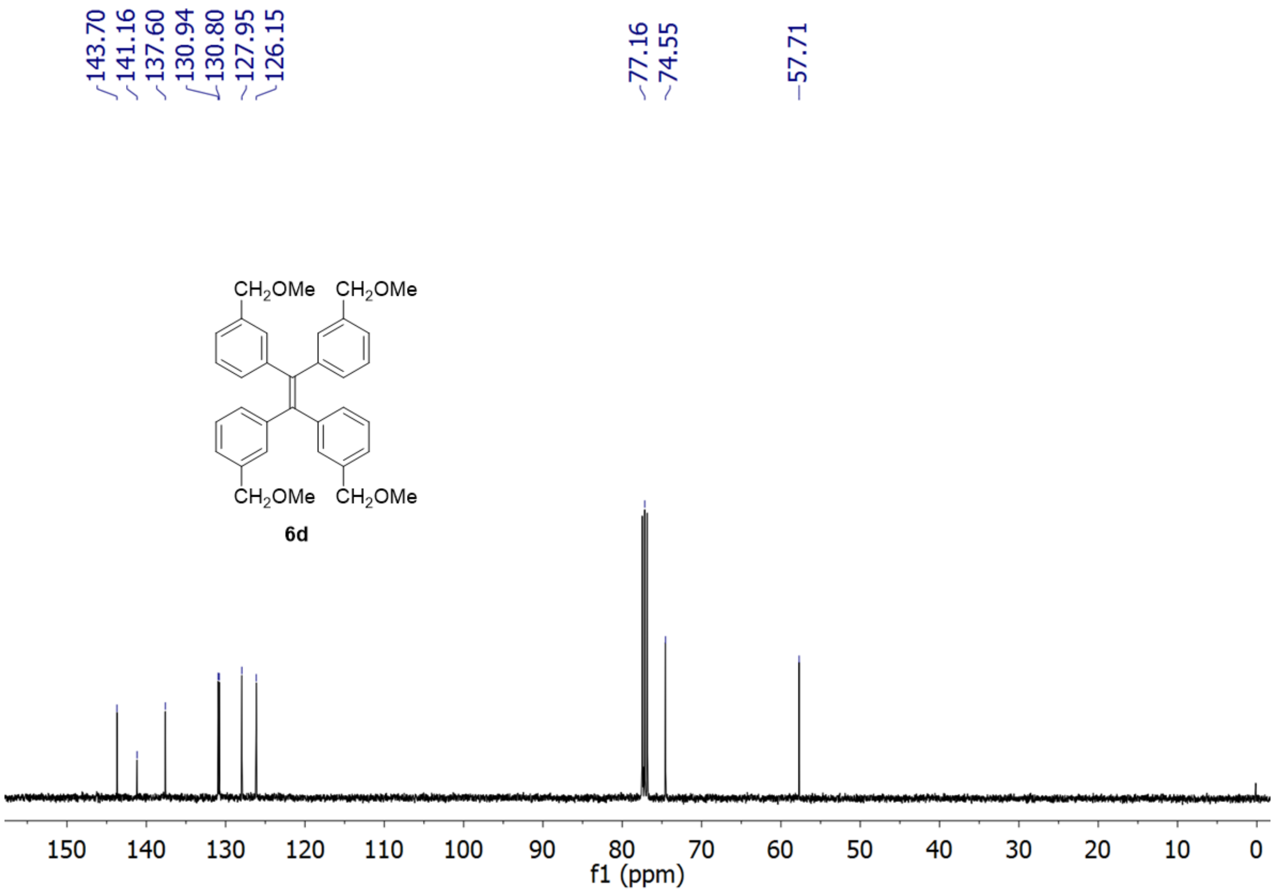

Figure S13. $^{13}C\{^{1}H\}$ NMR spectrum of **6d** (100.632 MHz, r.t., $CDCl_3$/TMS).

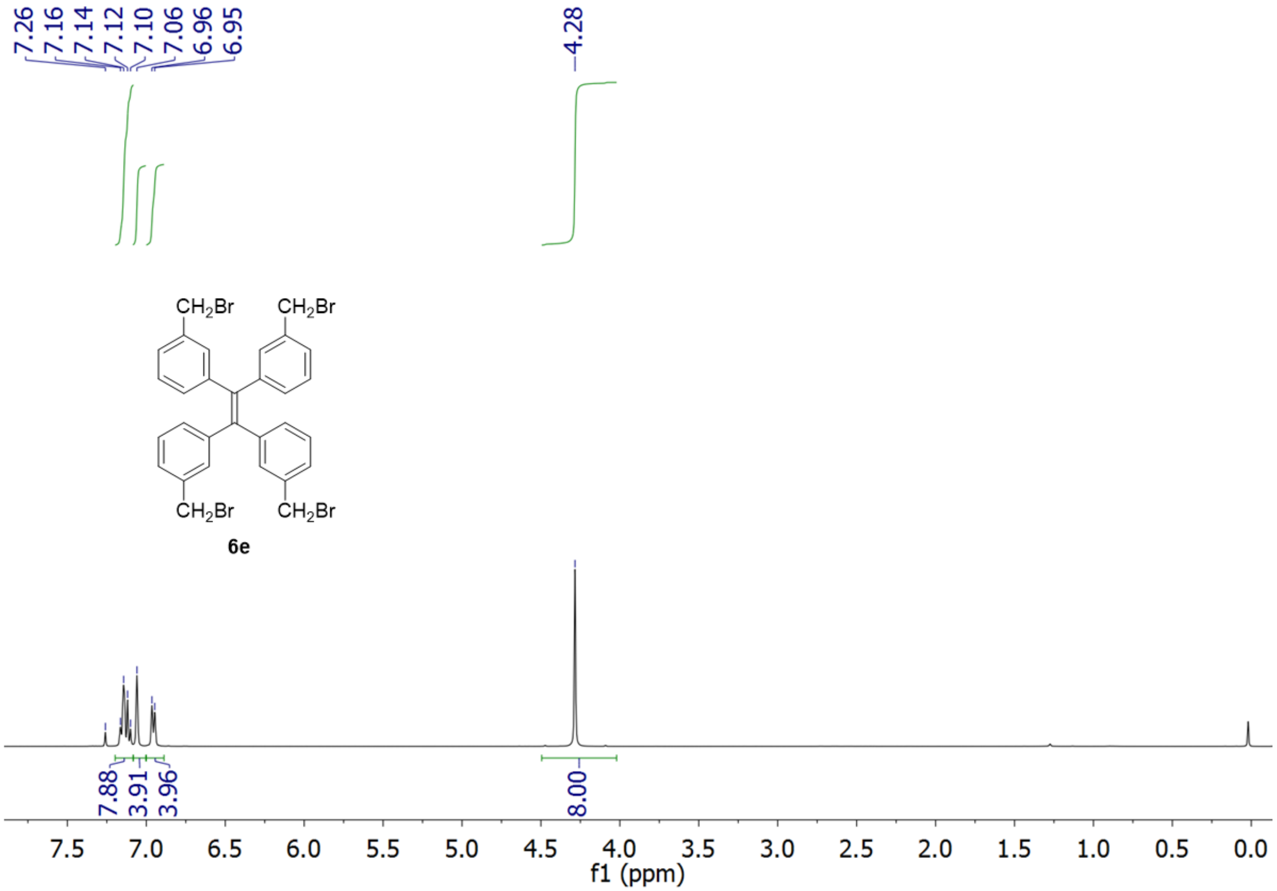

Figure S14. ^{1}H NMR spectrum of **6e** (400.132 MHz, r.t., $CDCl_3$/TMS).

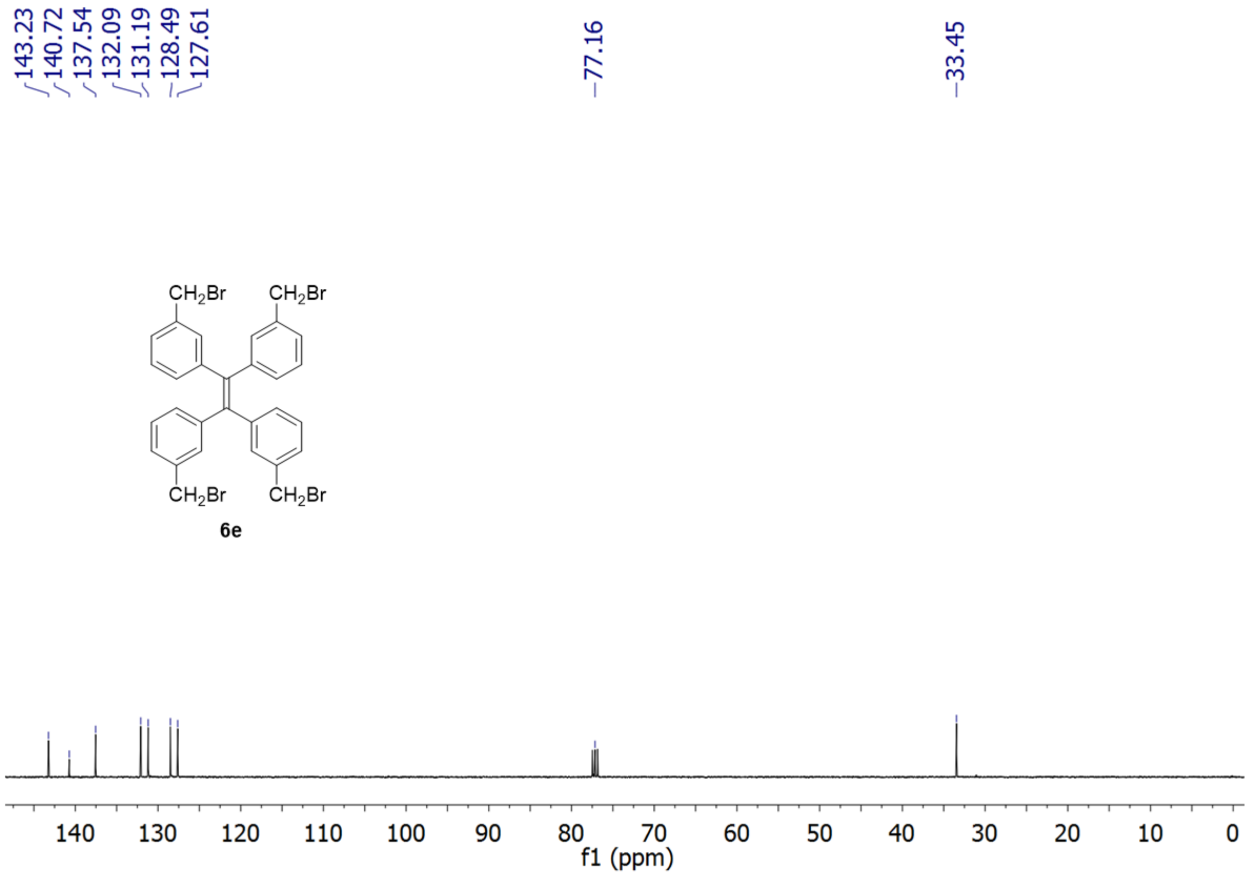

Figure S15. $^{13}C\{^1H\}$ NMR spectrum of **6e** (100.632 MHz, r.t., $CDCl_3$/TMS).

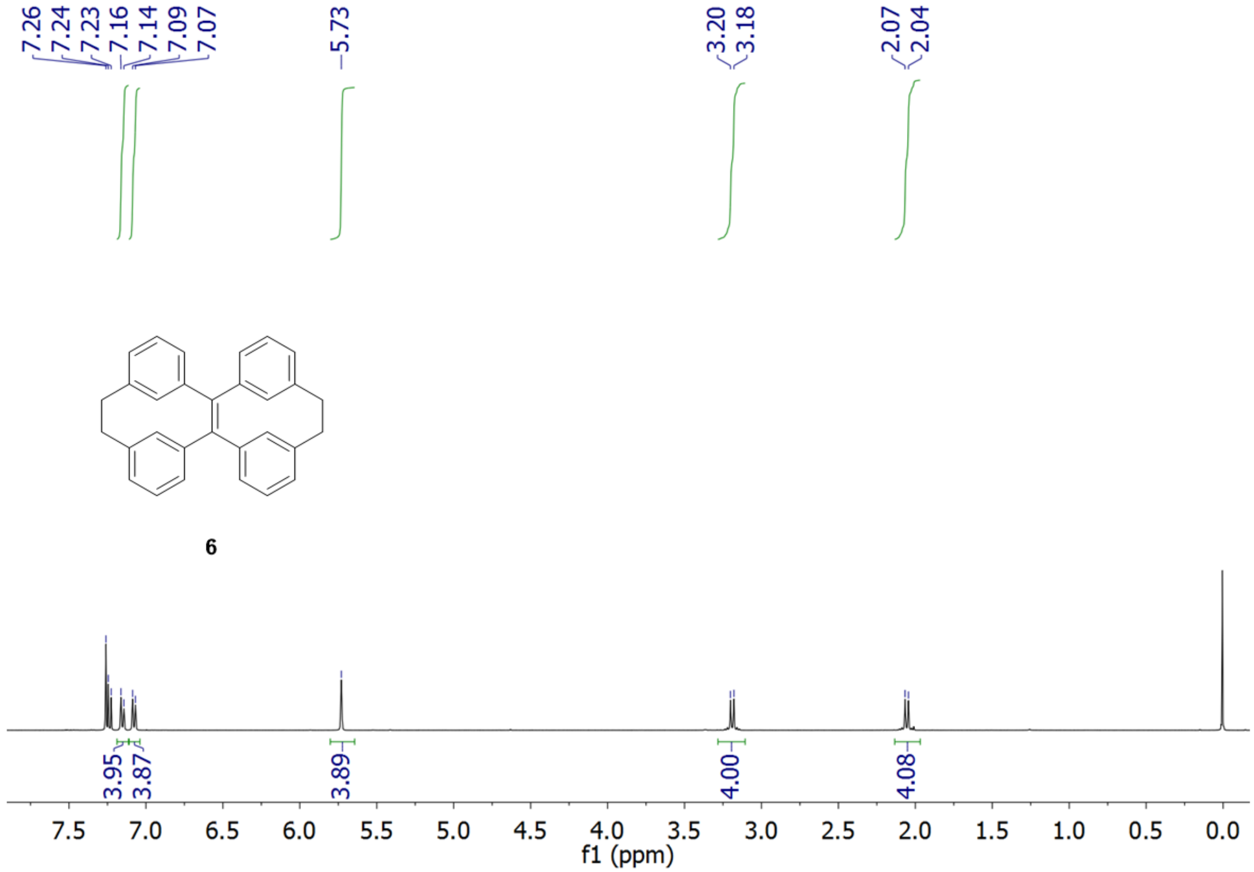

Figure S16. ^{1}H NMR spectrum of **6** (400.132 MHz, r.t., $CDCl_3$/TMS).

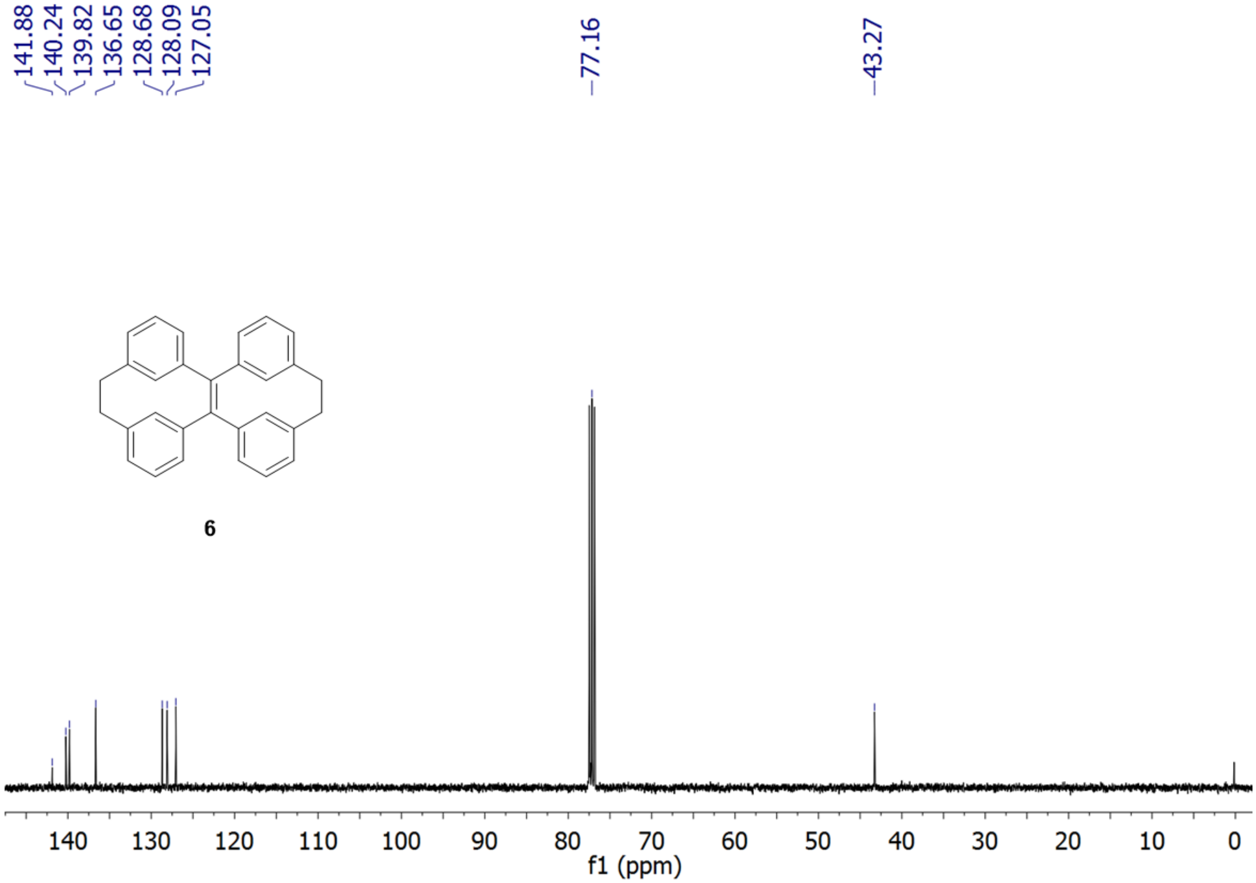

Figure S17. ^{13}C{^{1}H} NMR spectrum of **6** (100.632 MHz, r.t., $CDCl_3$/TMS).

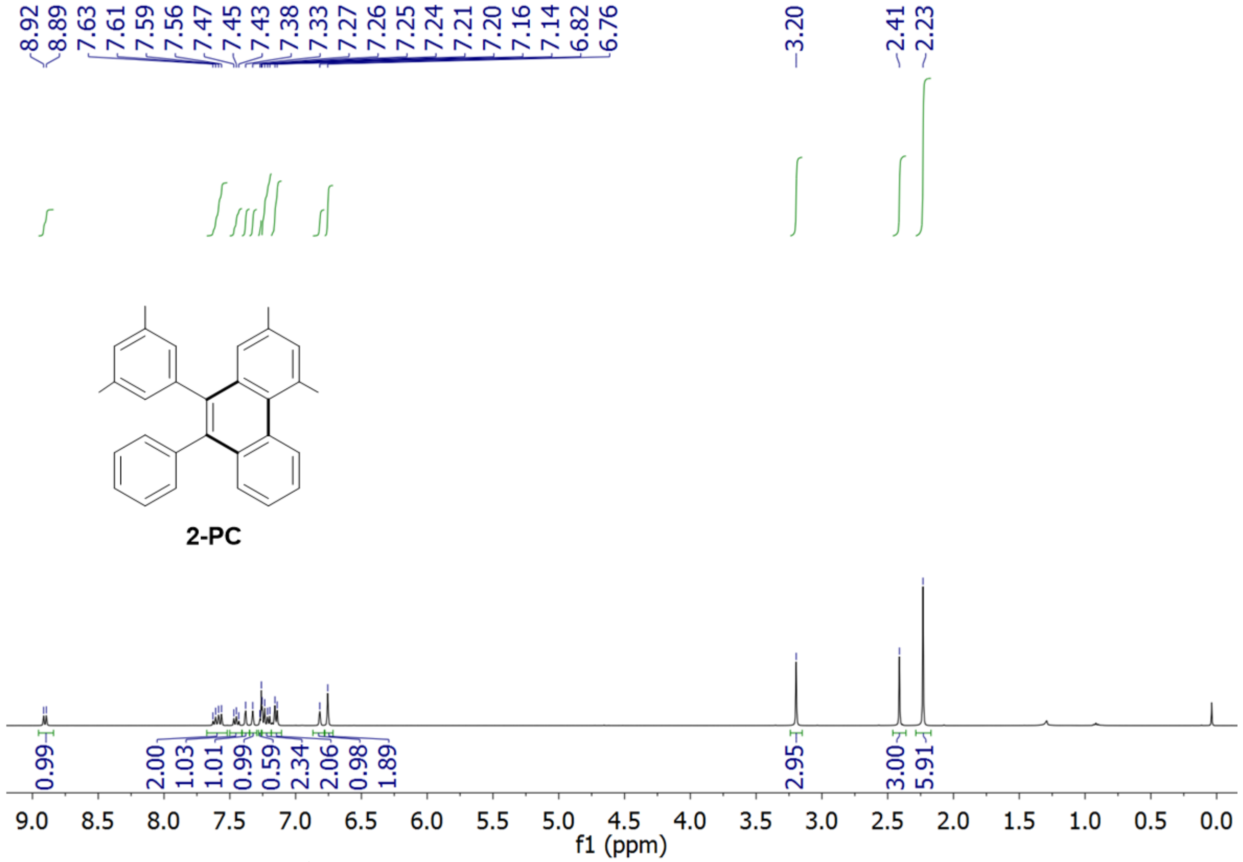

Figure S18. ^{1}H NMR spectrum of **2-PC** (400.132 MHz, r.t., $CDCl_3$/TMS).

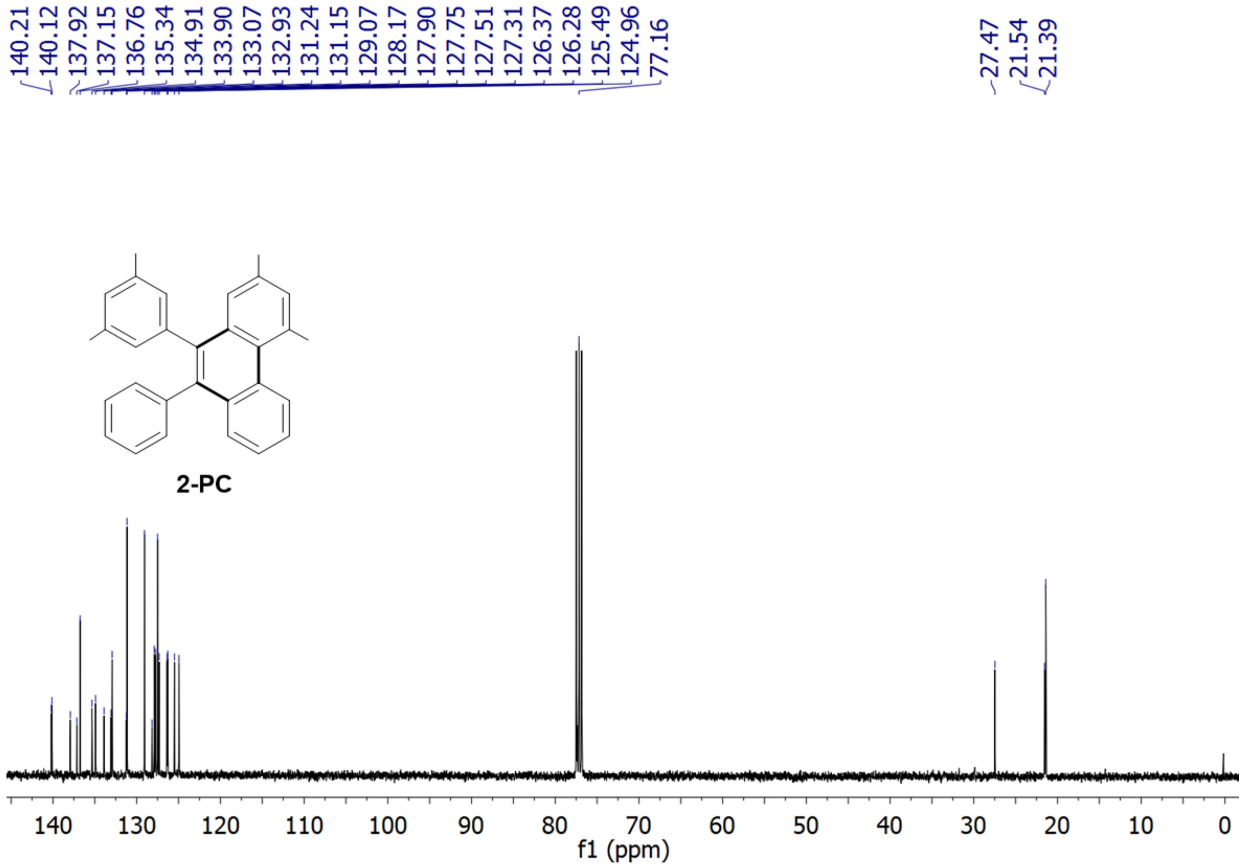

Figure S19. ^{13}C{^{1}H} NMR spectrum of **2-PC** (100.632 MHz, r.t., $CDCl_3$/TMS).

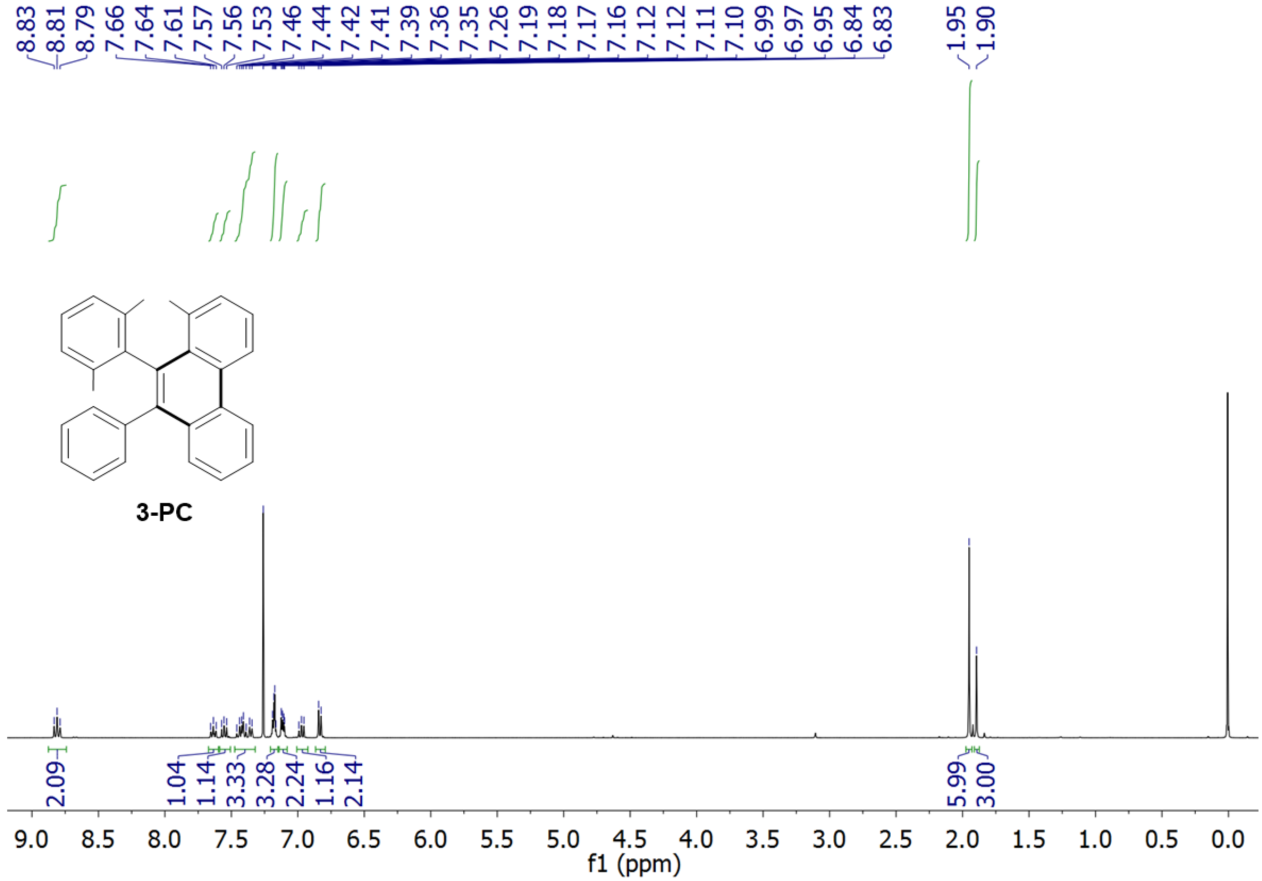

Figure S20. ^{1}H NMR spectrum of **3-PC** (400.132 MHz, r.t., $CDCl_3$/TMS).

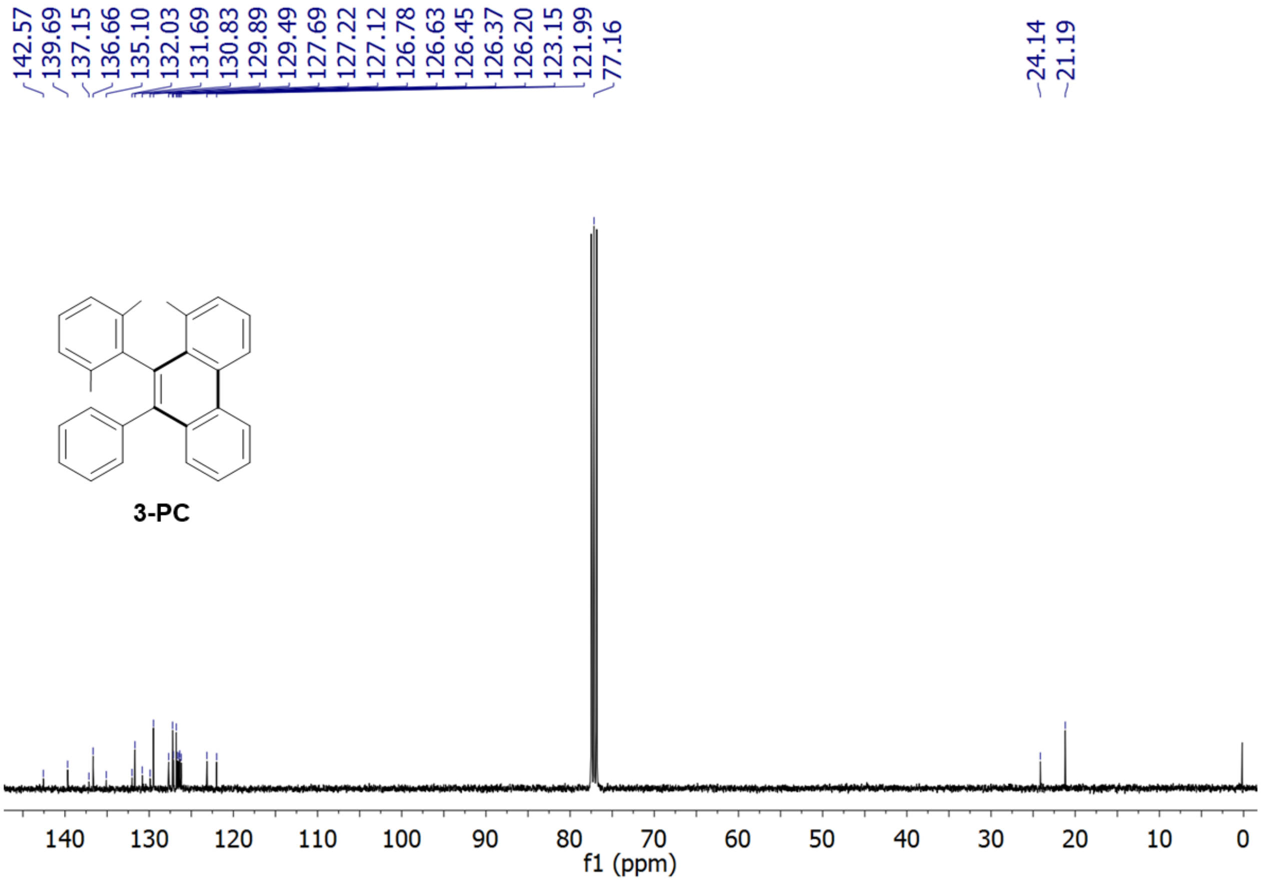

Figure S21. $^{13}C\{^{1}H\}$ NMR spectrum of **3-PC** (100.632 MHz, r.t., $CDCl_3$/TMS).

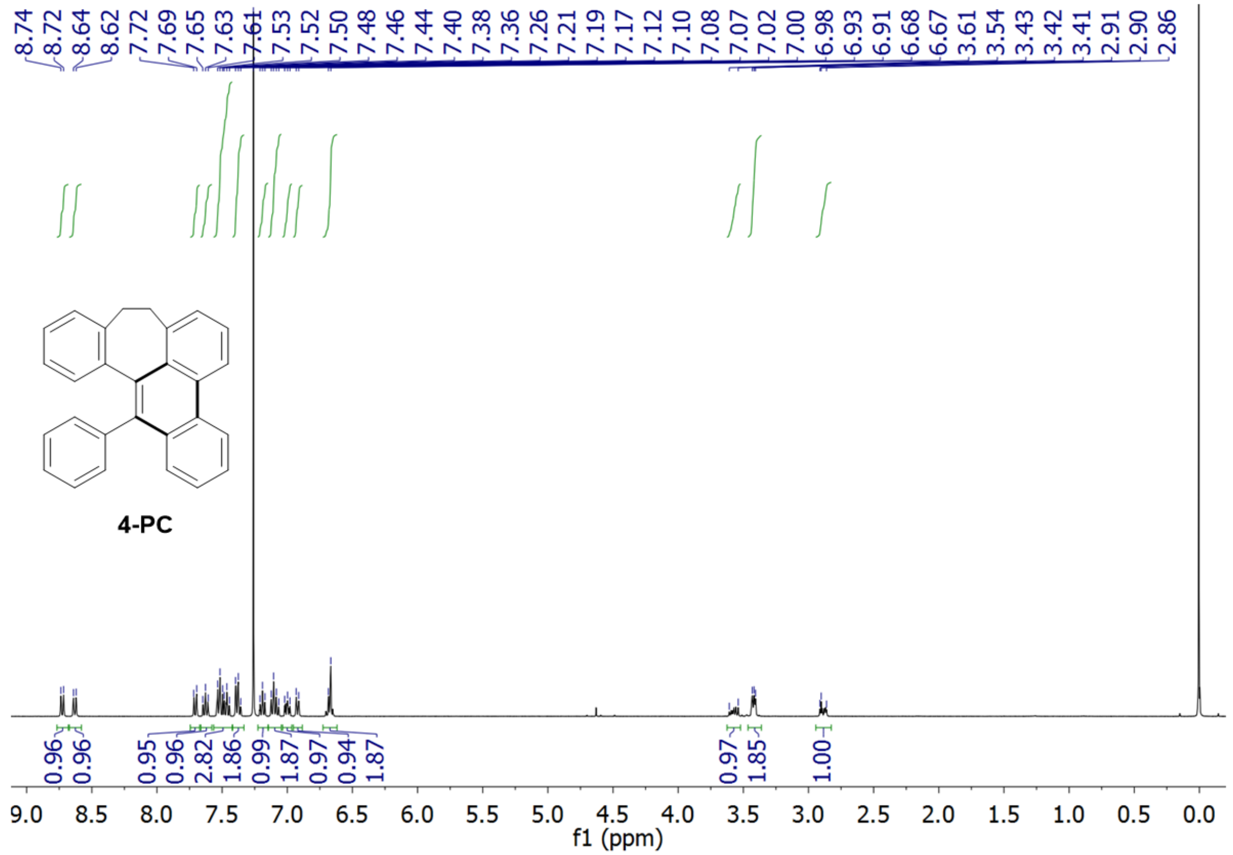

Figure S22. ^{1}H NMR spectrum of **4-PC** (400.132 MHz, r.t., $CDCl_3$/TMS).

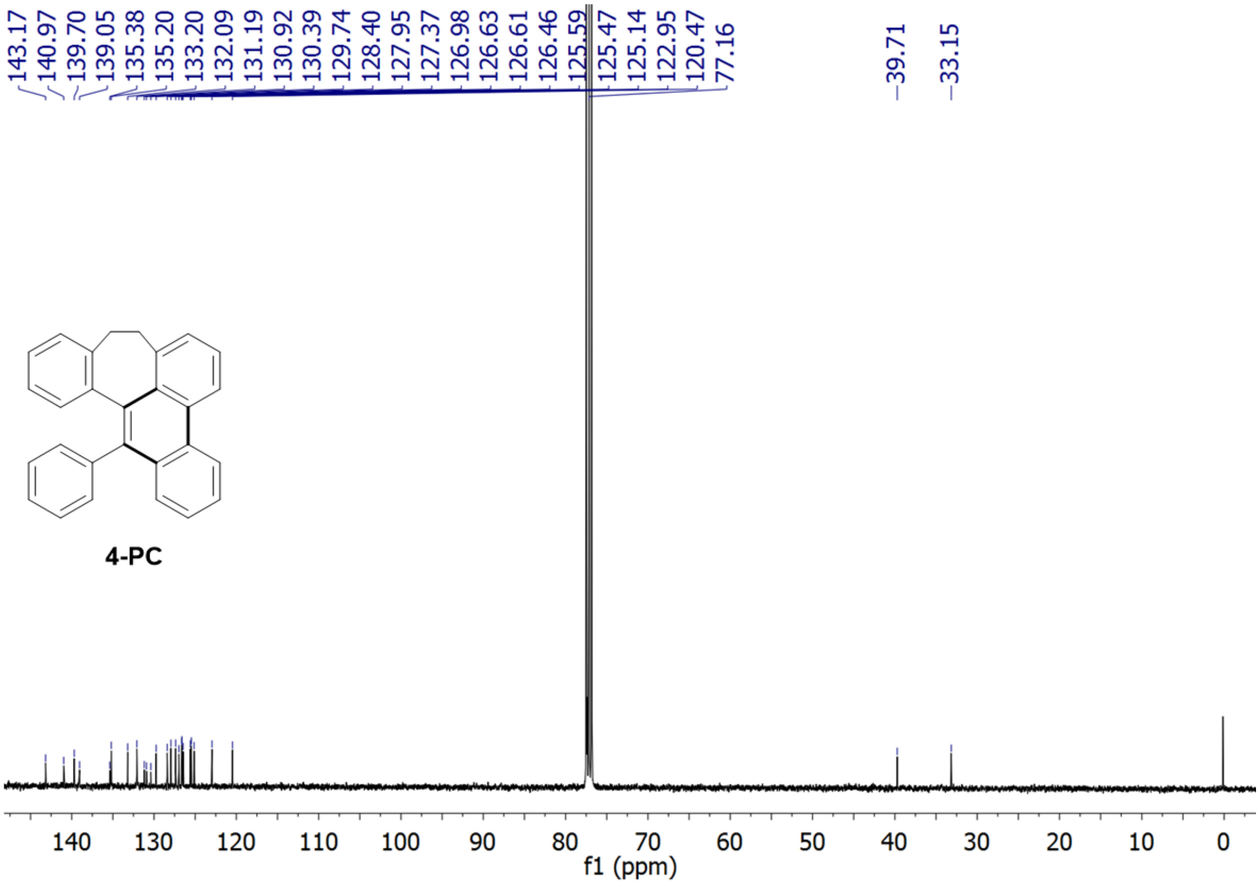

Figure S23. ^{13}C{^{1}H} NMR spectrum of **4-PC** (100.632 MHz, r.t., $CDCl_3$/TMS).

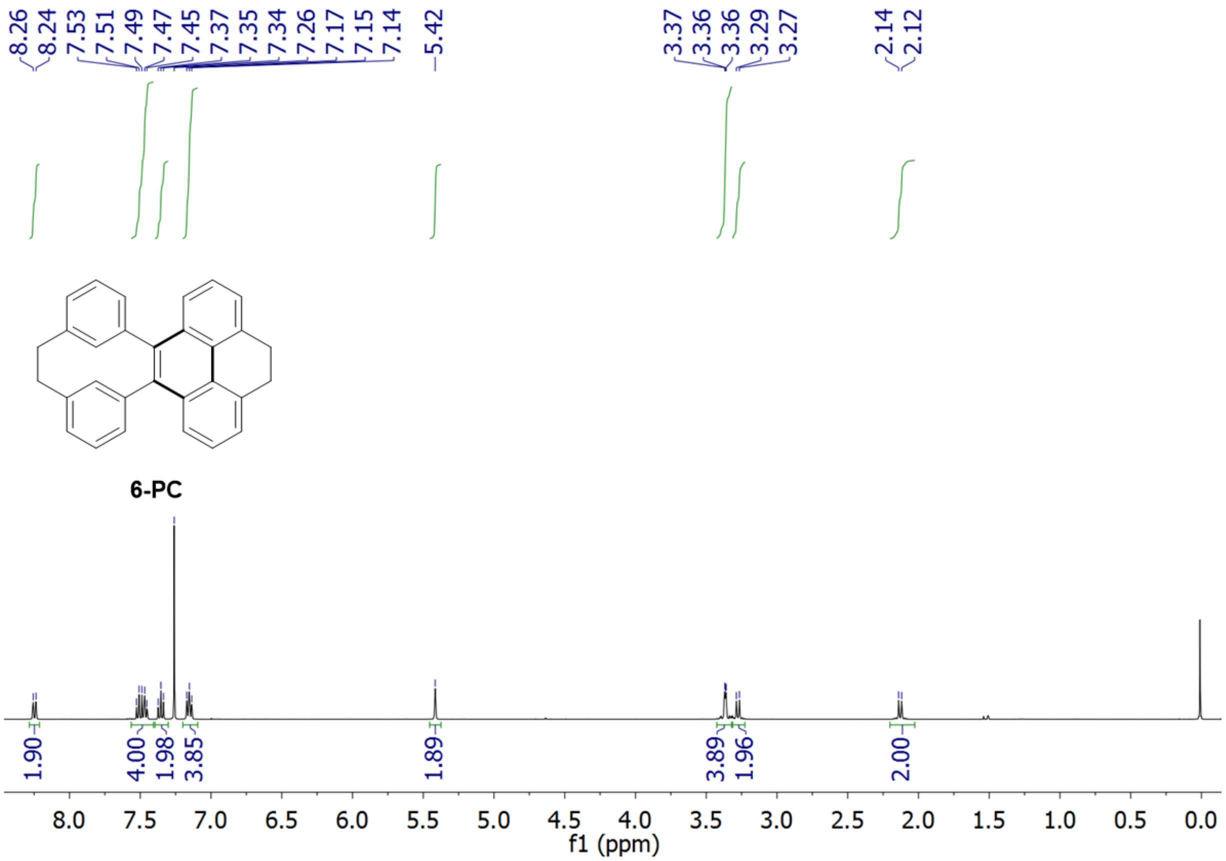

Figure S24. ^{1}H NMR spectrum of **6-PC** (400.132 MHz, r.t., $CDCl_3$/TMS).

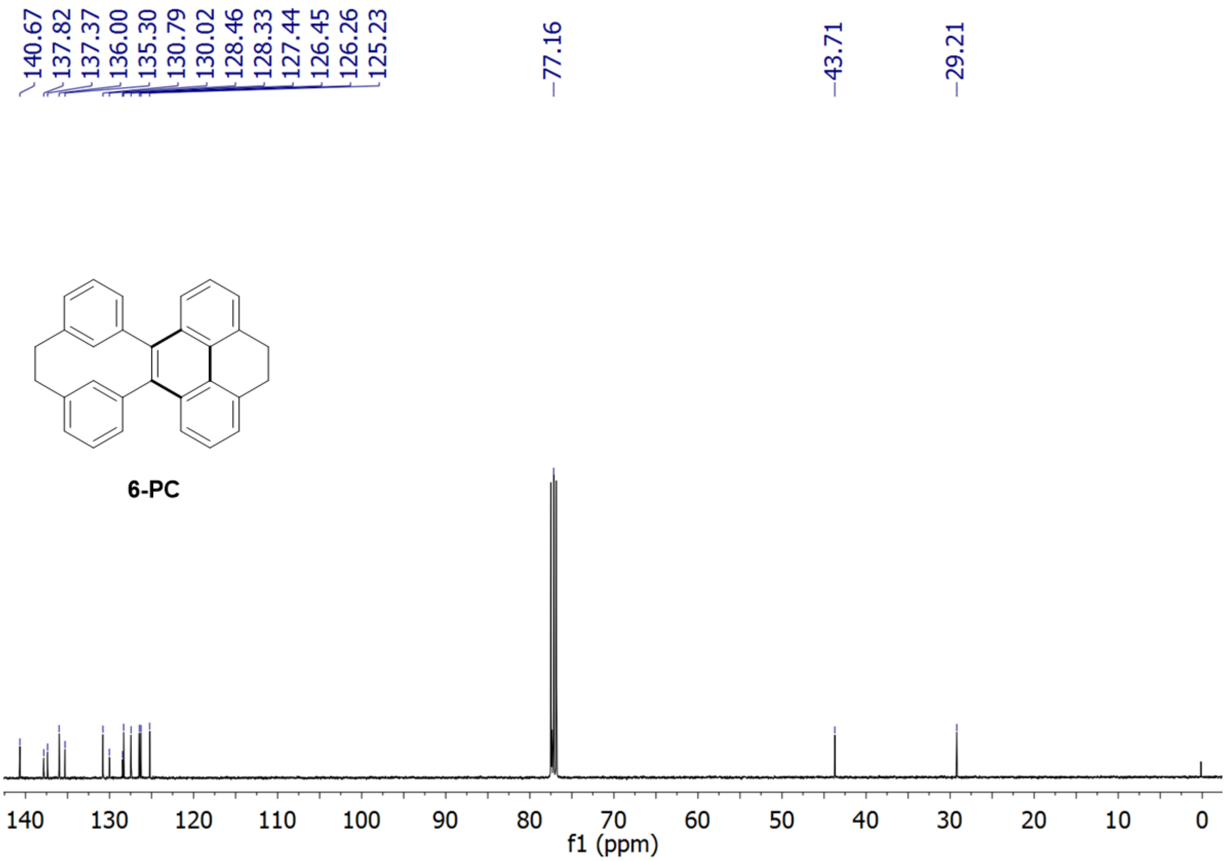

Figure S25. $^{13}C\{^{1}H\}$ NMR spectrum of **6-PC** (100.632 MHz, r.t., $CDCl_3$/TMS).

4 Single Crystal X-ray Crystallography

Suitable single crystals of TPE derivatives were selected under oil under ambient conditions. Single crystal X-ray diffraction intensity data were collected in a stream of cold nitrogen at 100K or at r.t. on a SuperNova, dual Cu/Mo Atlas diffractometer. Using Olex2, [15] the structure was solved with the ShelXS [16] structure solution program using Direct Methods and refined with the ShelXL [17] refinement package using Least Squares minimization. The details concerning x-ray crystallographic structure solutions and refinement for TPE derivatives are tabulated in **Table S1**.

Table S1. The crystallographic details of TPE derivatives.

	**2**	**3**	**4**	**6**
CCDC No.	1579022	1586452	1579026	1579025
empirical formula	$C_{30}H_{28}$	$C_{30}H_{28}$	$C_{28}H_{22}$	$C_{30}H_{24}$
formula weight	388.52	388.52	358.46	384.49
crystal dimensions, mm	0.35 × 0.3 × 0.28	0.4 × 0.35 × 0.35	0.60 × 0.12 × 0.04	0.18 × 0.15 × 0.13
crystal system	monoclinic	monoclinic	triclinic	monoclinic
space group	$P2_{1/c}$	$P2_1/n$	P-1	$P2_{1/c}$
a/Å	18.1203(5)	14.1772(3)	11.9827(13)	11.90924(15)
b/Å	6.99093(14)	10.8633(2)	13.0612(15)	17.5617(2)
c/Å	19.2375(5)	14.2137(3)	14.0684(17)	9.90343(13)
α/°	90	90	117.413(6)	90
β/°	112.450(3)	95.1137(16)	92.608(6)	101.3933(13)
γ/°	90	90	90.268(7)	90
Volume/Å^3	2252.27(11)	2180.36(7)	1951.7(4)	2030.45(5)
Z	4	4	4	4
ρ_{calc}, mg/mm^3	1.146	1.184	1.220	1.258
F(000)	832.0	832.0	760.0	816.0
λ/ Å	Mo Kα, λ = 0.71073	Mo Kα, λ = 0.71073	Mo Kα , 0.71073	Cu Kα, λ = 1.54184
μ/mm $^{-1}$	0.064	0.066	0.069	0.534
temperature/K	99.9(2)	100.00(10)	100.01(10)	100.01(10)
2θ range for data collection	6.394° to 52°	6.87° to 51.99	3.266° to 51.224°	9.096° to 133.996°
reflections collected	8156	12654	19701	10824
independent reflections. (R_{int})	4370(0.0156)	4222 (0.0226)	7147(0.0331)	3578(0.0127)
data/restraints/parameters	4370/0/275	4222/0/275	7147/1840/627	3578/0/271
goodness-of-fit on F^2	1.001	1.002	1.016	1.004
R_1,wR_2 [I>=2σ (I)]	0.0390, 0.0954	0.0371, 0.1066	0.0503, 0.1378	0.0352, 0.0869
R_1,wR_2 [all data]	0.0440, 0.0989	0.0482, 0.1132	0.0578, 0.1452	0.0369, 0.0883
largest diff. peak/hole / e Å^{-3}	0.22/-0.19	0.21/-0.19	0.25/-0.18	0.26/-0.19

Table S1. Continued.

	**2-PC**	**3-PC**	**4-PC**	**6-PC**
CCDC No.	1579024	1579027	1579021	1579023
empirical formula	$C_{30}H_{26}$	$C_{29}H_{24}$	$C_{28}H_{20}$	$C_{30}H_{22}$
formula weight	386.51	372.48	356.44	382.47
crystal dimensions, mm	0.4 × 0.35 × 0.02	0.35 × 0.08 × 0.05	0.2 × 0.18 × 0.04	0.08 × 0.06 × 0.05
crystal system	monoclinic	monoclinic	orthorhombic	monoclinic
space group	$P2_1/c$	$P2_1/c$	$P2_12_12_1$	C2/c
a/Å	17.4337(14)	7.3960(2)	8.34750(16)	18.2724(17)
b/Å	6.9903(4)	30.1314(11)	10.3140(2)	19.5178(15)
c/Å	17.9350(12)	18.3110(7)	21.0732(4)	18.301(2)
α/°	90	90	90	90
β/°	103.640(7)	91.801(3)	90	113.791(12)
γ/°	90	90	90	90
Volume/$Å^3$	2124.0(3)	4078.6(2)	1814.32(6)	5972.3(11)
Z	4	8	4	12
ρ_{calc}, mg/mm^3	1.209	1.213	1.305	1.276
F(000)	824.0	1584.0	752.0	2424.0
λ/ Å	Mo, Kα, λ = 0.71073	Mo, Kα, λ = 0.71073	Cu, Kα, λ = 1.54184	Cu, Kα, λ = 1.54184
μ/mm^{-1}	0.068	0.068	0.558	0.545
temperature/K	99.99(10)	99.93(16)	100.00(10)	220.03(10)
2θ range for data collection	6.306° to 51.998°	6.976° to 51.998°	9.548° to 133.954°	6.962° to 133.99°
reflections collected	12065	23858	3673	9314
independent reflections. (R_{int})	4123(0.0619)	7936(0.0529)	2661(0.0185)	5270(0.0533)
data/restraints/parameters	4123/0/275	7936/0/529	2661/0/253	5270/0/406
goodness-of-fit on F^2	1.003	1.001	1.003	1.000
R_1,wR_2 [I>=2σ (I)]	0.0615, 0.1268	0.0571, 0.1201	0.0285, 0.0690	0.0618, 0.1076
R_1,wR_2 [all data]	0.1085, 0.1463	0.0904, 0.1345	0.0326, 0.0713	0.1205, 0.1257
largest diff. peak/hole / e $Å^{-3}$	0.22/-0.28	0.25/-0.24	0.13/-0.14	0.27/-0.18

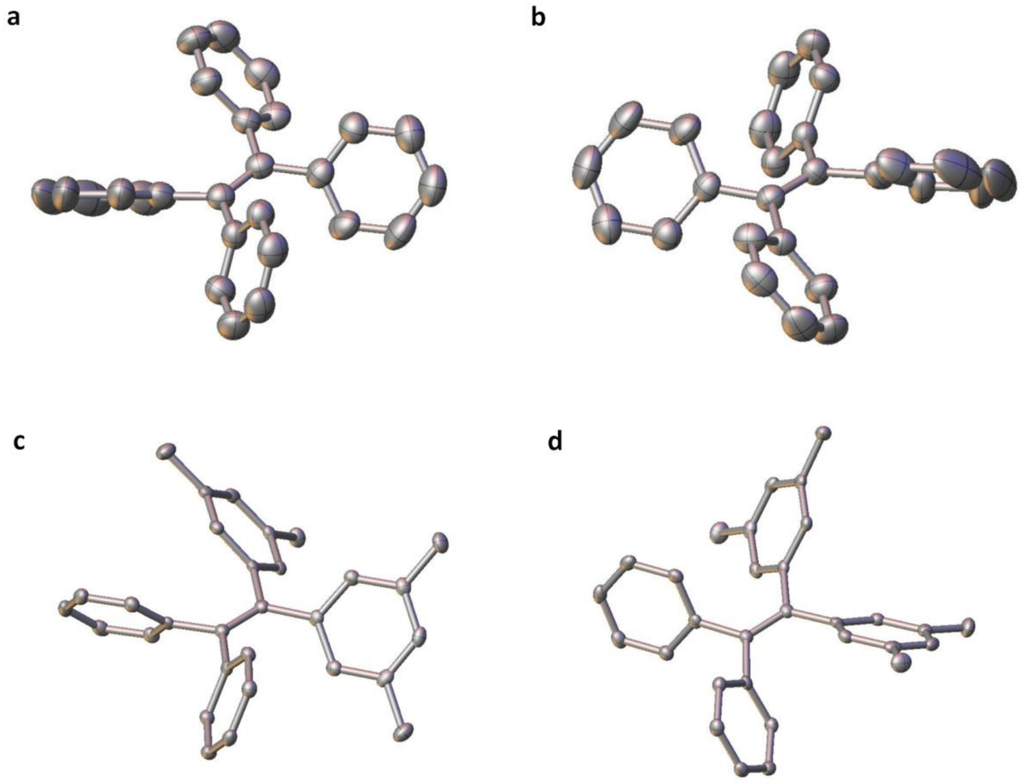

e

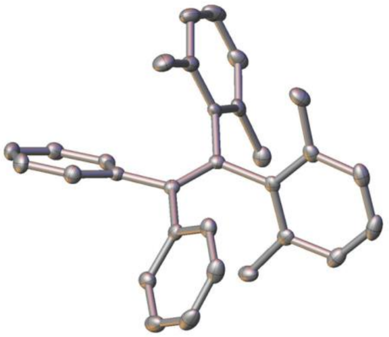

f

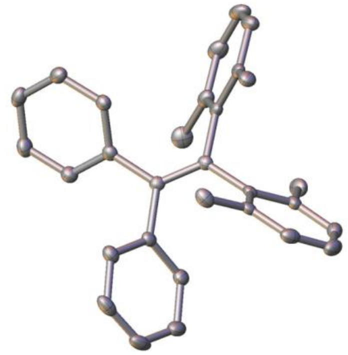

g

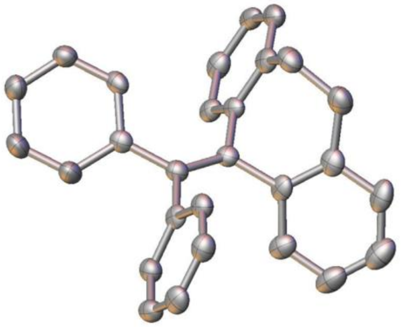

h

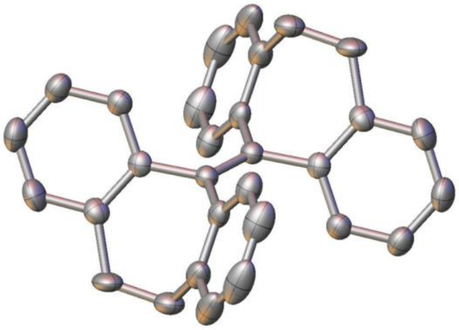

i

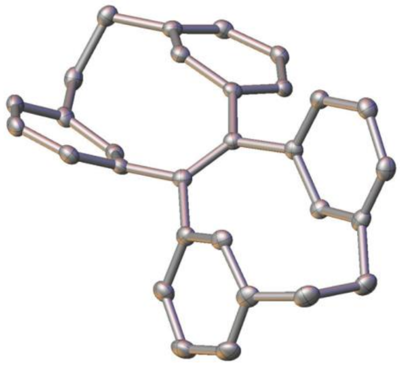

j

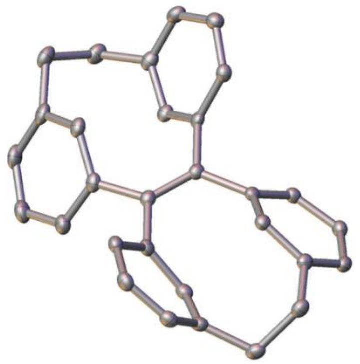

k

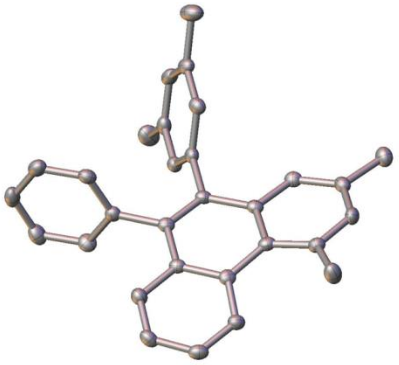

l

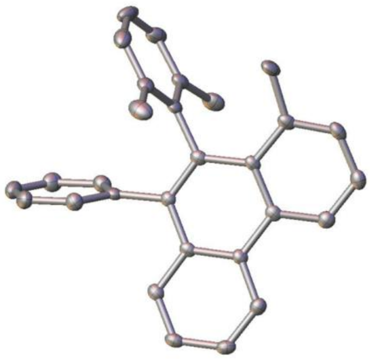

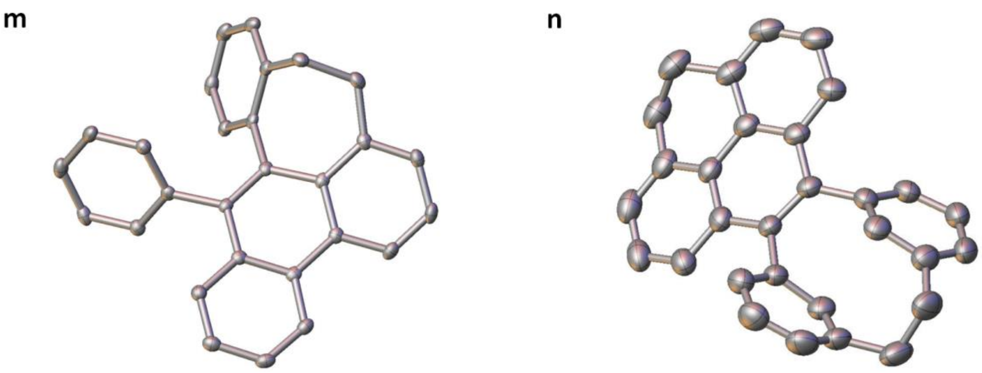

Figure S26. Thermal ellipsoids drawings of TPE derivatives: **(a) 1** (with phenyl group orientation anticlockwise); [18] **(b) 1** (with phenyl group orientation clockwise); [3] **(c) 2** (with phenyl group orientation anticlockwise); **(d) 2** (with phenyl group orientation clockwise); **(e) 3** (with phenyl group orientation anticlockwise); **(f) 3** (with phenyl group orientation clockwise); **(g) 4**; **(h) 5**; [5] **(i) 6** (with phenyl group orientation anticlockwise); **(j) 6** (with phenyl group orientation clockwise); **(k) 2-PC**; **(l) 3-PC**; **(m) 4-PC**; **(n) 6-PC**. All H atoms are omitted for clarity.

Since molecular structures of **1** and **6** have approximate D_2 geometrical symmetry, and these of **2** and **3** approximate C_2 geometrical symmetry in their respective single crystals, two different enantiomers of these compounds can be found in their repective unit cells. While molecular structure of **4** also has an approximate geometrical symmetry that belongs to a chiral point group (C_1), the two enantiomers of **4** have the same orientation of the phenyl groups and can be interconverted by a simple flip of the ethylene link between the two geminal phenyl groups it is connecting. Molecules of **5** in the solid state have an approximate C_i symmetry, thus displaying only one type of phenyl group orientation as well.

5 Photophysical properties

The UV-vis absorption spectra were obtained using a UV-vis spectrometer (Shimadzu, UV-2600, Japan). The PL measurements were carried out on a Horiba Fluoromax-4 spectrofluorometer. The absolute fluorescence quantum yields were measured using a Hamamatsu quantum yield spectrometer C11347 Quantaurus_QY. The hand-held UV lamp used in the experiment is equipped with 365nm/254nm 6 Watt UV Tubes (ENF-260C/FBE: BLE-6T365, BLE-6254S). The solvents used in the measurement of photophysical properties were: tetrahydrofuran (spectrophotometric grade, Merck) and acetonitrile (99.9%, Energy Chemical Company, dried by molecular sieves). The compounds **1**, **1-PC**, **3**, **5** and **5-PC** have already been reported and characterized somewhere else. [1-3, 5, 19] Similar photo-induced changes in the spectra of the TPE derivatives can be found in the literature [20].

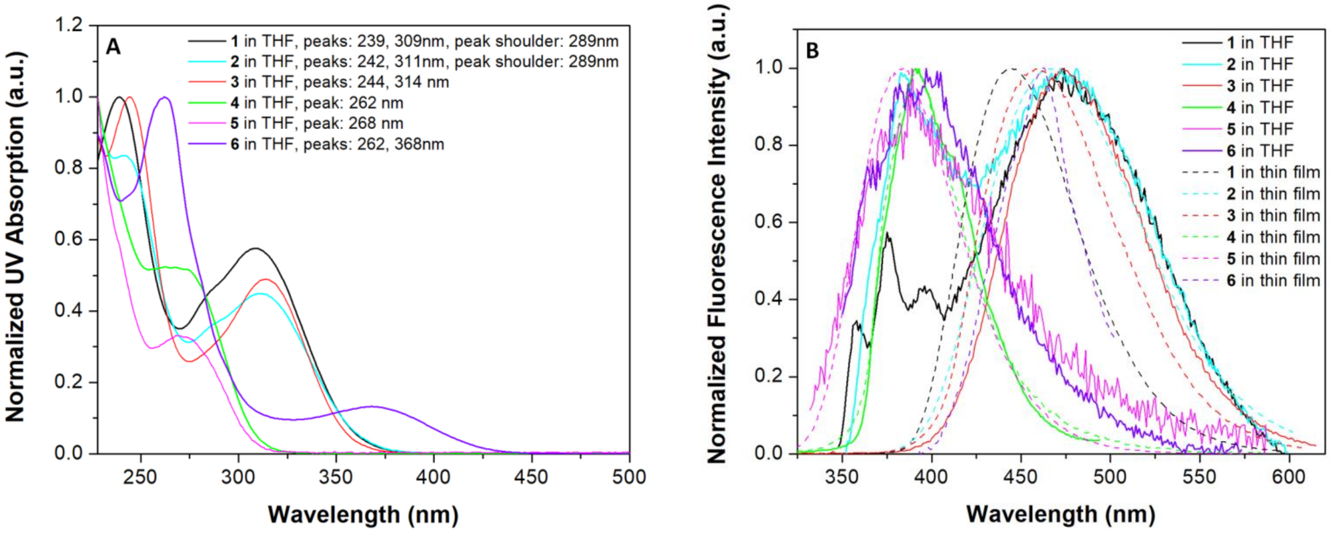

Figure S27. (A) UV-Vis spectra of **1-6** in THF solution (10^{-5} M); (B) PL spectra of **1-6** in THF solution (10^{-5} M) and in thin film *under the first time excitation*: **1** in THF, Ex308nm, slit5, PL peaks: 358, 375, 396, 474nm; **2** in THF, Ex310nm, slit5, PL peaks: 383, 472nm; **3** in THF, Ex314nm, slit1, PL peak: 473nm; **4** in THF, Ex276nm, slit5, PL peak: 391nm; **5** in THF, Ex300nm, slit2, PL peak: 389 nm; **6** in THF, Ex300nm, slit7, PL peak: 397nm, peak shoulders: 370, 387nm; **1** in thin film, Ex308nm, slit3, PL peak: 445nm; **2** in thin film, Ex310nm, slit5, PL peak: 466nm; **3** in thin film, Ex314nm, slit3, PL peak: 460nm; **4** in thin film, Ex305nm, slit5, PL peak: 393nm; **5** in thin film, Ex300nm, slit2.5, PL peak: 384nm; **6** in thin film, Ex330nm, slit15, PL peak: 462nm.

1-3 in solutions display similar UV absorption peaks at around 310 nm, the UV absorption maxima of **4** and **5** are markedly blue shifted (< 270 nm), and the absorption maximum of **6** is notably red shifted (368 nm).

While **1-6** in thin film show symmetric fluorescence spectra with only one emission peak, dilute solutions (10^{-5} M) of **1-2** and **4-6** in THF upon first excitation do not display symmetric spectra due to the photocyclization reaction. Extra peaks at 358 nm, 375 nm and 396 nm appear spontaneously in **1** upon excitation, which are similar to the classical fluorescence peaks of diphenylphenanthrene, [2] indicating that **1** undergoes photocyclization reaction upon first time excitation and new species **1-PC** might form. Other TPE derivatives have similar extra emission peaks upon the first excitation. Due to their different structures, they either show up separately (**2**) or overlap (**4-6**) with the emission peaks of the original compounds. However, a dilute solution (10^{-5} M) of **3** in THF has a symmetric fluorescent spectrum with a maximum peak at 473 nm upon the first excitation, and no obvious extra peaks were observed, indicating that **3** has the lowest yield of the formation of the newly photocyclized species in the TPE derivatives under consideration in this work.

5.1 Photophysical properties of 1 and 1-PC

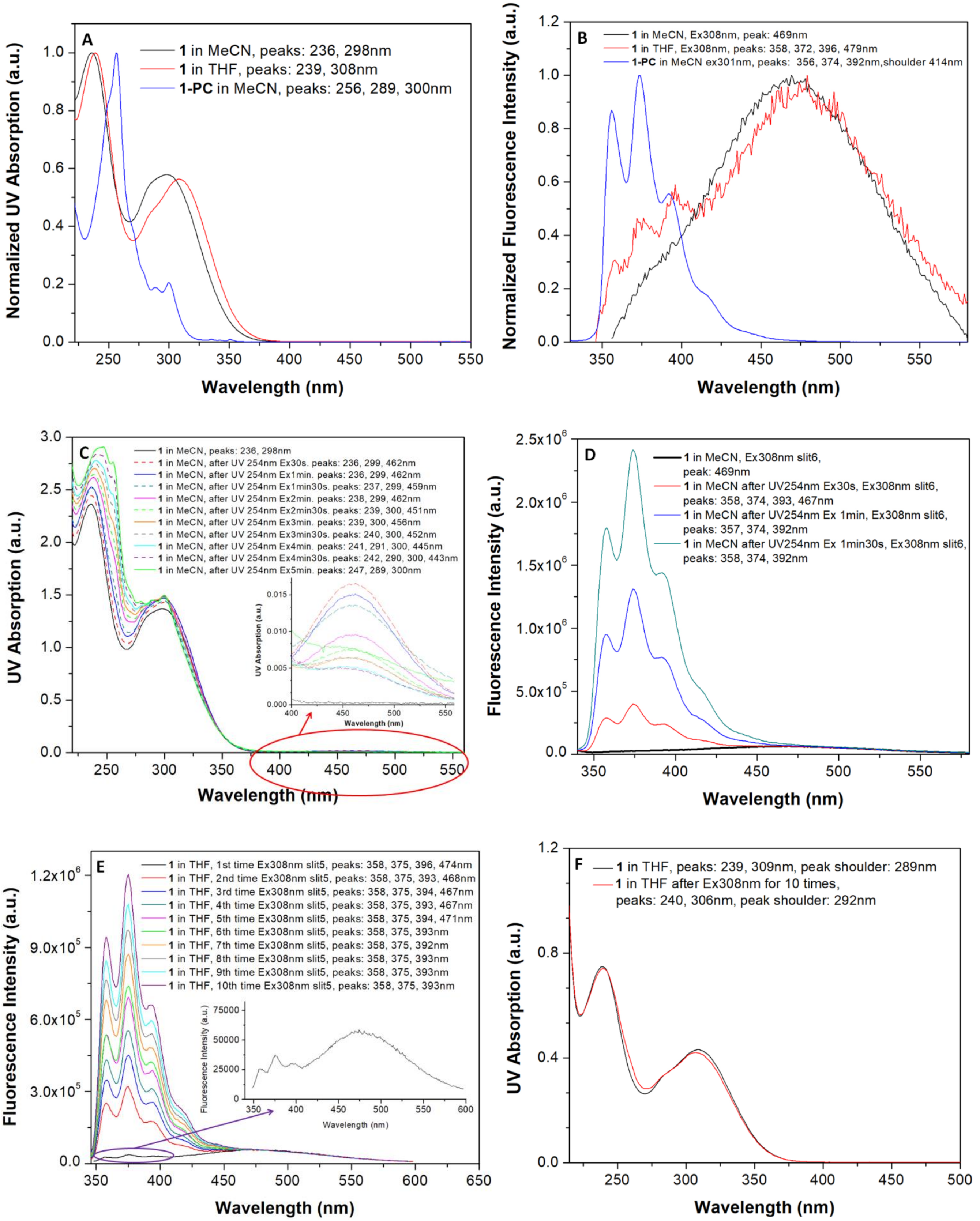

Figure S28. (A) UV-Vis spectra of **1** and **1-PC** in solution; (B) PL spectra of **1** and **1-PC** in solution; (C) UV-Vis spectra of **1** in MeCN after irradiating the sample 10 times (30s each time) at 254 nm using a hand-held UV lamp. (D) PL spectra of **1** in MeCN after irradiation at 254 nm using a hand-held UV

lamp; (E) PL spectra of **1** in THF (2.74×10^{-5} mol/L) before and after irradiation at 308 nm for 10 times in the PL fluorimeter; (F) UV-Vis spectra of **1** in THF (2.74×10^{-5} mol/L) before and after irradiation at 308 nm 10 times in the PL fluorimeter.

The UV absorption and PL emission spectra of **1** in MeCN are similar to those of **1** in THF (Figure S28. A, B). Although extra emission peaks at 358 nm, 372 nm and 396 nm due to the formation of the photocyclized product appear in the PL spectra of **1** in THF, they are not obvious as only a shoulder appears in the PL spectrum of **1** in MeCN upon the first time excitation. However, upon irradiating **1** in MeCN for a longer time (Figure S28. C, D), **1** undergoes photocyclization as evidenced by the growing intensity of the emssion peaks at 358 nm, 374 nm and 396 nm, which are similar to the emission peaks of **1-PC** (Figure S28. B). Similar PL spectra of **1** in THF can be observed after a short time irradiation (Figure S28. E). The UV absorption spectra change gradually with two new peaks at 289 nm and 300 nm emerging after 5 min irradiation, which are the same absorption peaks of **1-PC** in MeCN (Figure S28. A). During UV irradiation, we observed appearance of a very small peak at 462 nm with an extra low absorption intensity in the UV absorption spectra (Figure S28. C) with the decreased intensity as time increases, which can be attributed to the photocyclized intermdediate **1-IM** (Figure S1). The results suggest that **1** could be converted into **1-PC** upon UV irradiation in the presence of oxygen, and it requires a certain time of irradiation for the transformation to be completed. [20]

5.2 Photophysical properties of 2 and 2-PC

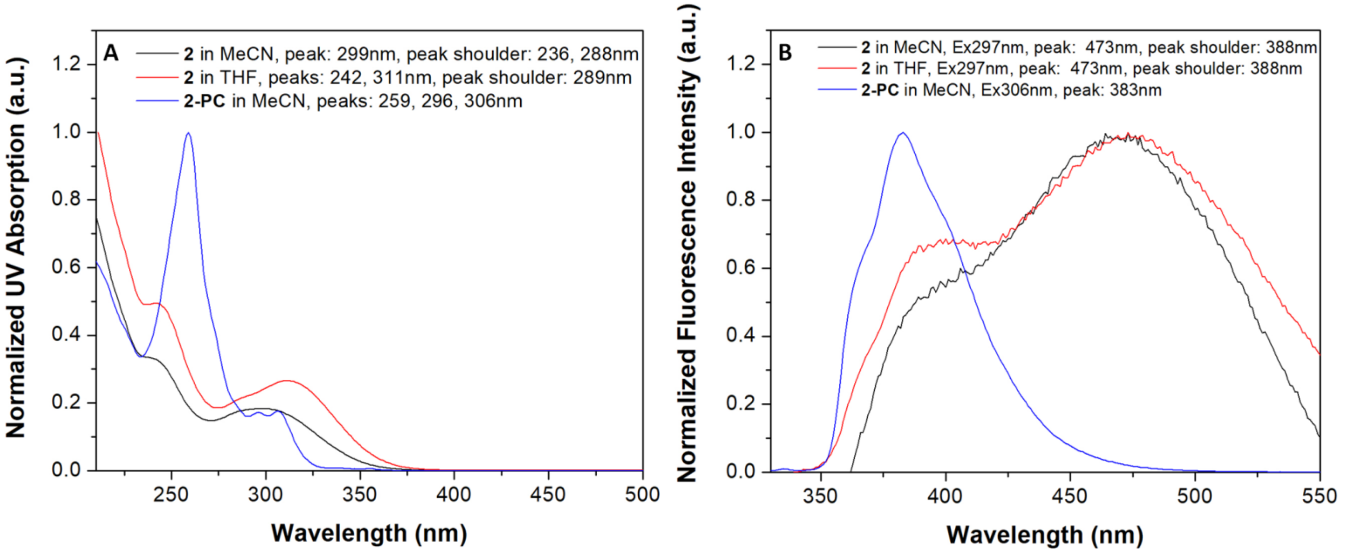

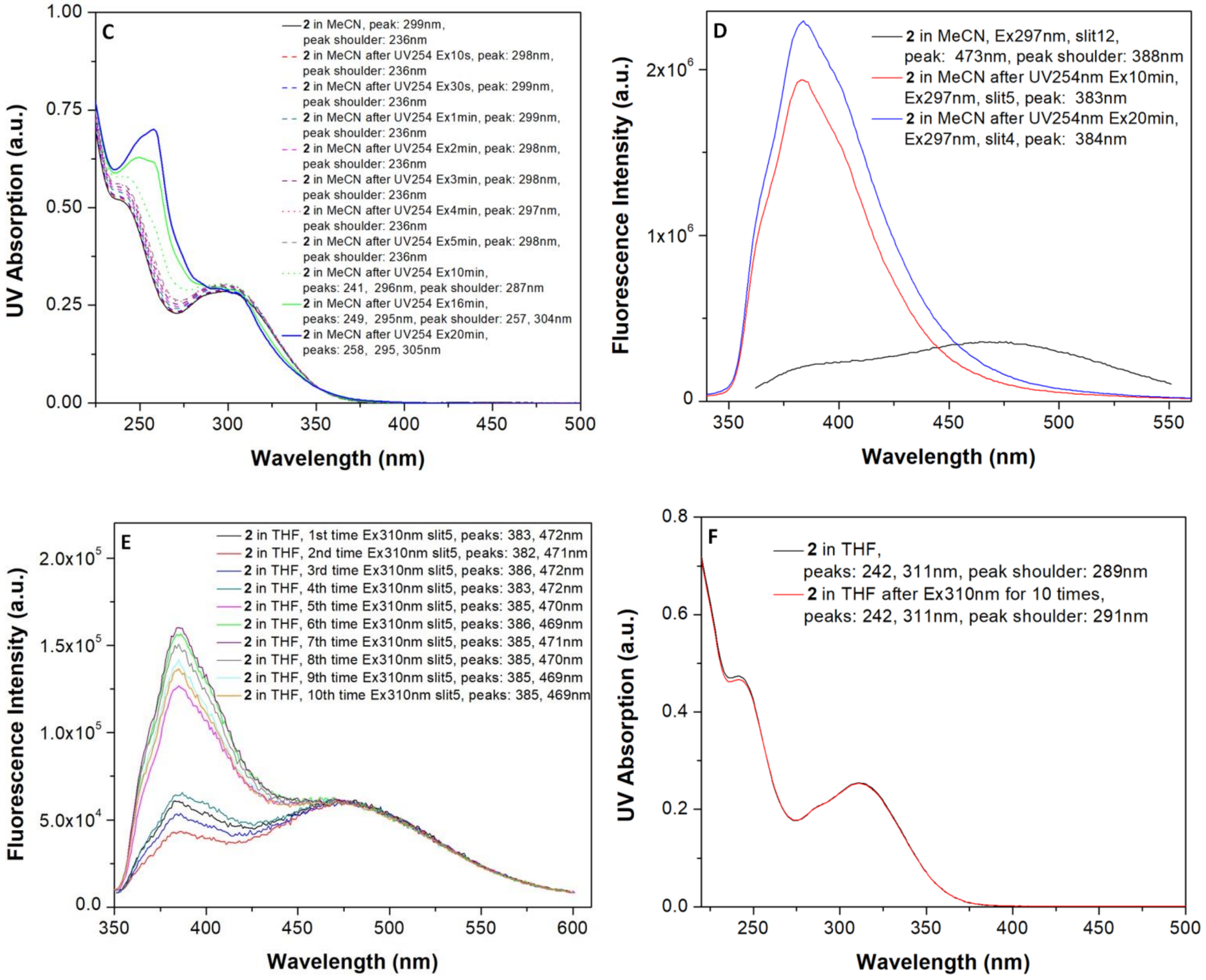

Figure S29. (A) UV-Vis spectra of **2** and **2-PC** in solution; (B) PL spectra of **2** and **2-PC** in solution; (C) UV-Vis spectra of **2** in MeCN after irradiating the sample at 254nm 10 times (10s for the first time, 30s for the second time, 1 min each for the following 5 times, 5 min for the 8th time, 6 min for the 9th time, 4 min for the 10th time) using a hand-held UV lamp; The transparent color of the solution **2** in MeCN *does not change obviously* under the UV lamp excitation at 254nm; the photo-cyclized product **2-PC** is formed after *20 min* UV excitation. (D) PL spectra of **2** in MeCN after irradiating the sample at 254nm using a hand-held UV lamp; (E) PL spectra of **2** in THF (1.69×10^{-5} mol/L) before and after 10 times excitation at 310 nm in the PL fluorimeter; (F) UV-Vis spectra of **2** in THF (1.69×10^{-5} mol/L) before and after 10 times excitation at 310 nm in the PL fluorimeter.

Similar changes in the fluorescence and absorption spectra were observed for **2** in THF and MeCN after UV irradiation as observed in **1**. Compound **2** undergoes photocyclization after UV irradiation with newly emerged absorption peaks (258 nm, 295 nm, 305 nm) (Figure S29.C) and increased fluorescence intensity at 385 nm (Figure S29. E), which are very similar to those peaks in its photocyclized product **2-PC**.

5.3 Photophysical properties of 3 and 3-PC

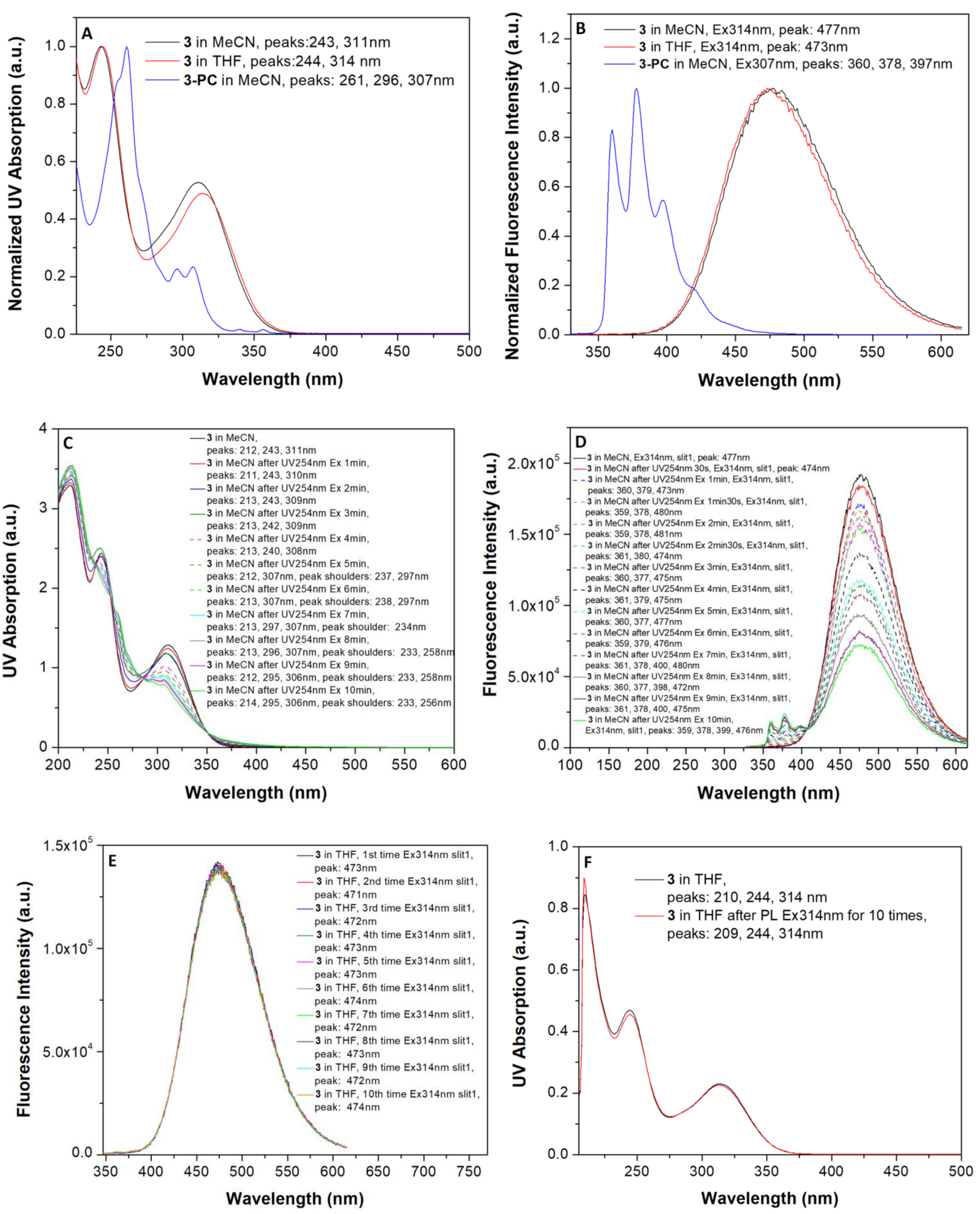

Figure S30. (A) UV-Vis spectra of **3** and **3-PC** in solution; (B) PL spectra of **3** and **3-PC** in solution; (C) UV-Vis spectra of **3** in MeCN after irradiating the sample at 254nm 10 times (1min each time) using a hand-held UV lamp; The transparent color of the solution **3** in MeCN *does not change* under the UV

lamp excitation at 254nm; the photo-cyclized product **3-PC** is formed after long time UV excitation. (D) PL spectra of **3** in MeCN after irradiating the sample at 254nm using a hand-held UV lamp; the PL spectra *does not change* under UV irradiation at 254nm *for the first 30s*; (E) PL spectra of **3** in THF (1.35×10^{-5} mol/L) before and after 10 times excitation at 314 nm in the PL fluorimeter; (F) UV-Vis spectra of **3** in THF (1.35×10^{-5} mol/L) before and after 10 times excitation at 314 nm in the PL fluorimeter.

The absorption and fluorescence spectra of **3** after UV irradiation show that only after a significantly longer irradiation time, new absorption peaks (295 nm, 306 nm) and emission peaks (359 nm, 378 nm, 399 nm) emerge (Figure S30. C, D), which are rather similar to those peaks of **3-PC**. This result reveals that **3** can still undergo photocyclization even if there is steric hindrance due to methyl groups in the *o*-position. As compared to **1** and **2**, the conversion of **3** to **3-PC** is not efficient within the short period of time upon excitation (Figure S30. E).

5.4 Photophysical properties of 4 and 4-PC

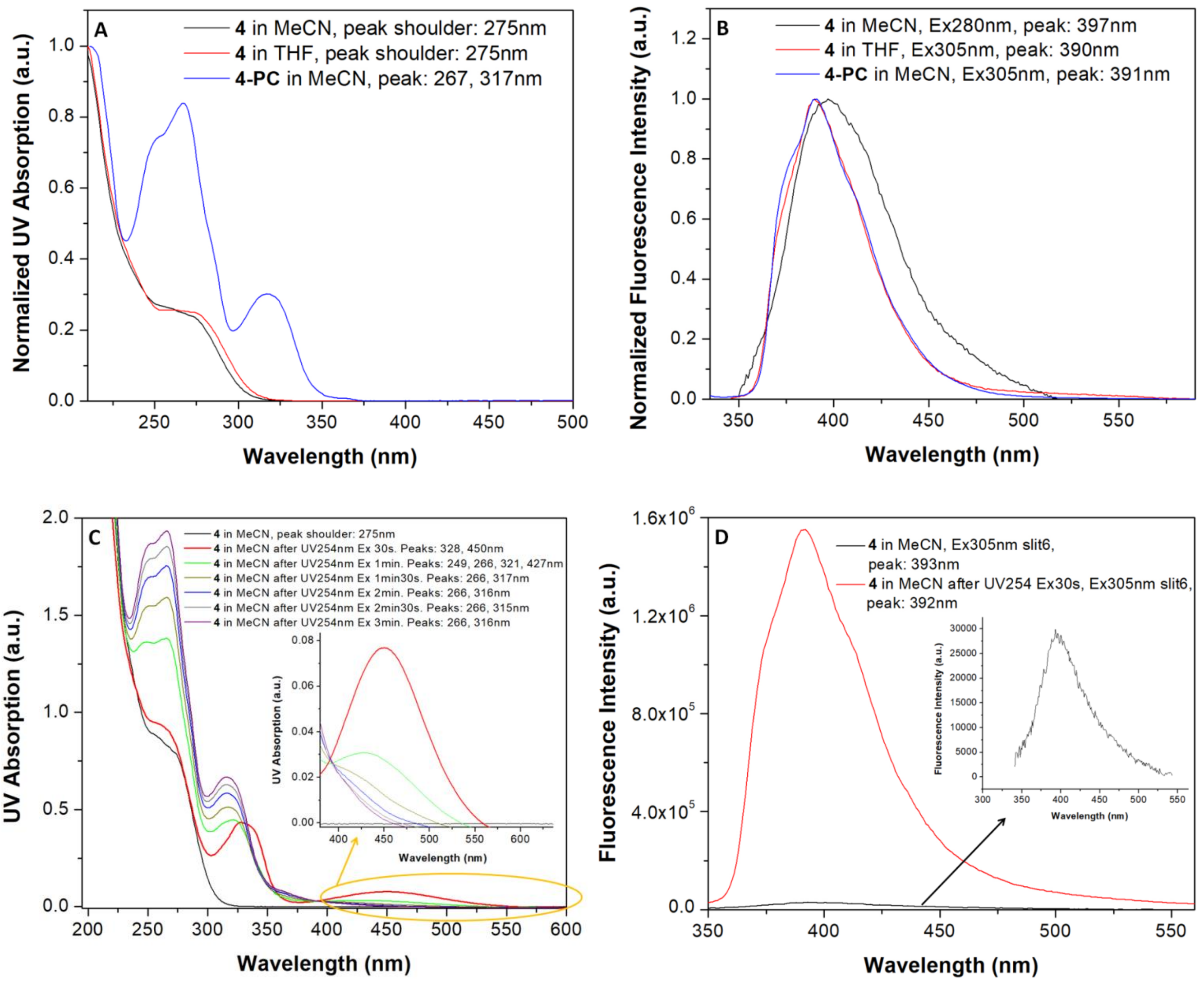

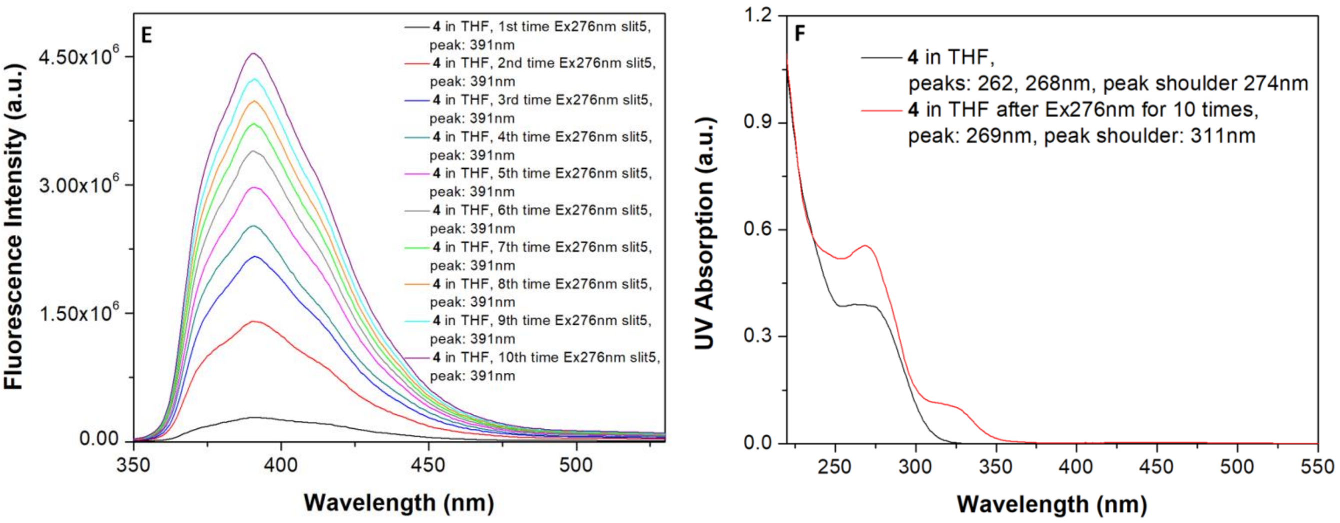

Figure S31. (A) UV-Vis spectra of **4** and **4-PC** in solution; (B) PL spectra of **4** and **4-PC** in solution; (C) UV-Vis spectra of **4** in MeCN after irradiating the sample at 254nm 6 times (30s each time) using a hand-held UV lamp; The transparent color of the solution **4** in MeCN turns to *pale yellow* under the UV lamp excitation at 254nm within seconds, as evidenced by a new UV peak at 450 nm emerging and this color can last for minutes, then the light yellow color turns to transparent with the UV peak at 450 nm disappearing, and the photo-cyclized product **4-PC** is formed. (D) PL spectra of **4** in MeCN after irradiating the sample at 254nm for 30s using a hand-held UV lamp; (E) PL spectra of **4** in THF (2.79×10^{-5} mol/L) before and after 10 times excitation at 276 nm in the PL fluorimeter; (F) UV-Vis spectra of **4** in THF (2.79×10^{-5} mol/L) before and after 10 times excitation at 276 nm in the PL fluorimeter.

The interesting phenomenon of **4** in solution after UV irradiation is that the transparent color of the solution turns to pale yellow which lasts for more than a min. This pale-yellow color can be attributed to the long-lived photocyclized intermediate **4-IM**. The yellow color is also evidenced by the UV-Vis absorption spectra of **4** after excitation (Figure S31. C), in which a new absorption peak at 450 nm emerges after 30s irradiation. Additionally, new absorption peaks at 266 nm and 316 nm emerge in the UV spectra of **4** in MeCN after 3 min UV irradiation, which are similar to those in the UV spectrum of **4-PC**. **4** in MeCN shows an asymmetric emission peak with a maximum emission at 397 nm. The intensity of this peak grows rapidly with two obvious shoulders emerging after UV irradiation for just 30s (Figure S31. D). Similar changes in absorption and fluorescence spectra can be observed by **4** in THF after UV irradiation, indicating that **4** can be converted into **4-PC** upon UV light irradiation.

5.5 Photophysical properties of 5 and 5-PC

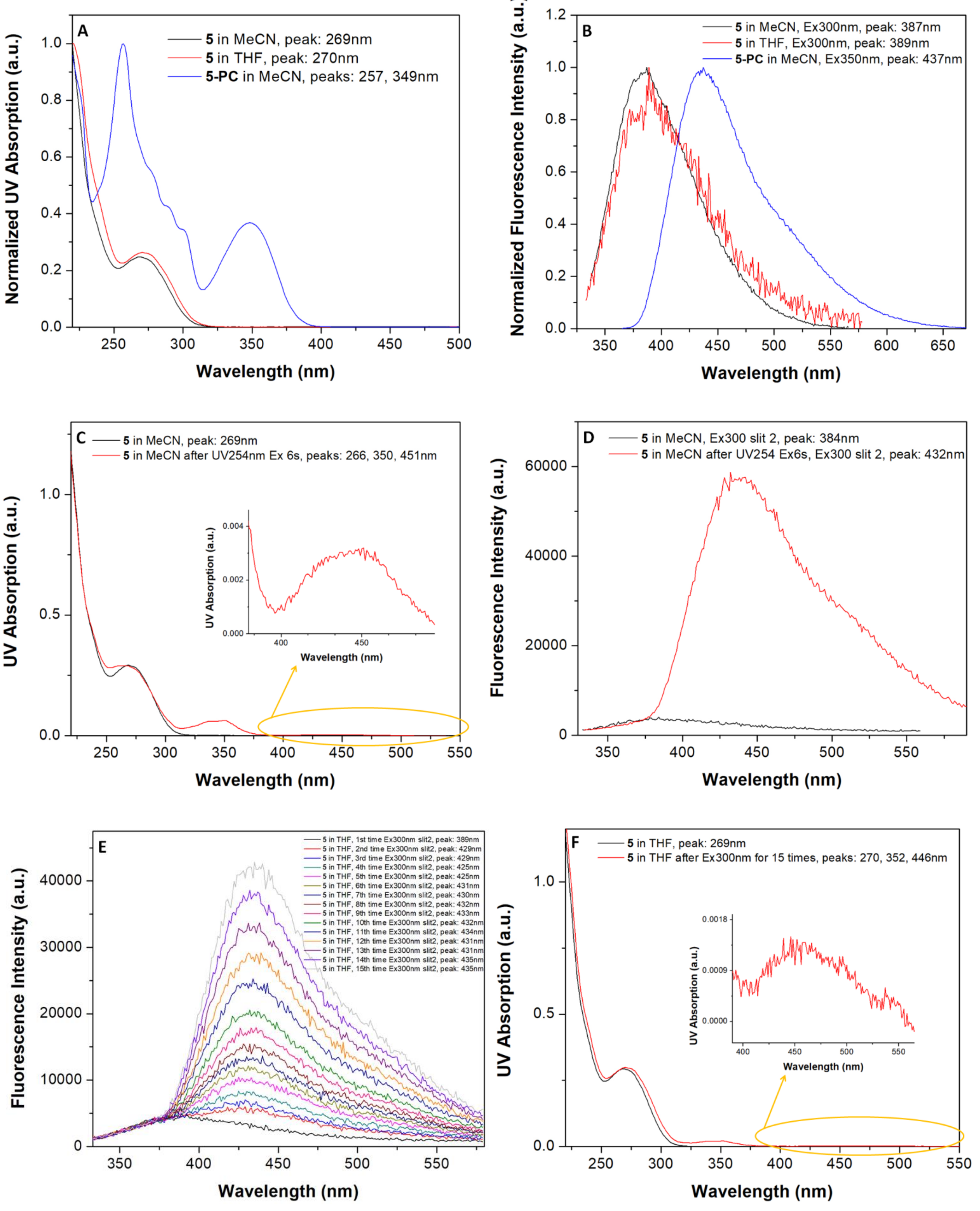

Figure S32. (A) UV-Vis spectra of **5** and **5-PC** in solution; (B) PL spectra of **5** and **5-PC** in solution (**5** in MeCN, slit5; **5** in THF, slit2; **5-PC** in THF, slit1); (C) UV-Vis spectra of **5** in MeCN after irradiating the sample at 254nm for 6s using a hand-held UV lamp; (D) PL spectra of **5** in MeCN after irradiating

the sample at 254nm for 6s using a hand-held UV lamp; (E) PL spectra of **5** in THF (1.07×10-5 mol/L) before and after 15 times excitation at 300 nm (slit 2) in the PL fluorimeter; (F) UV-Vis spectra of **5** in THF (1.07×10-5 mol/L) before and after 15 times excitation at 300 nm in the PL fluorimeter.

The compound **5** under UV excitation is very easy to form the photocyclized product **5-PC**, as it can be evidenced by the emergence of new emission peak at 429 nm after the 2nd time excitation at 300nm (Figure S32. E, B). During the process of **5** under the UV excitation, new UV absorption peaks at 350 nm and 451 nm emerge, which are attributed to the absorption of **5-PC** and **5-IM**, respectively (Figure S32. A, C, F).

5.6 Photophysical properties of 6 and 6-PC

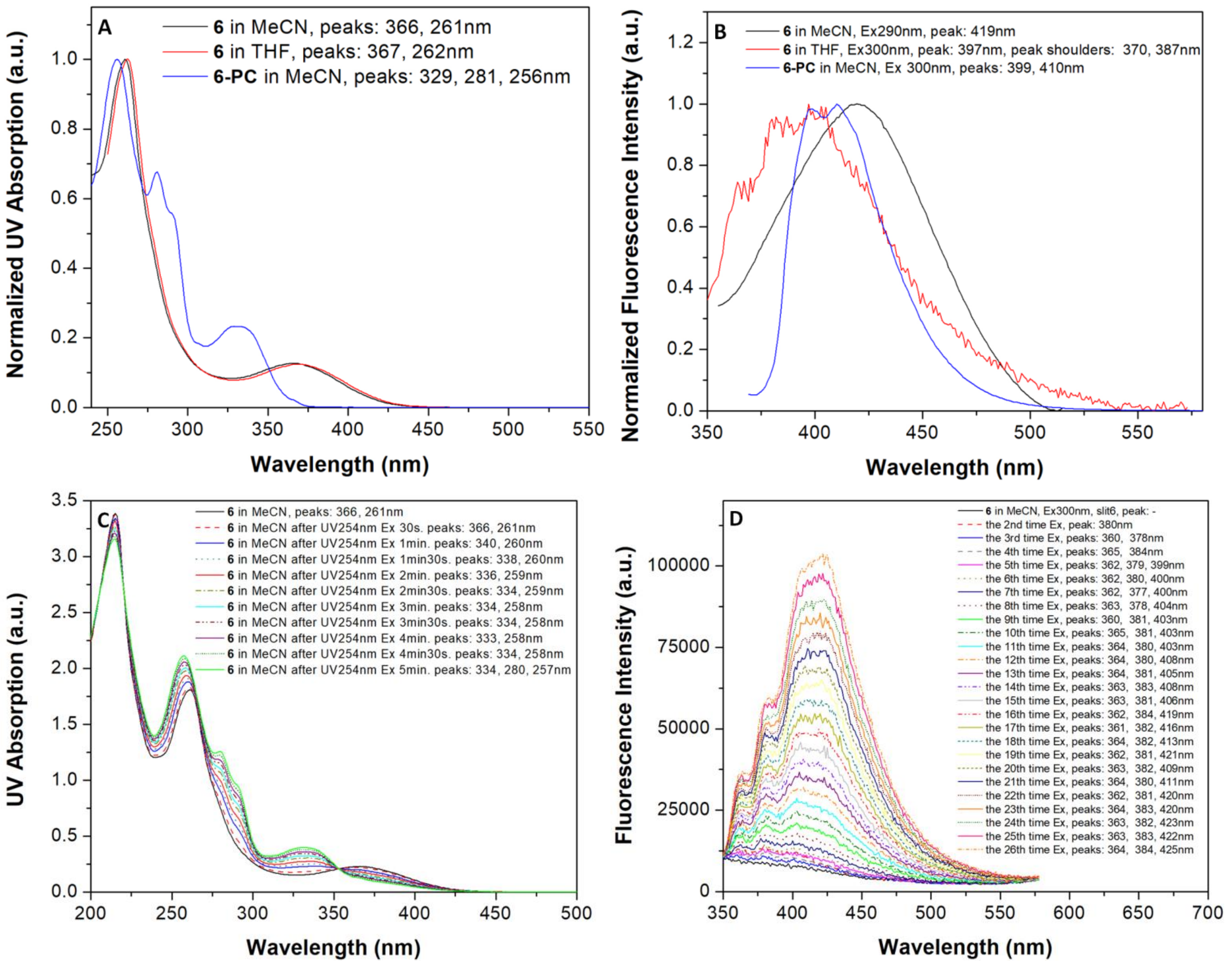

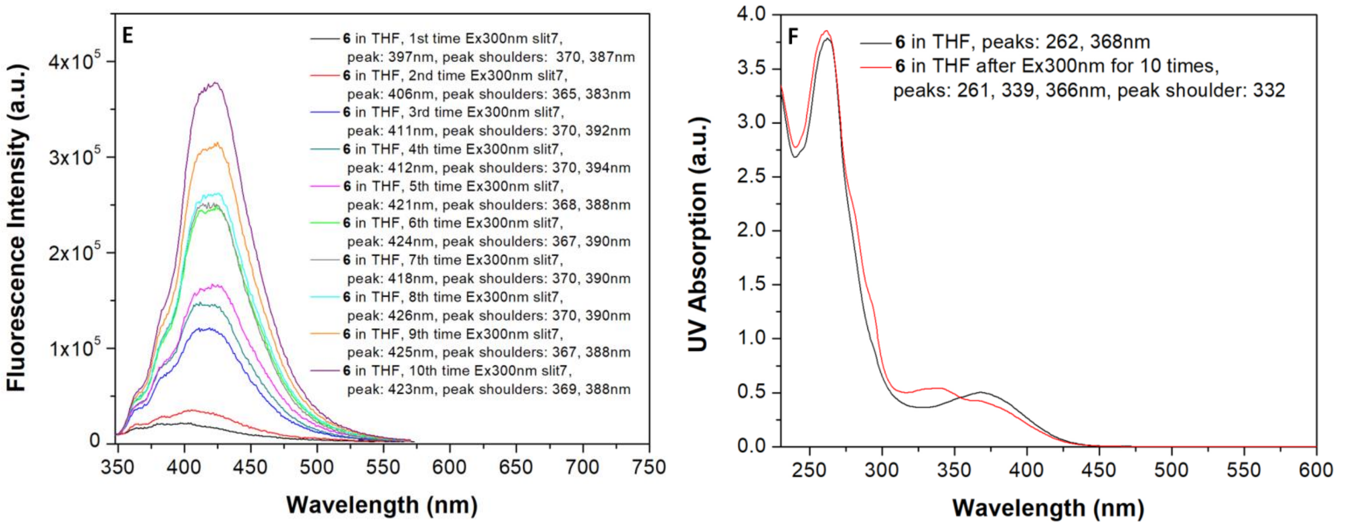

Figure S33. (A) UV-Vis spectra of **6** and **6-PC** in solution; (B) PL spectra of **6** and **6-PC** in solution; (C) UV-Vis spectra of **6** in MeCN after irradiating the sample 10 times (30s each time) at 254 nm using a hand-held UV lamp; The very pale yellow color of **6** in MeCN (or pale yellow color of **6** in THF) solution turns to *transparent* after irradiation at 254 nm and the photo-cyclized product **6-PC** is formed with a new peak at 280 nm emerging. (D) PL spectra of **6** in MeCN after 26 times excitation at 300 nm (slit: 6) acquired each 30s in the PL fluorimeter; (E) PL spectra of **6** in THF (2.60×10^{-5} mol/L) before and after 10 times excitation at 300 nm in the PL fluorimeter; (F) UV-Vis spectra of **6** in THF (2.60×10^{-5} mol/L) before and after 10 times excitation at 300 nm in the PL fluorimeter.

The pale-yellow solution of **6** in THF turns to transparent after UV light irradiation. While the fluorescence intensity of **6** in solution increases gradually after UV excitation, it undergoes photocyclization (Figure S33. C) as evidenced by the emergence of new absorption peaks at around 330 nm, 280 nm and 257 nm, which are rather similar to those absorption peaks in **6-PC**.

5.7 The absolute quantum yields and AIE properties of TPE derivatives

Table S2. The absolute quantum yields of TPE derivatives.

TPE derivatives	The absolute quantum yields (QY, %)	
	In soln (10^{-4} M)	As solid
**1** (Ex303nm)	0.8 [a]	24.1
**2** (Ex310nm)	0.6 [a]	30.2
**3** (Ex314nm)	60 [a]	97.6
**4** (Ex305nm)	1.0 [a]	1.3
**5** (Ex310nm)	0.9 [a]	16.5
**6** (Ex300nm)	0.5 [a]	0.7

[a] In THF solution.

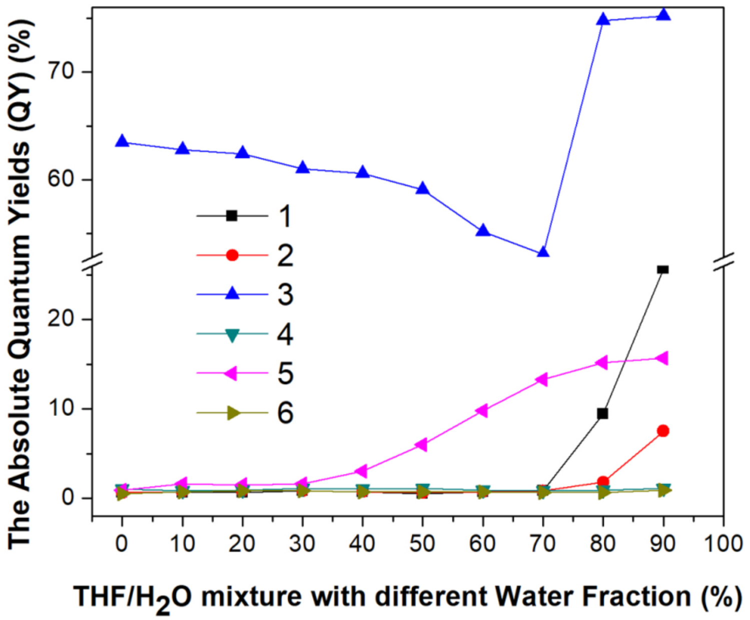

Figure S34. The absolute fluorescence quantum yields of **1-6** in THF/H_2O mixtures with different water fraction (Vol, %); the data for the specific points can be found in the Table S3.

Table S3. The absolute fluorescence quantum yields of TPE derivatives in THF/H_2O mixtures (10^{-5} M) with different fractions of H_2O content.

Water Fraction (Vol, %)	The absolute quantum yields (QY, %)					
	**1** (Ex308nm)	**2** (Ex310nm)	**3** (Ex314nm)	**4** (Ex305nm)	**5** (Ex310nm)	**6** (Ex300nm)
0	**0.6**	**0.6**	**63.5**	**1.0**	**0.9**	**0.5**
10	0.6	0.7	62.8	0.8	1.6	0.7
20	0.6	0.8	62.4	0.9	1.5	0.8
30	0.8	0.8	61.0	1.1	1.6	0.8
40	0.7	0.7	60.6	1.0	**3.0**	0.7
50	0.5	0.6	59.1	1.1	6.0	0.7
60	0.7	0.7	55.2	0.9	9.8	0.7
70	0.8	0.8	53.1	0.8	13.3	0.6
80	**9.4**	**1.8**	74.8	0.9	15.2	0.6
90	**25.7**	**7.5**	**75.2**	**1.1**	**15.7**	**0.9**

6 Ultrafast time-resolved spectroscopy

Several experimental studies of the excited-state dynamics in **1** upon excitation using ultrafast time-resolved spectroscopy have been reported. [21-25] Based on the findings of these reports, formation of zwitterionic state, [21-22] twisted radical species, [23] as well as significantly elongated [24] and twisted [26-27] excited states of **1** after excitation have been proposed. However, the interpretation of the reported experimental data across the relevant literature is highly inconsistent, making it less conclusive than desirable. In this paper, we employed femtosecond transient absorption spectroscopy (fs-TA), ns-TA, ns-TR^2, ns-TR^3 and time-resolved fluorescence in order to gain deep insight into the fundamental photophysical and photochemical processes taking place in the selected TPE derivatives **1-6** upon photoexcitation, and provide an interpretation of the obtained experimental data in the context of our AIE mechanism studies.

A. Femtosecond Transient Absorption (fs-TA) Experiment.

The fs-TA experiments were done employing an experimental setup and methods detailed previously [28] and only a brief description is provided here. Fs-TA measurements were done using a 1000Hz femtosecond regenerative amplified Ti:sapphire laser system (Maitai) in which the amplifier was seeded with the 120 fs laser pulses from an oscillator laser system. The laser probe pulse was produced by utilizing ~5% of the amplified 800 nm laser pulses to generate a white-light continuum (350-800 nm) in a CaF_2 crystal and then this probe beam was split into two parts before traversing the sample. One probe laser beam goes through the sample while the other probe laser beam goes to the reference spectrometer in order to monitor the fluctuations in the probe beam intensity. For the present experiments, the compounds **1-6** in MeCN solution were excited by a 267 nm pump beam (the third harmonic of the fundamental 800 nm from the regenerative amplifier). The 40 mL solutions were studied in a flowing 2 mm path-length cuvette with an absorbance of 0.5 at 267 nm throughout the data acquisition. And the absorbance of **1-6** in films was also around 0.5 at 267 nm throughout the data acquisition.

B. Nanosecond Transient Absorption (ns-TA) Experiment.

Nanosecond time-resolved transient absorption (ns-TA) measurements were carried out with a LP920 laser flash spectrometer provided by Edinburgh Instruments Ltd. The probe light source is a 450 W ozone free Xe arc lamp with 10 Hz to single shot operation versatile sample chamber with integral controller, high speed pump and probe port shutters, sample holder, filter holders, which produces a continuous spectrum between 280 to 600 nm. The pulse duration for the ns-TA measurements is 5 ns. Measurements of the ns-TA spectra were performed according to the following procedure. The fresh sample solutions were excited by a Q-switched Nd:YAG laser (4th harmonic line at λ=266 nm). The probe light from a pulsed xenon arc lamp was passed through various optical elements, samples, and a monochromator before being detected by a fast photomultiplier tube and recorded with a TDS 3012C digital signal analyzer. In the kinetics mode, a photomultiplier detector or InGaAs PIN detector is used and the transient signal acquired using a fast, high resolution oscilloscope. In the spectral mode an array detector is fitted to the spectrograph exit port to measure a full range of wavelengths simultaneously. Unless specified otherwise, the ns-TA experiments were performed in air saturated solutions and the compounds **1-6** in MeCN solution were made up to have an absorbance of 0.5 at 266 nm.

C. Nanosecond transient resonance Raman (ns-TR^2) and time-resolved resonance Raman (ns-TR^3) Experiments.

The ns-TR^2 spectrum was obtained from the difference of the spectra under 266 nm high power and low power of the pump laser by taking an appropriately scaled difference spectrum to subtract the precursor and solvent Raman bands. The nanosecond time-resolved resonance Raman (ns-TR^3) experiments were done using an experimental apparatus and methods discussed in detail previously, [29] and only a short description will be given here. The pump laser pulse with a wavelength of 266 nm generated from the fourth harmonic of a Nd:YAG nanosecond pulsed laser, a 355 nm probe laser pulse produced from the third harmonic of a Nd:YAG nanosecond pulsed laser and a 309.1 nm probe laser pulse produced from the first anti-Stokes hydrogen Raman shifted laser line from the second (355 nm) harmonic were employed in the TR^3 experiments. The two Nd:YAG lasers were synchronized electronically by a pulse delay generator to control the time delay of pump and probe lasers, and the time delay between the laser pulses was monitored by a fast photodiode and 500 MHz oscilloscope. The time resolution for the TR^3 experiments is approximately 10 ns. The pump and probe laser beams were lightly focused onto the sampling system, and the Raman light was collected using reflective optics into a spectrometer whose grating dispersed the light onto a liquid nitrogen cooled CCD detector. The Raman signal was acquired for 10-30 s by the CCD before reading out in the interfaced PC computer, and 10-30 scans of the signal were accumulated to produce a resonance Raman spectrum. The TR^3 spectra presented here were ob-

tained by the subtraction of a resonance Raman spectrum with negative time delay of -100 ns (probe-before-pump spectrum) from the resonance Raman spectrum with a positive time delay (pump-probe spectrum). The TR^3 spectra in this work were calibrated by using known MeCN solvent's Raman bands with an estimated accuracy of (5 cm^{-1}). Compounds **1**, **2**, **4**, **5** and **6** in MeCN solution were prepared to have an UV absorption 1~2 at 266 nm in a 1 mm path-length cuvette and then were used in the TR^2 and TR^3 experiments.

D. Femtosecond time-resolved Fluorescence (fs-TRF)

Femtosecond time-resolved fluorescence (fs-TRF) measurements [30] were performed on the same setup as fs-TA. The output 800 nm laser pulse (200 mw) is used as gate pulse while the 400 nm laser pulse (10 mw) (second harmonic) is used as the pump laser. After excitation by the pump laser, the sample fluorescence is focused into the nonlinear crystal (BBO) mixing with the gate pulse to generate the sum frequency signal. Broadband fluorescence spectra are obtained by changing the crystal angles and the spectra are detected by the air-cooled CCD. For the present experiments, the compound **3** in MeCN solution were excited by a 267 nm pump beam (the third harmonic of the fundamental 800 nm from the regenerative amplifier). The 40 mL solutions were studied in a flowing 2 mm path-length cuvette with an absorbance of 0.5 at 267 nm throughout the data acquisition.

6.1 Ultrafast time-resolved spectroscopy of TPE derivatives in solution

The optimized structures of the photocyclized intermediates in acetonitrile for UV/Vis and Raman spectra calculation are shown in the **Supporting Information section 7.4.1**. These are calculated at M062X/6-311G (d) level [31-32] using the Gaussian 09 package. [33] The mechanism of the formation and oxidization of similar photocyclized intermediates during the photocyclization process have been well proposed in the literature. [34-40]

6.1.1 fs-TA and ns-TR^2 spectra of 1

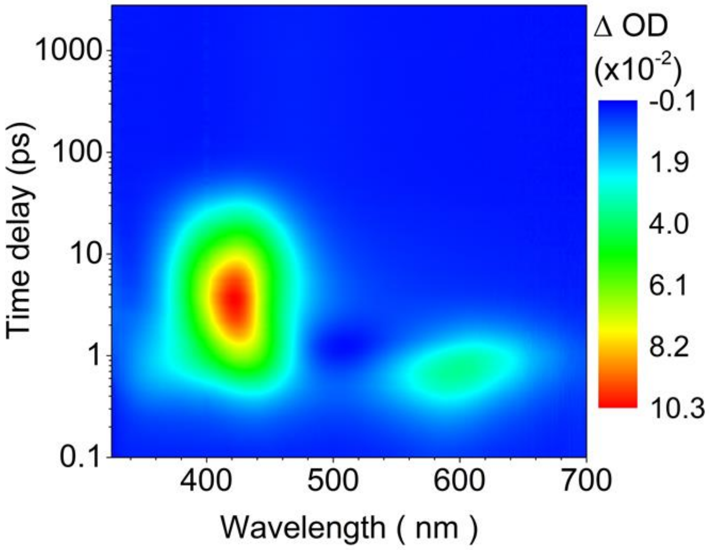

Figure S35. Contour plot of the time-resolved absorption spectroscopic responses of **1** in MeCN.

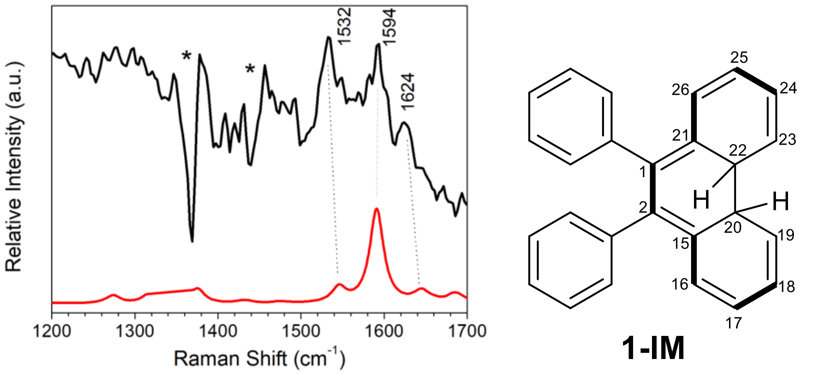

Figure S36. The TR^2 data of **1** acquired in MeCN solution upon excitation after 5-10 ns (black curve; obtained using a difference of the high and low power 266 nm resonance Raman spectra) is compared to the computed Raman spectrum of **1-IM** in MeCN (red curve). The stars represent the bands caused by solvent subtraction artifacts.

Femtosecond transient absorption (fs-TA) experiments were used to study the photoinduced cyclization processes of **1** in MeCN solution. As shown in Figure 2a, the S_1 exited state of **1** shows up at 0.6 ps with two absorption bands at 430 nm and 600 nm. Spontaneously, the band at 430 nm grows from 0.6 ps to 1.3 ps while the band at 600 nm decreases red-shifting to 613 nm within a few hundred of femtoseconds, which could be assigned to the ultrafast elongation of the ethylenic C=C bond. [23-24] After 1.3 ps, the transient band at 613 nm decreases with the sequential increasing of the band at 428 nm, which can be attributed to the quasi C=C bond twisting motion [23] coupled with phenyl torsion. At the later delay times, the band at 422 nm decays rapidly due to the torsion of the phenyl rings. Unlike the previous studies,[24, 27] a small but brand new species is observed after 105 ps with absorption bands at 465 nm. Therefore, an unexplored photoreaction pathway of **1** after the ethylenic twisting process can be proposed, which could be one of the non-radiative relaxation pathways for the ultralow fluorescence quantum yield observed in solution. Both Gao [41] and Aldred [2] mentioned that the cyclization process is favored for TPE derivatives in solution. The new species could be assigned to **1-IM**, which is formed during the decay process of torsion of the phenyl rings. Global analysis of the decay kinetics at all wavelengths indicates that a satisfactory fitting requires three exponential functions with time constants of 0.39 ps (τ_1), 1.2 ps (τ_2) and 18.9 ps (τ_3). The time constants 0.39 ps (τ_1) and 1.2 ps (τ_2) are corresponding to the C=C bond elongation and quasi C=C bond twisting processes, respectively. The time constant of 18.9 ps is assigned to the decay process of the phenyl torsion, *i.e.* the formation lifetime for the **1-IM** intermediate. Besides, the nanosecond transient absorption (ns-TA) and nanosecond time-resolved Raman (ns-TR^2) were also employed to further study the intermediate observed at later delay times in Figure 2e and Figure S36. As shown in Figure 2e, same spectra with absorption bands at 320 nm and 465 nm were obtained in ns-TA, to which the new species (**1-IM**) correspond can last more than 1 ms. This is consistent with photophysical property studies discussed above (Supporting Information Section 5 and Figure 2e, upper panel). In order to confirm the existence of the intermediates obtained both in the fs-TA and ns-TA results, TD-DFT calculation was employed to evaluate the electronic absorption spectra of **1-IM** as presented in Figure 2e (lower panel). The calculated absorption spectrum of **1-IM** displays two bands at 310 nm and 460 nm between 280-600 nm which is in good agreement with the experimental results. Therefore, the TD-DFT results provide further evidence for the generation of **1-IM**.

Figure S36 presents the 266 nm ns-TR^2 data compared to a computed DFT normal Raman spectrum for the intermediate **1-IM**. This comparison shows reasonable correlation for the vibrational frequency pattern of the calculated and experimental Raman bands. Although their intensities are different due to the resonance enhancement effects on the experimental data, the computed data does not have this effect. The vibrational feature at 1624 cm^{-1} is mostly attributed to the stretching motion of C_1=C_{21}, C_2=C_{15},

$C_{16}=C_{17}$, $C_{18}=C_{19}$, $C_{23}=C_{24}$ and $C_{25}=C_{26}$ bonds. The feature at 1594 cm^{-1} is mainly correlated with the stretching mode of $C_1=C_{21}$, $C_2=C_{15}$, C_1-C_2, C_{21}-C_{22}, C_{15}-C_{20}, C_{15}-C_{16}, C_{21}-C_{26}, $C_{16}=C_{17}$, $C_{18}=C_{19}$, $C_{23}=C_{24}$ and $C_{25}=C_{26}$ bonds. And the 1532 cm^{-1} feature is mainly ascribed to the stretching motion of $C_1=C_{21}$, $C_2=C_{15}$ and C_1-C_2 bonds.

6.1.2 fs-TA, ns-TA and ns-TR^2 spectra of 2

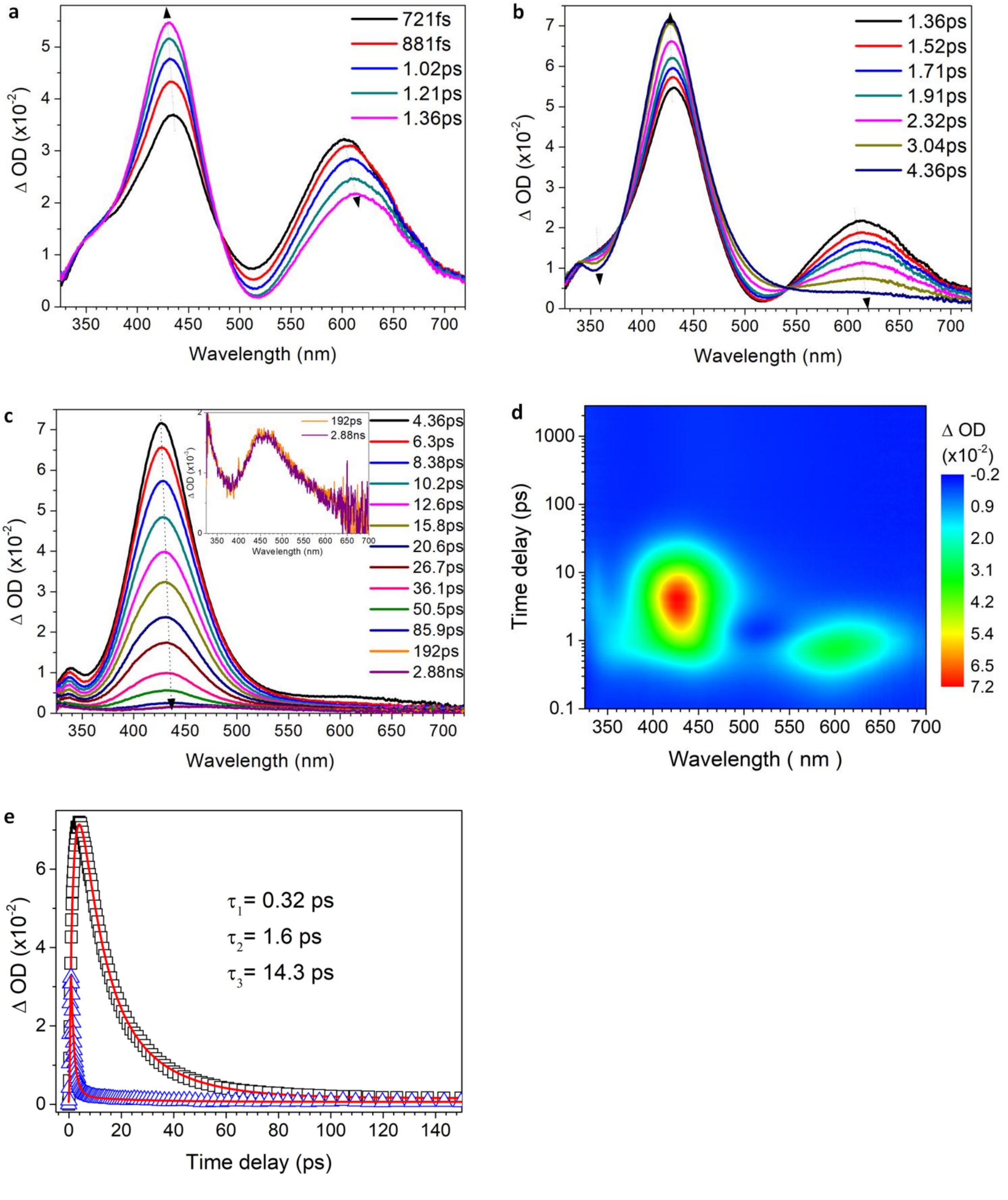

Figure S37. fs-TA of **2** in MeCN solution acquired after 267 nm irradiation: (a) contour plots of the

spectroscopic responses; (b-d) selected spectra at different delay times; (e) selected kinetics at 430 nm (black square) and 602 nm (blue triangle), the solid lines indicate the fitting trace to the experimental data points.

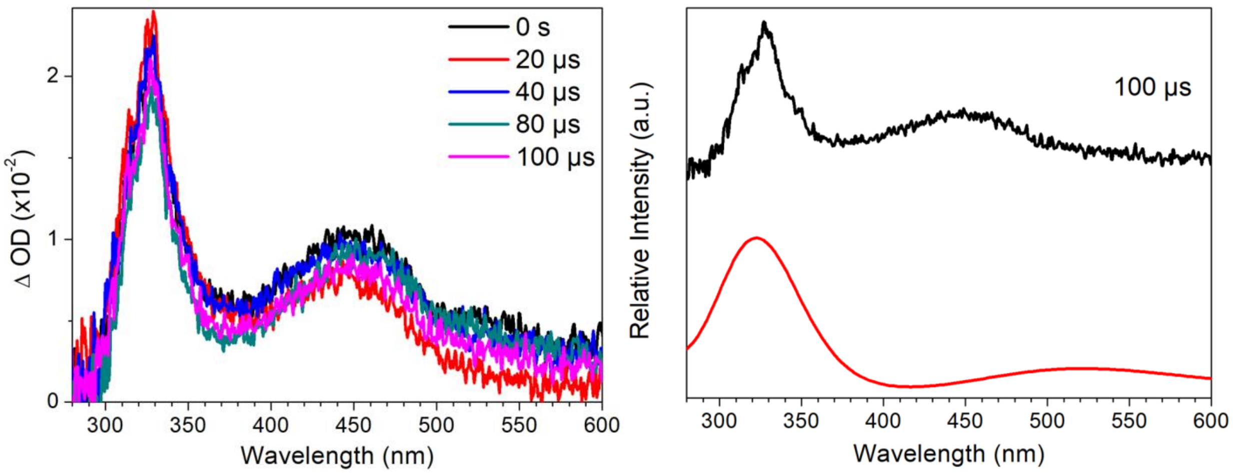

Figure S38. ns-TA of **2** in MeCN solution acquired after 266 nm irradiation: (Left) selected spectra at different delay times; (Right) comparison of the spectrum at 100 μs (top) with the calculated electronic spectrum of **2-IM** (bottom).

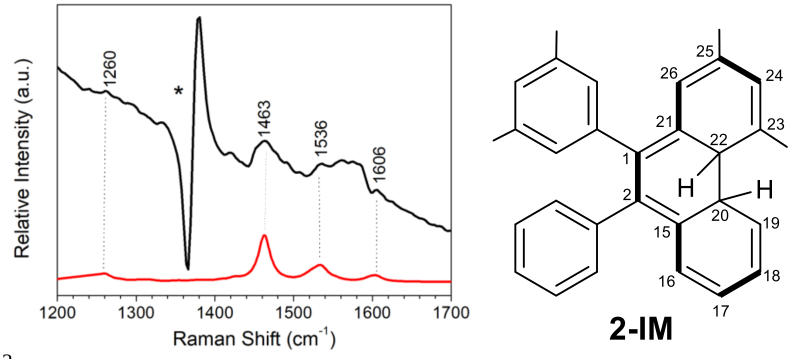

Figure S39. The TR^2 data of **2** acquired in MeCN solution upon excitation after 5-10 ns (black curve; obtained using a difference of the high and low power 266 nm resonance Raman spectra) is compared to the computed Raman spectrum of **2-IM** (red curve). The stars represent the bands caused by solvent subtraction artifacts.

The fs-TA, ns-TA and ns-TR^2 results were obtained for the compound **2** in Figure S37, Figure S38 and Figure S39. Compound **2** has similar excited state dynamic process as **1** discussed above. The global fitting results show that the time constants for the sequential dominant processes of C=C bond elongation, quasi C=C bond twisting and cyclization are 0.32 ps, 1.6 ps and 14.3 ps, respectively.

As shown in Figure S38 (Left), similar spectra with absorption bands at 330 nm and 450 nm as observed at 2.88 ns in fs-TA were obtained in ns-TA experiment, which can be attributed to the photocyclized intermediate that last more than 100 μs. In order to confirm the existence of the intermediates obtained both in the fs-TA and ns-TA results, TD-DFT calculation was employed to evaluate the electronic absorption spectra of **2-IM** as presented in Figure S38 (Right). The calculated absorption spectrum of **2-IM** displays two bands at 323 nm and 520 nm between 280-600 nm, which is in good agreement with the experimental results.

In ns-TR^2 spectra (Figure S39), the vibrational feature at 1606 cm^{-1} and 1536 cm^{-1} is mostly attributed to the stretching motion of C_1-C_2, C_1=C_{21}, C_2=C_{15}, C_{21}-C_{26}, C_{15}-C_{16}, C_{21}-C_{22} and C_{15}-C_{20} bonds. The feature at 1463 cm^{-1} is mainly correlated with the stretching mode of C_{21}-C_{26}, C_{24}-C_{25}, C_{21}-C_{22} and C_{22}-C_{23} bonds. And the 1260 cm^{-1} feature is mainly ascribed to the rocking and wagging modes of C_{22}-H and C_{20}-H bonds, and slight stretching mode of C_1-C_2, C_{21}-C_{26} and C_{15}-C_{16} bonds.

6.1.3 fs-TA spectra and time-resolved fluorescence spectra of 3

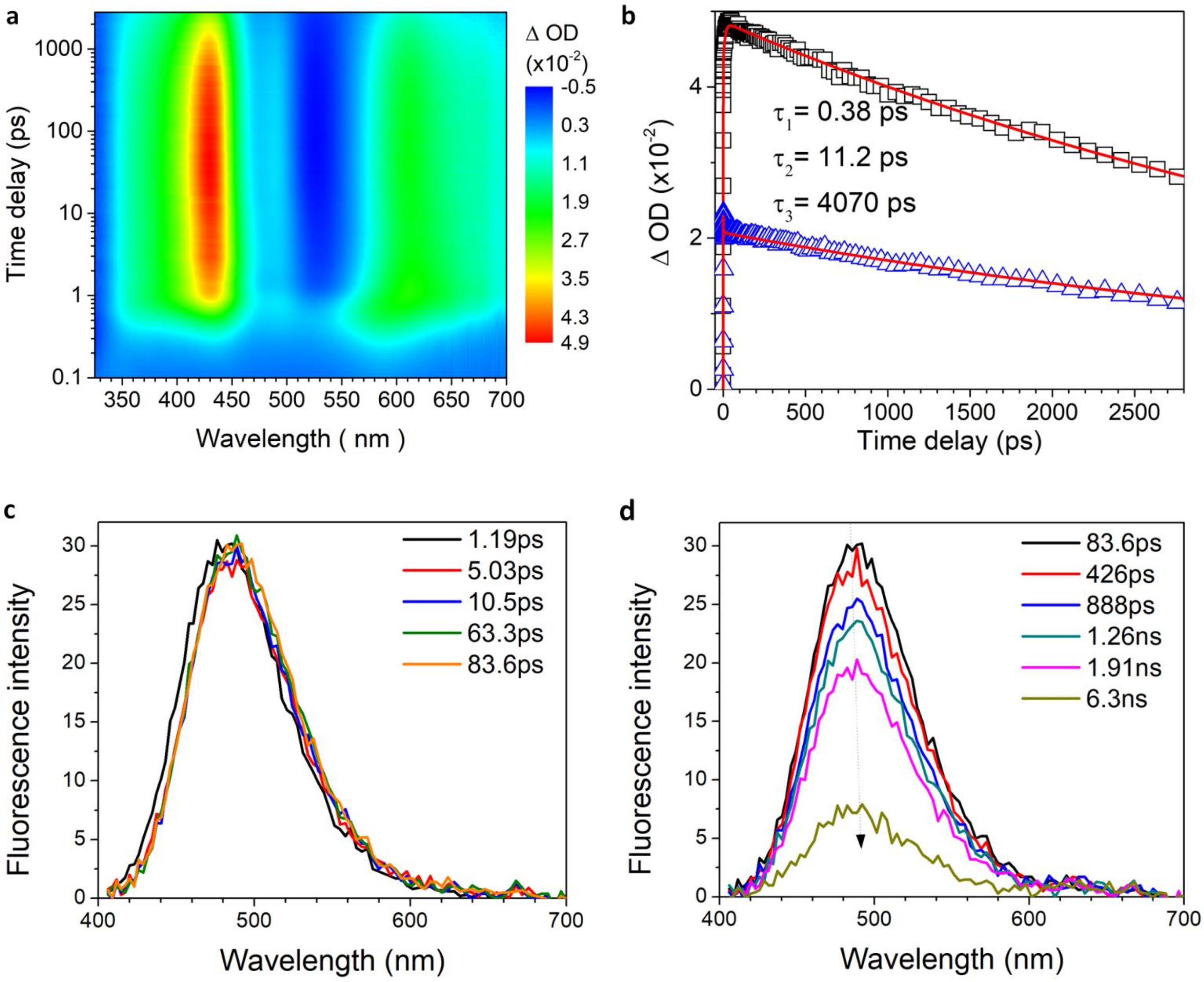

Figure S40. fs-TA of **3** in MeCN solution acquired after 267 nm irradiation: (a) contour plots of the spectroscopic responses; (b) selected kinetics at 430 nm (black square) and 610 nm (blue triangle), the solid lines indicate the fitting trace to the experimental data points; (c-d) Time-resolved fluorescence (TRF) spectra of **3** from 1.19 ps to 6.3 ns in MeCN after 267 nm excitation.

The initial fs-TA spectra of **3** in Figure 3a are similar to the ones shown for **1** in Figure 2a. As displayed in Figure 3a, the increased band at 432 nm with the red-shifted band at 600 nm can be assigned to the elongation of C=C bond process in **3** in the S_1. However, compared with the spectra in Figure 2b for **1**, the continuous changes of the ultrafast spectra in Figure 3b is much smaller: the bands at 610 nm decreases a little, while the band at 430 nm increases a bit, which indicate that the quasi C=C bond

twisting process might be inhibited. Besides, a small dip at 477 nm is observed both in Figure 3a and Figure 3b, which is due to the stimulated fluorescence in the S_1 excited state.

In Figure 3c, only the decay of the bands was obtained at later delay times. This is consistent with Gao's TD-DFT calculations [41] that predict that both the S_1 photocyclization and photoisomerization paths are blocked by the non-negligible barriers, and fluorescence is thus feasible in solution. Global analysis of the decay kinetics at all wavelengths indicates that a satisfactory fitting requires three exponential functions with time constants of 0.38 ps (τ_1), 11.2 ps (τ_2) and 4.07 ns (τ_3). The time constants 0.38 ps (τ_1) and 11.2 ps (τ_2) are assigned to the C=C elongation and quasi C=C bond twisting processes, respectively. Compared to the C=C bond twisting process of **1** (1.2 ps), the one observed in **3** is much slower. The time constant of 4.07 ns corresponds to the decay of the twisted S_1 excited state of **3**.

As shown in Figure 3d (upper panel), the absorption bands at 353 nm and 460 nm were obtained after 1 μs in ns-TA spectra, which indicates that the intermediate **3-IM** can also be formed, but at later delay time. In order to confirm the existence of the intermediate obtained by ns-TA, TD-DFT calculation was employed to evaluate the electronic absorption spectrum of **3-IM** as presented in Figure 3d (lower panel). The calculated absorption spectrum of **3-IM** displays two bands at 305 nm and 445 nm between 280-600 nm, which is in good agreement with the experimental results. Therefore, the TD-DFT results provide further evidence for the generation of **3-IM**.

6.1.4 ns-TR^3 spectra of 4

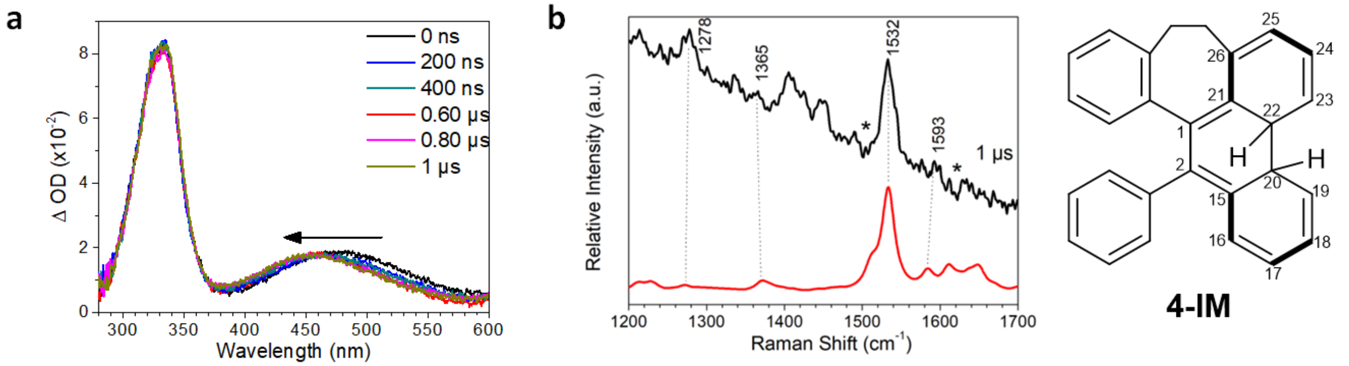

Figure S41. a, Ns-TA spectra of **4** in MeCN at varying time delays acquired after a nanosecond laser excitation at 266 nm. **b**, The experimental TR^3 spectrum (at 1 μs) of **4** observed in MeCN (black curve; 266 nm pump, 355 nm probe) compared to DFT computed Raman frequencies of **4-IM** (red curve).

As shown in Figure 4a, the initial spectrum of **4** after excitation yields three absorption bands at 350nm, 450 nm and 620 nm, respectively, in which the band at 620 nm redshifts to 630 nm from 800 fs to 1.1 ps, which is attributed to the elongation of the ethylenic C=C bond in the S_1 excited state. After 1.1 ps, the band at 630 nm decreases dramatically, while the band at 444 nm increases noticeably with a blue shift to 430 nm as shown in Figure 4b. This is associated with the twisting process of the quasi C=C bond coupled with phenyl torsion. Later, band at 430 nm decays after 3.8 ps giving rise to the bands at 335 nm and 485 nm, which are due to the cyclization process initiated by the torsion of the phenyl rings to form the ring-closed intermediate **4-IM**. Global analysis of the decay kinetics at all wavelengths indicates that a satisfactory fitting requires three exponential functions with time constants of 0.13 ps (τ_1), 1.2 ps (τ_2) and 14.0 ps (τ_3), which can be assigned to the ethylenic C=C bond elongation, quasi C=C bond twisting and cyclization (*i.e.* lifetime of the decay process of phenyl torsion) processes, respectively.

The ns-TA spectra of **4** are displayed in Figure 4f (upper panel) and similar long-lived **4-IM** species was observed. But unlike the ns-TA of **1**, the broad band at 485 nm blue shifts to 450 nm from 0 ns to 25 μs which could be due to the geometry changes upon relaxation of the compound **4-IM** in the ground

state from higher energy to lower energy (Figure S63 and Figure 4f, upper panel). At the late delay time (25-100 μs), few changes are observed. The comparison of the ns-TA spectra at 100 μs and TD-DFT calculated electronic spectrum of **4-IM** in Figure 4f (lower panel, computed UV peaks: 330 nm and 440 nm) indicates the generation of intermediate **4-IM** as proposed above.

Furthermore, ns-TR^3 experiments were employed using a 266 nm pump and 355 nm probe to explore finger print information for the intermediates involved after excitation of **4** in MeCN. Figure S41 presents TR^3 data compared to a DFT computed Raman spectrum for the intermediate **4-IM**. This comparison shows reasonable correlation for the vibrational frequency pattern between the calculated and experimental Raman bands. The vibrational feature at 1593 cm^{-1} is mostly attributed to the stretching motion of C_1=C_{21}, C_2=C_{15}, C_{23}=C_{24}, C_{25}=C_{26}, C_{18}=C_{19} and C_{16}=C_{17} bonds. The feature at 1532 cm^{-1} is mainly correlated with the stretching mode of C_1=C_{21} and C_1-C_2 bonds. And the feature at 1365 cm^{-1} and 1278 cm^{-1} is mainly ascribed to the C_{22}-H and C_{20}-H bonds' rocking and wagging modes.

6.1.5 ns-TA and ns-TR^3 spectra of 5

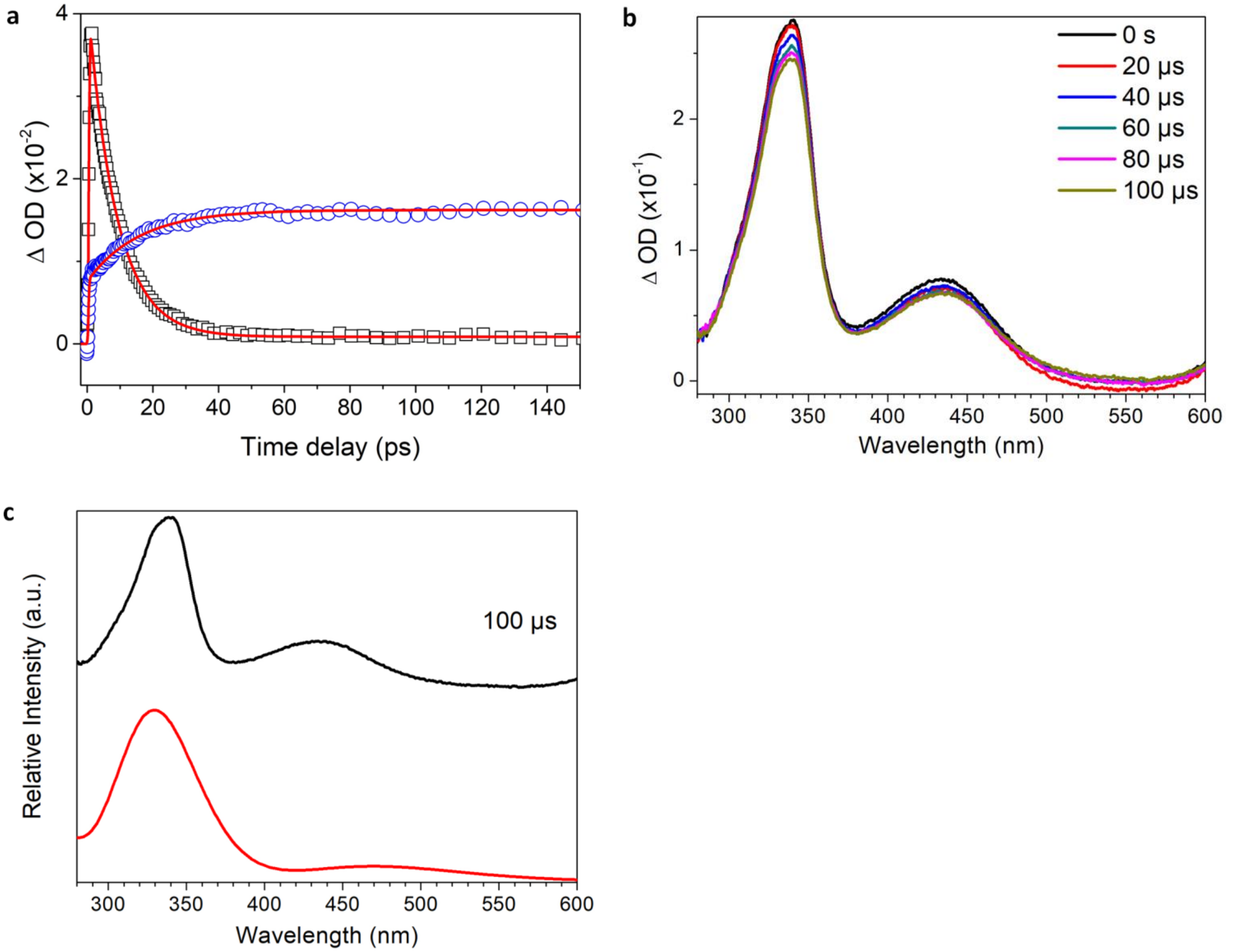

Figure S42. (a) selected kinetics at 626 nm and 340 nm of fs-TA, the solid lines indicate the fitting trace to the experimental data points; The global fitting analysis of the decay kinetics at all wavelengths yields two time constants: 0.3 ps and 8.9 ps. (b) ns-TA of **5** in MeCN solution acquired after 266 nm irradiation: selected spectra at different delay times; (c) comparison of the spectrum at 100 μs (top) with the calculated electronic spectrum of **5-IM** (bottom).

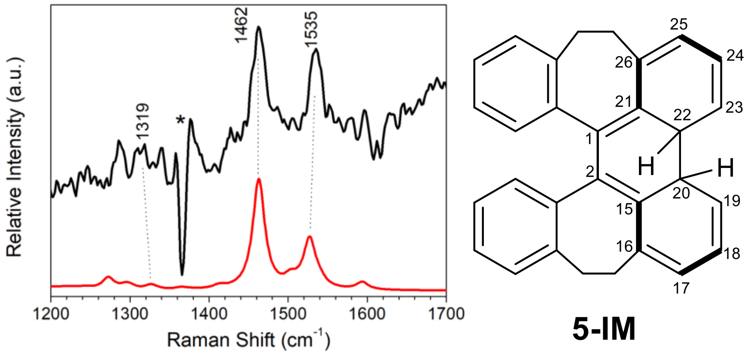

Figure S43. The experimental TR[3] spectrum (at 1 μs) of **5** observed in MeCN (black curve; 266 nm pump, 309.1 nm probe) compared to DFT computed Raman frequencies of **5-IM** (red curve).

The fs-TA spectra of **5** after 267 nm excitation are displayed in Figure 5a. Similar initial spectra as **4** were obtained for **5** with three absorption bands at 330 nm, 460 nm and 628 nm red shifting before 1.03 ps, which are mainly due to the ethylenic C=C bond elongation process in the S_1 excited state of **5** as discussed above. However, after 1 ps, both bands at 462 nm and 630 nm decrease, while the band at 330 nm increases with an emerging broad band at 435 nm, which indicates that the cyclization process to generate **5-IM** takes place shortly after the process of ethylenic bond elongation in S_1 of **5.** During this process, the quasi C=C bond twisting takes place spontaneously with the decay of the phenyl torsion to form the intermediate. Global analysis of the decay kinetics at all wavelengths indicates that a satisfactory fitting requires two exponential functions with time constants of 0.30 ps and 8.9 ps, which could be assigned to the elongation of ethylenic C=C bond and cyclization processes (*i.e.* the formation of cyclized intermediate **5-IM**).

Therefore, both the ns-TA and ns-TR[3] were employed to prove the existence of the intermediate in Figure S42 and Figure S43. The good agreement between the experimental results and calculation results indicates that the intermediate obtained at later delay times in fs-TA, ns-TA and TR[3] experiments can be assigned to **5-IM**. The vibrational feature at 1535 cm^{-1} is mostly attributed to the stretching motion of C_1-C_2, C_1=C_{21}, C_2=C_{15}, C_{21}-C_{26} and C_{15}-C_{16} bonds. The feature at 1462 cm^{-1} is mainly correlated with the stretching mode of C_{21}-C_{26}, C_{24}-C_{25}, C_{22}-C_{23}, C_{21}-C_{22}, C_{22}-C_{20}, C_{15}-C_{20}, C_{20}-C_{19}, C_{17}-C_{18} and C_{15}-C_{16} bonds. And the 1319 cm^{-1} feature is mainly ascribed to the rocking and wagging modes of C_{22}-H and C_{20}-H bonds, and the stretching mode of C_{22}-C_{20} and C_1-C_2 bonds.

6.1.6 EADS spectra of 6

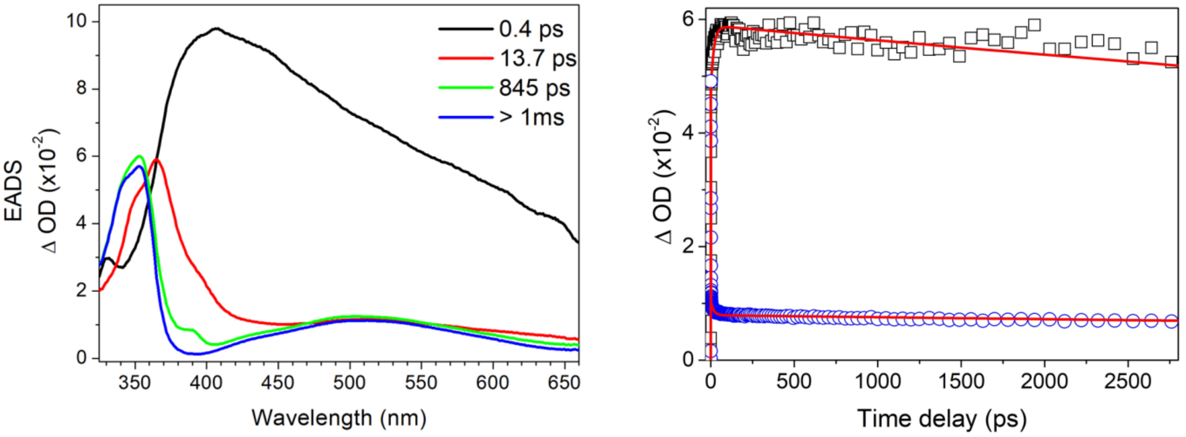

Figure S44. EADS spectra of **6** derived from the global fitting analysis (left); selected kinetics at 349 nm (black square) and 446 nm (blue circle) of fs-TA, the solid lines indicate the fitting trace to the experimental data points (right). The global fitting analysis of the decay kinetics at all wavelengths yields three time constants: 0.41 ps, 13.7 ps and 845 ps.

The fs-TA spectra of **6** after excitation of 267 nm are shown in Figure 5c-5e. Unlike the other compounds, the initial absorption band (after 700 fs) of **6** yields only one peak centered at 375 nm with a broad tail up to 650 nm in Figure 5c. Later, the band decreases a little to have a hypsochromic shift to 370 nm up to 4.7 ps, while the tail decays rapidly as shown in Figure 5c. After 4.7 ps, the band at 370 nm keeps hypsochromically shifting to 350 nm but with the intensity increase as shown in Figure 5d. Furthermore, the 370 nm band also displays an asymmetrical narrowing in which the right side of the peak narrows from 430 nm to 380 nm, which can be attributed to phenyl torsion during the cyclization process. And the tail keeps decreasing resulting in a broad band centered at 510 nm. Due to the slight difference between the continuous changes obtained in the fs-TA spectra, the resulting spectra can be analyzed by the global fitting with a sequential model to yield the evolution associated difference spectra (EADS) [42] as shown in Figure S44. And global analysis of the decay kinetics at all wavelengths indicates that a satisfactory fitting requires three exponential functions with time constants of 0.41 ps, 13.7 ps and 845 ps. The first EADS curve (black) indicates a new species emerges with a lifetime of 0.4 ps which could be due to the fast process of the quasi C=C bond twisting and phenyl torsion to form the intermediate **6-IM**. The second EADS curve (red) indicates a lifetime of 13.7 ps species which could be assigned to the relaxation process of phenyl torsion. The difference between the third EADS curve (green) and the fourth EADS curve (blue, infinite) is mainly about the small peak at 390 nm, which might indicates the relaxation process of the ground state **6-IM**, and this process (lifetime: 845ps) is much faster than **4-IM**.

Both the ns-TA and ns-TR^2 were employed to prove the existence of the **6-IM** as shown in Figure 5f. The good agreement between the experimental results and calculation results indicates that the intermediates obtained at later delay times in fs-TA, ns-TA and TR^2 experiments can be assigned to **6-IM**. The TR^2 spectra in Figure 5f (lower panel) of **6-IM** show that the vibrational features at 1738 cm^{-1} and 1641 cm^{-1} are mostly attributed to the stretching motion of C_1=C_{21}, C_2=C_{15}, C_{25}=C_{26}, C_{24}=C_{23}, C_{16}=C_{17}, C_{18}=C_{19} and C_{20}-C_{22}, C_1-C_2, C_{21}-C_{26}, C_{24}-C_{25}, C_{15}-C_{16}, C_{17}-C_{18}, C_{22}-C_{23}, C_{20}-C_{19} bonds. The vibrational feature at 1560 cm^{-1} is mainly correlated with the stretching mode of C_1-C_2, C_1=C_{21}, C_2=C_{15}, C_{15}-C_{20}, C_{15}-C_{16}, C_{21}-C_{22} and C_{21}-C_{26} bonds. And the vibrational features at 1304 cm^{-1} and 1259 cm^{-1} are mainly ascribed to the C_{22}-H and C_{20}-H rocking and wagging modes.

6.1.7 The lifetime of the photocyclized intermediates 1-IM - 6-IM

The decay of photocyclized intermediate's UV-Vis absorption spectra was obtained using a UV-Vis spectrophotometer (UH5300 HITACHI). The hand-held UV lamp equipped with 254nm 6 Watt UV tube (ENF-260C/FBE: BLE-6254S) was used to excite the dilute solutions of **1-6** in acetonitrile (10^{-4} M) in the experiment.

The solutions of **2**, **3** and **6** in MeCN (10^{-4} M) after UV lamp excitation at 254 nm for different periods of time have the UV-Vis absorption intensity of ~0.001 at 450 nm, ~0.004 at 460 nm and ~0.007 at 525 nm, respectively, which are too weak to analyze the lifetime of **2-IM**, **3-IM** and **6-IM**.

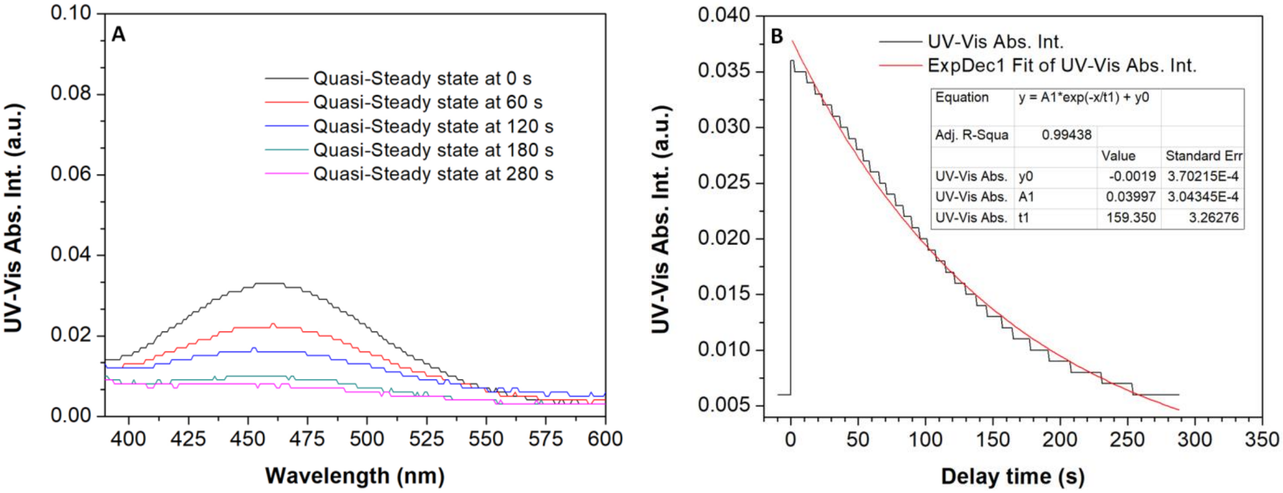

Figure S45. The lifetime of the photocyclized intermediate **1-IM**. (A) The UV-Vis absorption of **1-IM** at different delay time after the solution of **1** in MeCN (10^{-4} M) under the UV lamp excitation at 254 nm for 1min. (B) The monoexponential fitting of the decay of **1-IM**'s UV-Vis absorption at 465 nm.

The solution of **1** in MeCN (10^{-4} M) after UV lamp excitation at 254 nm for 1min have the maximum UV-Vis absorption intensity of 0.036 at 465 nm. The monoexponential fitting of the decay of **1-IM**'s UV-Vis absorption at 465 nm shows that **1-IM** has the lifetime of 159s.

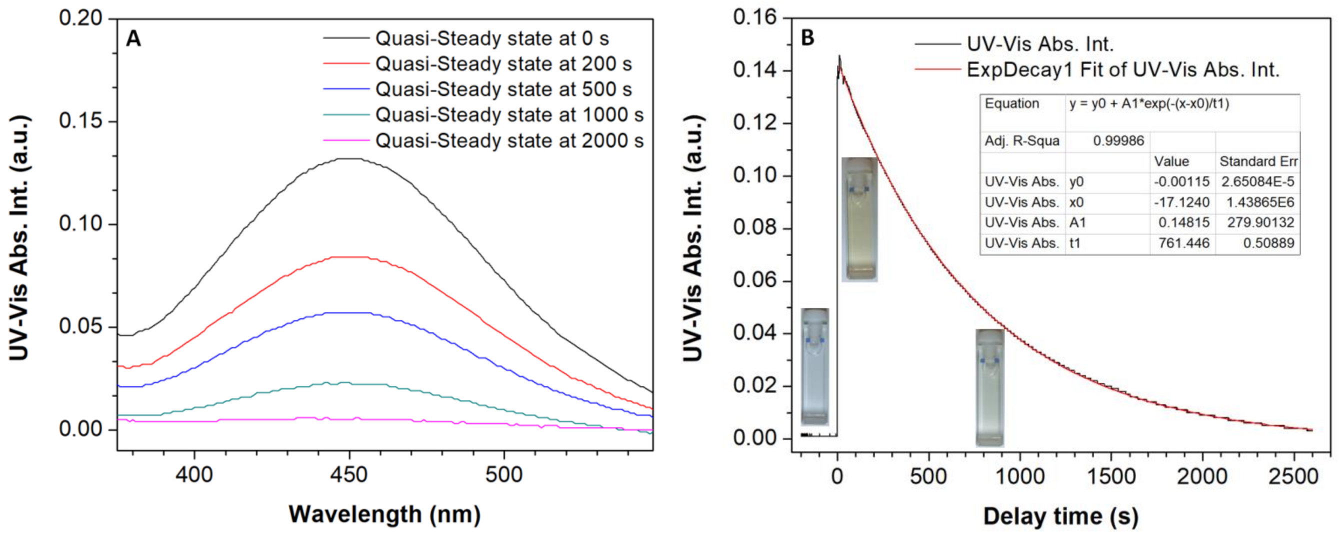

Figure S46. The lifetime of the photocyclized intermediate **4-IM**. (A) The UV-Vis absorption of **4-IM** at different delay time after the solution of **4** in MeCN (10^{-4} M) under the UV lamp excitation at 254 nm for 30s. (B) The monoexponential fitting of the decay of **4-IM**'s UV-Vis absorption at 450 nm; the photos of the solution before irradiation (left cuvette, colorless solution), after irradiating for 30s at the delay time of 0s (middle cuvette, yellow solution) and 760s (right cuvette, very pale yellow to colorless solution) are inserted.

The solution of **4** in MeCN (10^{-4} M) after UV lamp excitation at 254 nm for 30s have the maximum UV-Vis absorption intensity of 0.136 at 450 nm. The monoexponential fitting of the decay of **4-IM**'s UV-Vis absorption at 450 nm shows that **4-IM** has the lifetime of 761s.

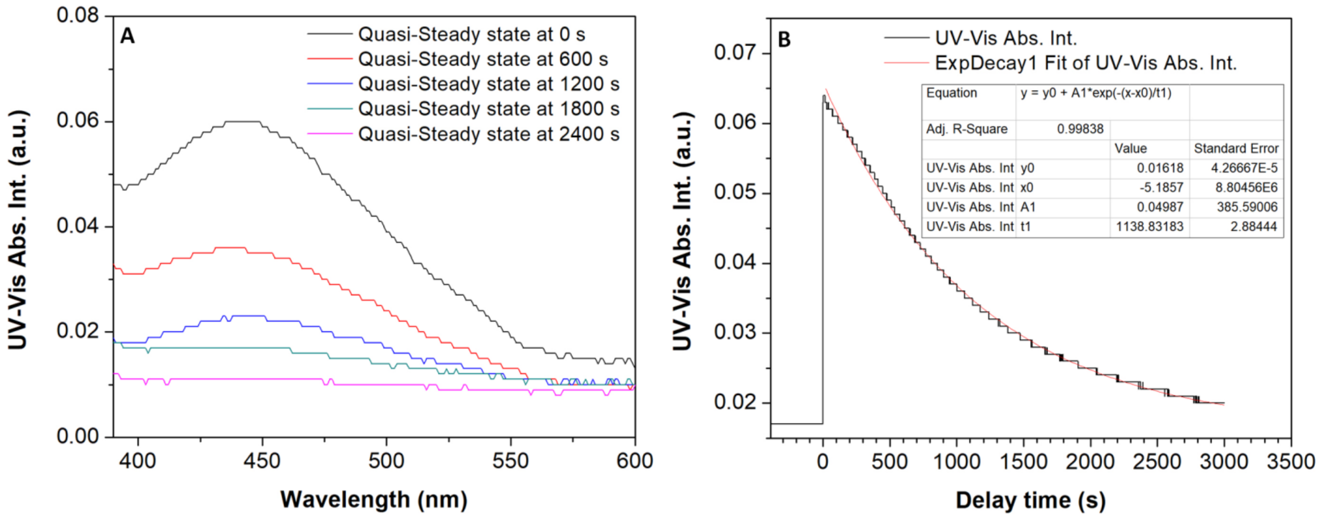

Figure S47. The lifetime of the photocyclized intermediate **5-IM**. (A) The UV-Vis absorption of **5-IM** at different delay time after the solution of **5** in MeCN (10^{-4} M) under the UV lamp excitation at 254 nm for 20s. (B) The monoexponential fitting of the decay of **5-IM**'s UV-Vis absorption at 438 nm.

The solution of **5** in MeCN (10^{-4} M) after UV lamp excitation at 254 nm for 20s have the maximum UV-Vis absorption intensity of 0.060 at 438 nm. The monoexponential fitting of the decay of **5-IM**'s UV-Vis absorption at 438 nm shows that **5-IM** has the lifetime of 1139s.

6.1.8 The excited state dynamic processes and time constants of 1-6

Table S4. The evolution of the peak wavelengths and intensities in the excited state dynamic processes of **1-6**. (Arrows' direction indicating the shift: arrows in blue indicating blue shift, arrows in red indicating red shift, arrows in black indicating no peak shift; Arrows' inclination indicating the change of the intensity of the peak.)

Dominant motions	Peak wavelengths (nm)											
	**1**				**2**				**3**			
Ethylenic C=C elongation		430 ↖		600 ↘		438 ↖		600 ↘		432 ↖		600 ↗
Quasi C=C twisting		428 ↖		613 ↓		432 ↖		615 ↘		430 ↖		610 ↓
Phenyl torsion		422 ↓				430 ↓				428 ↓		610 ↓

Dominant motions	Peak wavelengths (nm)											
	**4**				**5**				**6**			
Ethylenic C=C elongation	350	450 ↙		620 ↘	330		460 ↗	628 ↗				
Quasi C=C twisting	350 ↙	444 ↖		630 ↓	330 ↑		462 ↙	630 ↓		406¶ ↙		
Phenyl torsion	335 ↓	430 ↓										
Structural relaxation			485▿ ←							370 ↖	390 ↑	510
										350	390 ↓	510

▿ The peak observbed in the fs-TA spectra continues its blue shifting ultil ~1.0 μs, which was seen in the ns-TA experiment.
¶ Derived from EADS spectra.

Table S5. The peak wavelengths of the experimental ns-TA spectra and calculated UV-Vis absorption of the **1-IM**, **2-IM**, **3-IM**, **4-IM**, **5-IM** and **6-IM**.

	Peak wavelengths of absorption (nm)											
Photocyclized intermediates	**1-IM**		**2-IM**		**3-IM**		**4-IM**		**5-IM**		**6-IM**	
Experimental	320	465	330	450	353	460	334	450	340	438	345	525
Calculated	310	453	326	521	316	445	331	435	333	470	331	535

Table S6. The time constants of **1-6** in MeCN analyzed by global fitting of the fs-TA results and lifetime of the corresponding intermediates analyzed by monoexponential fitting of the decay of intermediate's UV-Vis absorption.

Lifetime	**1**	**2**	**3**	**4**	**5**	**6**¶
τ_1 (ethylenic C=C bond elongation)	0.39 ps	0.32 ps	0.38 ps	0.35 ps	0.30 ps	-
τ_2 (quasi C=C bond twisting)	1.2 ps	1.6 ps	11.2 ps	1.6 ps	8.9 ps▿	0.4 ps▿
τ_3 (phenyl torsion)	18.9 ps	14.3 ps	4.07 ns	12.9 ps		
τ_4 (photocyclized intermediate)	159 s	-	-	761 s	1139 s	-

▿ The decay of quasi C=C bond twisting and phenyl torsion take place at the same time.
¶ The process of the ethylenic C=C bond elongation of **6** in MeCN is within the instrument response time (τ<150 fs).

6.2 Ultrafast time-resolved spectroscopy of TPE derivatives in thin film

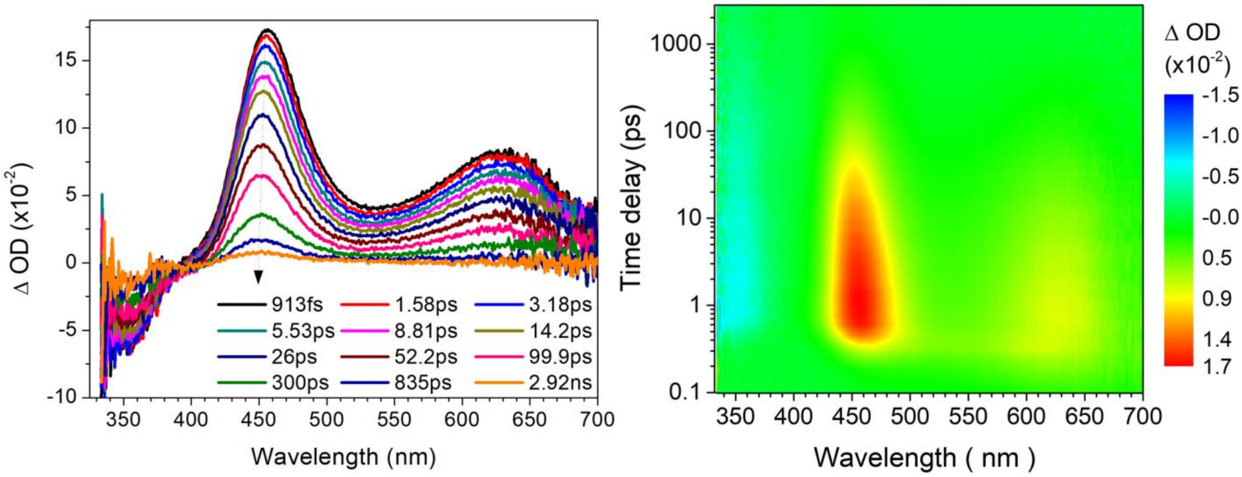

Figure S48. fs-TA of **1** in film acquired after 267 nm irradiation: (Left) selected spectra at different delay times; (Right) contour plots of the spectroscopic responses.

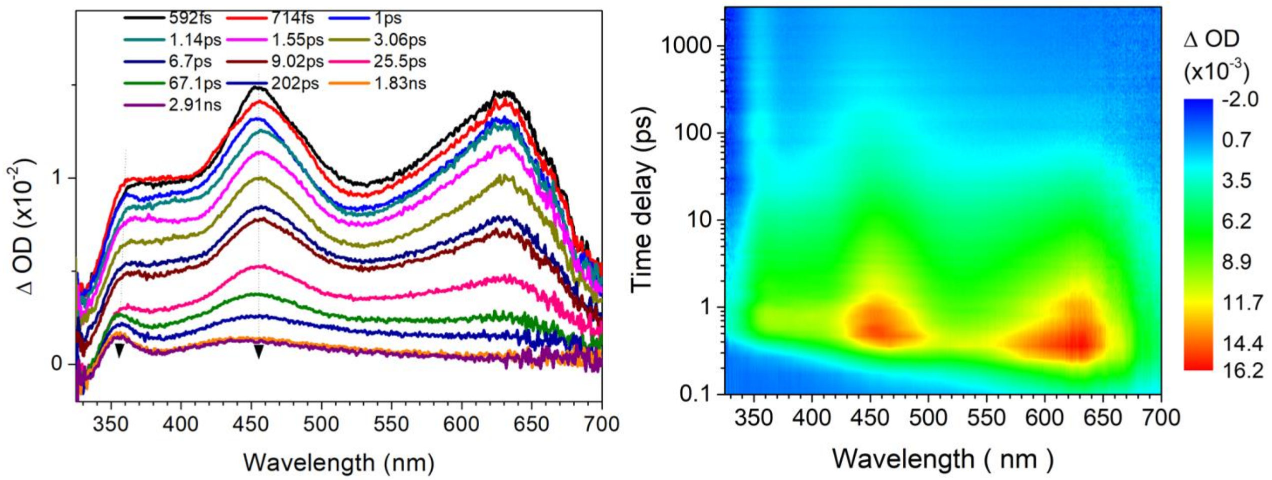

Figure S49. fs-TA of **2** in film acquired after 267 nm irradiation: (Left) selected spectra at different delay times; (Right) contour plots of the spectroscopic responses.

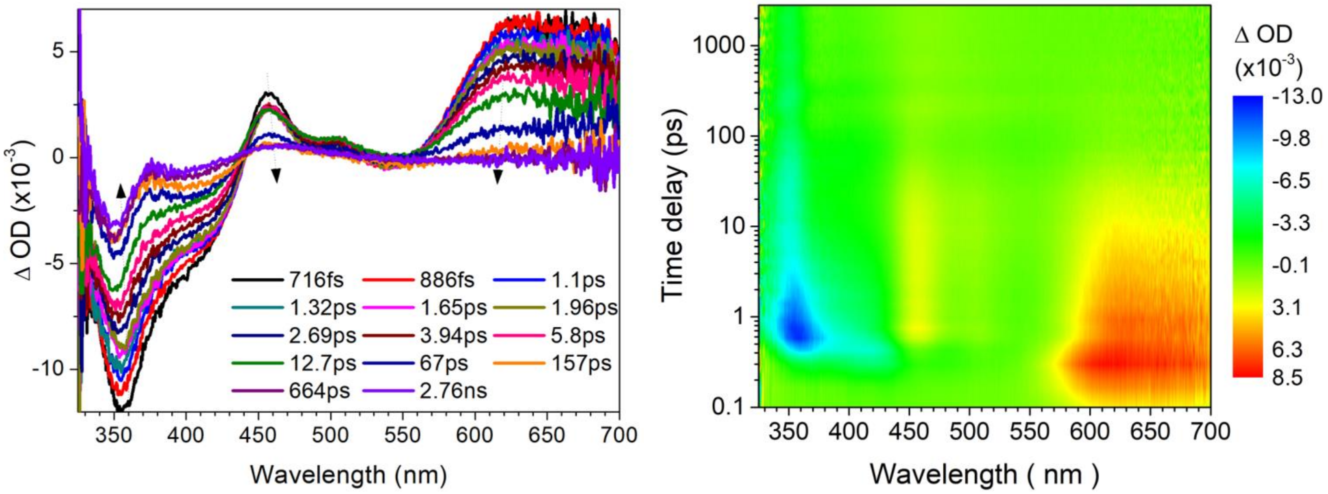

Figure S50. fs-TA of **3** in film acquired after 267 nm irradiation: (Left) selected spectra at different delay times; (Right) contour plots of the spectroscopic responses.

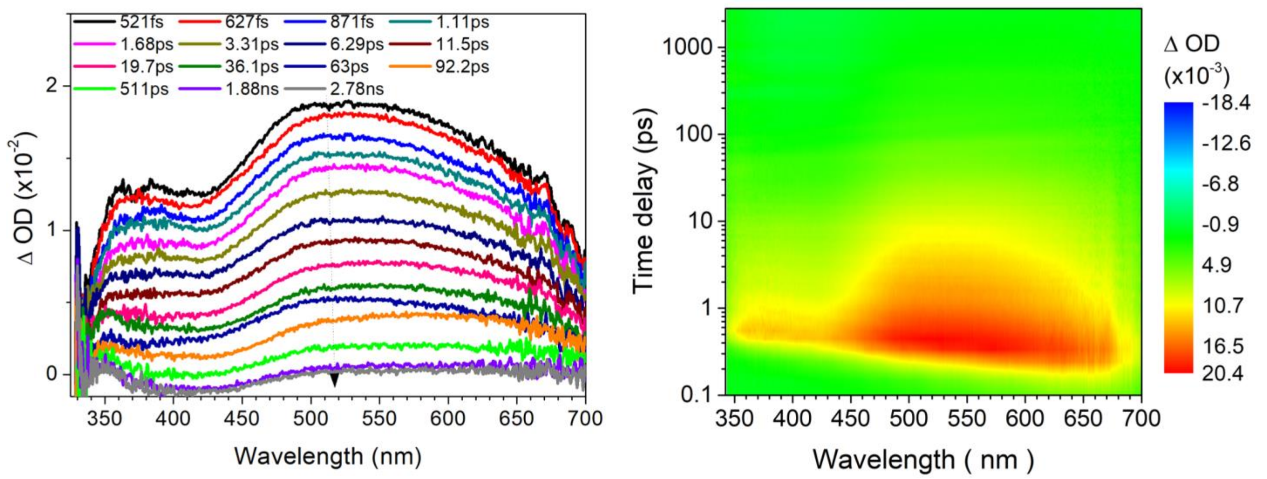

Figure S51. fs-TA of **4** in film acquired after 267 nm irradiation: (Left) selected spectra at different delay times; (Right) contour plots of the spectroscopic responses.

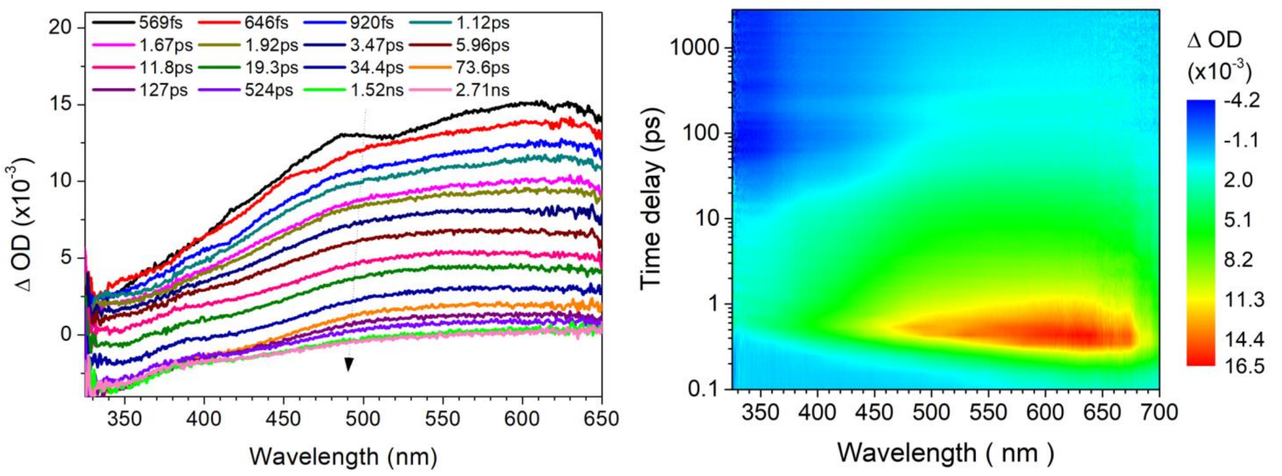

Figure S52. fs-TA of **5** in film acquired after 266 nm irradiation: (Left) selected spectra at different delay times; (Right) contour plots of the spectroscopic responses.

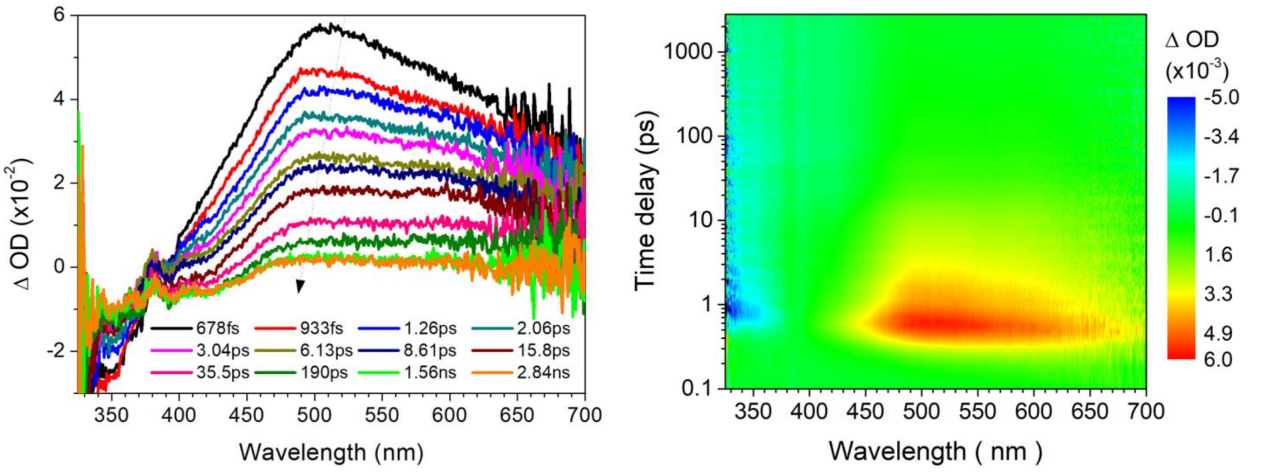

Figure S53. fs-TA of **6** in film acquired after 266 nm irradiation: (Left) selected spectra at different delay times; (Right) contour plots of the spectroscopic responses.

The continuous changes of the ultrafast spectra of **1-6** in film were also studied by the fs-TA after 267 nm excitation. Unlike the spectra obtained in solution, only one process with decaying absorption bands at 450 nm and 630 nm was observed for **1** in film as shown in the Figure S48. This indicates that both the ethylenic C=C bond twisting and phenyl torsion processes are inhibited and no photocyclized intermediate forms in film. Similar behavior is also observed for the TPE derivatives **2-6** in film upon excitation, however, there is no certainty if the photocyclized intermediate forms or not in **4** and **6**.

7 Computational studies

The computed UV-Vis and Raman spectra of **1-IM**, **2-IM**, **3-IM**, **4-IM**, **5-IM** and **6-IM** in acetonitrile are shown in the main manuscript and **Supporting Information section 6.1**. The optimized structures of the photocyclized-intermediates **1-IM**, **2-IM**, **3-IM**, **4-IM**, **5-IM** and **6-IM** in acetonitrile are shown in the **Supporting Information section 7.4.1**.

7.1 The electron density difference between S_{1-FC} and S_0 of TPE derivatives

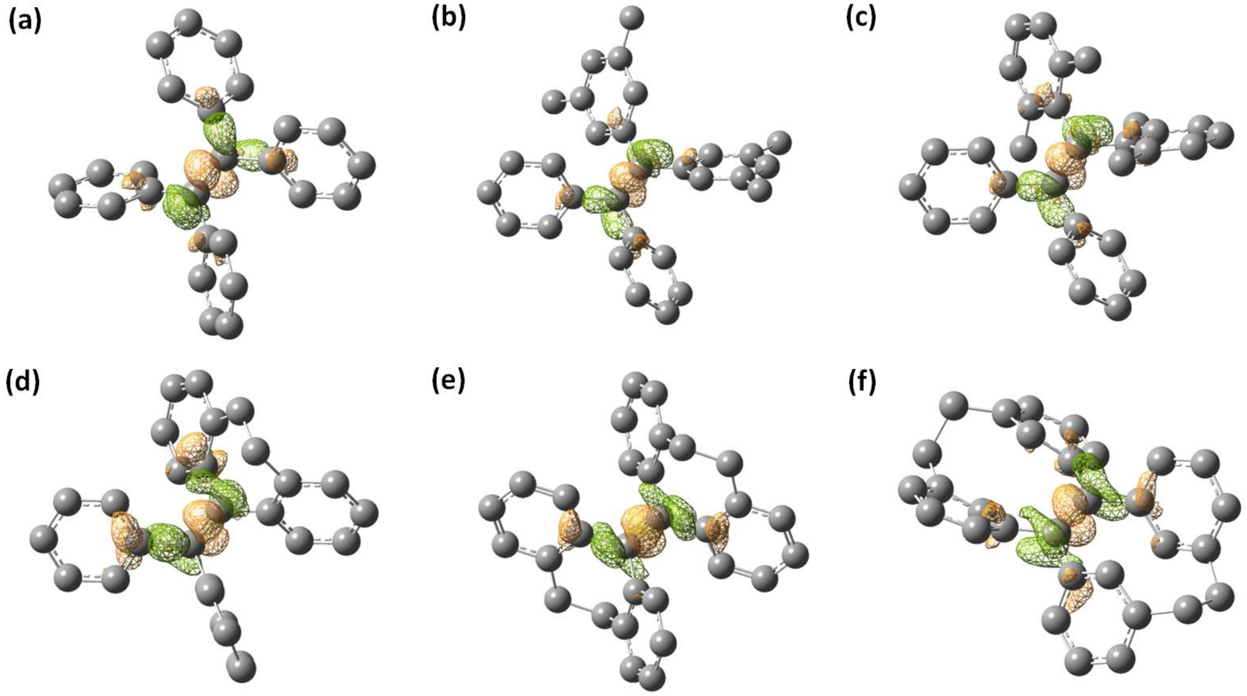

Figure S54. **The electron density difference (EDD) between the Frank-Condon excited state (S_{1-FC}) and the ground state (S_0) of TPE derivatives in acetonitrile upon photon absorption: (a) 1**; **(b) 2**; **(c) 3; (d) 4**; **(e) 5**; **(f) 6**. The source and destination of electron density flow are indicated with orange wireframe and green wireframe; EDD are calculated at M062X/6-311G(d) [31-32] using Gaussian 09 package; [33] All hydrogen atoms are omitted for clarity; Iso of density ＝0.003.

All shown plots demonstrate clearly that in all cases, **(a)-(f)**, the central C=C bond of the TPE unit is the major source of the electron density, which upon photoexcitation it is relocated to the adjacent C-C(Ph) bonds. This is further well reflected in the optimized geometries of these molecules in their first excited state (S_1) that show significant elongation of the C=C bond basically losing its double bond character which is concomitantly associated with a noticeable shortening of the C-C(Ph) bonds that serve fittingly as the destination of the electron density flow.

7.2 The IRC of 1 through the transition state of Ph torsion and C=C twisting in ground state

7.2.1 The IRC of 1 through the transition state of Ph torsion in ground state

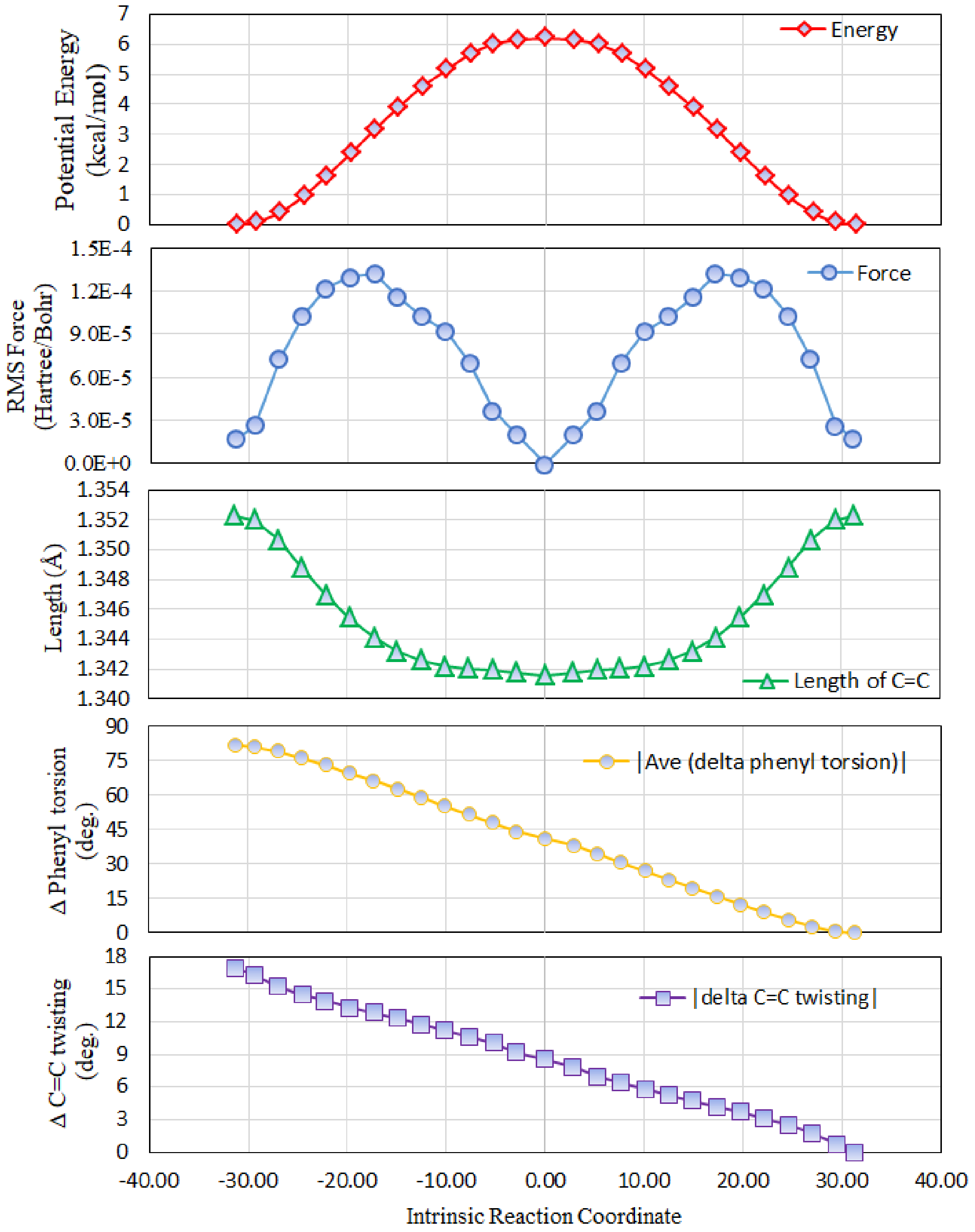

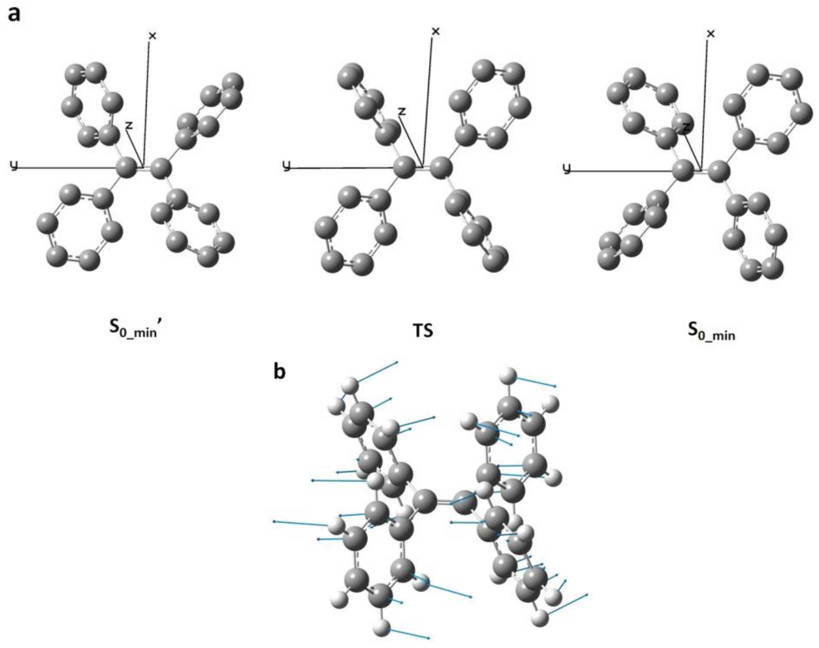

Figure S55. IRC (intrinsic reaction coordinate) [43] analysis of phenyl torsion of 1 in MeCN in the ground state. The change of potential energy, the root mean square (RMS) force, the ethylenic C=C bond length, the average changes in dihedral angles of phenyl torsion and the change in the dihedral angle of ethylenic C=C bond twisting from the S_{0_min} geometry (IRC = 31.29) as defined in Table S7 are shown in the figure from the top to bottom. The IRC = 0 corresponds to the transition state (TS) of the phenyl torsion, while the left and right ends correspond to energy minima in the S_0 state (S_{0_min}', S_{0_min}), and their corresponding structures are shown in (**a**). Normal mode displacement vectors in TS of the conrotatory movement of the phenyl rings for vibrations at -36.72 cm^{-1} are shown in (**b**). All calculated at M062X/6-311G (d) using Gaussian 09 package. [33] Selected data points can be found in the Table S7.

In the minima structures of **1** in MeCN, it has ethylenic C=C twisting angle ($\tau_{C21\text{-}C1\text{-}C2\text{-}C15}$) of about 8° ($S_{0\_min}$) or -8° ($S_{0\_min}$') with ethylenic bond length of 1.352 Å, while in transition state they are ~0° and 1.342 Å, respectively. Along the minimum energy path (MEP), the phenyl torsion of **1** in the ground state goes from one minimum energy structure with phenyl group orientation anticlockwise (S_{0_min}) to another minimum energy structure with the clockwise orientation of the phenyl ring (S_{0_min}') passing its transition state and resulting in the phenyl rings' torsion angles of ~50° (*e.g.* $\tau_{C1\text{-}C2\text{-}C15\text{-}C20}$).

In the ground state, the phenyl torsion in **1** in MeCN is weakly coupled with the motion of the ethylenic C=C bond twisting, in which the torsion of the phenyl rings can be < 90° from S_{0_min} to S_{0_min}' with slight changes of the ethylenic C=C twisting ($\Delta\tau_{C21\text{-}C1\text{-}C2\text{-}C15}$ < 17°) and the bond length ($d_{C1=C2}$ < 0.01 Å). The torsional barrier is rather small (< 6.21 kcal/mol). Furthermore, the IRC calculation result shows that the average change of the dihedral angles of phenyl rings increases as the ethylenic C=C bond twisting angle increases along the MEP. Their average change is well fitted by the linear function: Δ twisting = 0.1823 Δ average torsion + 1.0305 (Figure S56), which means that the average change of the phenyl torsion is linearly coupled with the ethylenic C=C twisting.

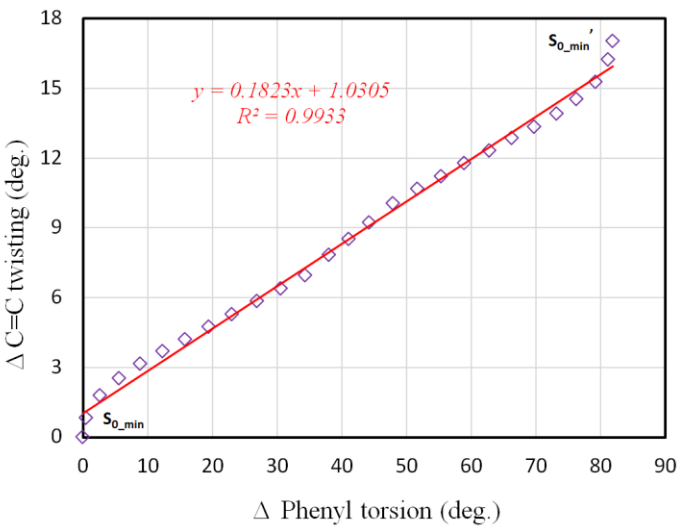

Figure S56. Phenyl torsion in **1** in MeCN in the ground state: linear fit of the change of ethylenic C=C bond twisting as a function of the average changes of dihedral angles of the phenyl rings. Data points can be found in the Table S7.

Table S7. IRC (-31.29~31.29) analysis of phenyl torsion of **1** in MeCN in the ground state.

IRC	Δ Energy (kcal/mol) ‡	Length of C=C (Å) §	**Δ Torsion (deg.)** ¶	Δ Twisting (deg.) Δ
-31.29	0.00	1.352	81.85	17.03
-29.37	0.10	1.352	81.22	16.21
-26.96	0.40	1.351	79.24	15.24
-24.55	0.94	1.349	76.34	14.53
-22.14	1.61	1.347	73.16	13.91
-19.73	2.36	1.345	69.76	13.34
-17.32	3.15	1.344	66.27	12.82
-14.91	3.91	1.343	62.74	12.31
-12.50	4.57	1.343	59.05	11.76
-10.09	5.15	1.342	55.36	11.20
-7.68	5.68	1.342	51.73	10.68
-5.28	5.99	1.342	47.95	10.06
-2.88	6.12	1.342	44.23	9.20
0.00	6.21	1.342	41.14	8.51
2.88	6.12	1.342	38.06	7.82
5.28	5.99	1.342	34.38	6.97
7.68	5.68	1.342	30.61	6.35
10.09	5.15	1.342	26.88	5.83
12.50	4.57	1.343	23.13	5.26
14.91	3.91	1.343	19.43	4.72
17.32	3.15	1.344	15.80	4.21
19.73	2.36	1.345	12.29	3.69
22.14	1.61	1.347	8.90	3.12
24.55	0.94	1.349	5.63	2.50
26.96	0.39	1.351	2.60	1.79
29.36	0.10	1.352	0.59	0.82
31.29	0.00	1.352	0.00	0.00

‡ The change of potential energy from the S_{0_min} geometry (IRC=31.29). § The length of ethylenic C=C bond. ¶ The average changes in dihedral angles of phenyl torsion from the S_{0_min} geometry: Δ Torsion = |Ave (delta phenyl torsion)| = |0.25Σ *(delta Tors$_i$)*|, *delta Tors$_i$* = $\Delta\tau_{C20\text{-}C15\text{-}C2\text{-}C1}$, $\Delta\tau_{C14\text{-}C9\text{-}C2\text{-}C1}$, $\Delta\tau_{C22\text{-}C21\text{-}C1\text{-}C2}$, $\Delta\tau_{C8\text{-}C3\text{-}C1\text{-}C2}$. See Supporting Information section 7.5. Δ The change in the dihedral angle of ethylenic C=C bond twisting from the S_{0_min} geometry: Δ Twisting = |delta C=C twisting|, delta C=C twisting = $\Delta\tau_{C21\text{-}C1\text{-}C2\text{-}C15}$.

7.2.2 The IRC of 1 through the transition state of C=C twisting in ground state

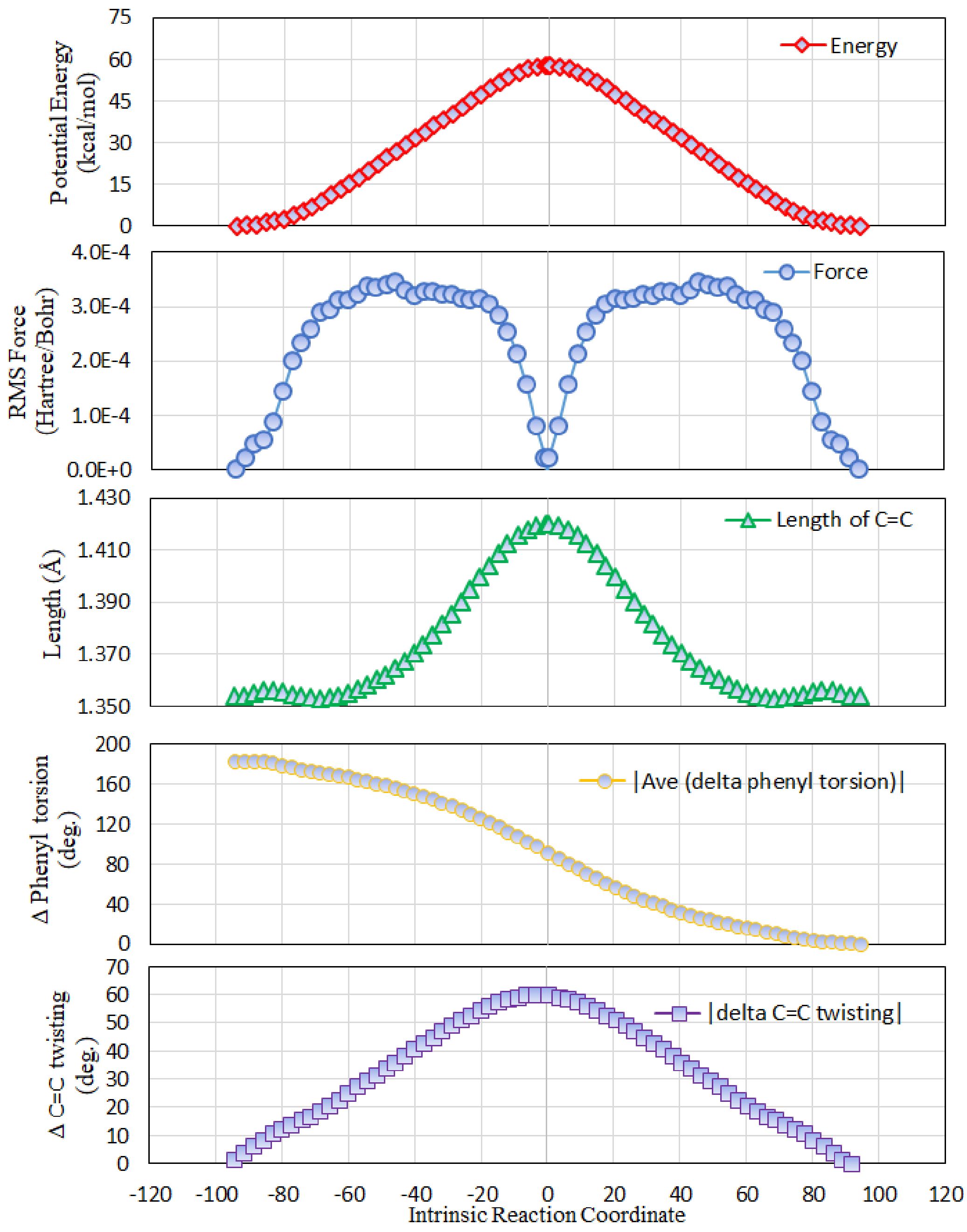

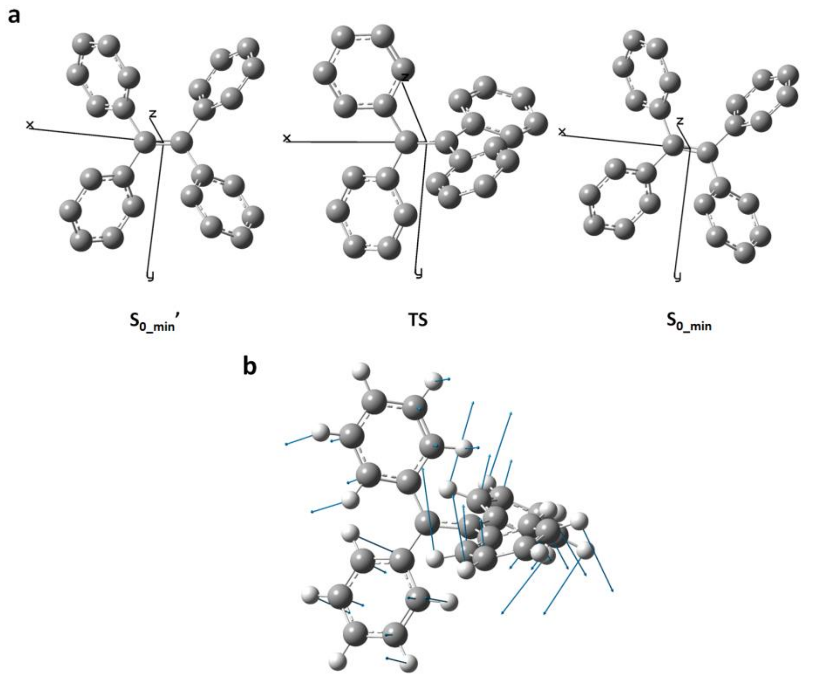

Figure S57. IRC (intrinsic reaction coordinate) [43] analysis of ethylenic C=C bond twisting of 1 in MeCN in the ground state. The change of potential energy, the root mean square (RMS) force, the ethylenic C=C bond length, the average changes in dihedral angles of phenyl torsion and the change in the dihedral angle of ethylenic C=C bond twisting from the S_{0_min} geometry (IRC = 94.36) as defined in Table S8 are shown in the figure from the top to bottom. The IRC = 0 corresponds to the transition state (TS) of the ethylenic C=C twisting, while the left and right ends correspond to energy minima in the S_0 state (S_{0_min}', S_{0_min}), and their corresponding structures are shown in (**a**). Normal mode displacement vectors in TS of ethylenic C=C twisting for vibrations at -52.76 cm^{-1} are shown in (**b**). All calculated at M062X/6-311G (d) using Gaussian 09 package. [33] Selected data points can be found in the Table S8.

Remarkably, the C=C bond twisting is associated with significant changes in the C=C bond length and potential energy. The motion of the ethylenic C=C bond twisting in **1** in MeCN in the ground state is strongly coupled with the phenyl torsion, with the transition state (~60.03°) along this coordinate being 57.83 kcal/mol higher than the S_{0_min}. Along the minimum energy path, the ethylenic C=C bond twists from the minimum (S_{0_min}) to the cusp at the transition state (TS) and then goes back while during this process the phenyl rings having a larger degree of torsional freedom complete a full 180° torsion and the length of C=C bond varies a lot from 1.354 Å to 1.420 Å. The IRC calculation result shows that the change of the twisting angle is as large as 60°. As the angle of the C=C bond twisting increases in the first half of the process and decreases in the later half of the process, the dihedral angles of the phenyl rings increase all the time. Their average change is well fitted by the quadratic function: Δ twisting = -0.0068 (Δ torsion) ^{2} +1.2562 Δ torsion + 3.8402 (Figure S58), which means the change of ethylenic C=C twisting is quadratically coupled with the phenyl torsion.

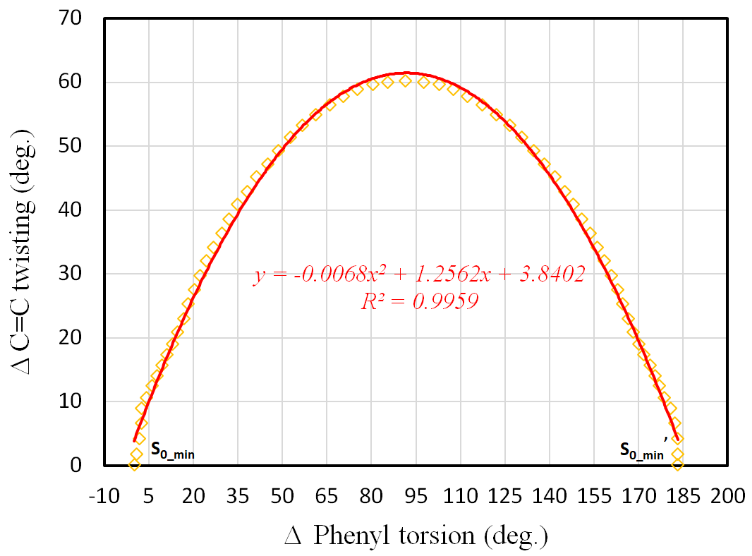

Figure S58. Ethylenic C=C twisting of **1** in MeCN in the ground state: polynomial fit of the change of ethylenic C=C twisting as a function of the average changes of the dihedral angles of the phenyl rings. Data points can be found in the Table S8.

Table S8. IRC (-94.36~94.36) analysis of ethylenic C=C twisting of **1** in MeCN in the ground state.

IRC	∆ Energy (kcal/mol) [ǂ]	Length of C=C (Å) [§]	∆ Torsion (deg.) [¶]	**∆ Twisting (deg.)** [∆]
-94.36	0.00	1.354	183.18	0.00
-91.52	0.19	1.354	183.26	1.66
-88.68	0.54	1.355	183.00	4.08
-85.84	1.02	1.356	182.22	6.50
-82.99	1.59	1.356	180.68	8.70
-80.15	2.46	1.355	178.80	10.56
-77.31	3.73	1.354	177.04	12.33
-74.47	5.30	1.354	175.34	13.95
-71.63	7.03	1.353	173.70	15.56
-68.79	8.98	1.353	171.95	17.17
-65.94	11.03	1.353	170.12	18.93
-63.10	13.16	1.354	168.30	20.86
-60.26	15.38	1.355	166.50	22.88
-57.41	17.57	1.356	164.70	25.09
-54.57	19.86	1.358	162.82	27.37
-51.73	22.19	1.360	160.76	29.61
-48.88	24.51	1.362	158.58	31.87
-46.04	26.89	1.364	156.14	34.04
-43.19	29.23	1.367	153.56	36.19
-40.35	31.47	1.370	150.93	38.41
-37.51	33.72	1.374	148.12	40.64
-34.66	36.01	1.377	145.06	42.79
-31.82	38.26	1.381	141.83	44.95
-28.98	40.52	1.385	138.34	47.10
-26.13	42.77	1.390	134.59	49.19
-23.29	44.97	1.395	130.64	51.19

-20.45	47.20	1.400	126.44	53.06
-17.60	49.41	1.404	121.98	54.75
-14.76	51.52	1.409	117.34	56.26
-11.92	53.47	1.412	112.56	57.58
-9.08	55.18	1.416	107.66	58.69
-6.24	56.54	1.418	102.67	59.41
-3.40	57.47	1.419	97.61	59.87
0.00	57.83	1.420	91.59	60.03
3.40	57.47	1.419	85.57	59.87
6.24	56.54	1.418	80.51	59.41
9.08	55.18	1.416	75.52	58.69
11.92	53.47	1.412	70.62	57.58
14.76	51.52	1.409	65.84	56.26
17.60	49.41	1.404	61.20	54.75
20.45	47.20	1.400	56.74	53.06
23.29	44.97	1.395	52.54	51.19
26.13	42.77	1.390	48.59	49.19
28.98	40.52	1.385	44.83	47.10
31.82	38.26	1.381	41.34	44.95
34.66	36.02	1.377	38.12	42.80
37.51	33.72	1.374	35.06	40.64
40.35	31.47	1.370	32.25	38.41
43.19	29.23	1.367	29.62	36.19
46.04	26.89	1.364	27.04	34.04
48.88	24.51	1.362	24.60	31.87
51.73	22.19	1.360	22.42	29.61
54.57	19.86	1.358	20.36	27.37
57.41	17.57	1.356	18.47	25.09
60.26	15.38	1.355	16.68	22.88
63.10	13.16	1.354	14.88	20.86
65.94	11.03	1.353	13.05	18.93
68.79	8.98	1.353	11.22	17.17
71.63	7.03	1.353	9.48	15.56
74.47	5.30	1.354	7.83	13.95
77.31	3.73	1.354	6.14	12.33
80.15	2.46	1.355	4.37	10.56
82.99	1.59	1.356	2.50	8.70
85.84	1.02	1.356	2.63	6.50
88.68	0.54	1.355	1.98	4.08
91.52	0.19	1.354	0.94	1.66
94.36	0.00	1.354	0.00	0.00

ǂ The change of potential energy from the S_{0_min} geometry (IRC=94.36). § The length of ethylenic C=C bond. Δ The change in the dihedral angle of ethylenic C=C bond twisting from the S_{0_min} geometry: ΔTwisting = |delta C=C twisting|, delta C=C twisting = $\Delta\tau_{C21\text{-}C1\text{-}C2\text{-}C15}$. ¶ The average changes in dihedral angles of phenyl torsion from the S_{0_min} geometry: Δ Torsion = |Ave (delta phenyl torsion)| = |*0.25Σ (delta Tors$_i$)*|, *delta Tors$_i$* = $\Delta\tau_{C20\text{-}C15\text{-}C2\text{-}C1}$, $\Delta\tau_{C14\text{-}C9\text{-}C2\text{-}C1}$, $\Delta\tau_{C22\text{-}C21\text{-}C1\text{-}C2}$, $\Delta\tau_{C8\text{-}C3\text{-}C1\text{-}C2}$. See Supporting Information section 7.5.

7.3 The potential energy hypersurface of 1 in ground state and excited state

To differentiate between several possible dominant relaxation channels (*i.e.* quasi C=C bond twisting, phenyl torsion and photocyclization) in **1**-**6** upon excitation, a quantitative understanding of the coupling relationship between the molecular motions is imperative. The ethylenic C=C bond lengths of **1** in MeCN in the PES calculation (Figure 1) [44] are 1.351 Å in S_0 and 1.471 Å in S_1, respectively. In the excited state, the elongation of the central C1-C2 bond in **1** facilitates the large angle of the quasi C=C twisting, which is strongly coupled with the torsion of the phenyl rings. However, the coupling relationship is different from that in the ground state. Along the MEP in the excited state, the quasi C=C twisting ($\tau_{C21\text{-}C1\text{-}C2\text{-}C15}$) increases from several degrees to 60 degrees while the dihedral angle of the phenyl ring ($\tau_{C20\text{-}C15\text{-}C2\text{-}C1}$) decreases from 50 degrees to 25 degrees, a process during which the potential energy is reduced by almost 20 kcal/mol.

While the dominant motion for the physically viable process of the motions of **1** in MeCN in the ground state is phenyl torsion, the major motion in the excited state is the quasi C=C twisting. Obviously, the ethylenic twisting that can be as large as ~60° is strongly coupled with phenyl torsion both in the excited state and ground state. The barrier of the ethylenic twisting is much higher (> 50 kcal/mol) than that of phenyl torsion in the ground state, in which the ethylenic twisting angle is less than 10°. The calculation results show that the change of the dihedral angle of the quasi C=C twisting in the excited state is well fitted by the quadratic function: Δ twisting = 0.0671 (Δ torsion) ^{2} - 3.9048 Δ torsion - 2.7952 (Figure S59), in which the quadratic coefficient (0.0671) is 10 times larger than that of the ethylenic twisting in the ground state, indicating that the quasi C=C twisting coupled with phenyl torsion is significantly stronger in the excited state. Additionally, the minimum energy path (Figure S59, Table S9) reveals that simple harmonic oscillations of the quasi C=C twisting may take place in the excited state and then the damping process may result in the relaxation to the S_{1_min}. [45] For example, the dihedral angle of the quasi C=C twisting varies from 39° to 54°, while the phenyl torsion angle remains roughly the same at 27°.

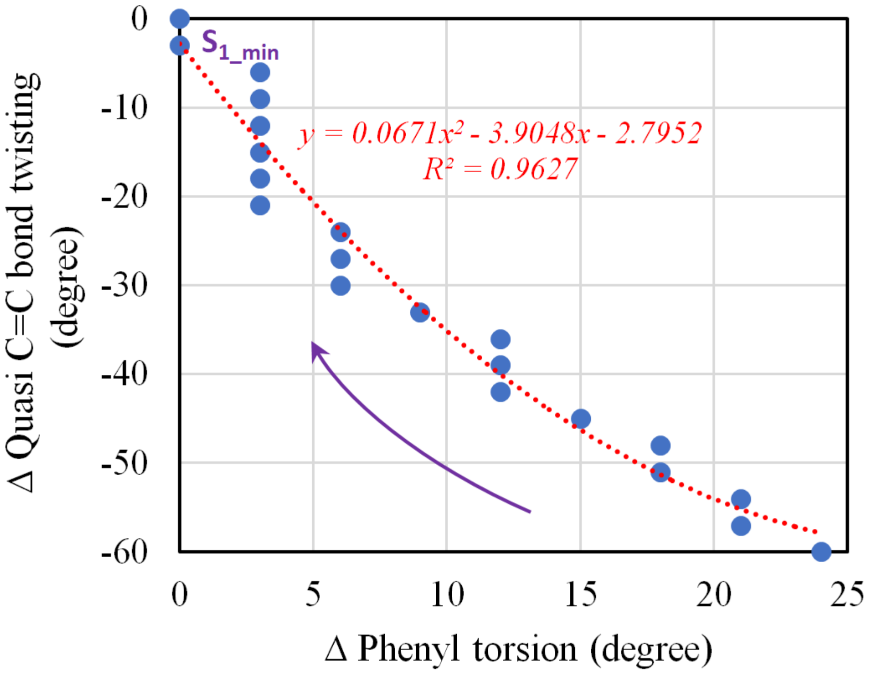

Figure S59. The minimum energy path of **1** in MeCN in the excited state: polynomial fit of the change of the quasi C=C twisting ($\Delta\tau_{C21\text{-}C1\text{-}C2\text{-}C15}$) as a function of the change of the dihedral angle of the phenyl torsion ($\Delta\tau_{C20\text{-}C15\text{-}C2\text{-}C1}$). The purple arrow indicates the direction to the S_{1_min} geometry. Data points can be found in the Table S9.

Table S9. The minimum energy path of PES as a function of the quasi C=C twisting ($\tau_{C21\text{-}C1\text{-}C2\text{-}C15}$) and the dihedral angle of the phenyl torsion ($\tau_{C20\text{-}C15\text{-}C2\text{-}C1}$) of **1** in MeCN in the excited state.

Twisting (deg.)	Torsion (deg.)	Energy (kcal/mol)	Δ Twisting(deg.)[Δ]	Δ Torsion (deg.)[¶]	Δ Energy (kcal/mol)[‡]
0	48	84.62	-60	24	25.00
3	45	82.21	-57	21	22.59
6	45	79.68	-54	21	20.06
9	42	77.24	-51	18	17.62
12	42	74.99	-48	18	15.37
15	39	72.73	-45	15	13.11
18	36	70.95	-42	12	11.34
21	36	68.88	-39	12	9.27
24	36	67.43	-36	12	7.81
27	33	65.82	-33	9	6.21
30	30	64.66	-30	6	5.04
33	30	63.35	-27	6	3.73
36	30	62.44	-24	6	2.82
39	27	61.57	-21	3	1.95
42	27	60.88	-18	3	1.27
45	27	60.44	-15	3	0.82
48	27	60.14	-12	3	0.52
51	27	59.94	-9	3	0.33
54	27	59.80	-6	3	0.19
57	24	59.71	-3	0	0.09
**60**	**24**	**59.62**	0	0	0.00

[Δ] The changes in dihedral angles of the quasi C=C twisting from the $\mathbf{S_{1\_min}}$ geometry (60°): ΔTwisting = $\Delta\tau_{C21\text{-}C1\text{-}C2\text{-}C15}$. [¶] The changes in dihedral angles of phenyl torsion from the $\mathbf{S_{1\_min}}$ geometry (24°): Δ Torsion = $\Delta\tau_{C20\text{-}C15\text{-}C2\text{-}C1}$. See Supporting Information section 7.5. [‡] The changes of potential energy from the $\mathbf{S_{1\_min}}$ geometry (59.62 kcal/mol).

7.4 The Gibbs free energy and MOs of the TPE derivatives

7.4.1 The Gibbs free energy of the TPE derivatives

Theoretical calculations were employed to gain more insight into energetics of the observed photocyclization processes and to study and compare thermodynamic stability of TPE derivatives **1-6**, with that of their photocyclized analogues and proposed intermediates. Their optimized ground state (S_0) geometries and Gibbs free energies of the reactions in acetonitrile were calculated at M062X/6-311G (d) level using Gaussian 09 software package. [33] While only one optimized (minimum energy) geometry for the TPE derivatives **1-6** and their corresponding photocyclized products is possible, for each 4a,4b-dihydrophenanthrene-type intermediate* of **1-6** there are at least two different possible structures that have to be considered. Namely, one with the two hydrogen atoms attached to the carbon atom of the newly formed C-C bond (highlighted with a red circle) in *anti*-conformation and one with the aforementioned hydrogen atoms in *syn*-conformation. We have computed and compared all possible structural configurations and the respective energies for each intermediate. In the following energy diagrams, optimized structures of the photocyclized intermediates **1-IM–6-IM** with the lowest energy are shown. The computed UV and Raman spectra of the intermediates **1-IM–6-IM** in acetonitrile are shown in the **Supporting Information section 6.1**.

* Although different cyclization patterns leading to intermediary products other than dihydrophenanthrene-type derivatives are also conceivable during the photocyclization of compounds **1-6**, we note that an empirically verified rule states that cyclization occurs only from those species whose "free valence number" exceeds the value of 1(unity). [46-51] In agreement with this rule, our experimental findings, and an abundant body of literature [2, 36-40, 52], the 4a, 4b-dihydrophenanthrene-type intermediates were considered.

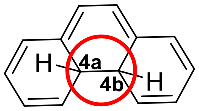

4a, 4b-dihydrophenanthrene

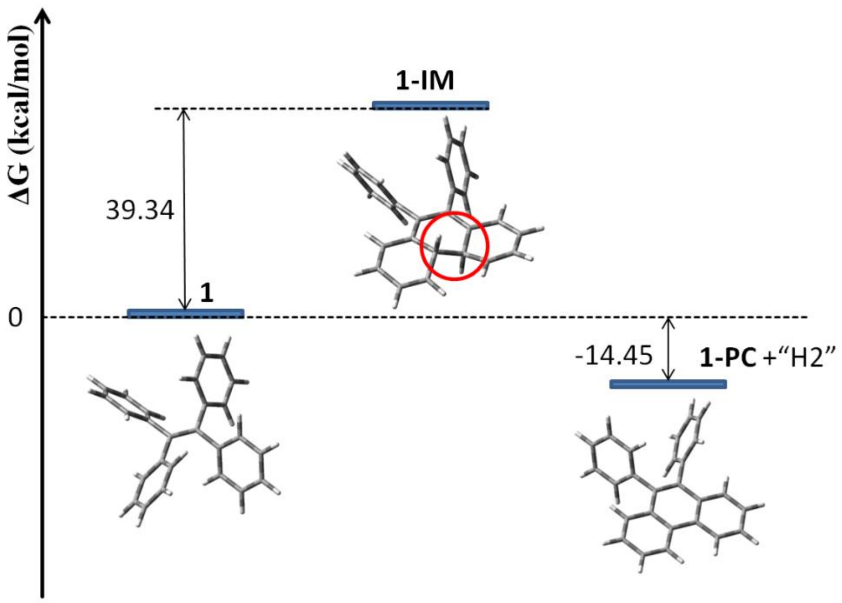

Figure S60. The Gibbs free energies and geometric structures of **1**, **1-IM** and **1-PC**.

In case of molecule **1** (TPE), the polycyclic photocyclized 4a,4b-dihydrophenanthrene-type intermediate **1-IM** of the lowest energy has the hydrogen atoms in 4a and 4b positions (highlighted with the red circle) in *anti(periplanar)* conformation, and is roughly 39.3 kcal/mol higher in energy than **1**, which can be certainly attributed to its highly strained geometry resulting from the obvious distortion of the phenyl rings involved in the photocyclization process from planarity and partial loss of aromaticity/conjugation upon intermediate formation. The presence of the oxidizing agents (I_2, O_2) causes the facile abstraction of two hydrogens and facilitates formation of dehydrogenated ring-fused phenanthrene-type photocyclized product **1-PC**. According to our calculations, it is thermodynamically somewhat more stable (14.4 kcal/mol) than the original compound **1**, due to the presence of a more extended fully aromatic phenanthrene π system in **1-PC**. The dehydrogenation step is strongly exothermic with ΔG amounting to as much as ca. 54 kcal/mol confirming spontaneous formation of **1-PC** in accordance with experimental observations.

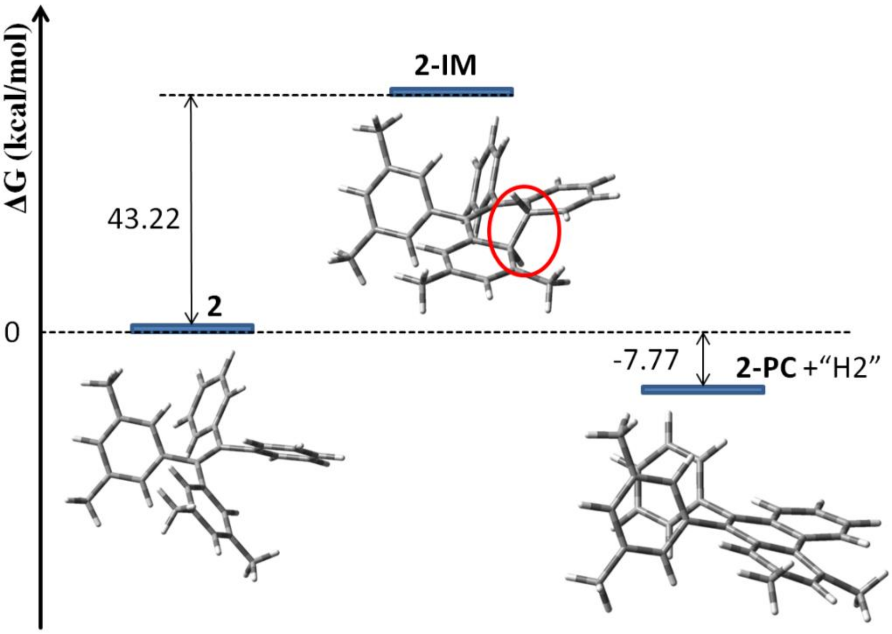

Figure S61. The Gibbs free energies and geometric structures of **2**, **2-IM** and **2-PC**.

Similarly, according to our calculations, **2** would form the polycyclic photocyclized dihydrophenanthrene-type intermediate **2-IM** upon photocyclizations, in which the hydrogen atoms at the saturated tetrahedral carbon atoms (4a and 4b positions, highlighted with the red circle) are in *anti(periplanar)* conformation. This structure is ca. 43 kcal/mol higher in energy than **2**, which in analogy to the structurally very similar **1**, can be certainly attributed to its strained geometry resulting from the distortion of the phenyl rings from planarity and partial loss of aromaticity/conjugation upon **2-IM**'s formation. The presence of the Me groups in *meta* position of the phenyl rings in **2** does not appear to noticeably destabilize the intermediate. Also, in this case, calculations suggest that formal abstraction of two hydrogens leads to formation of dehydrogenated ring-fused phenanthrene-type photocyclized product **2-PC**, which is thermodynamically more stable than **2**. Interestingly, in contrast to **1-PC**, **2-PC** is more stable than its parent compound by only roughly 7 kcal/mol, one possible reason for that could be the fact that the phenanthrene ring in **2-PC** is not ideally planar, thus somewhat diminishing the stabilizing effect of re-aromatization in **2-PC** upon dehydrogenation. While the overall transformation process **2** to **2-PC** is on-

ly weakly exothermic, the dehydrogenation step is almost as strongly exothermic for **2** as it is for **1** (ca. 51 kcal).

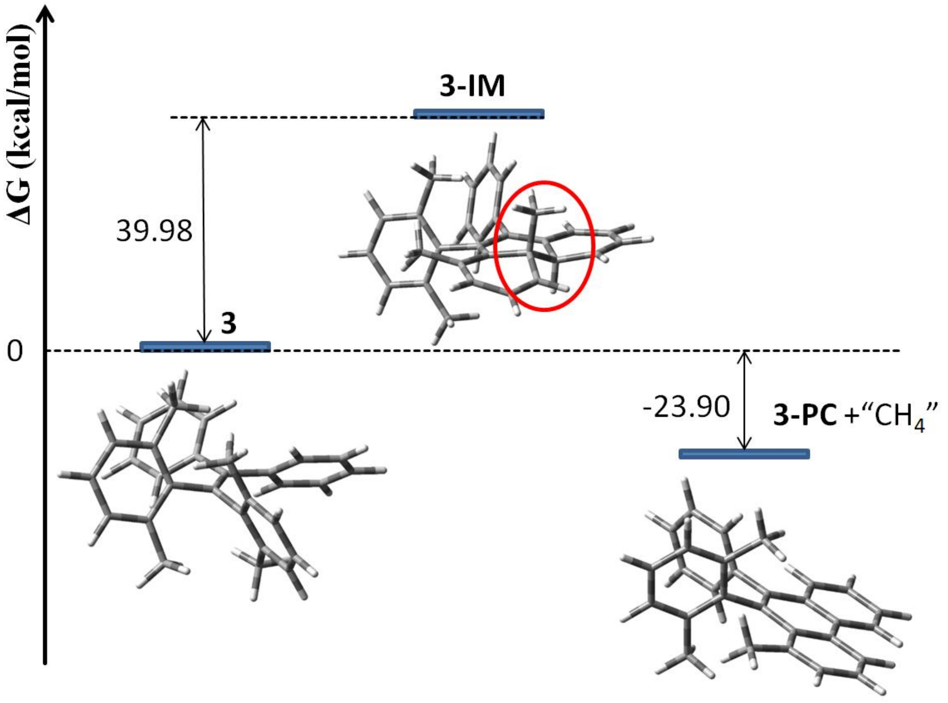

Figure S62. The Gibbs free energies and geometric structures of **3**, **3-IM** and **3-PC**.

Photocyclization of **3** is different from all the other compounds in the series in that our calculations suggest that its photocyclized hydrophenanthrene-type intermediate **3-IM** has a methyl group in the 4a position (highlighted with the red circle) in its energetically lowest *anti(periplanar)* conformation. Similar to **1** and **2**, this structure is about 39 kcal/mol higher in energy than its parent compound **3**, which can be attributed to the same reasons that hold true for the formation of **1-IM** and **2-IM**. Remarkably, according to our results, the presence of the methyl groups in *ortho* position of the phenyl rings and in the 4-position of the hydrophenanthrene ring in **3-IM** does not appear to noticeably destabilize the intermediate. Contrary to the previous examples, formation of the stable photocyclized ring-fused phenanthrene-type product **3-PC**, would require formal abstraction of the very stable methane molecule, which is the thermodynamic driving force behind the progress of this reaction. In addition to that, the phenanthrene ring in **3-PC** is almost ideally planar, providing better aromatic stabilization in **3-PC**. Thus, the overall transformation process from **3** to **3-PC** is predicted to be spontaneous and notably more exothermic (ΔG=24 kcal/mol) than in the case of compounds **1** (ΔG= ca. 14 kcal/mol) and **2** (ΔG= ca. 7 kcal/mol), with the re-aromatization step being strongly exothermic (ca. 64 kcal) compared to those of **2** (54 kcal) and **1** (ca. 51 kcal).

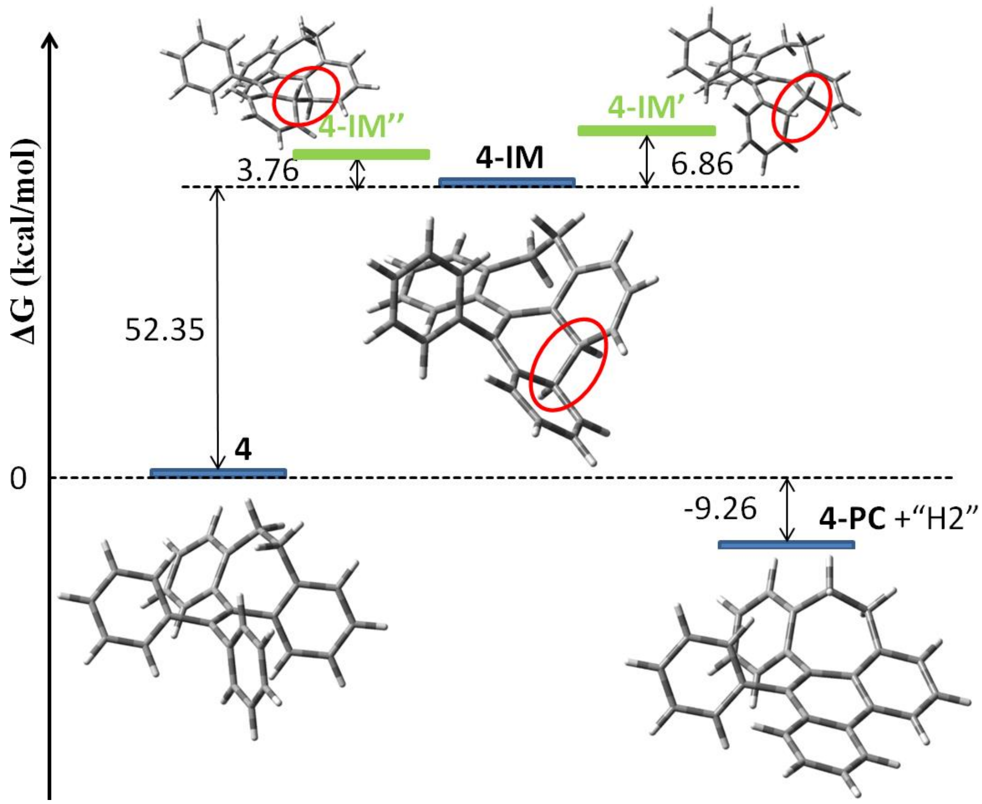

Figure S63. The Gibbs free energies and geometric structures of **4**, **4-IM** and **4-PC**.

Compound **4** appears to be unique bacause in this case our calculations suggest that its overly rigid structure and ensuing lack of flexibility could potentially lead to formation of three polycyclic photocyclized dihydrophenanthrene-type intermediates: *synclinal* **4-IM**, (approximately) *synperiplanar* **4-IM'**, and *antiperiplanar* **4-IM''**, of very similar energies, with **4-IM'** being ca. 7 kcal/mol higher in energy than **4-IM**, and **4-IM''** only ca. 4 kcal/mol higher in energy than **4-IM**, upon photocyclization. Interestingly, in two of them (**4-IM** and **4-IM'**), the hydrogen atoms in 4a and 4b positions of the dihydrophenanthrene ring (highlighted with the red circle) are in *syn*-conformation, as opposed to the more intuitive and prevalently predicted *antiperiplanar*-configuration in the intermediates of all the other compounds. Although we cannot exclude the possibility of **4-IM'**and **4-IM''** being formed and involved in the photocyclization process, we'll limit ourselves in this discussion to **4-IM**, as this specific isomer is the one with the lowest energy, and thus the most likely one to be involved and observed experimentally. Although, being the intermediate of the lowest energy, remarkably, **4-IM** is a long-lived intermediate and has the highest parent compound-intermediate energy gap of ca. 52 kcal/mol of all studied compounds in this series. In analogy to the structurally related but significantly more flexible **1**, **2** and **3**, the energy gap in this case is also due to the significant distortion of the molecule, strain in the 1,4-cycloheptadiene ring and loss of aromatic stabilization in the intermediate. However, the unusually large energy gap is most likely to the extreme rigidity of **4** caused by the presence of the "locking" ethylene link (-CH_2-CH_2-) in it. In this case, the motion of the "locked" phenyl rings is strongly restrained, thus imposing a highly unfavorable *syn*-geometry on the photocyclized intermediate. Also, in this case, calculations suggest that formal abstraction of two hydrogen atoms is a highly exothermic (ΔG=62 kcal/mol) and spontaneous process that leads to formation of phenanthrene-type photocyclized product **4-PC**, which is only roughly 9 kcal/mol thermodynamically more stable than the parent compound **4**. One possible explanation for that could be the considerable ring strain in **4-PC** arising from the ethylene bridge link connecting two phenyl groups in **4-PC**, and thus forming a strained 1,4-cycloheptadiene ring and causing visible distortion of the phenanthrene ring in **4-PC** from planarity, thus reducing the stabilizing effect of rearomatization in **4-PC**.

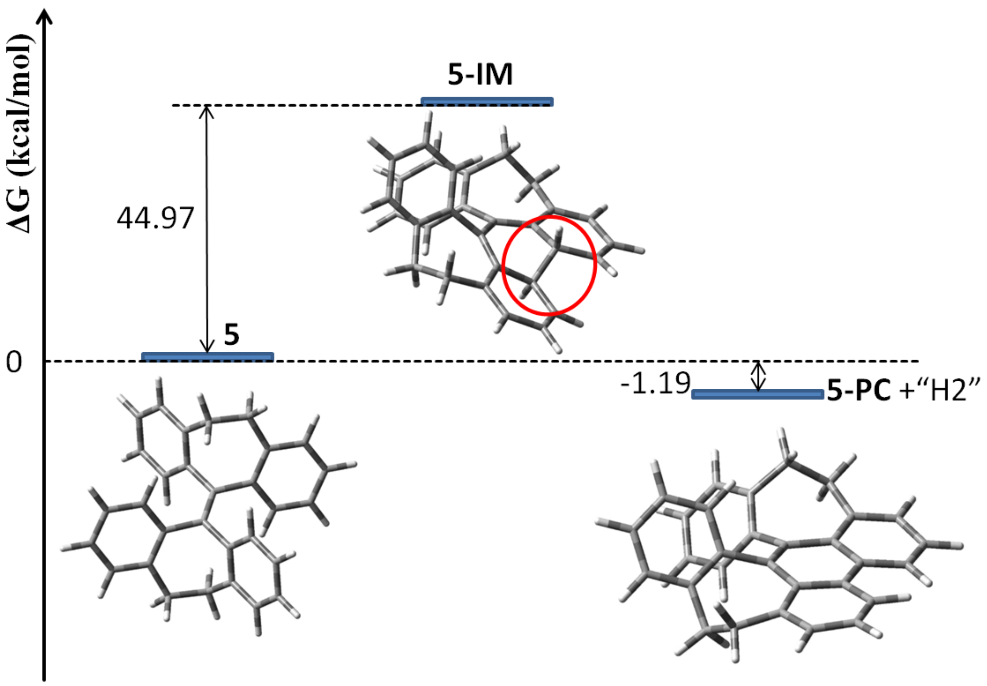

Figure S64. The Gibbs free energies and geometric structures of **5**, **5-IM** and **5-PC**.

Similarly, in **5**, ethylene links on both ends of the TPE increase inherent rigidity of the structure, which, in this case, leads to preferred formation of the polycyclic photocyclized dihydrophenanthrene-type intermediates **5-IM** with the hydrogen atoms in 4a and 4b positions in *antiperiplanar* conformation (highlighted with the red circle). In contrast to **4-IM**, experimental findings indicate that **5-IM** is relatively short-lived and has a parent compound-intermediate energy gap of ca. 45 kcal/mol. In contrast to the structurally related but by far more flexible **1**, **2** and **3**, the large energy gap in this case is primarily due to the strain of the 1,4-cycloheptadiene ring and loss of aromatic stabilization in the intermediate as we saw them in **1-IM**, **2-IM** and **3-IM**. The extreme rigidity of **5** caused by the presence of the “locking” ethylene links prevents the formation of highly distorted and thus energetically unfavorable structures. In this case, the motion of all four “locked” phenyl rings is restrained to such a degree that only minor reorganizations in structure of **5** are possible, and a minor rotation of the phenyl rings will lead to the formation of the photocyclized intermediate. Also, in this case, calculations suggest that dehydrogenation of **5-IM** is a spontaneous and exothermic (ΔG=56 kcal/mol) process that leads to formation of the phenanthrene-type photocyclized product **5-PC**, which, according to our calculations, is barely any more thermodynamically stable than (1 kcal/mol) the parent compound **5**. One possible explanation for that could be that the stabilizing effect of re-aromatization in **5-PC**, is almost entirely offset by the additional ring strain that is introduced into the system upon photocyclization.

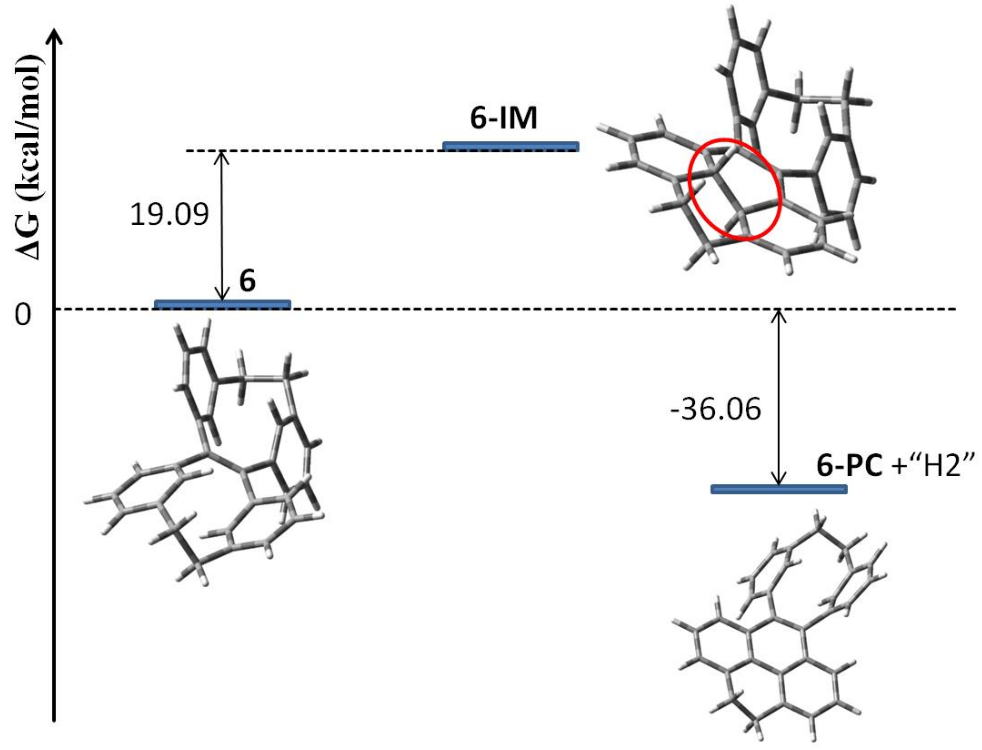

Figure S65. The Gibbs free energies and geometric structures of **6**, **6-IM** and **6-PC**.

For compound **6**, our calculations suggest formation of a photocyclized dihydrophenanthrene-type intermediate, **6-IM**, in its energetically lowest *antiperiplanar* conformation. Remarkably, **6-IM** is only 19 kcal/mol higher in energy than its parent compound **6**. A general rule of thumb stating that reactions with a barrier of 21 kcal/mol or less proceed readily at room temperature, one would expect extraordinary facile formation of **6-IM** under given conditions. [53] This could be attributed to the fact that, although designed to be significantly more rigid than its non-locked analogues, in the optimized structure of **6**, the phenyl rings that form the dihydrophenanthrene ring upon cyclization are offset stacked. Thus, only a minimum of additional structural reorganization in **6** is required for the intermediate to form upon photoexcitation. The overall transformation process from **6** to **6-PC** is predicted to be spontaneous and notably more exothermic (ΔG=36 kcal/mol) than in the case of other locked compounds **4** and **5**, with the dehydrogenation and re-aromatization step being similarly strongly exothermic (ca. 56 kcal/mol) compared to **4** (62 kcal/mol) and **5** (ca. 56 kcal/mol). This is most certainly due to the presence of the additional phenanthrene ring in **6-PC** that is almost ideally planar and provides better aromatic stabilization in **6-PC**.

7.4.2 The MOs of the TPE derivatives

The molecular orbitals (MOs) of TPE derivatives in acetonitrile are calculated at M062X/6-311G (d) level using the Gaussian 16 package. [54]

In all intermediates, the central carbon-carbon bond is elongated to such an extent that on a case-by-case basis it can be considered to be either a somewhat elongated or an actual single bond (Figure S1), thus, effectively interrupting the conjugation of the π-system in the newly formed 4a,4b-dihydrophenanthrene-type ring in the respective intermediate.

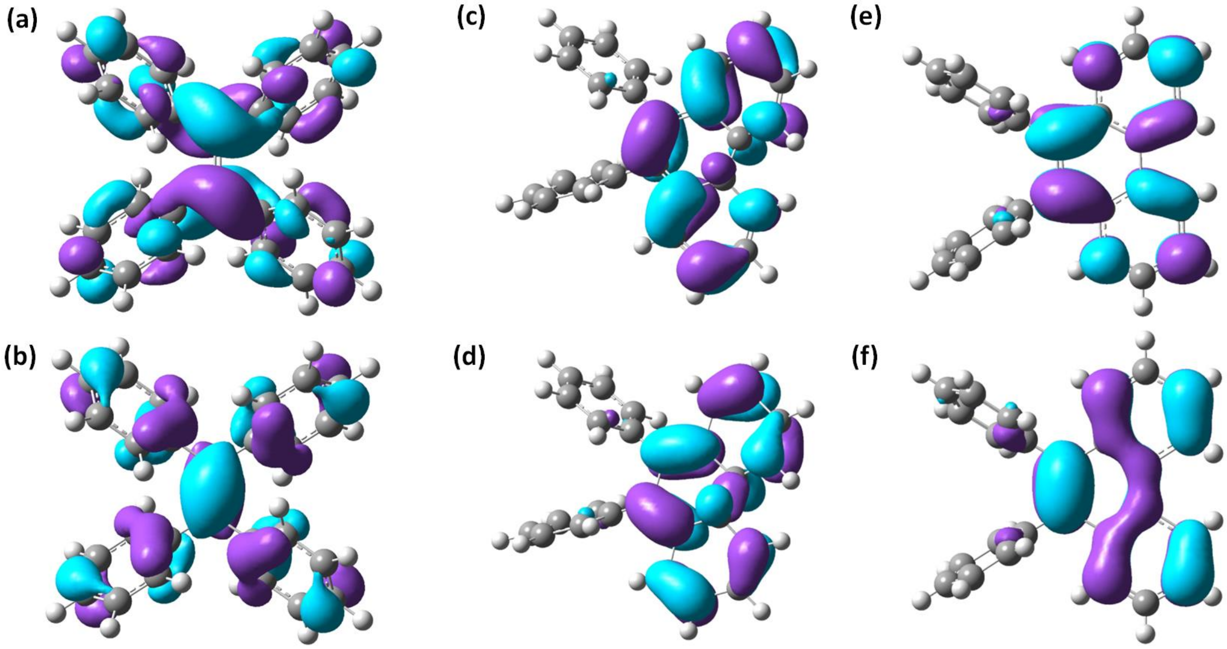

Figure S66. (a) LUMO and (b) HOMO of **1**, (c) LUMO and (d) HOMO of **1-IM**, (e) LUMO and (f) HOMO of **1-PC**; *iso* = 0.03.

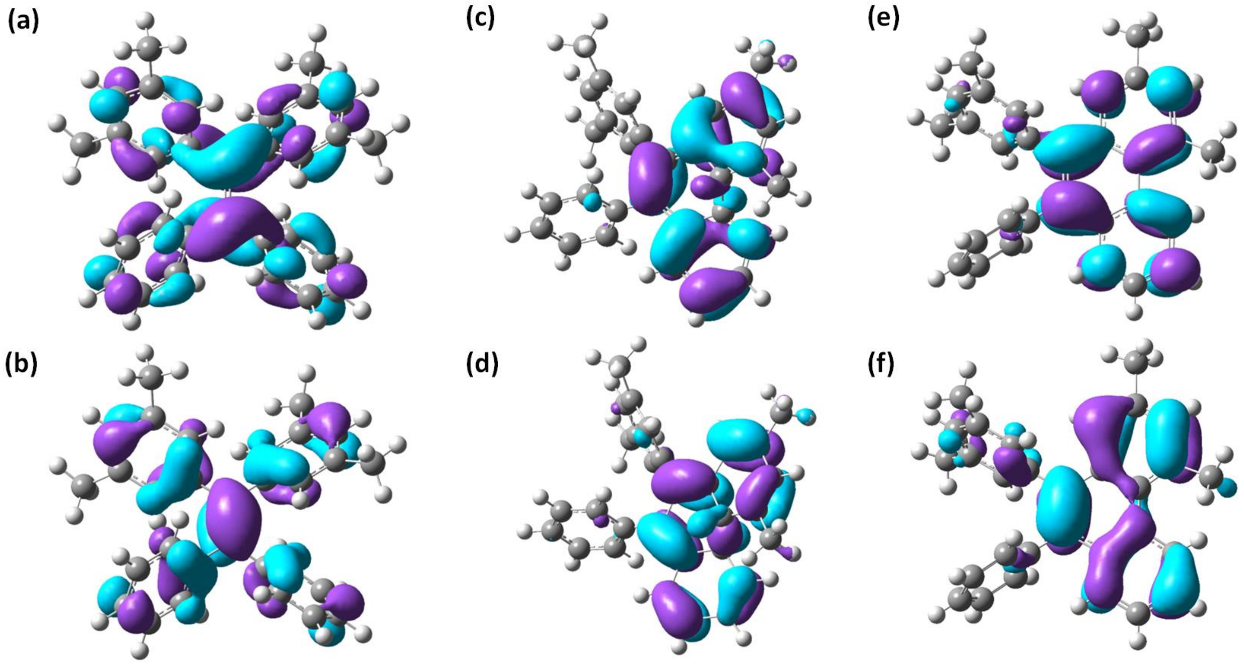

Figure S67. (a) LUMO and (b) HOMO of **2**, (c) LUMO and (d) HOMO of **2-IM**, (e) LUMO and (f) HOMO of **2-PC**; *iso* = 0.03.

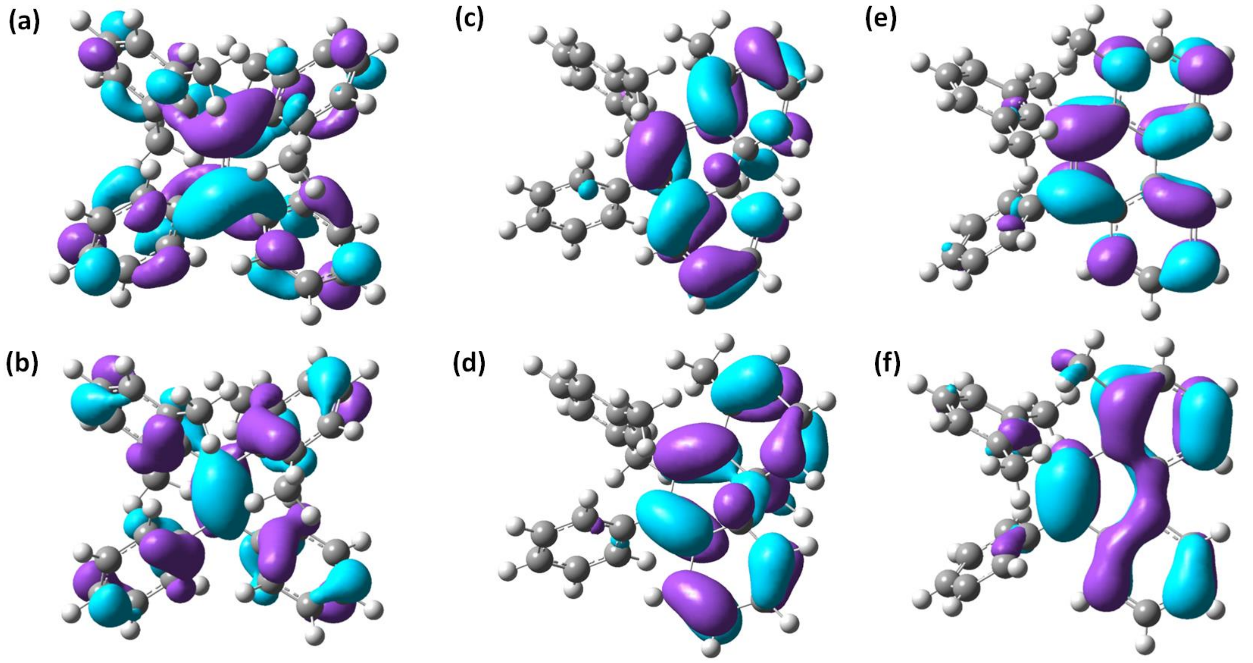

Figure S68. (a) LUMO and (b) HOMO of **3**, (c) LUMO and (d) HOMO of **3-IM**, (e) LUMO and (f) HOMO of **3-PC**; *iso* = 0.03.

HOMOs and LUMOs of the non-locked TPE derivatives **1-3** are delocalized and mainly comprised of the p-orbitals of the C=C bond with less significant yet substantial contributions from the π-system of the adjacent phenyl rings, demonstrating that a certain amount of stabilization for these systems can be attributed to the significantly conjugated and delocalized π-system present in these molecules. Obviously, HOMOs in **1-3** have a pronounced bonding character, while their LUMOs are strongly anti-bonding in nature, which is also well-reflected in the notably elongated carbon-carbon bond of the central TPE-moiety in the excited state of these molecules.

As two of the phenyl rings not involved in the photocyclization processes of the intermediates (**1-IM**, **2-IM** and **3-IM**) are essentially perpendicular, HOMOs and LUMOs of the intermediates reside almost exclusive on the newly formed 4a,4b-dihydrophenanthrene-type ring. Remarkably, HOMOs and LUMOs of the intermediates consist mainly of the π-system of the 4a,4b-dihydrophenanthrene-type ring with an additional contribution from the newly formed sigma C-C single bond for the HOMOs. This distribution of the frontier orbitals indicates a significant amount of conjugation still present in this type of molecules.

HOMOs and LUMOs in the photocyclized compounds (**1-PC**, **2-PC** and **3-PC**) resemble those in the intermediates in terms of their location, and those of the original non-locked compounds **1-3** in terms of their bonding/anti-bonding nature.

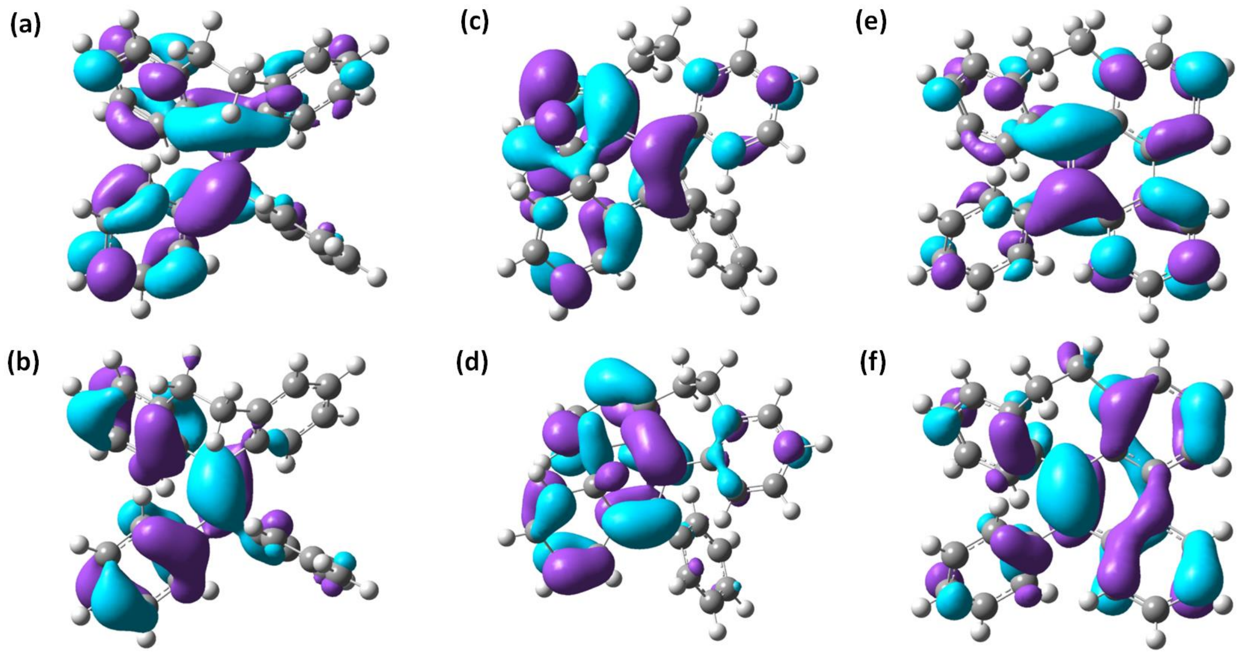

Figure S69. (a) LUMO and (b) HOMO of **4**, (c) LUMO and (d) HOMO of **4-IM**, (e) LUMO and (f) HOMO of **4-PC**; *iso* = 0.03.

In **4**, HOMO and LUMO are primarily located on the C=C bond and two phenyl rings (one locked and one non-locked) on the same side of the C=C bond. HOMO in **4** has a pronounced bonding character, while LUMO in **4** is clearly anti-bonding in its nature. Similarly, in the intermediate **4-IM**, HOMO and LUMO reside almost exclusively on the newly formed 4a,4b-dihydrophenanthrene-type ring with minor contributions from the phenyl ring on the locked side of the molecule to both HOMO and LUMO as well as the newly formed sigma C-C bond to LUMO. This distribution clearly reflects conjugation pattern of the π-system in the 4a,4b-dihydrophenanthrene-type ring. It is noteworthy that there is a partial shift of electron density within the 4a,4b-dihydrophenanthrene-type ring upon excitation (HOMO-LUMO transition), with the electron density being relocated from the π-system of the 4a,4b-dihydrophenanthrene-type ring into the σ-(C-C) framework of this ring. In contrast, after oxidative dehydrogenation of **4-IM** and formation of **4-PC**, a well-conjugated phenanthrene π-system is established with the adjacent phenyl rings connected to it being, at least partially, part of it. This is well reflected in the wide spread HOMO and LUMO in **4-PC** encompassing almost the whole entirety of the molecule, with HOMO and LUMO being bonding and anti-bonding in nature, respectively.

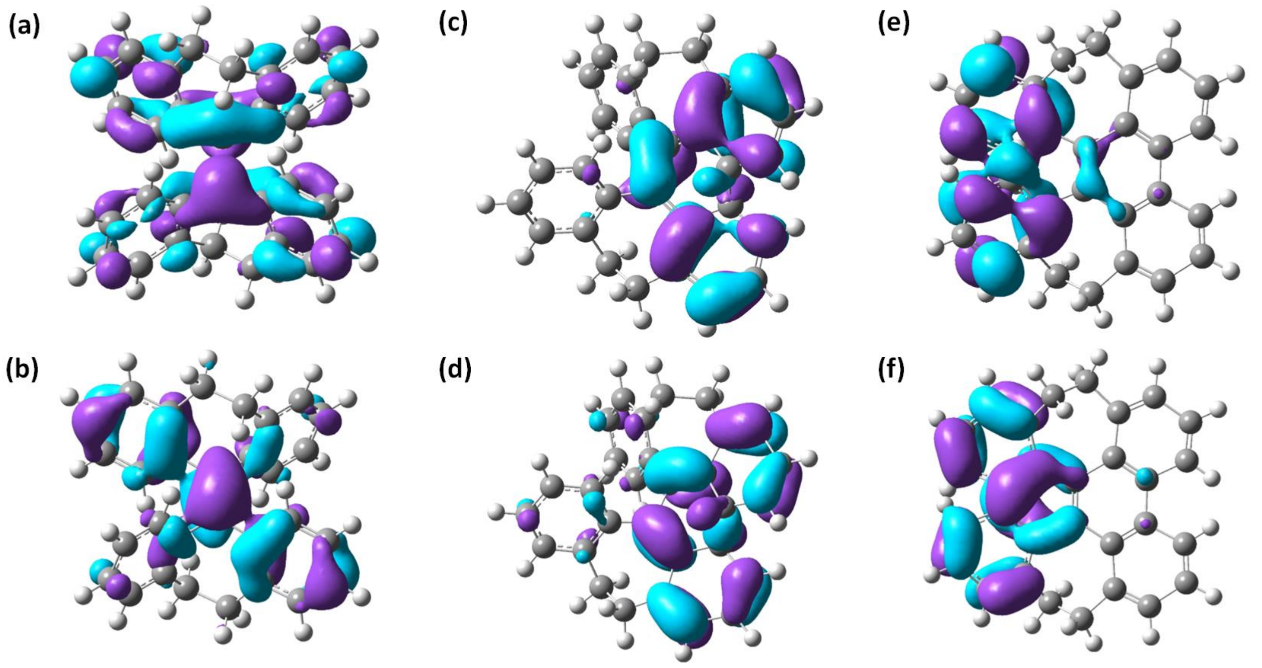

Figure S70. (a) LUMO and (b) HOMO of **5**, (c) LUMO and (d) HOMO of **5-IM**, (e) LUMO and (f) HOMO of **5-PC**; *iso* = 0.03.

In contrast to **4**, HOMO and LUMO in **5** are strongly delocalized over the whole π-system of the molecule. HOMO in **5** has a pronounced bonding character, while LUMO in **5** is clearly anti-bonding in its nature. In contrast to **5**, HOMO and LUMO in the intermediate **5-IM** are located almost exclusively on the newly formed 4a,4b-dihydrophenanthrene-type ring with negligible contributions from the phenyl rings not involved in the ring formation process. While this distribution clearly reflects conjugation pattern of the π-system in the 4a,4b-dihydrophenanthrene-type ring of **5-IM**, we note that HOMO of **5-IM** also has significant contributions coming from the newly formed σ(C-C) bond in the 4a,4b-dihydrophenanthrene-type ring and *antiperiplanar* C-H bonds in 4a,4b-positions of the same ring. The latter contribute to the LUMO in **5-IM** as well. Similar to **4-IM**, the electron density is relocated from the π-system of the 4a,4b-dihydrophenanthrene-type ring into the σ-(C-C) framework of this ring upon excitation. Interestingly, after oxidative dehydrogenation of **5-IM**, a somewhat perturbed but reasonably well-conjugated phenanthrene π-system of **5-PC** is established, but unlike in case of **4-PC** and **6-PC**, the frontier orbitals in **5-PC** remain essentially localized on the phenanthrene ring, which is almost certainly a consequence of the significantly distorted geometry of **5-PC** that does not allow for planarization and more for effective conjugation in it. HOMO and LUMO in **5-PC** have bonding and anti-bonding character, respectively.

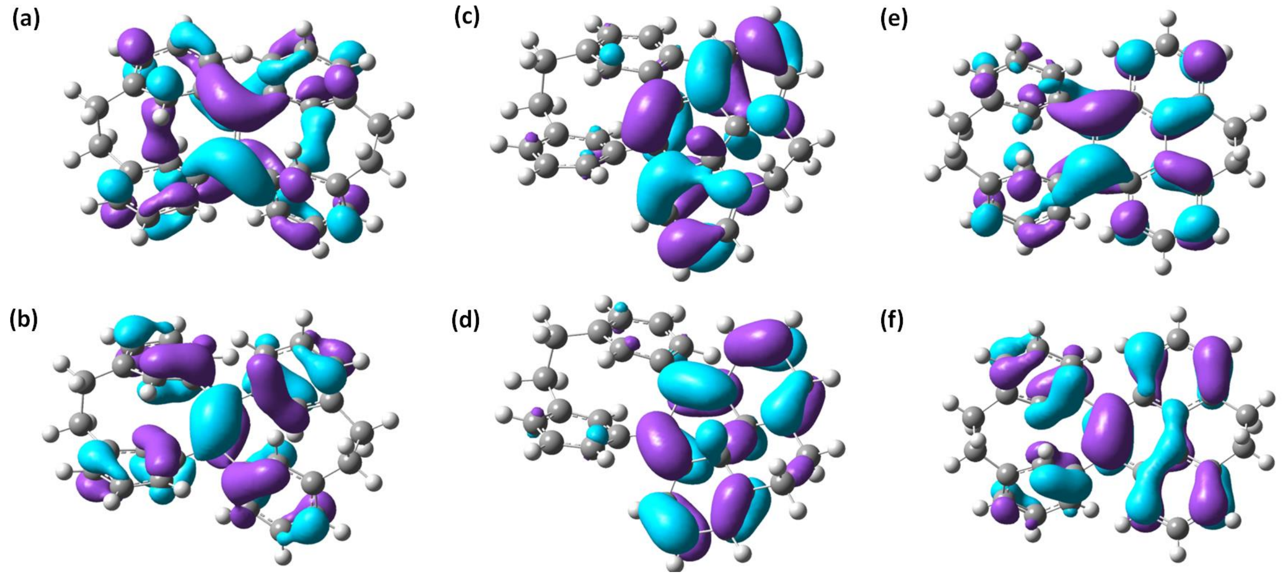

Figure S71. (a) LUMO and (b) HOMO of **6**, (c) LUMO and (d) HOMO of **6-IM**, (e) LUMO and (f) HOMO of **6-PC**; *iso* = 0.03.

In **6**, HOMO and LUMO are strongly delocalized over the whole π-system of the molecule. HOMO in **6** has a pronounced bonding character, while LUMO in **6** is clearly anti-bonding in its nature. Remarkably, due to the spatial proximity there is a significant overlap between the MOs located on the carbon atoms that form the new σ(C-C) bond of the 4a,4b-dihydrophenanthrene-type ring in LUMO of **6**. In contrast, in the intermediate **6-IM**, HOMO and LUMO reside almost exclusively on the newly formed 4a,4b-dihydrophenanthrene-type ring with negligible contributions from the phenyl rings not involved in the ring formation process. While this distribution clearly reflects conjugation pattern of the π-system in the 4a,4b-dihydrophenanthrene-type ring, it is noteworthy that HOMO of **6-IM** also has significant contributions coming from the σ(C-C) bonds of the ethylene (-CH_2-CH_2-) bridge as well as the newly formed C-C bond in 4a,4b-dihydrophenanthrene-type ring. Peculiarly, C-H bonds in 4a,4b-positions of the dihydrophenanthrene ring contribute significantly to LUMO in **6-IM**. Similar to **4-IM**, the electron density is relocated from the π-system of the 4a,4b-dihydrophenanthrene-type ring into the σ-(C-C) framework of this ring upon excitation. In contrast, after oxidative dehydrogenation of **6-IM** and formation of **6-PC**, a well-conjugated phenanthrene π-system is established with the adjacent phenyl rings connected to it being, at least partially, part of it. HOMO and LUMO in **6-PC** are delocalized all over the π-system of the molecule with HOMO and LUMO being bonding and anti-bonding in nature, respectively.

7.5 The geometry changes of TPE derivatives at S_0 and S_1 in solution and in solid state

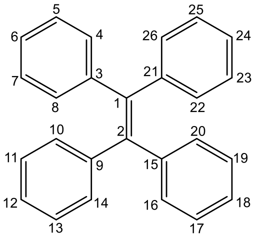

1

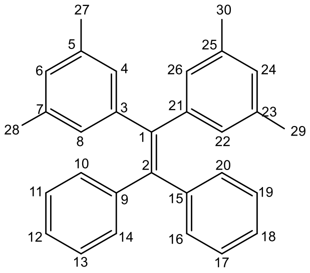

2

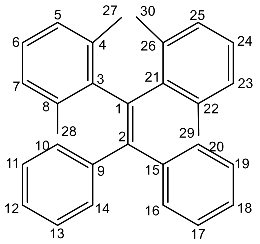

3

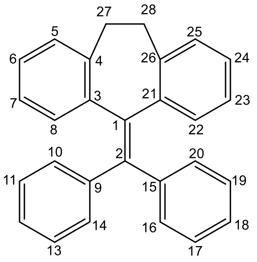

4

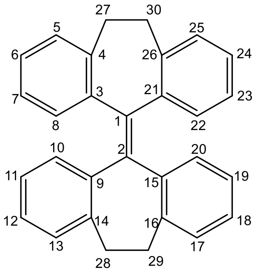

5

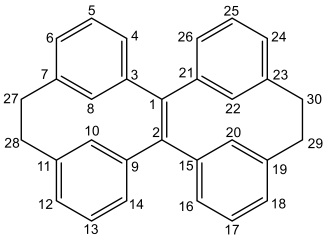

6

Table S10. The metric parameters for **1** at the ground state (S_0), first excited state (S_1), and the corresponding structural difference Δ (S_1-S_0) in both THF solution and solid state obtained at the M062X/6-311G(d) level using the Gaussian 09 package.[33] Bond lengths (*d*) are given in Å, angles in °.

1	in THF solution			in solid state			Crystal structure [55]
Structural parameters	S_0	S_1	Δ (S_1-S_0)	S_0	S_1	Δ (S_1-S_0)	
***d*(C1-C2)**	**1.351**	**1.470**	**0.119**	**1.351**	**1.445**	**0.094**	**1.356**
d(C1-C3)	1.492	1.442	-0.050	1.491	1.454	-0.037	1.492
d(C2-C9)	1.492	1.442	-0.050	1.491	1.450	-0.041	1.496
***d*(C2-C15)**	**1.492**	**1.442**	**-0.050**	**1.486**	**1.440**	**-0.046**	1.503
d(C1-C21)	1.492	1.442	-0.050	1.489	1.433	-0.056	1.491
**∠ (C3-C1-C21)**	**114.585**	**123.765**	**9.180**	113.713	118.755	**5.042**	115.368
**∠(C9-C2-C15)**	**114.585**	**123.767**	**9.182**	115.209	118.118	**2.909**	113.905
∠ (C3-C1-C2)	122.707	118.110	-4.597	121.245	119.942	-1.303	123.012
∠(C21-C1-C2)	122.708	118.125	-4.583	124.984	121.164	-3.820	121.581
∠(C9-C2-C1)	122.708	118.123	-4.585	121.463	120.973	-0.490	124.975
∠(C15-C2-C1)	122.708	118.110	-4.598	123.270	120.898	-2.372	121.103
τ (C4-C3-C1-C2)	-130.384	-153.545	-23.161	-124.248	-129.590	-5.342	-138.074
τ (C26-C21-C1-C2)	-130.388	-153.505	-23.117	-134.126	-148.559	-14.433	-132.750
τ (C10-C9-C2-C1)	-130.387	-153.508	-23.121	-131.891	-139.497	-7.606	-137.781
τ (C16-C15-C2-C1)	-130.386	-153.552	-23.166	-140.294	-138.682	1.612	-123.786
***τ* (C8-C3-C1-C2)**	**50.921**	**21.878**	**-29.043**	56.911	42.361	**-14.550**	45.792
***τ* (C22-C21-C1-C2)**	**50.917**	**21.901**	**-29.016**	47.949	26.507	**-21.442**	47.489
***τ* (C14-C9-C2-C1)**	**50.918**	**21.903**	**-29.015**	47.495	34.777	**-12.718**	45.002
***τ* (C20-C15-C2-C1)**	**50.919**	**21.878**	**-29.041**	43.104	31.830	**-11.274**	55.877
τ (C3-C1-C2-C9)	5.923	62.726	56.803	7.051	18.836	11.785	8.721
τ (C3-C1-C2-C15)	-174.077	-117.241	56.836	-170.032	-162.377	7.655	-169.669

τ (C21-C1-C2-C9)	-174.077	-117.306	56.771	-175.904	-156.824	19.080	-173.655
**τ (C21-C1-C2-C15)**	**5.923**	**62.727**	**56.804**	7.014	21.963	**14.949**	7.955
**plane angle between C3-C1-C21 and C9-C2-C15**	**5.923**	**62.725**	**56.802**	8.425	20.865	**12.440**	8.925
***d*(C20-C22)**	**3.250**	**3.635**	**0.385**	**3.168**	**2.820**	**-0.348**	3.246
d(C8-C10)	3.851	3.989	0.138	3.829	3.807	-0.022	3.978
d(C20-C26)	3.851	3.989	0.138	3.889	3.879	-0.010	3.846
d(C16-C22)	3.851	3.991	0.140	4.089	3.661	-0.428	3.614
d(C16-C26)	5.315	5.467	0.152	5.470	5.427	-0.043	5.200
d(C4-C10)	5.315	5.467	0.152	5.192	5.257	0.065	5.494
d(C4-C14)	3.851	3.991	0.140	3.613	3.601	-0.012	4.020
***d*(C8-C14)**	**3.250**	**3.635**	**0.385**	**3.234**	**3.070**	**-0.164**	3.204

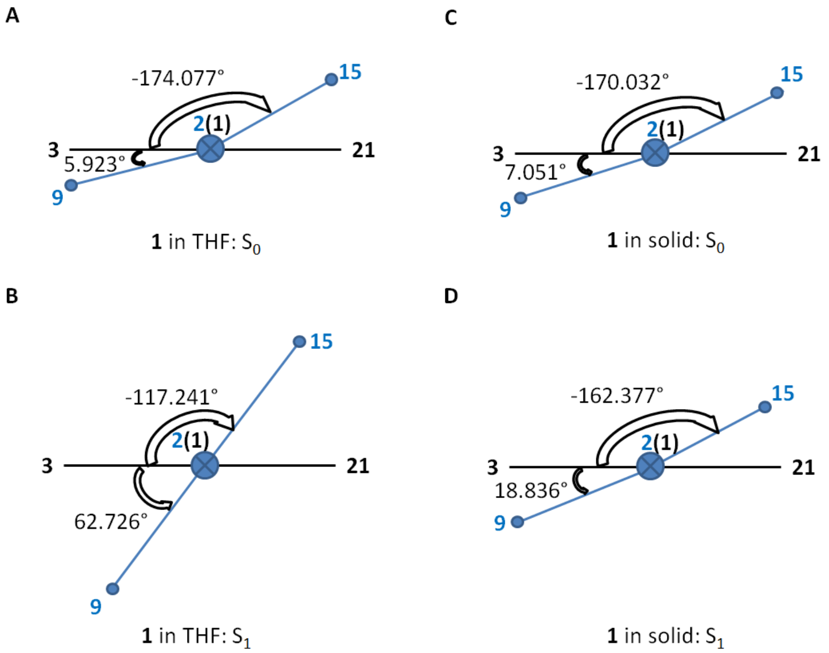

Figure S72. The changes in the dihedral angels τ (C3-C1-C2-C9) and τ (C3-C1-C2-C15) of **1** in solution (THF) and in solid state, at S_0 and S_1, respectively, with molecules looked upon along the C2-C1 bond with C2 as the proximal carbon atom are shown.

Detailed list of the major structural parameters of **1** in solution and solid phase both in ground (S_0) and excited (S_1) states is shown in Table S10. The Δ (S_1-S_0) of each parameter is used to illustrate the structural change after **1**'s excitation in both solution/solid.

In the ground state, the four phenyl groups are individually connected to C1 and C2 through the C-C single bonds with nearly identical bond lengths of 1.492 Å in solution. And the C1-C2 bond is a typical ethylenic double bond with a bond length of 1.351 Å. The four non-substituted phenyl rings have the same orientation character, which results in an approximate D_2 symmetry of the molecule with τ (C8-C3-C1-C2) = τ (C22-C21-C1-C2) = τ (C14-C9-C2-C1) = τ (C20-C15-C2-C1) = 50.9°.

Upon an electronic excitation, the C1-C2 bond is significantly elongated from 1.351 Å to 1.470 Å, while the bonds connecting the ethylenic C1-C2 bond with the peripheral four phenyl groups in S_1 are markedly shortened from 1.492 Å to 1.442 Å, indicating that a better conjugation around the central C-C bond in the molecule is established upon **1**'s excitation. Similar changes in these bond lengths can be

seen in the other TPE derivatives as well. [56] With these bond lengths changing at the molecular center, the angles ∠ (C3-C1-C21) and ∠(C9-C2-C15) become larger, too, increasing by more than 9°. Furthermore, the distances between the carbon atoms of the neighboring phenyl rings *d* (C20-C22) and *d* (C8-C14), which can potentially facilitate the formation of the dihydrophenanthrene intermediate, become larger due to the increased amplitude of the C1-C2 bond twisting motion after its elongation. This is likely to increase the time span for phenyl torsion to find the specific configuration to form the intermediate. The biggest configuration change after excitation is the increase of the twisting angles (*e.g.* τ (C21-C1-C2-C15)) of the ethylenic C1-C2 bond by 56.8°, which is coupled with a big increase of the torsion of the phenyl rings by 29.0°, i.e. τ (C20-C15-C2-C1). At this point, it is clear that the electronic excitation magnifies the nonplanar character of the molecular geometry, as also can be seen from the Figure S72.

While **1** undergoes similar structural changes upon excitation in the solid state and solution, the amplitude of these changes in the solid is significantly smaller, with the only difference being the distances *d*(C20-C22) and *d*(C8-C14) becoming much shorter upon excitation in the solid state, as compared to their change in solution, indicating that the molecular motion is notably restricted in the solid state, especially for the twisting mode of the C1-C2 bond and torsion of phenyl rings (Figure S72).

Table S11. The metric parameters for **2** at the ground state (S_0), first excited state (S_1), and the corresponding structural difference △ (S_1-S_0) in both THF solution and solid state obtained at the M062X/6-311G(d) level using the Gaussian 09 package.[33] Bond lengths (*d*) are given in Å, angles in °.

2	in THF solution			in solid state			Crystal structure
Structural parameters	S_0	S_1	△ (S_1-S_0)	S_0	S_1	△ (S_1-S_0)	
***d* (C1-C2)**	**1.354**	**1.468**	**0.114**	**1.351**	**1.441**	**0.090**	1.355
d (C1-C3)	1.491	1.444	-0.047	1.492	1.443	-0.049	1.493
d (C2-C9)	1.491	1.443	-0.048	1.492	1.442	-0.050	1.497
***d* (C2-C15)**	**1.491**	**1.443**	**-0.048**	**1.496**	**1.460**	**-0.036**	1.500
d (C1-C21)	1.491	1.444	-0.047	1.494	1.454	-0.040	1.498
**∠ (C3-C1-C21)**	**115.080**	**122.432**	**7.352**	115.784	118.407	**2.623**	116.019
**∠ (C9-C2-C15)**	**114.981**	**122.425**	**7.444**	112.530	115.151	**2.621**	112.629
∠ (C3-C1-C2)	122.460	118.784	-3.676	123.459	121.823	-1.636	123.291
∠ (C21-C1-C2)	122.460	118.784	-3.676	120.752	119.716	-1.036	120.691
∠ (C9-C2-C1)	122.509	118.790	-3.719	124.614	123.581	-1.033	124.551
∠ (C15-C2-C1)	122.509	118.785	-3.724	122.856	121.266	-1.590	122.831
d (C5-C27)	1.507	1.507	0.000	1.508	1.508	0.000	1.510
d (C7-C28)	1.507	1.508	0.001	1.508	1.509	0.001	1.514
d (C25-C30)	1.507	1.507	0.000	1.507	1.507	0.000	1.508
d (C23-C29)	1.507	1.508	0.001	1.506	1.506	0.000	1.511
τ (C4-C3-C1-C2)	132.531	151.782	19.251	138.959	144.543	5.584	140.901
τ (C26-C21-C1-C2)	132.531	151.776	19.245	130.475	134.467	3.992	131.901
**τ (C8-C3-C1-C2)**	**-48.547**	**-23.285**	**25.262**	-42.997	-31.718	**11.279**	-40.720
**τ (C22-C21-C1-C2)**	**-48.547**	**-23.285**	**25.262**	-48.429	-38.911	**9.518**	-46.662
**τ (C10-C9-C2-C1)**	**-48.542**	**-23.381**	**25.161**	-58.855	-40.049	**18.806**	-60.052
**τ (C20-C15-C2-C1)**	**-48.542**	**-23.355**	**25.187**	-61.305	-49.117	**12.188**	-60.992
τ (C14-C9-C2-C1)	132.779	151.622	18.843	125.005	135.571	10.566	123.401
τ (C16-C15-C2-C1)	132.779	151.646	18.867	120.293	126.786	6.493	120.391
τ (C3-C1-C2-C9)	-9.456	-49.052	-39.596	-5.193	-15.984	-10.791	-6.462

τ (C3-C1-C2-C15)	170.544	130.940	-39.604	174.961	164.569	-10.392	173.371
τ (C21-C1-C2-C9)	170.544	130.962	-39.582	175.642	166.733	-8.909	174.401
***τ* (C21-C1-C2-C15)**	**-9.456**	**-49.047**	**-39.591**	-4.204	-12.714	**-8.510**	-5.772
**plane angle between C3-C1-C21 and C9-C2-C15**	**9.457**	**49.048**	**39.591**	4.706	14.390	**9.684**	6.156
***d*(C20-C22)**	**3.228**	**3.308**	**0.080**	**3.293**	**3.113**	**-0.180**	3.297
***d*(C8-C10)**	**3.228**	**3.309**	**0.081**	**3.322**	**3.043**	**-0.279**	3.329
d(C20-C26)	3.828	3.862	0.034	3.940	3.864	-0.076	3.956
d(C16-C22)	3.829	3.861	0.032	3.582	3.574	-0.008	3.564
d(C16-C26)	5.322	5.441	0.119	5.193	5.236	0.043	5.195
d(C4-C10)	3.828	3.863	0.035	4.181	4.074	-0.107	4.222
d(C4-C14)	5.322	5.441	0.119	5.395	5.477	0.082	5.387
d(C8-C14)	3.829	3.861	0.032	3.777	3.803	0.026	3.719

The changes in the dihedral angels *τ* (C3-C1-C2-C9) and *τ* (C3-C1-C2-C15) of **2** in solution (THF) and in solid state, at S_0 and S_1, respectively, with molecules looked upon along the C2-C1 bond with C2 as the proximal carbon atom are shown below.

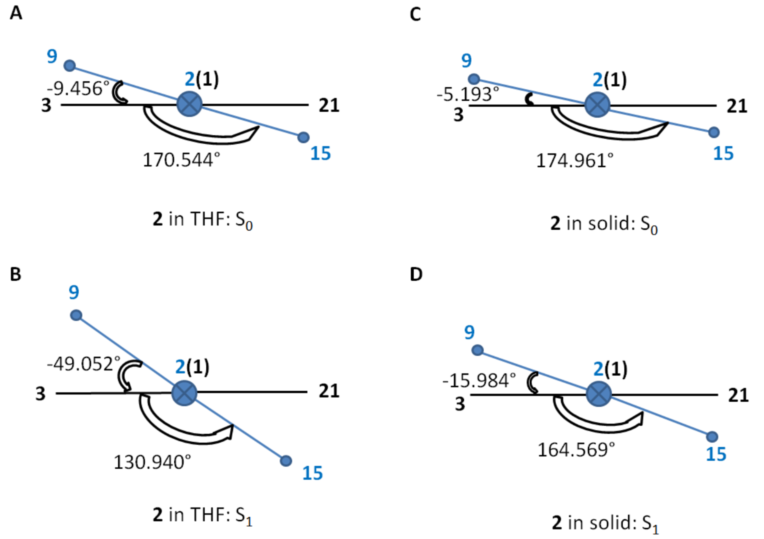

Figure S73. The changes in the dihedral angels *τ* (C3-C1-C2-C9) and *τ* (C3-C1-C2-C15) of **2** in solution (THF) and in solid state, at S_0 and S_1, respectively, with molecules looked upon along the C2-C1 bond with C2 as the proximal carbon atom are shown.

In solution in the ground state (S_0), **2** has identical bond lengths of 1.491 Å for the C-C single bonds connecting the four phenyl groups to the typical ethylenic C1=C2 double bond with a bond length of 1.354 Å. The four phenyl rings result in an approximate symmetry in the molecule with dihedral angles *τ* (C8-C3-C1-C2) = *τ* (C22-C21-C1-C2) = *τ* (C10-C9-C2-C1) = *τ* (C20-C15-C2-C1) = -48.5°, which are smaller than those of **1**. Thus, except for the intrinsically enhanced rigidity of the structure introduced by the *m*-position methyl substituted phenyl rings, they have no big influence on the geometry changes as compared to **1** in terms of a S_1-S_0 comparison.

Upon an electronic excitation, similar changes in the central C-C bond lengths can be seen in **2** as observed in **1**, indicating a better conjugation around the central C-C bond in the molecule is established upon **2**'s excitation. With these bond lengths changing at the molecular center, the angles ∠ (C3-C1-

C21) and ∠(C9-C2-C15) become wider, too, increasing by more than 7°, which is smaller than those in **1**. The biggest configuration change after excitation is the increase of the twisting angles (*e.g.* τ (C21-C1-C2-C15)) of the ethylenic C1-C2 bond by 39.6° which is coupled with a big increase of the torsional angel of the phenyl ring by 25.2°, *i.e.* τ (C20-C15-C2-C1). These angles are smaller than those in **1**, which is probably due to the more rigid structure caused by the *m*-position substituted phenyl rings. Furthermore, the distances of two atoms in the phenyl rings, *d* (C20-C22) and *d* (C8-C10), become larger due to the increased amplitude of the C1-C2 twisting motion after its elongation.

While **2** undergoes similar structural changes upon excitation in the solid state and solution, the amplitude of these changes in the solid is significantly smaller (Figure S73).

Table S12. The metric parameters for **3** at the ground state (S_0), first excited state (S_1), and the corresponding structural difference ∆ (S_1-S_0) in both THF solution and solid state obtained at the M062X/6-311G(d) level using the Gaussian 09 package.[33] Bond lengths (*d*) are given in Å, angles in °.

3	in THF solution			in solid state			Crystal structure
Structural parameters	S_0	S_1	∆ (S_1-S_0)	S_0	S_1	∆ (S_1-S_0)	
***d* (C1-C2)**	**1.355**	**1.461**	**0.106**	**1.357**	**1.451**	**0.094**	1.357
d (C1-C3)	1.502	1.453	-0.049	1.502	1.459	-0.043	1.502
d (C2-C9)	1.490	1.446	-0.044	1.492	1.452	-0.040	1.493
***d* (C2-C15)**	**1.490**	**1.446**	**-0.044**	**1.490**	**1.445**	**-0.045**	1.487
d (C1-C21)	1.502	1.453	-0.049	1.499	1.454	-0.045	1.507
**∠ (C3-C1-C21)**	**115.336**	**120.860**	**5.524**	117.437	119.652	**2.215**	116.798
**∠ (C9-C2-C15)**	**115.236**	**121.975**	**6.739**	116.485	120.626	**4.141**	116.068
∠ (C3-C1-C2)	122.329	119.571	-2.758	120.819	119.181	-1.638	121.241
∠ (C21-C1-C2)	122.334	119.569	-2.765	121.739	121.163	-0.576	121.971
∠ (C9-C2-C1)	122.382	119.013	-3.369	120.861	118.750	-2.111	120.529
∠ (C15-C2-C1)	122.382	119.012	-3.370	122.608	120.590	-2.018	123.391
d (C4-C27)	1.510	1.509	-0.001	1.507	1.509	0.002	1.501
d (C8-C28)	1.510	1.507	-0.003	1.508	1.505	-0.003	1.506
d (C26-C30)	1.510	1.509	-0.001	1.510	1.511	0.001	1.506
d (C22-C29)	1.510	1.507	-0.003	1.509	1.507	-0.002	1.503
τ (C4-C3-C1-C2)	125.082	135.440	10.358	123.478	128.007	4.529	122.201
τ (C26-C21-C1-C2)	125.093	135.433	10.340	122.274	127.926	5.652	122.221
**τ (C8-C3-C1-C2)**	**-57.672**	**-41.345**	**16.327**	-56.901	-46.537	**10.364**	-57.731
**τ (C22-C21-C1-C2)**	**-57.667**	**-41.345**	**16.322**	-58.505	-46.396	**12.109**	-60.011
**τ (C10-C9-C2-C1)**	**-44.268**	**-23.488**	**20.780**	-43.750	-32.046	**11.704**	-45.182
**τ (C20-C15-C2-C1)**	**-44.267**	**-23.494**	**20.773**	-41.312	-23.960	**17.352**	-39.852
τ (C14-C9-C2-C1)	136.504	152.293	15.789	133.434	140.083	6.649	132.581
τ (C16-C15-C2-C1)	136.509	152.287	15.778	141.410	150.557	9.147	142.801
τ (C3-C1-C2-C9)	-9.084	-43.046	-33.962	-11.926	-26.562	-14.636	-11.182
τ (C3-C1-C2-C15)	170.916	136.955	-33.961	165.536	151.331	-14.205	166.871
τ (C21-C1-C2-C9)	170.913	136.954	-33.959	168.845	154.175	-14.670	169.209
**τ (C21-C1-C2-C15)**	**-9.087**	**-43.045**	**-33.958**	-13.693	-27.932	**-14.239**	-12.752
**plane angle between C3-C1-C21 and C9-C2-C15**	**9.085**	**43.044**	**33.959**	13.049	27.331	**14.282**	12.065
***d*(C20-C22)**	**3.315**	**3.488**	**0.173**	**3.381**	**3.234**	**-0.147**	3.389
***d*(C8-C10)**	**3.315**	**3.488**	**0.173**	**3.266**	**3.230**	**-0.036**	3.294
d(C20-C26)	3.665	3.571	-0.094	3.570	3.503	-0.067	3.595
d(C16-C22)	4.008	4.097	0.089	4.067	4.104	0.037	4.139

d(C16-C26)	5.295	5.307	0.012	5.253	5.317	0.064	5.293
d(C4-C10)	3.665	3.571	-0.094	3.510	3.421	-0.089	3.513
d(C4-C14)	5.295	5.307	0.012	5.154	5.147	-0.007	5.141
d(C8-C14)	4.008	4.097	0.089	3.828	3.811	-0.017	3.836

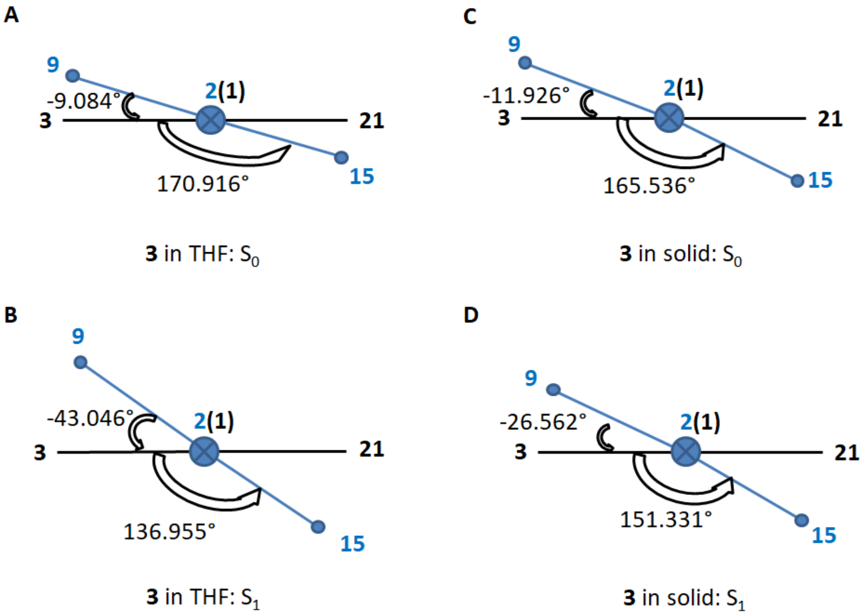

Figure S74. The changes in the dihedral angels *τ* (C3-C1-C2-C9) and *τ* (C3-C1-C2-C15) of **3** in solution (THF) and in solid state, at S_0 and S_1, respectively, with molecules looked upon along the C2-C1 bond with C2 as the proximal carbon atom are shown.

In solution, upon excitation **3** displays geometry changes similar to the ones predicted for **1** and **2** with the difference being that in **3** the changes in dihedral angles of phenyl torsion are smaller: $\Delta\tau$ (C8-C3-C1-C2) = $\Delta\tau$ (C22-C21-C1-C2) = 16.3° and $\Delta\tau$ (C10-C9-C2-C1) = $\Delta\tau$ (C20-C15-C2-C1) = 20.8°, which is indicative of the out of sync torsion of the four phenyl rings due to the steric hindrance caused by the methyl groups in the *o*-positions of two phenyl rings. Similar bond lengths' changes can be found in the central C1-C2 bond in the molecule. Similar to **1** and **2**, the changes in the distances *d* (C20-C22) and *d* (C8-C10) become larger. However, the ∠(C3-C1-C21) and ∠(C9-C2-C15) angles increased by less than 7°, and the change of the C1-C2 twisting angle (*e.g.* *τ* (C21-C1-C2-C15)) is almost 34°, indicating a more rigid structure as compared to **1** and **2**.

While **3** undergoes similar structural changes upon excitation in the solid state and solution, the amplitude of these changes in the solid is significantly smaller (Figure S74).

Table S13. The metric parameters for **4** at the ground state (S_0), first excited state (S_1), and the corresponding structural difference ∆ (S_1-S_0) in both THF solution and solid state obtained at the M062X/6-311G(d) level using the Gaussian 09 package.[33] Bond lengths (*d*) are given in Å, angles in °.

**4**	in THF solution	in solid state	Crystal structure
Structural parameters	S_0	S_0	
d (C1-C2)	1.346	1.348	1.359
d (C1-C3)	1.495	1.498	1.525
d (C1-C21)	1.493	1.492	1.512
d (C2-C9)	1.492	1.502	1.505
d (C2-C15)	1.497	1.497	1.518
∠ (C3-C1-C21)	114.784	113.954	119.300
∠ (C9-C2-C15)	115.064	116.656	115.430
∠ (C3-C1-C2)	123.530	121.837	121.200
∠ (C21-C1-C2)	121.684	124.197	120.014
∠ (C9-C2-C1)	124.013	119.382	121.030
∠ (C15-C2-C1)	120.835	123.771	123.430
d (C4-C27)	1.522	1.518	1.493
d (C27-C28)	1.532	1.530	1.542
d (C26-C28)	1.505	1.505	1.492
τ (C3-C4-C27-C28)	-2.673	2.313	6.300
τ (C4-C27-C28-C26)	60.939	54.738	55.018
τ (C27-C28-C26-C21)	-72.408	-73.128	-77.300
∠ (C3-C4-C27)	126.066	126.198	126.300
∠ (C4-C27-C28)	118.366	118.881	119.113
∠ (C27-C28-C26)	111.839	113.431	114.512
∠ (C28-C26-C21)	118.463	118.572	118.115
τ (C4-C3-C1-C2)	127.625	124.146	132.300
τ (C8-C3-C1-C2)	-55.057	-58.232	-58.400
τ (C26-C21-C1-C2)	-109.670	-112.265	-121.200
τ (C22-C21-C1-C2)	71.720	68.800	75.300
τ (C10-C9-C2-C1)	137.766	110.766	107.440
τ (C14-C9-C2-C1)	-44.371	-68.424	-73.850
τ (C16-C15-C2-C1)	-87.720	-140.069	-138.840
τ (C20-C15-C2-C1)	93.580	40.772	43.250
τ (C3-C1-C2-C9)	-6.724	-7.221	-7.200
τ (C3-C1-C2-C15)	176.864	177.984	178.200
τ (C21-C1-C2-C9)	173.840	174.134	173.111
τ (C21-C1-C2-C15)	-2.571	-0.661	-1.411
plane angle between C3-C1-C21 and C9-C2-C15	5.113	5.255	6.112
d(C20-C22)	4.073	3.327	3.343
d(C8-C10)	3.998	3.422	3.379
d(C20-C26)	4.023	3.579	3.632
d(C16-C22)	3.317	4.324	4.283
d(C16-C26)	4.633	5.308	5.322
d(C4-C10)	5.402	4.941	5.025
d(C4-C14)	3.802	3.806	4.086
d(C8-C14)	3.256	3.579	3.687

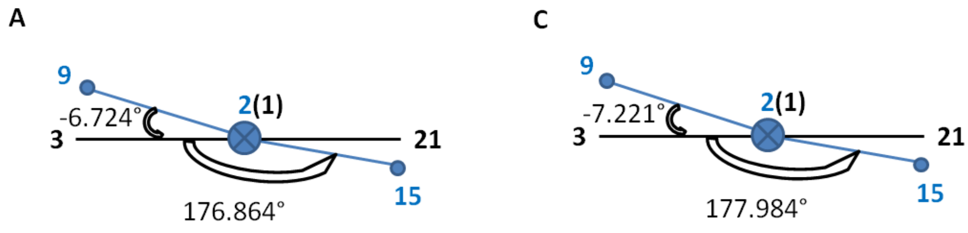

4 in THF: S_0 **4** in solid: S_0

Figure S75. The changes in the dihedral angels τ (C3-C1-C2-C9) and τ (C3-C1-C2-C15) of **4** in solution (THF) and in solid state, at S_0 and S_1, respectively, with molecules looked upon along the C2-C1 bond with C2 as the proximal carbon atom are shown.

The excited state calculations of **4** in both solution and solid state did not converge. Thus, the geometry changes in **4** upon excitation and comparison to **1-3** in terms of Δ (S_1-S_0) could not be studied.

Table S14. The metric parameters for **5** at the ground state (S_0), first excited state (S_1), and the corresponding structural difference △ (S_1-S_0) in both THF solution and solid state obtained at the M062X/6-311G(d) level using the Gaussian 09 package.[33] Bond lengths (*d*) are given in Å, angles in °.

5	in THF solution			in solid state			Crystal structure[5]
Structural parameters	S_0	S_1	△ (S_1-S_0)	S_0	S_1	△ (S_1-S_0)	
***d* (C1-C2)**	**1.347**	**1.467**	**0.120**	**1.348**	**1.454**	**0.106**	1.347
d (C1-C3)	1.492	1.462	-0.030	1.494	1.459	-0.035	1.490
d (C2-C9)	1.498	1.453	-0.045	1.501	1.459	-0.042	1.491
***d* (C2-C15)**	**1.492**	**1.462**	**-0.030**	**1.494**	**1.459**	**-0.035**	1.490
d (C1-C21)	1.498	1.452	-0.046	1.501	1.459	-0.042	1.491
**∠ (C3-C1-C21)**	**114.955**	**117.222**	**2.267**	114.264	116.546	**2.282**	114.159
**∠ (C9-C2-C15)**	**114.955**	**117.222**	**2.267**	114.264	116.546	**2.282**	114.159
∠ (C3-C1-C2)	122.183	119.549	-2.634	122.722	120.444	-2.278	122.631
∠ (C21-C1-C2)	122.767	121.524	-1.243	122.914	120.942	-1.972	123.031
∠ (C9-C2-C1)	122.767	121.524	-1.243	122.913	120.941	-1.972	123.031
∠ (C15-C2-C1)	122.183	119.550	-2.633	122.723	120.443	-2.280	122.631
d (C4-C27)	1.506	1.500	-0.006	1.503	1.499	-0.004	1.612
d (C27-C30)	1.530	1.524	-0.006	1.524	1.520	-0.004	1.436
d (C26-C30)	1.521	1.518	-0.003	1.517	1.515	-0.002	1.555
d (C14-C28)	1.521	1.518	-0.003	1.517	1.515	-0.002	1.555
d (C28-C29)	1.530	1.524	-0.006	1.524	1.520	-0.004	1.436
d (C16-C29)	1.506	1.500	-0.006	1.503	1.499	-0.004	1.612
τ (C4-C27-C30-C26)	-60.165	-60.262	-0.097	-58.227	-56.109	2.118	-56.212
τ (C14-C28-C29-C16)	60.165	60.260	0.095	58.232	56.112	-2.120	56.212
∠ (C3-C4-C27)	118.453	118.189	-0.264	118.118	117.641	-0.477	116.730
∠ (C21-C26-C30)	126.338	126.317	-0.021	126.774	126.580	-0.194	127.950
∠ (C9-C14-C28)	126.338	126.317	-0.021	126.773	126.578	-0.195	127.950
∠ (C29-C16-C15)	118.453	118.189	-0.264	118.120	117.642	-0.478	116.730
τ (C3-C4-C27-C30)	72.992	75.209	2.217	75.642	77.850	2.208	76.880

τ (C27-C30-C26-C21)	2.816	0.264	-2.552	-0.308	-4.283	-3.975	0.315
τ (C9-C14-C28-C29)	-2.816	-0.261	2.555	0.305	4.283	3.978	-0.315
τ (C28-C29-C16-C15)	-72.992	-75.210	-2.218	-75.640	-77.853	-2.213	-76.880
τ (C4-C3-C1-C2)	114.889	134.451	19.562	116.953	137.337	20.384	119.282
τ (C16-C15-C2-C1)	-114.889	-134.450	-19.561	-116.948	-137.346	-20.398	-119.282
***τ* (C8-C3-C1-C2)**	**-65.582**	**-45.238**	**20.344**	-64.488	-44.540	**19.948**	-62.582
***τ* (C20-C15-C2-C1)**	**65.582**	**45.236**	**-20.346**	64.492	44.534	**-19.958**	62.582
τ (C26-C21-C1-C2)	-130.705	-153.008	-22.303	-129.944	-149.995	-20.051	-127.232
τ (C14-C9-C2-C1)	130.705	153.007	22.302	129.946	150.007	20.061	127.232
***τ* (C22-C21-C1-C2)**	**52.649**	**33.763**	**-18.886**	54.121	37.284	**-16.837**	55.942
***τ* (C10-C9-C2-C1)**	**-52.649**	**-33.760**	**18.889**	-54.122	-37.283	**16.839**	-55.942
τ (C3-C1-C2-C9)	-3.719	-15.292	-11.573	-3.859	-16.931	-13.072	-5.420
τ (C3-C1-C2-C15)	-180.000	179.998	0.002	179.995	179.995	0.000	180.001
τ (C21-C1-C2-C9)	180.000	179.999	-0.001	179.997	179.986	-0.011	-180.001
***τ* (C21-C1-C2-C15)**	**3.719**	**15.289**	**11.570**	3.852	16.912	**13.060**	5.420
**plane angle between C3-C1-C21 and C9-C2-C15**	**0.000**	**0.004**	**0.004**	0.004	0.013	**0.009**	0.008
***d*(C20-C22)**	**3.444**	**2.958**	**-0.486**	**3.481**	**3.063**	**-0.418**	3.511
***d*(C8-C10)**	**3.444**	**2.958**	**-0.486**	**3.482**	**3.063**	**-0.419**	3.511
d(C20-C26)	4.036	4.019	-0.017	4.043	3.990	-0.053	3.940
d(C16-C22)	3.513	3.575	0.062	3.599	3.650	0.051	3.683
d(C16-C26)	5.172	5.460	0.288	5.209	5.452	0.243	5.189
d(C4-C10)	3.513	3.575	0.062	3.599	3.650	0.051	3.683
d(C4-C14)	5.172	5.460	0.288	5.209	5.452	0.243	5.189
d(C8-C14)	4.036	4.018	-0.018	4.403	3.990	-0.053	3.940

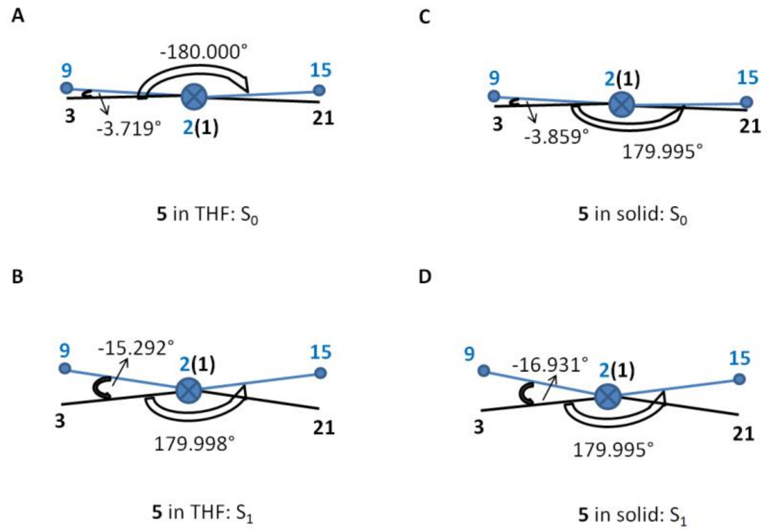

Figure S76. The changes in the dihedral angels *τ* (C3-C1-C2-C9) and *τ* (C3-C1-C2-C15) of **5** in solution (THF) and in solid state, at S_0 and S_1, respectively, with molecules looked upon along the C2-C1 bond with C2 as the proximal carbon atom are shown.

The top-bottom locked compound **5** has an even more rigid structure as compared to **1-4**, which is evidenced by the very small changes (~2.3°) in the angles of ∠ (C3-C1-C21) and ∠(C9-C2-C15), and nearly a 0° change of the plane (C3-C1-C21) to plane (C9-C2-C15) angle upon excitation. Although the C1-C2 twisting angle does change ($\Delta\tau$ (C21-C1-C2-C15) = 11.57°), it is much smaller than the twisting angle changes in **1-3**. While the changes due to the phenyl torsion upon excitation is similar to those in **3** (*e.g.* $\Delta\tau$ (C20-C15-C2-C1) = -20.35°, $\Delta\tau$ (C22-C21-C1-C2) = -18.89°), it is indicative of the out of sync torsion of the four phenyl rings in **5**. That further demonstrates that the introduction of the two ethylene bridges in the top-bottom positons in TPE resulting in **5** significantly restricts the C1-C2 twisting, while at the same time exerting some influence on the phenyl rings' motion that given their small amplitude can be classified as vibrations. Another big difference in **5** as compared to **1-3** is that the *d* (C20-C22) and *d* (C8-C10) distances become much smaller (2.958 Å) upon excitation, which might be due to the small angles of phenyl rings' vibration and the restricted C1-C2 bond twisting in this highly rigid structure. Similar bond lengths' changes can be found in the central C-C bond in **5** as compared to **1-3**.

5 undergoes similar amplitude of structural changes upon excitation in the solid state and solution (Figure S76), which might be due to its rigid structure.

Table S15. The metric parameters for **6** at the ground state (S_0), first excited state (S_1), and the corresponding structural difference △ (S_1-S_0) in THF solution at the M062X/6-311G(d) level using the Gaussian 09 package.[33] Bond lengths (*d*) are given in Å, angles in °.

**6**	in THF solution			in solid state	Crystal structure
Structural parameters	S_0	S_1	△ (S_1-S_0)	S_0	
***d* (C1-C2)**	**1.371**	**1.400**	**0.029**	**1.371**	**1.373**
d (C1-C3)	1.498	1.495	-0.003	1.497	1.500
d (C2-C9)	1.498	1.494	-0.004	1.500	1.498
***d* (C2-C15)**	**1.498**	**1.452**	**-0.046**	1.500	1.502
d (C1-C21)	**1.498**	**1.451**	**-0.047**	1.498	1.497
**∠ (C3-C1-C21)**	**115.119**	**117.530**	**2.411**	115.682	115.679
**∠ (C9-C2-C15)**	**115.117**	**117.459**	**2.342**	115.014	115.209
∠ (C3-C1-C2)	122.444	122.052	-0.392	121.446	122.730
∠ (C21-C1-C2)	122.437	120.213	-2.224	122.746	121.530
∠ (C9-C2-C1)	122.438	122.162	-0.276	122.919	122.020
∠ (C15-C2-C1)	122.445	120.155	-2.290	121.974	122.710
τ (C23-C30-C29-C19)	65.504	57.617	-7.887	64.497	64.240
τ (C7-C27-C28-C11)	65.505	65.946	0.441	64.237	64.690
τ (C22-C23-C30-C29)	-78.058	-59.884	-18.174	-81.264	-81.740
τ (C30-C29-C19-C20)	-78.060	-60.076	-17.984	-74.666	-73.440
τ (C8-C7-C27-C28)	-78.054	-78.600	-0.546	-82.298	-79.100
τ (C27-C28-C11-C10)	-78.067	-78.381	-0.314	-73.871	-75.980
∠ (C8-C7-C27)	119.436	119.586	0.150	120.452	119.450
∠ (C7-C27-C28)	109.720	109.472	-0.248	109.498	110.180
∠ (C27-C28-C11)	109.718	109.484	-0.234	109.937	109.379
∠ (C28-C11-C10)	119.437	119.581	0.144	119.719	119.339
∠(C22-C23-C30)	119.439	116.301	-3.138	119.804	119.730
∠ (C23-C30-C29)	109.721	108.783	-0.938	109.764	109.529
∠ (C30-C29-C19)	109.718	108.701	-1.017	109.679	110.088
∠ (C29-C19-C20)	119.433	116.229	-3.204	119.851	119.320
τ (C4-C3-C1-C2)	-126.604	-124.167	2.437	-119.944	-126.750

**τ (C14-C9-C2-C1)**	**-126.594**	**-124.505**	**2.089**	-131.354	-125.310
**τ (C8-C3-C1-C2)**	**40.932**	**42.494**	**1.562**	47.234	38.900
τ (C10-C9-C2-C1)	40.952	42.108	1.156	35.711	43.400
τ (C26-C21-C1-C2)	-126.600	-152.961	-26.361	-123.821	-119.870
τ (C16-C15-C2-C1)	-126.600	-152.149	-25.549	-127.494	-131.960
**τ (C22-C21-C1-C2)**	**40.936**	**18.283**	**-22.653**	41.142	47.240
**τ (C20-C15-C2-C1)**	**40.946**	**18.580**	**-22.366**	41.988	35.670
τ (C3-C1-C2-C9)	-7.363	-9.328	-1.965	-7.701	-8.460
τ (C3-C1-C2-C15)	172.627	176.248	3.621	175.973	168.020
τ (C21-C1-C2-C9)	172.647	176.026	3.379	168.044	174.990
**τ (C21-C1-C2-C15)**	**-7.363**	**1.601**	**8.964**	-8.282	-8.530
**plane angle between C3-C1-C21 and C9-C2-C15**	**7.363**	**3.649**	**-3.714**	10.091	10.079
***d*(C20-C22)**	**2.600**	**1.700**	**0.900**	2.600	2.583
***d*(C8-C10)**	**2.600**	**2.604**	**0.004**	2.603	2.582
d(C20-C26)	3.790	3.616	-0.174	3.754	3.676
d(C16-C22)	3.790	3.608	-0.182	3.816	3.882
d(C16-C26)	5.377	5.515	0.138	5.366	5.354
d(C4-C10)	3.790	3.778	-0.012	3.676	3.804
d(C4-C14)	5.377	5.374	-0.003	5.344	5.377
d(C8-C14)	3.790	3.785	-0.005	3.865	3.771

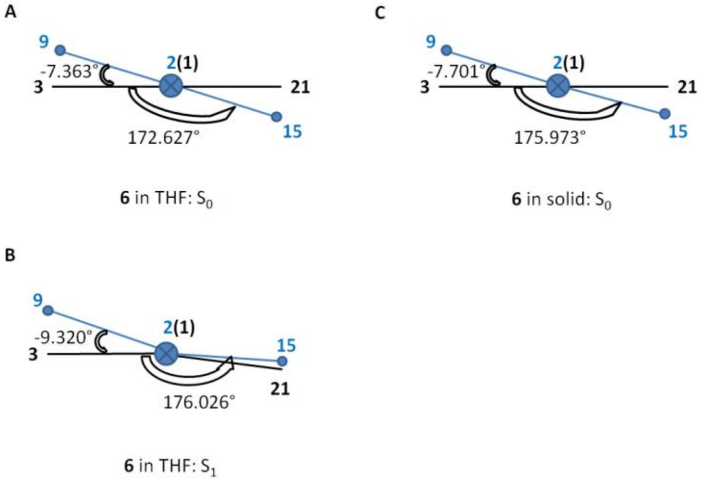

Figure S77. The changes in the dihedral angels τ (C3-C1-C2-C9) and τ (C3-C1-C2-C15) of **6** in solution (THF) and in solid state, at S_0 and S_1, respectively, with molecules looked upon along the C2-C1 bond with C2 as the proximal carbon atom are shown.

Remarkably, in **6** in solution, the elongation of the bond C1-C2 upon excitation is rather small (from 1.371 Å to 1.400 Å). The asymmetric changes in the lengths of the bonds connecting the ethylenic C1-C2 bond with the peripheral four phenyl groups upon excitation reveal that only two bonds (C2-C15 and C1-C21) in one side of the bilaterally locked TPE **6** are markedly shortened from 1.498 Å to 1.452 Å. This happens to be the side on which a very small *d* (C20-C22) distance of 1.70 Å is predicted which is rather close to the length of a C-C single bond, that subsequently forms in the corresponding intermediate **6-IM**. The distance *d* (C8-C10) on the other side of the bicyclic **6** in the excited state is predicted to remain approximately the same (2.60 Å) as compared to its ground state.

Similarly, small changes (~2.4°) in the ∠(C3-C1-C21) and ∠(C9-C2-C15) angles, and very small change (~3.7°) of plane (C3-C1-C21) to plane (C9-C2-C15) angle are found in **6** upon excitation as compared to **5**. The changes of the phenyl torsion upon excitation differ markedly depending on which

side of the bicyclic system of **6** they are located. On the side that ends up constituting the carbon framework of the dihydrophenanthrene ring after cyclization, one has similar changes in phenyl torsion (*e.g.* $\Delta\tau$ (C20-C15-C2-C1) = -22.37°, $\Delta\tau$ (C22-C21-C1-C2) = -22.65°) as in **3**, while on the other side there is no significant torsional changes (*e.g.* $\Delta\tau$ (C8-C3-C1-C2) = 1.56°, $\Delta\tau$ (C14-C9-C2-C1) = 2.09°). Thus, the changes of the four phenyl rings' torsion in **6** are out of sync. Additionally, the changes in the C1-C2 twisting angle (*e.g.* $\Delta\tau$ (C21-C1-C2-C15) = 8.96°) upon excitation is smaller than that in **5**. All of the above demonstrates that the compound **6** is the most rigid TPE derivative under consideration in this work.

The excited state calculation of **6** in solid state did not converge, thus the geometry changes in **6** as solid upon excitation and their comparison to **1**-**3** and **5** in terms of $\Delta(S_1\text{-}S_0)$ will not be discussed.

7.6 The optimized geometry of TPE derivatives

The nature of stationary points on the PES (potential energy surface) was determined by calculations of full Hessian matrix followed by frequency calculations. All optimized minimum energy structures reported were found to be true minima on the PES with no imaginary frequencies.

Quantum mechanical calculations for **1-6** in solution were performed at the DFT level of theory using M062X functional [31] and 6-311G (d) basis set [32] as implemented in the D0.1 version of the Gaussian 09 software package. [33]

The optimized geometry of **1-6** in solid state were analyzed using ONIOM(QM:MM) approach, an integrated quantum mechanics: molecular mechanics (QM:MM) method.[57-60] In the two-layer ONIOM calculation the system was divided into two parts, a selected centrally located molecule was treated by a DFT/TD-DFT (QM) method using M062X functional [31] while the packing surroundings in the crystal were treated by a low-level method, molecular mechanics (MM) method, the Universal Force Feld (UFF).[61] Geometry optimization, vibrational frequency and excited state calculations were performed using the 6-311G(d) basis set as implemented in the D0.1 version of Gaussian 09 software package. [33] The solid-phase Hessian of S_0 was evaluated analytically at the DFT level, while that of S_1 was calculated numerically at the TD-DFT level. The electrostatic interactions in the model were calculated semiclassically incorporating the MM charges into the QM Hamiltonian (electronic embedding). [60, 62] In our QM/MM model we neglect possible intermolecular excitonic couplings and assume that the intramolecular motions dominate the photophysical process. During the QM/MM simulations, only the central (QM) molecule was active for optimization, while all the surrounding (MM) molecules remained rigid.

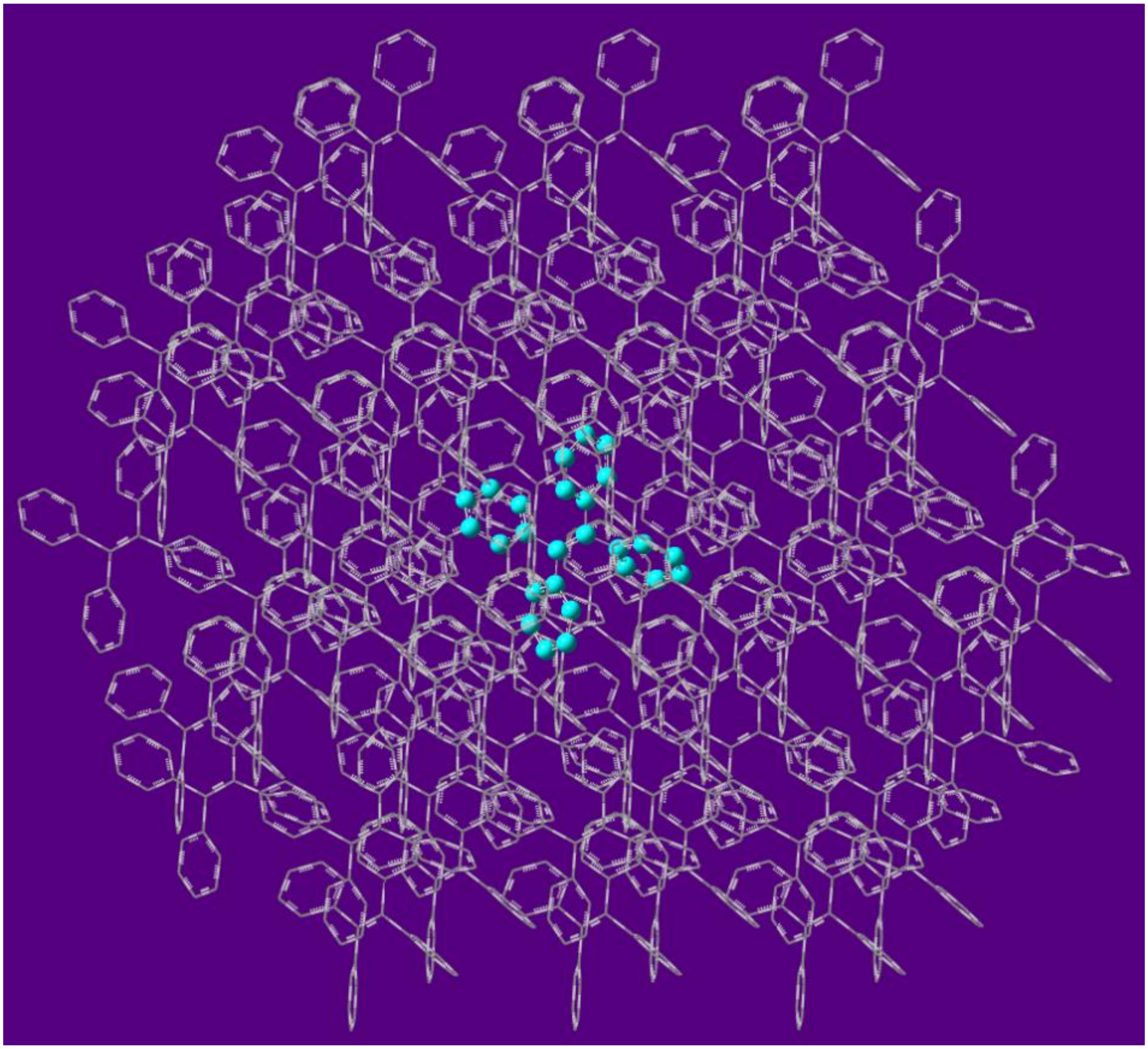

Figure S78. QM molecule of 1 (highlighted by cyan) surrounded by 70 MM molecules. All hydrogen atoms are omitted for clarity. Optimized cluster with **1** in the ground state and excited state are calculated using ONIOM QM:MM model with the high-level part of model shown using ball-and-stick representation (QM), and the low-level part of the model using wire representation (MM). All calculated at the M062X/6-311G (d) level [31-32] using the Gaussian 09 package.[33]

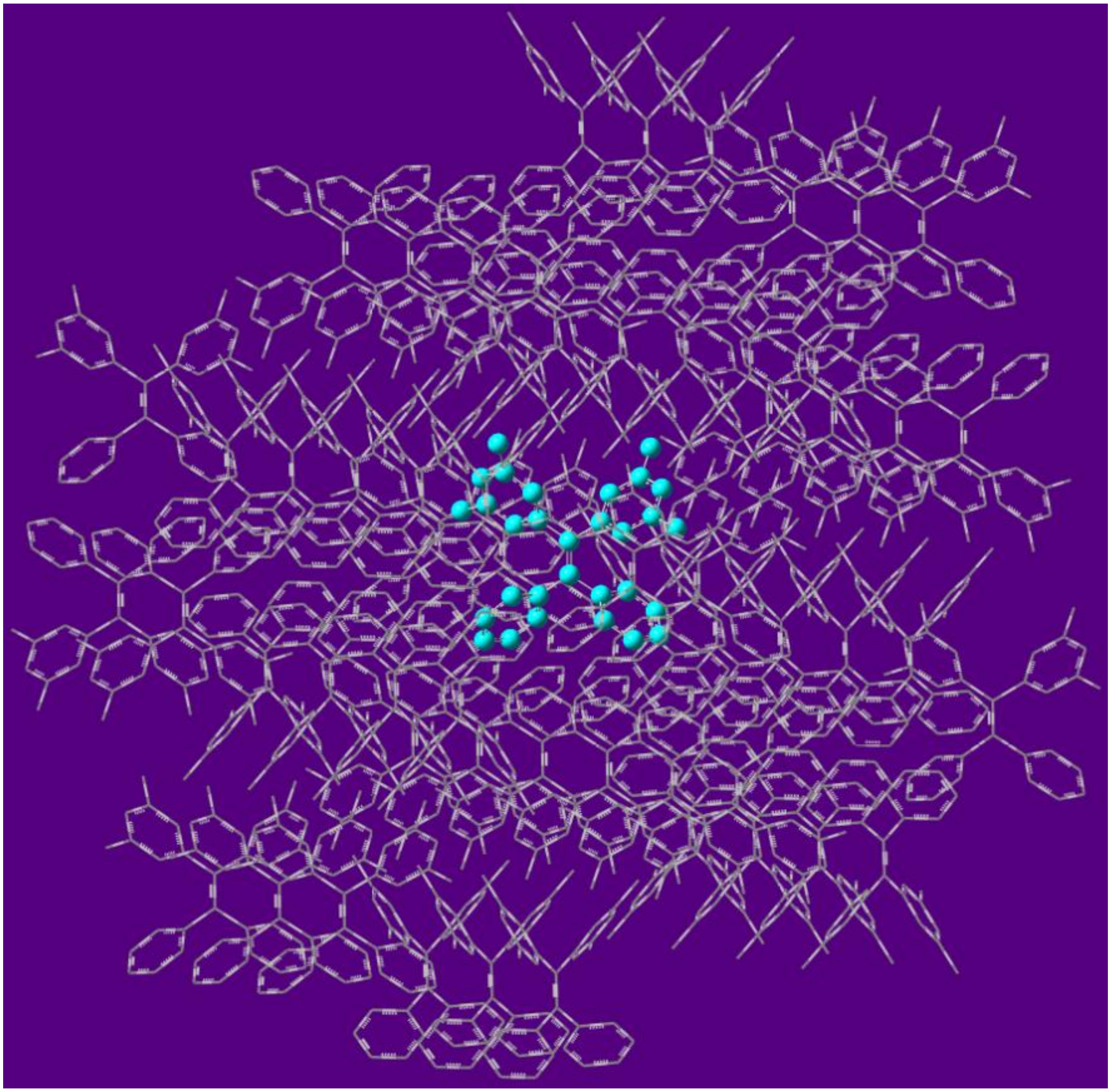

Figure S79. QM molecule of 2 (highlighted by cyan) surrounded by 58 MM molecules. All hydrogen atoms are omitted for clarity. Optimized cluster with **2** in the ground state and excited state are calculated using ONIOM QM:MM model with the high-level part of model shown using ball-and-stick representation (QM), and the low-level part of the model using wire representation (MM). All calculated at the M062X/6-311G (d) level [31-32] using the Gaussian 09 package.[33]

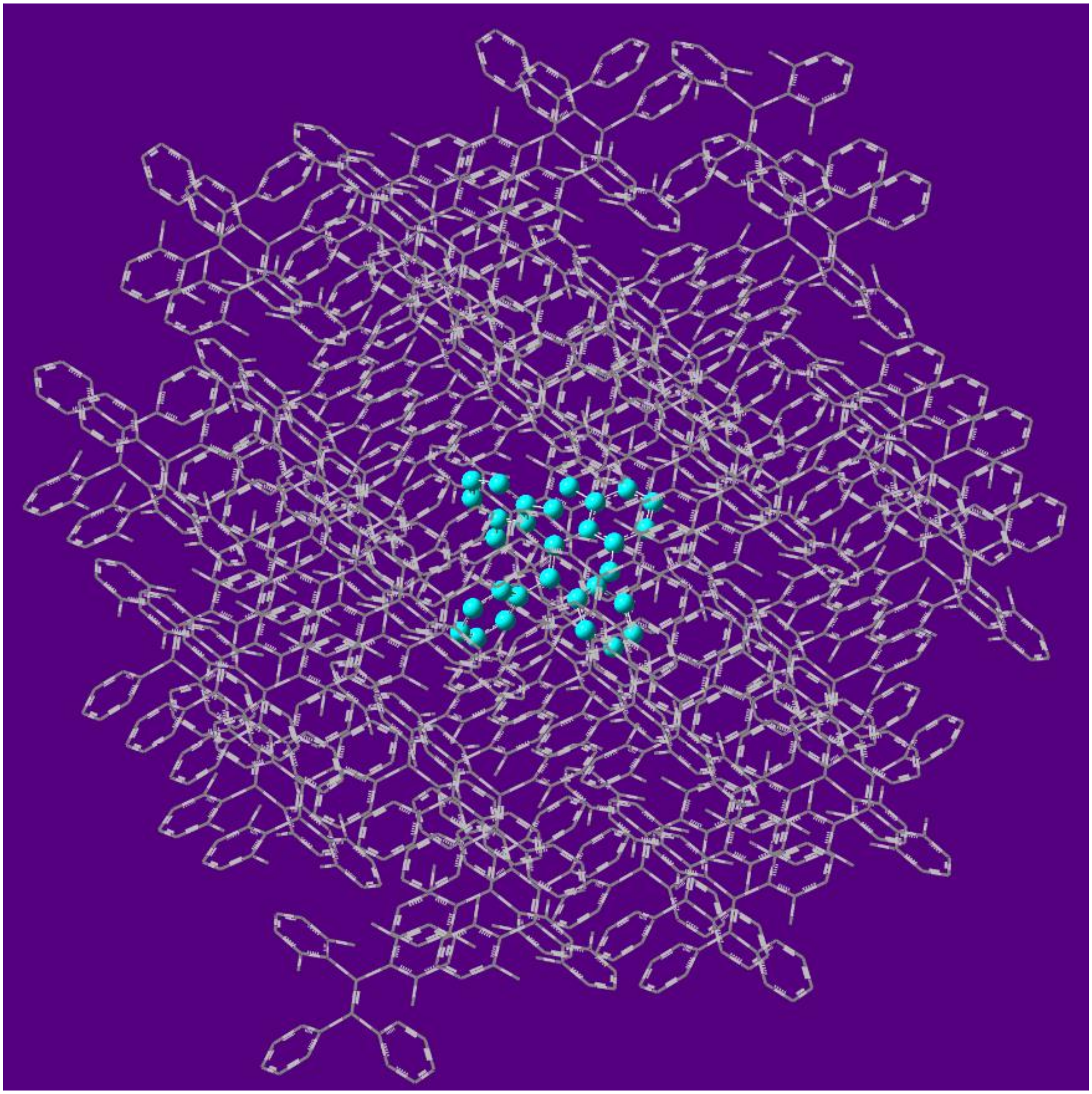

Figure S80. QM molecule of 3 (highlighted by cyan) surrounded by 60 MM molecules. All hydrogen atoms are omitted for clarity. Optimized cluster with **3** in the ground state and excited state are calculated using ONIOM QM:MM model with the high-level part of model shown using ball-and-stick representation (QM), and the low-level part of the model using wire representation (MM). All calculated at the M062X/6-311G (d) level [31-32] using the Gaussian 09 package.[33]

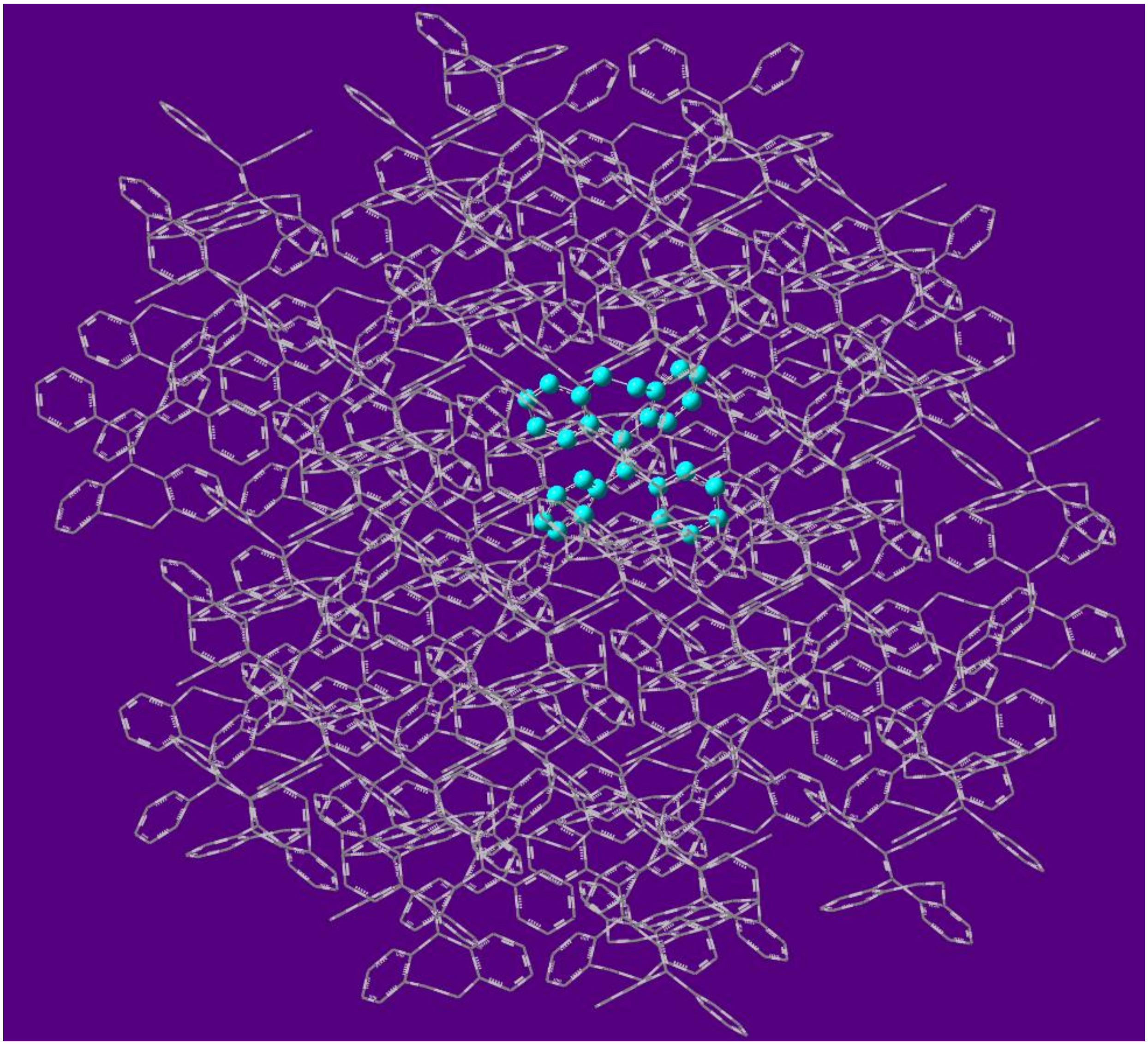

Figure S81. QM molecule of 4 (highlighted by cyan) surrounded by 75 MM molecules. All hydrogen atoms are omitted for clarity. Optimized cluster with **4** in the ground state is calculated using ONIOM QM:MM model with the high-level part of model shown using ball-and-stick representation (QM), and the low-level part of the model using wire representation (MM). All calculated at the M062X/6-311G (d) level [31-32] using the Gaussian 09 package.[33]

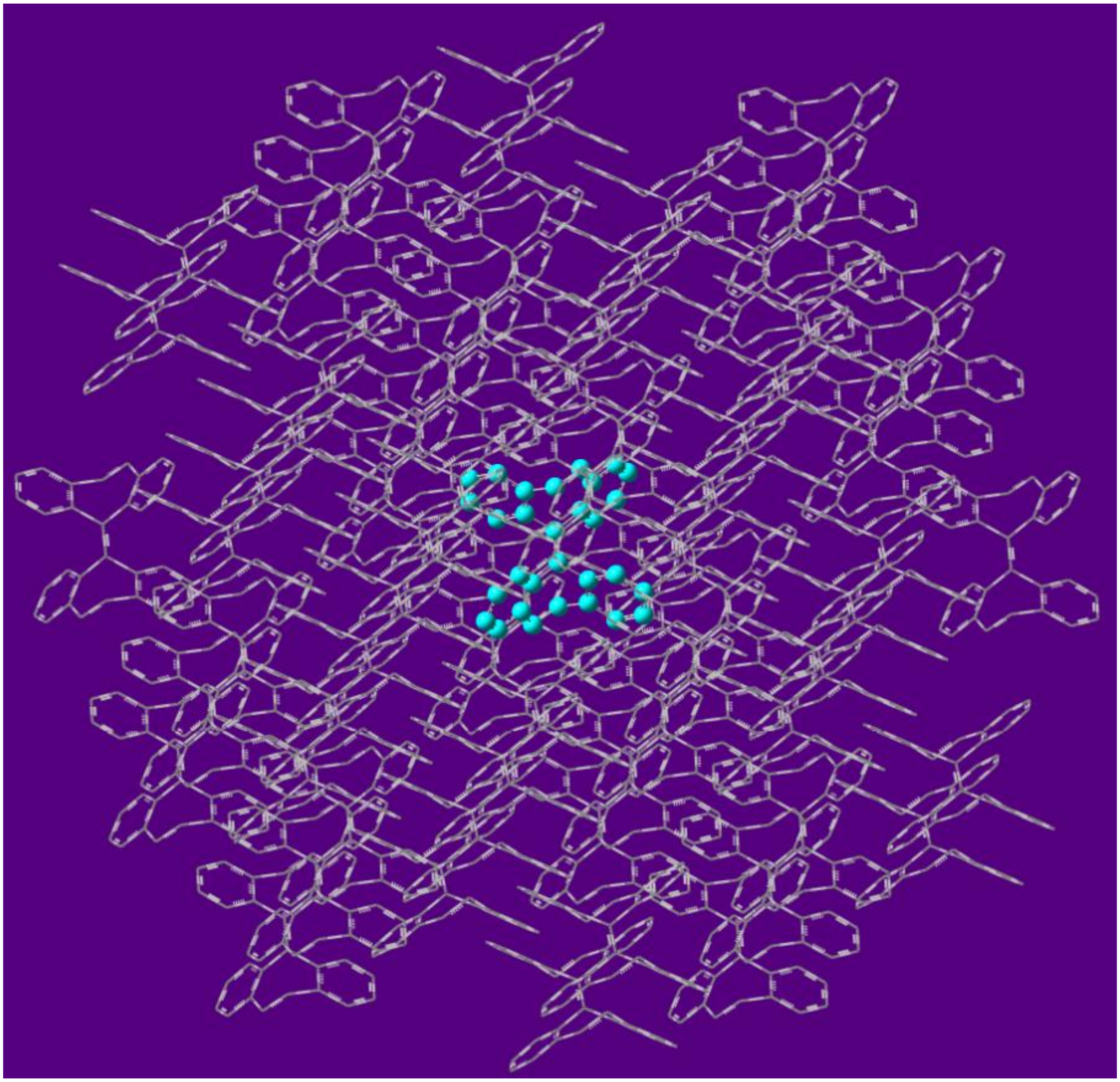

Figure S82. QM molecule of 5 (highlighted by cyan) surrounded by 70 MM molecules. All hydrogen atoms are omitted for clarity. Optimized cluster with **5** in the ground state and excited state are calculated using ONIOM QM:MM model with the high-level part of model shown using ball-and-stick representation (QM), and the low-level part of the model using wire representation (MM). All calculated at the M062X/6-311G (d) level [31-32] using the Gaussian 09 package.[33]

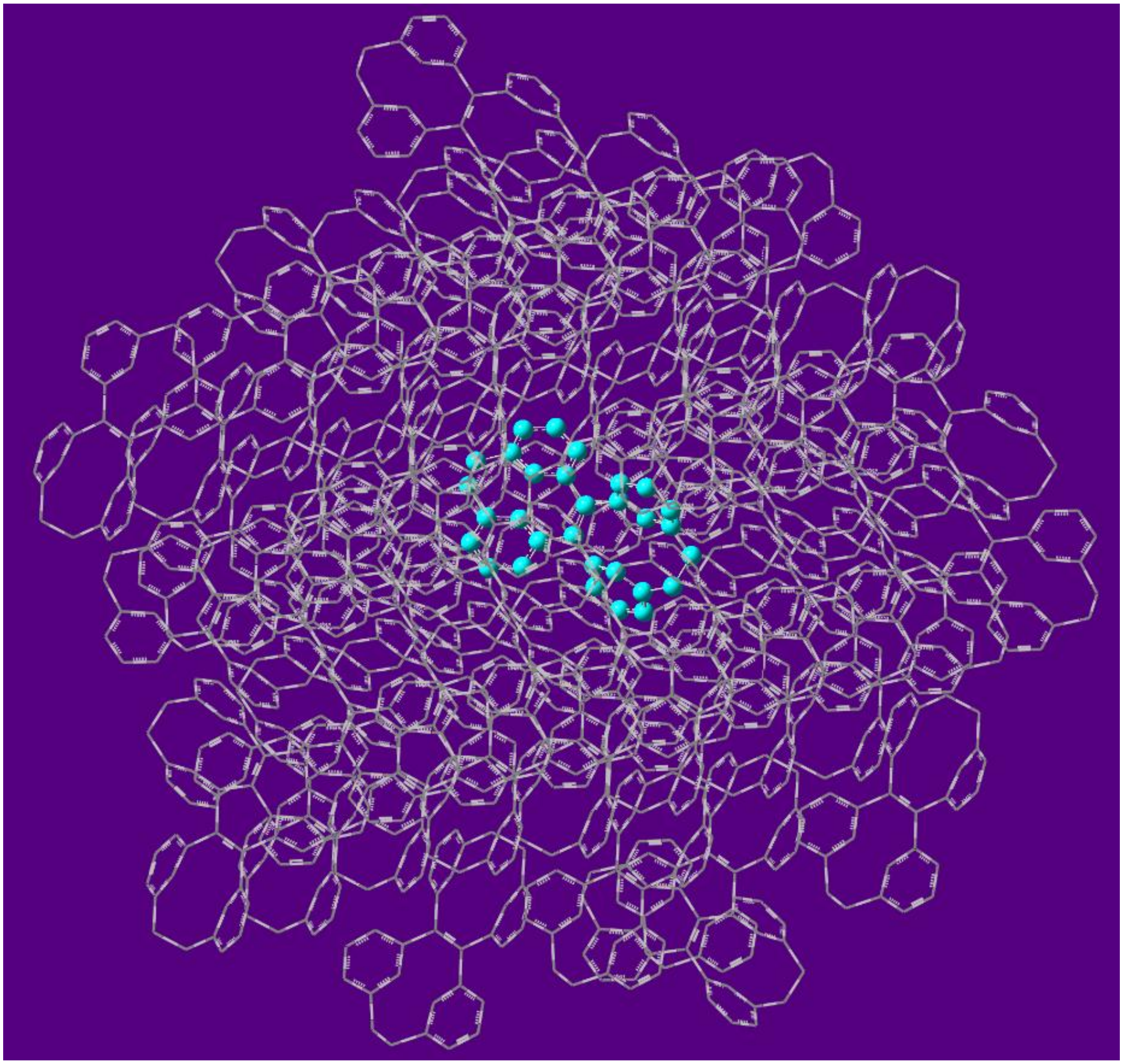

Figure S83. QM molecule of 6 (highlighted by cyan) surrounded by 58 MM molecules. All hydrogen atoms are omitted for clarity. Optimized cluster with **6** in the ground state is calculated using ONIOM QM:MM model with the high-level part of model shown using ball-and-stick representation (QM), and the low-level part of the model using wire representation (MM). All calculated at the M062X/6-311G (d) level [31-32] using the Gaussian 09 package.[33]

Table S16. The optimized geometric structure of **1** in MeCN at the transition state (**TS**) of ethylenic C=C bond twist performed at M062X/6-311G(d) using the Gaussian 09 package.[33]

Atom	x	y	z
C	-0.710000	-0.000000	0.000000
C	0.710000	0.000000	0.000000
C	-1.434300	-1.051800	-0.754400
C	-2.733900	-0.797600	-1.245100
C	-0.792100	-2.189200	-1.297100
C	-3.383000	-1.674400	-2.108000
H	-3.235300	0.128000	-1.003400
C	-1.445900	-3.071800	-2.138700
H	0.247700	-2.386000	-1.075600
C	-2.761000	-2.837300	-2.538300
H	-4.381500	-1.427900	-2.451100
H	-0.912000	-3.940700	-2.507000
H	-3.269600	-3.527300	-3.201200
C	-1.434300	1.051800	0.754400
C	-0.792100	2.189200	1.297100
C	-2.733900	0.797500	1.245100
C	-1.445900	3.071800	2.138700
H	0.247700	2.386000	1.075600
C	-3.383000	1.674400	2.108000
H	-3.235300	-0.128000	1.003400
C	-2.761000	2.837300	2.538300
H	-0.912000	3.940700	2.507000
H	-4.381500	1.427900	2.451100
H	-3.269600	3.527300	3.201200
C	1.434300	-1.051800	0.754300
C	2.734000	-0.797700	1.245000
C	0.792100	-2.189300	1.296900
C	3.383000	-1.674600	2.107800
H	3.235300	0.127800	1.003400
C	1.445900	-3.072000	2.138400
H	-0.247700	-2.386000	1.075400
C	2.761000	-2.837500	2.538000
H	4.381500	-1.428200	2.450900
H	0.912000	-3.940900	2.506600
H	3.269600	-3.527600	3.200800
C	1.434300	1.051800	-0.754300
C	2.734000	0.797700	-1.245000
C	0.792100	2.189300	-1.296900
C	3.383000	1.674600	-2.107800
H	3.235300	-0.127800	-1.003400
C	1.445800	3.072000	-2.138400
H	-0.247700	2.386000	-1.075400
C	2.760900	2.837500	-2.538000
H	4.381500	1.428200	-2.450900
H	0.912000	3.940900	-2.506600
H	3.269600	3.527600	-3.200800

Table S17. The optimized geometric structure of **1** in MeCN at the transition state (**TS**) of phenyl rotation performed at M062X/6-311G(d) using the Gaussian 09 package.[33]

Atom	x	y	z
C	0.019500	0.670400	-0.010200

C	-0.019500	-0.670400	0.010300
C	1.311000	1.424600	-0.077100
C	1.878100	2.014100	1.054800
C	1.955700	1.567500	-1.307600
C	3.068600	2.727400	0.958700
H	1.401000	1.894900	2.022100
C	3.146900	2.280000	-1.404500
H	1.519400	1.111300	-2.190600
C	3.705800	2.863400	-0.271400
H	3.501600	3.171900	1.847800
H	3.638100	2.379000	-2.366200
H	4.634200	3.418100	-0.345000
C	-1.220700	1.504300	-0.052200
C	-2.095800	1.420600	-1.138400
C	-1.510700	2.404700	0.976300
C	-3.242000	2.207800	-1.188000
H	-1.871500	0.737800	-1.950600
C	-2.660100	3.186200	0.931400
H	-0.840900	2.488200	1.825000
C	-3.529800	3.090100	-0.151200
H	-3.909600	2.132400	-2.039100
H	-2.876000	3.871200	1.743600
H	-4.424300	3.701600	-0.187200
C	-1.311000	-1.424600	0.077100
C	-1.955700	-1.567500	1.307600
C	-1.878100	-2.013900	-1.054900
C	-3.146900	-2.279900	1.404500
H	-1.519400	-1.111400	2.190600
C	-3.068700	-2.727200	-0.958700
H	-1.401000	-1.894700	-2.022100
C	-3.705800	-2.863300	0.271300
H	-3.638100	-2.379000	2.366100
H	-3.501600	-3.171700	-1.847900
H	-4.634300	-3.417900	0.344800
C	1.220700	-1.504400	0.052300
C	1.510500	-2.404900	-0.976100
C	2.096000	-1.420500	1.138300
C	2.660000	-3.186400	-0.931300
H	0.840700	-2.488400	-1.824800
C	3.242100	-2.207800	1.187900
H	1.871800	-0.737700	1.950500
C	3.529800	-3.090200	0.151200
H	2.875800	-3.871500	-1.743500
H	3.909800	-2.132300	2.039000
H	4.424200	-3.701800	0.187100

Table S18. The optimized geometric structure of **1** in MeCN at the ground state ($\mathbf{S_0}$) performed at M062X/6-311G(d) using the Gaussian 09 package.[33]

Atom	x	y	z
C	0.000000	0.000000	-0.675200
C	0.000000	0.000000	0.675200
C	-1.254000	-0.063800	1.480600
C	-1.458800	0.864800	2.505900
C	-2.622100	0.838800	3.266200
C	-3.587900	-0.135800	3.029200
C	-3.381400	-1.082000	2.028400

C	-2.224000	-1.045200	1.257900
H	-2.067800	-1.781000	0.476000
H	-4.123300	-1.852100	1.848800
H	-4.492800	-0.161900	3.625700
H	-2.772800	1.577000	4.046000
H	-0.702500	1.619900	2.697300
C	1.254000	0.063800	1.480600
C	1.458800	-0.864800	2.505900
C	2.622100	-0.838800	3.266200
C	3.587900	0.135800	3.029200
C	3.381400	1.082000	2.028400
C	2.224000	1.045200	1.257900
H	2.067800	1.781000	0.476000
H	4.123300	1.852100	1.848800
H	4.492800	0.161900	3.625700
H	2.772800	-1.577000	4.046000
H	0.702500	-1.619900	2.697300
C	1.254000	-0.063800	-1.480600
C	1.458800	0.864800	-2.505900
C	2.622100	0.838800	-3.266200
C	3.587900	-0.135800	-3.029200
C	3.381400	-1.082000	-2.028400
C	2.224000	-1.045200	-1.257900
H	2.067800	-1.781000	-0.476000
H	4.123300	-1.852100	-1.848800
H	4.492800	-0.161900	-3.625700
H	2.772800	1.577000	-4.046000
H	0.702500	1.619900	-2.697300
C	-1.254000	0.063800	-1.480600
C	-2.224000	1.045200	-1.257900
C	-3.381400	1.082000	-2.028400
C	-3.587900	0.135800	-3.029200
C	-2.622100	-0.838800	-3.266200
C	-1.458800	-0.864800	-2.505900
H	-0.702500	-1.619900	-2.697300
H	-2.772800	-1.577000	-4.046000
H	-4.492800	0.161900	-3.625700
H	-4.123300	1.852100	-1.848800
H	-2.067800	1.781000	-0.476000

Table S19. The optimized geometric structure of **1-IM** in MeCN at the ground state (**S$_0$**) performed at M062X/6-311G(d) using the Gaussian 09 package.[33]

Atom	x	y	z
C	0.69122800	-0.32893800	2.55518700
C	1.42947600	-0.13774400	1.24096700
C	0.73337900	-0.02829900	0.07693500
C	-0.73337900	0.02829900	0.07693500
C	-1.42947600	0.13774400	1.24096700
C	-2.88244400	0.17195900	1.28659300
C	-3.55258900	0.04862400	2.44941300
C	-2.84188800	-0.18607100	3.69939900
C	-1.50869600	-0.10641200	3.74792400
C	-0.69122800	0.32893800	2.55518700
H	-0.53102700	1.42277300	2.65579900
H	-0.99016800	-0.31764200	4.67659400

H	-3.41070600	-0.45921800	4.58154100
H	-4.63677600	0.04905900	2.45386400
H	-3.43149000	0.24085600	0.35535800
C	-1.42947600	-0.01281300	-1.24365800
C	-2.10909000	1.10803800	-1.72466300
C	-2.71536500	1.08863100	-2.97676300
C	-2.64762900	-0.05562600	-3.76592900
C	-1.97470100	-1.17963000	-3.29378900
C	-1.36841600	-1.15658800	-2.04261100
H	-0.83596600	-2.03078600	-1.68145600
H	-1.92183400	-2.07601700	-3.90176000
H	-3.11620500	-0.07269100	-4.74344000
H	-3.23551000	1.96932400	-3.33686100
H	-2.14972200	2.00467500	-1.11366300
C	1.42947600	0.01281300	-1.24365800
C	2.10909000	-1.10803800	-1.72466300
C	2.71536500	-1.08863100	-2.97676300
C	2.64762900	0.05562600	-3.76592900
C	1.97470100	1.17963000	-3.29378900
C	1.36841600	1.15658800	-2.04261100
H	0.83596600	2.03078600	-1.68145600
H	1.92183400	2.07601700	-3.90176000
H	3.11620500	0.07269100	-4.74344000
H	3.23551000	-1.96932400	-3.33686100
H	2.14972200	-2.00467500	-1.11366300
C	2.88244400	-0.17195900	1.28659300
C	3.55258900	-0.04862400	2.44941300
C	2.84188800	0.18607100	3.69939900
C	1.50869600	0.10641200	3.74792400
H	0.99016800	0.31764200	4.67659400
H	3.41070600	0.45921800	4.58154100
H	4.63677600	-0.04905900	2.45386400
H	3.43149000	-0.24085600	0.35535800
H	0.53102700	-1.42277300	2.65579900

Table S20. The optimized geometric structure of **1-PC** in MeCN at the ground state ($\mathbf{S_0}$) performed at M062X/6-311G(d) using the Gaussian 09 package.[33]

Atom	x	y	z
C	0.051500	-0.682000	0.016800
C	0.051700	0.682300	-0.017100
C	1.300000	1.419900	-0.063000
C	1.305900	2.830000	-0.169400
H	0.363200	3.361200	-0.209800
C	2.485600	3.535300	-0.232700
H	2.468000	4.615800	-0.316300
C	3.709300	2.851000	-0.195600
H	4.642300	3.400700	-0.244700
C	3.728400	1.477200	-0.105500
H	4.687500	0.976800	-0.091100
C	2.534200	0.726700	-0.041300
C	2.534000	-0.727200	0.041300
C	1.299600	-1.420000	0.062800
C	1.305000	-2.830100	0.169200
H	0.362100	-3.361000	0.209400
C	2.484600	-3.535700	0.232600
H	2.466600	-4.616300	0.316100

C	3.708500	-2.851800	0.195800
H	4.641200	-3.401700	0.245000
C	3.727900	-1.478000	0.105800
H	4.687200	-0.977900	0.091600
C	-1.236600	1.437300	-0.019300
C	-1.617300	2.174500	1.104200
H	-0.961500	2.203600	1.969000
C	-2.828800	2.856900	1.124900
H	-3.115300	3.419900	2.006100
C	-3.671300	2.815800	0.017100
H	-4.615300	3.348400	0.031800
C	-3.295000	2.090300	-1.109700
H	-3.944900	2.057500	-1.977000
C	-2.084400	1.404500	-1.127100
H	-1.794700	0.832100	-2.002300
C	-1.236900	-1.436900	0.019100
C	-2.084500	-1.404100	1.127100
H	-1.794600	-0.831600	2.002200
C	-3.295100	-2.089800	1.109800
H	-3.944900	-2.056900	1.977200
C	-3.671500	-2.815400	-0.016900
H	-4.615600	-3.348000	-0.031400
C	-2.829200	-2.856600	-1.124700
H	-3.115800	-3.419700	-2.005900
C	-1.617700	-2.174200	-1.104300
H	-0.962100	-2.203200	-1.969200

Table S21. The optimized geometric structure of **2** in MeCN at the ground state ($\mathbf{S_0}$) performed at M062X/6-311G(d) using the Gaussian 09 package.[33]

Atom	x	y	z
C	-2.788500	-1.823900	-3.965200
C	-1.825200	-2.031600	-2.824700
C	-1.361300	-0.962200	-2.067000
C	-0.483500	-1.162300	-0.994500
C	0.000000	0.000000	-0.194600
C	0.000000	0.000000	1.159400
C	-0.692900	-1.052600	1.956000
C	-2.013700	-1.413200	1.670700
H	-2.534900	-0.915700	0.859500
C	-2.655400	-2.400400	2.408400
H	-3.680500	-2.666000	2.174800
C	-1.985000	-3.045400	3.445200
H	-2.482900	-3.820700	4.016600
C	-0.676500	-2.681100	3.750800
H	-0.150900	-3.174000	4.561200
C	-0.040300	-1.683300	3.020000
H	0.978600	-1.399100	3.264400
C	0.692900	1.052600	1.956000
C	0.040300	1.683300	3.020000
H	-0.978600	1.399100	3.264400
C	0.676500	2.681100	3.750800
H	0.150900	3.174000	4.561200
C	1.985000	3.045400	3.445200
H	2.482900	3.820700	4.016600
C	2.655400	2.400400	2.408400
H	3.680500	2.666000	2.174800
C	2.013700	1.413200	1.670700

H	2.534900	0.915700	0.859500
C	0.483500	1.162300	-0.994500
C	1.361300	0.962200	-2.067000
H	1.690200	-0.046100	-2.302400
C	1.825200	2.031600	-2.824700
C	1.378400	3.319200	-2.509400
H	1.729900	4.160600	-3.101800
C	0.486600	3.546000	-1.465100
C	0.045400	2.454600	-0.714200
H	-0.650700	2.614800	0.104400
C	0.000000	4.934100	-1.135700
H	0.292900	5.218700	-0.121900
H	-1.090100	4.989300	-1.185000
H	0.408000	5.673500	-1.825700
C	2.788500	1.823900	-3.965200
H	2.388200	2.233800	-4.895500
H	2.995900	0.765100	-4.123500
H	3.738200	2.328800	-3.771500
C	-0.045400	-2.454600	-0.714200
C	-0.486600	-3.546000	-1.465100
C	-1.378400	-3.319200	-2.509400
H	-1.729900	-4.160600	-3.101800
C	0.000000	-4.934100	-1.135700
H	1.090100	-4.989300	-1.185000
H	-0.408000	-5.673500	-1.825700
H	-0.292900	-5.218700	-0.121900
H	0.650700	-2.614800	0.104400
H	-1.690200	0.046100	-2.302400
H	-2.388200	-2.233800	-4.895500
H	-2.995900	-0.765100	-4.123500
H	-3.738200	-2.328800	-3.771500

Table S22. The optimized geometric structure of **2-IM** in MeCN at the ground state ($\mathbf{S_0}$) performed at M062X/6-311G(d) using the Gaussian 09 package.[33]

Atom	x	y	z
C	-2.44638600	-0.94993200	0.59369500
C	-1.24493500	-1.80227000	0.18070400
C	-0.07692300	-1.15856800	-0.09509500
C	-0.03121000	0.31211200	-0.02047600
C	-1.19074000	1.02413700	-0.10710300
C	-2.48385100	0.24890000	-0.37198000
C	-3.74014600	1.09955300	-0.39728800
C	-3.67302000	2.41985600	-0.18675300
H	-4.57614900	3.01980800	-0.27969200
C	-2.42773000	3.12575600	0.11537400
C	-1.25832800	2.45641400	0.11101800
H	-0.33843000	2.98413000	0.33652700
C	-2.51761600	4.59513900	0.40294100
H	-1.53494100	5.03251300	0.58085200
H	-3.14351700	4.77860500	1.28187000
H	-2.98672500	5.12427100	-0.43224200
C	-5.03646700	0.46725100	-0.84769300
H	-4.86125800	-0.47066400	-1.37810400
H	-5.57322100	1.14354400	-1.51519200
H	-5.70293700	0.24558300	-0.01125000
H	-2.40618600	-0.18028800	-1.38845900

C	1.29522500	0.95846400	0.18085400
C	1.77681400	1.91851400	-0.71481100
H	1.15698600	2.21214200	-1.55768100
C	3.03984700	2.48379200	-0.55741700
C	3.83253300	2.06209100	0.51312900
H	4.82623800	2.48614800	0.63557400
C	3.38252500	1.10469900	1.42003300
C	2.10900800	0.56299600	1.24151600
H	1.74791100	-0.19303600	1.93427600
C	4.24094100	0.65574000	2.57493400
H	3.83453900	1.01249100	3.52501200
H	5.25977800	1.03403800	2.48411100
H	4.28453700	-0.43454500	2.62927700
C	3.54224400	3.53780400	-1.51085500
H	2.98228800	3.52642400	-2.44688200
H	4.59929000	3.39217400	-1.74118200
H	3.43780500	4.53505300	-1.07430300
C	1.17641400	-1.88417500	-0.44187400
C	1.86054800	-1.58502700	-1.62389500
H	1.45022700	-0.84265100	-2.30163600
C	3.05673000	-2.22245700	-1.93331800
H	3.57259400	-1.98221800	-2.85633700
C	3.59384100	-3.16498100	-1.05956800
H	4.52915400	-3.65868800	-1.29773500
C	2.92494400	-3.46661700	0.12305400
H	3.33920200	-4.19329900	0.81320900
C	1.72684100	-2.82919400	0.42832300
H	1.21691700	-3.04969500	1.36088500
C	-1.40882100	-3.24318300	0.07927800
H	-0.55645200	-3.83654600	-0.22970400
C	-2.59281100	-3.84108800	0.30667900
H	-2.68799600	-4.91602800	0.20044200
C	-3.77163900	-3.06479200	0.68049500
H	-4.69875400	-3.59020100	0.88248200
C	-3.71822100	-1.73351600	0.77650600
H	-4.59348500	-1.18024500	1.09448800
H	-2.21508400	-0.52333400	1.58895600

Table S23. The optimized geometric structure of **2-PC** in MeCN at the ground state (**S_0**) performed at M062X/6-311G(d) using the Gaussian 09 package.[33]

Atom	x	y	z
C	-5.086900	-0.751400	0.484400
C	-3.701300	-1.219700	0.093900
C	-2.555300	-0.373700	-0.039400
C	-2.598500	1.087700	-0.046200
C	-1.390200	1.824900	0.075600
C	-0.116300	1.138700	0.081900
C	-0.065700	-0.215300	-0.063800
C	-1.284000	-0.999800	-0.147000
C	-1.193000	-2.398300	-0.317500
C	-2.311600	-3.198800	-0.308100
C	-3.548000	-2.586100	-0.053500
H	-4.425700	-3.218800	0.050900
C	-2.231800	-4.686800	-0.516400
H	-1.197100	-5.026100	-0.572200
H	-2.733800	-4.975400	-1.443500
H	-2.725300	-5.222700	0.297900

H	-0.217200	-2.849700	-0.452300
C	1.258000	-0.908000	-0.096200
C	1.671500	-1.678600	0.993700
H	1.007300	-1.789800	1.846800
C	2.923000	-2.287200	1.007800
C	3.762800	-2.112400	-0.095700
H	4.747000	-2.574100	-0.088900
C	3.371900	-1.357800	-1.198900
C	2.109300	-0.760100	-1.186500
H	1.793600	-0.154600	-2.031700
C	4.279500	-1.184200	-2.389400
H	3.924700	-1.773600	-3.239200
H	5.297500	-1.503600	-2.163400
H	4.311200	-0.140300	-2.709000
C	3.362800	-3.131800	2.176100
H	2.792800	-2.893800	3.075100
H	4.422600	-2.985600	2.392600
H	3.216600	-4.194100	1.961900
C	1.147200	1.925700	0.204700
C	1.904500	1.873800	1.375300
H	1.554700	1.273400	2.208900
C	3.106400	2.569200	1.470000
H	3.687800	2.518300	2.383900
C	3.562900	3.325100	0.394500
H	4.501600	3.862800	0.466200
C	2.808700	3.388400	-0.774700
H	3.157700	3.975200	-1.617100
C	1.606400	2.695600	-0.866000
H	1.021300	2.737800	-1.779600
C	-1.437800	3.235000	0.157600
H	-0.515100	3.782800	0.302300
C	-2.624600	3.920700	0.038300
H	-2.638500	5.002800	0.099700
C	-3.805100	3.207200	-0.201700
H	-4.738800	3.733600	-0.363500
C	-3.787200	1.829500	-0.241300
H	-4.709500	1.324000	-0.470600
H	-5.056900	0.061600	1.210300
H	-5.628700	-1.582000	0.936900
H	-5.676400	-0.416600	-0.372700

Table S24. The optimized geometric structure of **3** in MeCN at the ground state (**S_0**) performed at M062X/6-311G(d) using the Gaussian 09 package.[33]

Atom	x	y	z
C	-1.892800	1.179900	-2.400400
C	-1.783100	-0.312600	-2.203200
C	-0.915800	-0.884900	-1.246200
C	0.000000	0.000000	-0.450200
C	0.000000	0.000000	0.903700
C	-1.033000	-0.717600	1.703100
C	-2.392500	-0.619900	1.390900
H	-2.702800	-0.005800	0.552300
C	-3.344900	-1.297000	2.144400
H	-4.395100	-1.206700	1.890300
C	-2.953300	-2.082800	3.225000
H	-3.696200	-2.613200	3.810000
C	-1.604300	-2.172900	3.558100

H	-1.292000	-2.776700	4.402900
C	-0.655200	-1.487100	2.809700
H	0.395400	-1.559500	3.073400
C	1.033000	0.717600	1.703100
C	2.392500	0.619900	1.390900
H	2.702800	0.005800	0.552300
C	3.344900	1.297000	2.144400
H	4.395100	1.206700	1.890300
C	2.953300	2.082800	3.225000
H	3.696200	2.613200	3.810000
C	1.604300	2.172900	3.558100
H	1.292000	2.776700	4.402900
C	0.655200	1.487100	2.809700
H	-0.395400	1.559500	3.073400
C	0.915800	0.884900	-1.246200
C	1.783100	0.312600	-2.203200
C	2.600900	1.138000	-2.971700
H	3.274900	0.688500	-3.694200
C	2.573700	2.518600	-2.818300
H	3.213200	3.150000	-3.425500
C	1.723300	3.078200	-1.879200
H	1.691800	4.156400	-1.752700
C	0.890900	2.285100	-1.085000
C	0.000000	3.016200	-0.109000
H	-0.763400	2.383500	0.338100
H	-0.494700	3.850800	-0.611900
H	0.595900	3.431700	0.708600
C	1.892800	-1.179900	-2.400400
H	1.914400	-1.712700	-1.448200
H	2.807700	-1.421800	-2.942300
H	1.048800	-1.577000	-2.970500
C	-0.890900	-2.285100	-1.085000
C	-1.723300	-3.078200	-1.879200
C	-2.573700	-2.518600	-2.818300
C	-2.600900	-1.138000	-2.971700
H	-3.274900	-0.688500	-3.694200
H	-3.213200	-3.150000	-3.425500
H	-1.691800	-4.156400	-1.752700
C	0.000000	-3.016200	-0.109000
H	0.763400	-2.383500	0.338100
H	0.494700	-3.850800	-0.611900
H	-0.595900	-3.431700	0.708600
H	-1.914400	1.712700	-1.448200
H	-2.807700	1.421800	-2.942300
H	-1.048800	1.577000	-2.970500

Table S25. The optimized geometric structure of **3-IM** in MeCN at the ground state (**S_0**) performed at M062X/6-311G (d) using the Gaussian 09 package. [33]

Atom	x	y	z
C	2.46341300	0.94029400	-0.51576300
C	1.15413600	1.67098300	-0.31571400
C	0.02390900	0.95502300	-0.09749900
C	0.06952300	-0.51443200	0.01760000
C	1.24616300	-1.17514500	0.17699900
C	2.55263100	-0.37532600	0.28519700
C	3.72548500	-1.16812000	-0.25605600
H	4.60651600	-0.62343500	-0.57792300

C	3.71708600	-2.50104000	-0.28599000
H	4.58128100	-3.05673600	-0.63418100
C	2.54879200	-3.23173700	0.17951400
H	2.64491100	-4.30091900	0.34210600
C	1.35801800	-2.63403400	0.40169100
C	0.21749000	-3.48094800	0.91655300
H	-0.27267500	-3.00857200	1.77044000
H	0.60512900	-4.44802500	1.23977600
H	-0.55322700	-3.66057100	0.16479000
C	2.79391600	-0.09989700	1.78956800
H	2.81213400	-1.04451000	2.33666900
H	3.74512600	0.41038400	1.95402100
H	1.99032000	0.52223100	2.19381600
C	-1.25629100	-1.20086400	-0.10463300
C	-1.61078000	-1.74636500	-1.35061300
C	-2.87073300	-2.32088900	-1.50439500
H	-3.15085700	-2.74211400	-2.46481600
C	-3.77011400	-2.35657200	-0.44321900
H	-4.74926300	-2.80423700	-0.57539900
C	-3.40884400	-1.81911100	0.78354000
H	-4.10425000	-1.85206600	1.61720300
C	-2.15476600	-1.23364100	0.96834400
C	-1.79900300	-0.70122200	2.33443700
H	-0.80238100	-0.26045000	2.36274900
H	-1.83024600	-1.50695300	3.07388400
H	-2.51435200	0.06064300	2.65272100
C	-0.64128200	-1.72882900	-2.50617100
H	-0.23910200	-0.72657800	-2.67989500
H	-1.12383200	-2.06960500	-3.42272700
H	0.21585900	-2.38008000	-2.30921900
C	-1.29762500	1.64769100	-0.00454300
C	-2.21953500	1.58708500	-1.05364100
H	-1.97382300	1.02465800	-1.94872400
C	-3.44460600	2.23676400	-0.95899100
H	-4.14948800	2.17929600	-1.78099300
C	-3.76579100	2.96066600	0.18764900
H	-4.72278400	3.46470700	0.26324400
C	-2.85071100	3.03832000	1.23196200
H	-3.09043800	3.60517700	2.12479500
C	-1.62410300	2.38620000	1.13384400
H	-0.91051400	2.44315700	1.95011800
C	1.14368200	3.11516000	-0.50761800
H	0.18695300	3.61784100	-0.59001800
C	2.28662900	3.82353600	-0.55272500
H	2.25851500	4.89899700	-0.68761700
C	3.57677200	3.16775800	-0.36577200
H	4.46574500	3.78067400	-0.26390500
C	3.66369500	1.83578300	-0.31052000
H	4.63351600	1.37317700	-0.16422600
H	2.47828600	0.64136500	-1.58488000

Table S26. The optimized geometric structure of **3-PC** in MeCN at the ground state (**S_0**) performed at M062X/6-311G(d) using the Gaussian 09 package.[33]

Atom	x	y	z
C	-0.327500	-3.574300	0.461300

C	-1.501900	-2.636000	0.270800
C	-2.748800	-3.235800	0.283300
C	-3.932400	-2.500900	0.176200
C	-3.871300	-1.135100	0.074300
C	-2.631900	-0.462100	0.050300
C	-2.598800	0.992400	-0.048200
C	-1.355500	1.661000	-0.058800
C	-0.133600	0.888800	0.003700
C	-0.158800	-0.479000	0.057400
C	-1.420100	-1.209400	0.121900
C	1.165300	-1.177200	-0.023600
C	1.965200	-1.313200	1.118200
C	3.227800	-1.892300	0.991600
H	3.851800	-2.004700	1.872600
C	3.688000	-2.330500	-0.244300
H	4.673800	-2.774100	-0.331900
C	2.877600	-2.209400	-1.366900
H	3.230400	-2.564500	-2.330100
C	1.609200	-1.638400	-1.271200
C	0.714300	-1.568000	-2.482800
H	-0.114600	-2.278600	-2.393500
H	1.266200	-1.810200	-3.391300
H	0.263300	-0.580200	-2.604000
C	1.456200	-0.886700	2.470800
H	1.403200	0.201500	2.551900
H	2.108900	-1.252000	3.264400
H	0.447900	-1.269700	2.652400
C	1.164100	1.635800	0.001200
C	1.525300	2.391800	1.120000
H	0.853900	2.433500	1.972400
C	2.727100	3.091500	1.149600
H	2.993900	3.666900	2.029100
C	3.581100	3.055300	0.051500
H	4.517700	3.600800	0.071400
C	3.221100	2.323000	-1.076300
H	3.875000	2.298800	-1.940800
C	2.020300	1.620900	-1.101300
H	1.747600	1.055600	-1.985500
C	-1.326500	3.070800	-0.183100
H	-0.372500	3.580500	-0.220900
C	-2.487600	3.801800	-0.269400
H	-2.445000	4.880600	-0.366100
C	-3.725700	3.143500	-0.237600
H	-4.647000	3.711200	-0.300600
C	-3.775700	1.772300	-0.134200
H	-4.749200	1.302800	-0.122900
H	-4.797400	-0.581800	0.015200
H	-4.890600	-3.007500	0.190500
H	-2.808600	-4.313200	0.393500
H	0.319600	-3.633400	-0.414600
H	-0.707400	-4.576700	0.658600
H	0.300300	-3.288600	1.305200

Table S27. The optimized geometric structure of **4** in MeCN at the ground state (**S_0**) performed at M062X/6-311G(d) using the Gaussian 09 package.[33]

Atom	x	y	z
C	0.953600	-2.864500	1.512100

C	1.757100	-2.253800	0.373500
C	1.374900	-1.152600	-0.411000
C	0.061300	-0.455500	-0.257900
C	-0.058800	0.873200	-0.086100
C	1.097900	1.794700	0.113900
C	2.133600	1.483100	1.001700
C	3.195600	2.360400	1.187600
C	3.241900	3.564300	0.489100
C	2.210200	3.890900	-0.385600
C	1.141700	3.018500	-0.561300
H	0.335000	3.287500	-1.234800
H	2.232900	4.828900	-0.928800
H	4.071500	4.247200	0.632700
H	3.987600	2.105500	1.882900
H	2.102600	0.547900	1.550600
C	-1.406400	1.519500	-0.003500
C	-1.995300	1.722300	1.246300
C	-3.244100	2.326700	1.349800
C	-3.912900	2.748400	0.204000
C	-3.326800	2.562800	-1.044600
C	-2.080600	1.951100	-1.146800
H	-1.628000	1.802200	-2.122200
H	-3.841300	2.889400	-1.941400
H	-4.885900	3.219800	0.284800
H	-3.695000	2.468400	2.325900
H	-1.470100	1.398900	2.140000
C	-1.132200	-1.349800	-0.323600
C	-1.991700	-1.363300	-1.420400
C	-3.084000	-2.225200	-1.448300
C	-3.317600	-3.080800	-0.376800
C	-2.454000	-3.076800	0.715300
C	-1.356800	-2.218100	0.752700
C	-0.377700	-2.211500	1.896200
H	-0.203000	-1.184000	2.231400
H	-0.793300	-2.758100	2.745600
H	-2.635800	-3.745600	1.551200
H	-4.169600	-3.751300	-0.390900
H	-3.748700	-2.227100	-2.304800
H	-1.800500	-0.698700	-2.255600
C	2.229600	-0.707100	-1.430200
C	3.450400	-1.315000	-1.672700
C	3.840900	-2.397100	-0.886900
C	2.997600	-2.850300	0.115400
H	3.301100	-3.695800	0.726800
H	4.793800	-2.885400	-1.057600
H	4.091000	-0.951600	-2.468100
H	1.915200	0.135100	-2.037800
H	0.760600	-3.913400	1.259300
H	1.596700	-2.885700	2.397700

Table S28. The optimized geometric structure of **4-IM** in MeCN at the ground state (**S_0**) performed at M062X/6-311G(d) using the Gaussian 09 package.[33]

Atom	x	y	z
C	2.18414800	-2.97873100	-0.14498000
C	2.64245600	-1.61486800	-0.58862200
C	1.78504100	-0.48992200	-0.66352900
C	0.33282600	-0.50205500	-0.34077300

C	-0.42381100	0.79242600	-0.33154200
C	0.20007500	1.92693100	0.40718200
C	0.82965200	1.69860200	1.63537300
C	1.41055900	2.74574500	2.34163400
C	1.37946200	4.03972200	1.82912800
C	0.75951900	4.27877900	0.60573100
C	0.17726600	3.23098700	-0.09889600
H	-0.28194200	3.41562200	-1.06504800
H	0.73617800	5.28161300	0.19411000
H	1.83905800	4.85498100	2.37630400
H	1.89015400	2.55084200	3.29449100
H	0.85964300	0.69102500	2.03860200
C	-1.72981700	0.82038100	-0.69749600
C	-2.66898700	1.88765400	-0.40475700
C	-3.97507500	1.77540800	-0.72015600
C	-4.50319200	0.58407200	-1.37864300
C	-3.71250800	-0.46309300	-1.63557600
C	-2.25735400	-0.46841700	-1.27653800
H	-1.67797200	-0.69643100	-2.18148400
C	-1.89980500	-1.61408900	-0.29740900
C	-0.42840700	-1.57265400	0.07429200
C	-0.02622100	-2.63464700	0.98194400
C	-0.97336100	-3.15243300	1.80956100
C	-2.34302100	-2.66818200	1.84455700
C	-2.80182200	-1.86809600	0.87108900
H	-3.81230600	-1.47298500	0.87002400
H	-2.97057100	-2.95090000	2.68279100
H	-0.66146100	-3.86590000	2.56673300
C	1.42337100	-2.96790100	1.17946600
H	1.88197400	-2.23604900	1.85546900
H	1.49857900	-3.93962000	1.67246900
H	-1.95687900	-2.52842700	-0.91467500
H	-4.10617900	-1.35502100	-2.11504500
H	-5.55350000	0.56177900	-1.64914300
H	-4.66110100	2.57784000	-0.47282400
H	-2.31509200	2.77454000	0.10693800
C	2.31849500	0.67940600	-1.24164400
C	3.64115300	0.78242600	-1.64163300
C	4.49297100	-0.30420000	-1.48934700
C	3.97727300	-1.48739500	-0.97966100
H	4.62418400	-2.35466600	-0.89440400
H	5.53417400	-0.24246800	-1.78488800
H	3.99862300	1.70919400	-2.07620900
H	1.67605400	1.53152500	-1.40868500
H	1.53638600	-3.41187300	-0.91786500
H	3.05667800	-3.62974700	-0.07017500

Table S29. The optimized geometric structure of **4-PC** in MeCN at the ground state (**S_0**) performed at M062X/6-311G(d) using the Gaussian 09 package.[33]

Atom	x	y	z
C	-0.431200	-3.505800	0.721800
C	-1.509200	-2.583800	0.169600
C	-2.741500	-3.179400	-0.038100
C	-3.868800	-2.440000	-0.400900
C	-3.785300	-1.069200	-0.455300

C	-2.561300	-0.409400	-0.230800
C	-2.506800	1.046100	-0.141600
C	-1.266000	1.679500	0.092200
C	-0.050700	0.890900	0.060700
C	-0.096600	-0.474000	-0.053400
C	-1.379000	-1.175800	-0.029900
C	1.183100	-1.195800	-0.345200
C	1.647700	-2.237300	0.461900
C	0.715500	-2.825600	1.476800
H	0.319600	-2.060900	2.153300
H	1.237600	-3.565400	2.086300
C	2.898700	-2.799700	0.211900
H	3.254400	-3.601800	0.851200
C	3.681900	-2.356900	-0.847600
H	4.654800	-2.801000	-1.026800
C	3.197100	-1.360700	-1.690100
H	3.782600	-1.028400	-2.540000
C	1.957400	-0.787700	-1.436500
H	1.584200	-0.005100	-2.087800
C	1.260900	1.596800	0.174800
C	2.106300	1.304400	1.247000
H	1.789100	0.576400	1.987200
C	3.346000	1.923800	1.363000
H	3.991600	1.683600	2.200300
C	3.756600	2.846100	0.405000
H	4.725100	3.325700	0.490800
C	2.916300	3.152300	-0.662600
H	3.230200	3.869400	-1.412900
C	1.674500	2.536000	-0.772800
H	1.025200	2.770800	-1.610800
C	-1.235100	3.074100	0.322200
H	-0.290000	3.554700	0.539600
C	-2.385200	3.828500	0.279400
H	-2.341900	4.896000	0.462200
C	-3.612100	3.209000	0.003700
H	-4.522600	3.796100	-0.034100
C	-3.668400	1.847300	-0.195400
H	-4.636700	1.398500	-0.370200
H	-4.681700	-0.503700	-0.668400
H	-4.814400	-2.938400	-0.580500
H	-2.835100	-4.250200	0.115800
H	-0.005400	-4.111600	-0.085700
H	-0.934900	-4.206700	1.392100

Table S30. The optimized geometric structure of **5** in MeCN at the ground state (**S_0**) performed at M062X/6-311G(d) using the Gaussian 09 package.[33]

Atom	x	y	z
C	-0.847100	0.874700	3.018400
H	-0.849300	0.875300	4.110500
H	0.202200	0.873700	2.702100
C	-1.536400	-0.361500	2.505100
C	-1.391500	-0.694000	1.152700
C	-0.577000	0.201700	0.281900
C	-1.178400	1.548800	0.022300
C	-1.612100	2.414400	1.040800
C	-2.184600	3.639200	0.672200
H	-2.507300	4.314200	1.460100

C	-2.352400	4.009800	-0.652000
H	-2.801200	4.965900	-0.896800
C	-1.949800	3.136900	-1.659900
H	-2.088000	3.395400	-2.703500
C	-1.374000	1.925400	-1.316100
H	-1.071600	1.239700	-2.099600
C	-1.522900	2.160700	2.537500
H	-1.007800	3.013300	2.991200
H	-2.542900	2.182400	2.937700
C	0.577000	-0.201700	-0.281900
C	1.178400	-1.548800	-0.022300
C	1.612100	-2.414400	-1.040800
C	1.522900	-2.160700	-2.537500
C	0.847100	-0.874700	-3.018400
H	0.849300	-0.875300	-4.110500
H	-0.202200	-0.873700	-2.702100
C	1.536400	0.361500	-2.505100
C	1.391500	0.694000	-1.152700
C	2.007000	1.834900	-0.641900
H	1.877600	2.083200	0.407000
C	2.769600	2.651400	-1.471300
H	3.244700	3.538900	-1.068800
C	2.916900	2.326200	-2.816200
H	3.508300	2.960100	-3.467600
C	2.300000	1.187200	-3.327600
H	2.405700	0.940700	-4.379800
H	1.007800	-3.013300	-2.991200
H	2.542900	-2.182400	-2.937700
C	2.184600	-3.639200	-0.672200
H	2.507300	-4.314200	-1.460100
C	2.352400	-4.009800	0.652000
H	2.801200	-4.965900	0.896800
C	1.949800	-3.136900	1.659900
H	2.088000	-3.395400	2.703500
C	1.374000	-1.925400	1.316100
H	1.071600	-1.239700	2.099600
C	-2.007000	-1.834900	0.641900
H	-1.877600	-2.083200	-0.407000
C	-2.769600	-2.651400	1.471300
H	-3.244700	-3.538900	1.068800
C	-2.916900	-2.326200	2.816200
H	-3.508300	-2.960100	3.467600
C	-2.300000	-1.187200	3.327600
H	-2.405700	-0.940700	4.379800

Table S31. The optimized geometric structure of **5-IM** in MeCN at the ground state (**S_0**) performed at M062X/6-311G(d) using the Gaussian 09 package.[33]

**Atom**	**x**	**y**	**z**
C	0.57599400	0.51353800	2.47247300
C	1.43962900	0.20311600	1.24544900
C	0.74424300	-0.08294500	0.10748900
C	-0.74424300	0.08294500	0.10748900
C	-1.43962900	-0.20311600	1.24544900
C	-0.57599400	-0.51353800	2.47247300
C	-1.31748900	-0.59474400	3.77549200
H	-0.73273300	-0.78794000	4.66753900
C	-2.64712500	-0.53986600	3.84219100

H	-3.16079500	-0.64606100	4.79144800
C	-3.43949800	-0.40662300	2.62966500
H	-4.51938600	-0.46935000	2.72402800
C	-2.90319100	-0.24042700	1.40150300
C	-3.85240600	-0.15213000	0.22822100
H	-4.05778000	0.89729100	-0.01033900
H	-4.80469400	-0.58943000	0.53570400
C	-3.33419800	-0.84434400	-1.04097900
H	-2.78930100	-1.75446000	-0.76706600
H	-4.17351800	-1.14363800	-1.67110700
C	-2.45562900	0.11486100	-1.78620300
C	-1.29677100	0.61341500	-1.17452600
C	-0.57599400	1.62060000	-1.83181900
C	-0.97276300	2.10902300	-3.06900800
C	-2.10521100	1.58538500	-3.68549200
C	-2.84123800	0.60271600	-3.03497300
H	-3.73907700	0.20526900	-3.49807800
H	-2.41956500	1.94716400	-4.65803500
H	-0.39655000	2.89034500	-3.55183600
H	0.31772700	2.02090800	-1.36315000
H	-0.09991200	-1.50049300	2.32399900
C	1.29677100	-0.61341500	-1.17452600
C	0.57599400	-1.62060000	-1.83181900
C	0.97276300	-2.10902300	-3.06900800
H	0.39655000	-2.89034500	-3.55183600
C	2.10521100	-1.58538500	-3.68549200
H	2.41956500	-1.94716400	-4.65803500
C	2.84123800	-0.60271600	-3.03497300
H	3.73907700	-0.20526900	-3.49807800
C	2.45562900	-0.11486100	-1.78620300
C	3.33419800	0.84434400	-1.04097900
C	3.85240600	0.15213000	0.22822100
C	2.90319100	0.24042700	1.40150300
C	3.43949800	0.40662300	2.62966500
H	4.51938600	0.46935000	2.72402800
C	2.64712500	0.53986600	3.84219100
H	3.16079500	0.64606100	4.79144800
C	1.31748900	0.59474400	3.77549200
H	0.73273300	0.78794000	4.66753900
H	4.05778000	-0.89729100	-0.01033900
H	4.80469400	0.58943000	0.53570400
H	4.17351800	1.14363800	-1.67110700
H	2.78930100	1.75446000	-0.76706600
H	-0.31772700	-2.02090800	-1.36315000
H	0.09991200	1.50049300	2.32399900

Table S32. The optimized geometric structure of **5-PC** in MeCN at the ground state (**S_0**) performed at M062X/6-311G(d) using the Gaussian 09 package.[33]

Atom	x	y	z
C	0.849800	-3.725800	0.264000
C	0.405800	-2.852800	1.426400
C	0.138500	-1.446100	1.366800
C	0.002400	-0.690700	0.127800
C	-0.002400	0.690700	0.127800
C	-0.138500	1.446100	1.366800
C	-0.042800	0.728600	2.589100

C	0.042800	-0.728600	2.589100
C	-0.002400	-1.437700	3.808600
H	-0.159400	-0.918500	4.742300
C	0.124100	-2.803500	3.840200
H	0.073400	-3.337100	4.782300
C	0.377000	-3.491600	2.652200
H	0.581300	-4.557000	2.689100
C	0.002400	1.437700	3.808600
C	-0.124100	2.803500	3.840200
C	-0.377000	3.491600	2.652200
C	-0.405800	2.852800	1.426400
C	-0.849800	3.725800	0.264000
C	-1.580400	2.991900	-0.865000
C	-0.537800	2.358000	-1.732600
C	0.277500	1.363200	-1.185400
C	1.391200	0.931700	-1.915900
C	1.656500	1.424700	-3.186700
H	2.522200	1.069700	-3.734400
C	0.804400	2.369600	-3.750400
H	0.991200	2.752200	-4.747600
C	-0.275500	2.841500	-3.013300
H	-0.924000	3.605700	-3.430400
H	2.048300	0.184600	-1.482800
H	-2.173700	3.702200	-1.443600
H	-2.269200	2.250200	-0.446700
H	0.007300	4.257500	-0.163500
H	-1.506500	4.493000	0.680000
H	-0.581300	4.557000	2.689100
H	-0.073400	3.337100	4.782300
H	0.159400	0.918500	4.742300
C	-0.277500	-1.363200	-1.185400
C	0.537800	-2.358000	-1.732600
C	1.580400	-2.991900	-0.865000
H	2.269200	-2.250200	-0.446700
H	2.173700	-3.702200	-1.443600
C	0.275500	-2.841500	-3.013300
C	-0.804400	-2.369600	-3.750400
C	-1.656500	-1.424700	-3.186700
C	-1.391200	-0.931700	-1.915900
H	-2.048300	-0.184600	-1.482800
H	-2.522200	-1.069700	-3.734400
H	-0.991200	-2.752200	-4.747600
H	0.924000	-3.605700	-3.430400
H	-0.007300	-4.257500	-0.163500
H	1.506500	-4.493000	0.680000

Table S33. The optimized geometric structure of **6** in MeCN at the ground state (**S_0**) performed at M062X/6-311G(d) using the Gaussian 09 package.[33]

**Atom**	**x**	**y**	**z**
C	4.877600	-0.454800	-0.641100
H	5.809100	-0.281600	-1.185300
H	4.862500	-1.501100	-0.324100
C	4.877600	0.454800	0.641100
H	5.809100	0.281600	1.185300
H	4.862500	1.501100	0.324100
C	3.682500	0.122400	1.500500
C	2.424500	0.613800	1.146600

C	1.261700	-0.084200	1.488600
C	0.000000	0.000000	0.685400
C	0.000000	0.000000	-0.685400
C	1.261700	0.084200	-1.488600
C	2.424500	-0.613800	-1.146600
H	2.360600	-1.431600	-0.436000
C	3.682500	-0.122400	-1.500500
C	3.753700	0.909100	-2.440800
H	4.723300	1.299900	-2.734600
C	2.591900	1.464400	-2.966900
H	2.654500	2.247600	-3.714300
C	1.352300	1.088900	-2.456700
H	0.456400	1.620300	-2.761400
C	-1.261700	-0.084200	-1.488600
C	-2.424500	0.613800	-1.146600
H	-2.360600	1.431600	-0.436000
C	-3.682500	0.122400	-1.500500
C	-4.877600	0.454800	-0.641100
H	-5.809100	0.281600	-1.185300
H	-4.862500	1.501100	-0.324100
C	-4.877600	-0.454800	0.641100
H	-5.809100	-0.281600	1.185300
H	-4.862500	-1.501100	0.324100
C	-3.682500	-0.122400	1.500500
C	-2.424500	-0.613800	1.146600
C	-1.261700	0.084200	1.488600
C	-1.352300	1.088900	2.456700
C	-2.591900	1.464400	2.966900
C	-3.753700	0.909100	2.440800
H	-4.723300	1.299900	2.734600
H	-2.654500	2.247600	3.714300
H	-0.456400	1.620300	2.761400
H	-2.360600	-1.431600	0.436000
C	-3.753700	-0.909100	-2.440800
H	-4.723300	-1.299900	-2.734600
C	-2.591900	-1.464400	-2.966900
H	-2.654500	-2.247600	-3.714300
C	-1.352300	-1.088900	-2.456700
H	-0.456400	-1.620300	-2.761400
C	1.352300	-1.088900	2.456700
C	2.591900	-1.464400	2.966900
C	3.753700	-0.909100	2.440800
H	4.723300	-1.299900	2.734600
H	2.654500	-2.247600	3.714300
H	0.456400	-1.620300	2.761400
H	2.360600	1.431600	0.436000

Table S34. The optimized geometric structure of **6-IM** in MeCN at the ground state (**S$_0$**) performed at M062X/6-311G(d) using the Gaussian 09 package.[33]

Atom	x	y	z
C	-0.43834400	-0.62905400	5.02521900
H	-0.23357500	-1.24391100	5.90405400
H	-1.49058800	-0.31867800	5.07903600
C	0.43834400	0.62905400	5.02521900
H	0.23357500	1.24391100	5.90405400
H	1.49058800	0.31867800	5.07903600
C	0.23854700	1.42899300	3.77011900
C	0.38787900	0.65576500	2.48172800

C	0.07646000	1.42228700	1.20857100
C	0.00009300	0.74560100	0.02205400
C	-0.00009300	-0.74560100	0.02205400
C	-0.07646000	-1.42228700	1.20857100
C	-0.38787900	-0.65576500	2.48172800
H	-1.46298300	-0.38433800	2.42031300
C	-0.23854700	-1.42899300	3.77011900
C	-0.04830800	-2.75377700	3.75721500
H	0.01162000	-3.30351500	4.69172300
C	0.08011200	-3.48720000	2.50485000
H	0.23289900	-4.56005200	2.54788000
C	0.05284600	-2.86415200	1.30946500
H	0.21087300	-3.43495200	0.40228100
C	-0.18930000	-1.48610000	-1.26819000
C	0.52642300	-1.19687400	-2.43538300
H	1.41633200	-0.57768300	-2.37333000
C	0.00009300	-1.49330300	-3.69759700
C	0.40676500	-0.67127300	-4.89465000
H	0.19529500	-1.20565200	-5.82398800
H	1.47355200	-0.43247500	-4.87744700
C	-0.40676500	0.67127300	-4.89465000
H	-0.19529500	1.20565200	-5.82398800
H	-1.47355200	0.43247500	-4.87744700
C	-0.00009300	1.49330300	-3.69759700
C	-0.52642300	1.19687400	-2.43538300
C	0.18930000	1.48610000	-1.26819000
C	1.32890200	2.29497700	-1.38477800
C	1.75889200	2.75459400	-2.62352100
C	1.12738400	2.31059900	-3.77981200
H	1.54384100	2.55156800	-4.75347900
H	2.63728200	3.38696000	-2.68974300
H	1.91356300	2.51867000	-0.49903600
H	-1.41633200	0.57768300	-2.37333000
C	-1.12738400	-2.31059900	-3.77981200
H	-1.54384100	-2.55156800	-4.75347900
C	-1.75889200	-2.75459400	-2.62352100
H	-2.63728200	-3.38696000	-2.68974300
C	-1.32890200	-2.29497700	-1.38477800
H	-1.91356300	-2.51867000	-0.49903600
C	-0.05284600	2.86415200	1.30946500
C	-0.08011200	3.48720000	2.50485000
C	0.04830800	2.75377700	3.75721500
H	-0.01162000	3.30351500	4.69172300
H	-0.23289900	4.56005200	2.54788000
H	-0.21087300	3.43495200	0.40228100
H	1.46298300	0.38433800	2.42031300

Table S35. The optimized geometric structure of **6-PC** in MeCN at the ground state ($\mathbf{S_0}$) performed at M062X/6-311G(d) using the Gaussian 09 package.[33]

Atom	x	y	z
C	4.858000	0.641600	0.452500
H	4.849700	0.328300	1.499900
H	5.785100	1.191200	0.272300
C	3.655800	1.490400	0.120600
C	2.402700	1.144800	0.635500

C	1.230600	1.481200	-0.046000
C	-0.034400	0.685800	0.081900
C	-0.034400	-0.685900	-0.082100
C	1.230500	-1.481400	0.046000
C	2.402600	-1.145300	-0.635700
H	2.350900	-0.447200	-1.465700
C	3.655700	-1.490700	-0.120700
C	4.857900	-0.641900	-0.452900
H	4.849400	-0.328700	-1.500400
H	5.785000	-1.191500	-0.272900
C	3.715700	-2.401900	0.934700
H	4.681200	-2.685300	1.343200
C	2.547700	-2.899500	1.503200
H	2.600000	-3.613200	2.317800
C	1.314600	-2.399300	1.098900
H	0.416300	-2.676600	1.640900
C	-1.287900	-1.394400	-0.272000
C	-2.513300	-0.698400	-0.187800
C	-2.513300	0.698400	0.187800
C	-1.287900	1.394300	0.271900
C	-1.312400	2.760800	0.634400
C	-2.504000	3.394900	0.905100
C	-3.714200	2.687000	0.845900
C	-3.729600	1.350600	0.504900
C	-4.997700	0.534700	0.543300
H	-5.868100	1.186700	0.447400
H	-5.068800	0.049100	1.524700
C	-4.997800	-0.534600	-0.543100
H	-5.868200	-1.186600	-0.447200
H	-5.068900	-0.049000	-1.524500
C	-3.729600	-1.350500	-0.504900
C	-3.714300	-2.687000	-0.845900
H	-4.645900	-3.188200	-1.088800
C	-2.504100	-3.394900	-0.905300
H	-2.506200	-4.442200	-1.186100
C	-1.312500	-2.760800	-0.634700
H	-0.381400	-3.306300	-0.722800
H	-4.645700	3.188300	1.088800
H	-2.506000	4.442300	1.185800
H	-0.381200	3.306300	0.722400
C	1.314600	2.399700	-1.098500
C	2.547700	2.900000	-1.502700
C	3.715700	2.402100	-0.934400
H	4.681200	2.685700	-1.342900
H	2.600000	3.614000	-2.316900
H	0.416300	2.677200	-1.640200
H	2.351000	0.446200	1.465100

Table S36. The optimized geometric structure of **1** in THF at the ground state (**S_0**) performed at M062X/6-311G(d) using the Gaussian 09 package.[33]

Atom	x	y	z
C	0.000000	0.675500	-0.000000
C	0.000000	-0.675500	0.000000
C	-1.253600	1.481600	0.064800
C	-1.451800	2.516500	-0.854500
C	-2.229700	1.250600	1.038000
C	-2.614200	3.277700	-0.827700

H	-0.690100	2.715400	-1.602000
C	-3.386200	2.021800	1.075500
H	-2.079400	0.460600	1.766300
C	-3.585900	3.032100	0.138400
H	-2.759500	4.065200	-1.558700
H	-4.133000	1.834800	1.839100
H	-4.490400	3.629400	0.165100
C	1.253600	1.481600	-0.064900
C	2.229800	1.250500	-1.038000
C	1.451700	2.516600	0.854300
C	3.386300	2.021700	-1.075500
H	2.079500	0.460500	-1.766200
C	2.614200	3.277800	0.827400
H	0.690000	2.715500	1.601800
C	3.585900	3.032100	-0.138600
H	4.133100	1.834700	-1.839200
H	2.759500	4.065400	1.558400
H	4.490400	3.629500	-0.165300
C	1.253600	-1.481600	0.064900
C	1.451800	-2.516600	-0.854300
C	2.229700	-1.250500	1.038100
C	2.614200	-3.277800	-0.827400
H	0.690100	-2.715500	-1.601800
C	3.386200	-2.021800	1.075700
H	2.079400	-0.460500	1.766400
C	3.585900	-3.032100	0.138700
H	2.759500	-4.065400	-1.558400
H	4.132900	-1.834800	1.839400
H	4.490300	-3.629500	0.165500
C	-1.253600	-1.481600	-0.064800
C	-1.451800	-2.516500	0.854400
C	-2.229700	-1.250500	-1.038000
C	-2.614200	-3.277700	0.827500
H	-0.690100	-2.715400	1.601900
C	-3.386200	-2.021800	-1.075600
H	-2.079400	-0.460600	-1.766300
C	-3.585900	-3.032100	-0.138600
H	-2.759500	-4.065300	1.558500
H	-4.133000	-1.834800	-1.839300
H	-4.490300	-3.629400	-0.165300

Table S37. The optimized geometric structure of **1** in THF at the first excited state (**S_1**) performed at M062X/6-311G(d) using the Gaussian 09 package.[33]

Atom	x	y	z
C	0.002800	0.734900	-0.000900
C	-0.002800	-0.735000	-0.000900
C	-1.080300	1.418400	0.661300
C	-1.498200	2.723200	0.296100
C	-1.837300	0.752000	1.656800
C	-2.576900	3.328200	0.917200
H	-0.997200	3.235100	-0.516900
C	-2.901900	1.373700	2.286600
H	-1.557700	-0.257000	1.942100
C	-3.279700	2.666900	1.925900
H	-2.884900	4.319600	0.604000
H	-3.447800	0.845400	3.060400

H	-4.121700	3.147100	2.410900
C	1.091300	1.410400	-0.662400
C	1.843900	0.738900	-1.658000
C	1.518900	2.711700	-0.296100
C	2.913500	1.353000	-2.286700
H	1.556800	-0.267700	-1.944200
C	2.602500	3.308900	-0.916100
H	1.021400	3.226700	0.517100
C	3.300900	2.643000	-1.924800
H	3.456000	0.821100	-3.060500
H	2.917900	4.297700	-0.601900
H	4.146900	3.117100	-2.408900
C	1.080300	-1.418400	0.661300
C	1.498200	-2.723300	0.296300
C	1.837400	-0.751900	1.656800
C	2.576900	-3.328200	0.917500
H	0.997200	-3.235200	-0.516600
C	2.902000	-1.373600	2.286600
H	1.557800	0.257100	1.942000
C	3.279700	-2.666800	1.926000
H	2.884900	-4.319700	0.604300
H	3.447900	-0.845200	3.060400
H	4.121700	-3.147000	2.411100
C	-1.091400	-1.410500	-0.662300
C	-1.518900	-2.711700	-0.296000
C	-1.843900	-0.738900	-1.657900
C	-2.602600	-3.308900	-0.916000
H	-1.021400	-3.226800	0.517200
C	-2.913600	-1.353000	-2.286500
H	-1.556800	0.267600	-1.944100
C	-3.301000	-2.643000	-1.924600
H	-2.918000	-4.297700	-0.601700
H	-3.456000	-0.821100	-3.060400
H	-4.147000	-3.117100	-2.408700

Table S38. The optimized geometric structure of **2** in THF at the ground state (**S_0**) performed at M062X/6-311G(d) using the Gaussian 09 package.[33]

Atom	x	y	z
C	-2.814500	-1.800100	-3.964400
C	-1.852300	-2.018000	-2.824800
C	-1.369100	-0.952400	-2.074300
C	-0.486500	-1.160500	-1.007000
C	0.000000	0.000000	-0.206600
C	0.000000	0.000000	1.147100
C	-0.670200	-1.064200	1.948600
C	-1.981100	-1.463300	1.669900
H	-2.516000	-0.992500	0.852000
C	-2.595300	-2.456700	2.422700
H	-3.613200	-2.752300	2.194600
C	-1.907800	-3.069300	3.467500
H	-2.385500	-3.847500	4.052000
C	-0.608300	-2.668600	3.764600
H	-0.068400	-3.135300	4.580900
C	-0.000000	-1.665100	3.018700
H	1.010300	-1.348800	3.258000
C	0.670200	1.064200	1.948600
C	0.000000	1.665100	3.018700
H	-1.010300	1.348800	3.258000
C	0.608300	2.668600	3.764600

H	0.068400	3.135300	4.580900
C	1.907800	3.069300	3.467500
H	2.385500	3.847500	4.052000
C	2.595300	2.456700	2.422700
H	3.613200	2.752300	2.194600
C	1.981100	1.463300	1.669900
H	2.516000	0.992500	0.852000
C	0.486500	1.160500	-1.007000
C	1.369100	0.952400	-2.074300
H	1.687300	-0.059500	-2.309100
C	1.852300	2.018000	-2.824800
C	1.421300	3.310300	-2.507300
H	1.792300	4.149200	-3.091200
C	0.523600	3.544900	-1.470200
C	0.061100	2.456800	-0.727400
H	-0.634800	2.624100	0.089700
C	0.054400	4.938100	-1.137000
H	0.317300	5.201000	-0.109500
H	-1.031900	5.017000	-1.224200
H	0.501400	5.678700	-1.801000
C	2.814500	1.800100	-3.964400
H	2.374000	2.116500	-4.913200
H	3.091400	0.749400	-4.055700
H	3.729000	2.380700	-3.822100
C	-0.061100	-2.456800	-0.727400
C	-0.523600	-3.544900	-1.470200
C	-1.421300	-3.310300	-2.507300
H	-1.792300	-4.149200	-3.091200
C	-0.054400	-4.938100	-1.137000
H	1.031900	-5.017000	-1.224200
H	-0.501400	-5.678700	-1.801000
H	-0.317300	-5.201000	-0.109500
H	0.634800	-2.624100	0.089700
H	-1.687300	0.059500	-2.309100
H	-2.374000	-2.116500	-4.913200
H	-3.091400	-0.749400	-4.055700
H	-3.729000	-2.380700	-3.822100

Table S39. The optimized geometric structure of **2** in THF at the first excited state (**S_1**) performed at M062X/6-311G(d) using the Gaussian 09 package.[33]

Atom	x	y	z
C	-3.007900	4.291800	0.522700
C	-2.710800	2.929800	-0.050600
C	-1.508400	2.294500	0.201500
C	-1.225400	1.002300	-0.314300
C	0.000000	0.306900	0.000300
C	-0.000200	-1.160900	0.000200
C	-1.040700	-1.855700	0.719200
C	-1.729900	-1.222400	1.784200
H	-1.421800	-0.229500	2.092900
C	-2.761700	-1.859800	2.453000
H	-3.255900	-1.356100	3.276600
C	-3.173400	-3.135200	2.069400
H	-3.991100	-3.626000	2.584600
C	-2.535700	-3.765600	0.998200
H	-2.869700	-4.743500	0.668900
C	-1.491400	-3.145200	0.336700

H	-1.041400	-3.630800	-0.521300
C	1.039900	-1.855800	-0.719100
C	1.490000	-3.145700	-0.337300
H	1.039800	-3.631600	0.520500
C	2.534000	-3.766300	-0.999000
H	2.867500	-4.744500	-0.670300
C	3.172000	-3.135600	-2.069900
H	3.989600	-3.626500	-2.585400
C	2.761000	-1.859700	-2.452800
H	3.255400	-1.355800	-3.276100
C	1.729400	-1.222200	-1.783700
H	1.421900	-0.229000	-2.091800
C	1.225800	1.001800	0.314700
C	1.509200	2.293900	-0.201000
H	0.786900	2.771800	-0.853500
C	2.711800	2.928800	0.050700
C	3.672700	2.278600	0.840400
H	4.620000	2.774100	1.037400
C	3.446300	1.001900	1.350600
C	2.238800	0.370300	1.070500
H	2.058600	-0.625400	1.465000
C	4.482200	0.302900	2.193900
H	4.680900	-0.701400	1.813000
H	4.137200	0.198000	3.225600
H	5.422600	0.854700	2.208500
C	3.009400	4.290600	-0.522500
H	3.168200	5.022100	0.273900
H	2.190300	4.646000	-1.148500
H	3.917500	4.270600	-1.129700
C	-2.238500	0.371300	-1.070400
C	-3.445600	1.003300	-1.350700
C	-3.671600	2.280100	-0.840500
H	-4.618700	2.776100	-1.037700
C	-4.481700	0.304700	-2.194300
H	-4.135900	0.198400	-3.225600
H	-5.421400	0.857700	-2.210200
H	-4.681800	-0.698900	-1.812600
H	-2.058600	-0.624500	-1.465000
H	-0.786100	2.772100	0.854200
H	-3.166300	5.023300	-0.273800
H	-2.188800	4.646800	1.148800
H	-3.916100	4.272100	1.129700

Table S40. The optimized geometric structure of **3** in THF at the ground state (**S$_0$**) performed at M062X/6-311G(d) using the Gaussian 09 package.[33]

Atom	x	y	z
C	-0.551000	-2.399100	-2.148100
C	-1.493500	-2.207300	-0.984600
C	-1.269200	-1.238200	0.018200
C	0.000100	-0.434600	0.000000
C	-0.000000	0.920200	-0.000000
C	-1.245700	1.718300	-0.181000
C	-2.187800	1.386900	-1.159500
H	-2.001500	0.542800	-1.814400
C	-3.355700	2.127400	-1.301300
H	-4.074900	1.854600	-2.065500
C	-3.599200	3.216900	-0.470000
H	-4.512900	3.790700	-0.577000

C	-2.656900	3.573400	0.490900
H	-2.834400	4.425700	1.137300
C	-1.487000	2.836200	0.626100
H	-0.756300	3.115400	1.378700
C	1.245500	1.718500	0.180900
C	2.187700	1.387500	1.159400
H	2.001600	0.543300	1.814300
C	3.355300	2.128300	1.301200
H	4.074600	1.855600	2.065400
C	3.598500	3.217800	0.469900
H	4.512100	3.791900	0.577000
C	2.656100	3.574000	-0.491000
H	2.833400	4.426400	-1.137400
C	1.486400	2.836500	-0.626200
H	0.755700	3.115500	-1.378800
C	1.269500	-1.238000	-0.018100
C	1.493800	-2.207200	0.984700
C	2.639000	-2.998200	0.934200
H	2.807100	-3.732100	1.716100
C	3.565000	-2.855200	-0.090900
H	4.450500	-3.480600	-0.120700
C	3.347400	-1.900000	-1.070200
H	4.066300	-1.779000	-1.875100
C	2.215400	-1.081800	-1.051900
C	2.091200	-0.079500	-2.174200
H	1.092700	0.342800	-2.269600
H	2.360600	-0.548100	-3.123600
H	2.778900	0.755200	-2.010100
C	0.551500	-2.398800	2.148300
H	0.192900	-1.445800	2.540700
H	1.057500	-2.926700	2.957700
H	-0.329200	-2.980900	1.864400
C	-2.215100	-1.082000	1.052000
C	-3.347200	-1.900200	1.070200
C	-3.564800	-2.855100	0.090700
C	-2.638800	-2.998200	-0.934400
H	-2.806900	-3.731900	-1.716400
H	-4.450500	-3.480400	0.120300
H	-4.066000	-1.779200	1.875100
C	-2.090800	-0.079900	2.174500
H	-1.092300	0.342700	2.269700
H	-2.359700	-0.548800	3.123800
H	-2.778800	0.754600	2.010800
H	-0.192800	-1.446000	-2.540900
H	-1.056700	-2.927500	-2.957300
H	0.329900	-2.980700	-1.863900

Table S41. The optimized geometric structure of **3** in THF at the first excited state (**S_1**) performed at M062X/6-311G(d) using the Gaussian 09 package.[33]

Atom	x	y	z
C	-0.982000	-2.845900	-1.559800
C	-1.662200	-2.335000	-0.313200
C	-1.185500	-1.213800	0.437600
C	-0.000000	-0.496800	-0.000000
C	-0.000000	0.964200	0.000000
C	-1.166300	1.665700	-0.489800
C	-2.066300	1.045100	-1.393300

H	-1.850600	0.047700	-1.757100
C	-3.203000	1.698800	-1.843700
H	-3.859100	1.201000	-2.549200
C	-3.510200	2.979000	-1.391000
H	-4.407200	3.482200	-1.733100
C	-2.660300	3.599300	-0.470600
H	-2.906400	4.582000	-0.083400
C	-1.515500	2.962600	-0.030200
H	-0.898600	3.442100	0.721000
C	1.166300	1.665700	0.489800
C	2.066200	1.044900	1.393400
H	1.850400	0.047600	1.757200
C	3.203000	1.698600	1.843800
H	3.859000	1.200800	2.549400
C	3.510300	2.978700	1.391100
H	4.407300	3.481900	1.733200
C	2.660500	3.599100	0.470600
H	2.906700	4.581800	0.083400
C	1.515600	2.962500	0.030200
H	0.898800	3.442000	-0.721000
C	1.185400	-1.213800	-0.437700
C	1.662100	-2.334900	0.313100
C	2.850500	-2.949400	-0.064200
H	3.221000	-3.779200	0.529400
C	3.578700	-2.518400	-1.167200
H	4.500200	-3.018600	-1.443300
C	3.110100	-1.444300	-1.914800
H	3.657500	-1.123400	-2.795600
C	1.936500	-0.783100	-1.574800
C	1.438700	0.300100	-2.496800
H	0.362200	0.222500	-2.667700
H	1.941900	0.229800	-3.462100
H	1.627200	1.299000	-2.096200
C	0.982000	-2.845800	1.559700
H	0.645000	-2.030000	2.204400
H	1.672700	-3.467800	2.130100
H	0.098500	-3.449200	1.333200
C	-1.936500	-0.783100	1.574700
C	-3.110200	-1.444300	1.914700
C	-3.578800	-2.518400	1.167100
C	-2.850500	-2.949500	0.064200
H	-3.220900	-3.779300	-0.529400
H	-4.500200	-3.018700	1.443200
H	-3.657600	-1.123500	2.795500
C	-1.438700	0.300000	2.496800
H	-0.362200	0.222500	2.667500
H	-1.941800	0.229500	3.462200
H	-1.627500	1.299000	2.096400
H	-0.645100	-2.030100	-2.204500
H	-1.672600	-3.468100	-2.130000
H	-0.098300	-3.449100	-1.333200

Table S42. The optimized geometric structure of **4** in THF at the ground state (**S$_0$**) performed at M062X/6-311G(d) using the Gaussian 09 package.[33]

Atom	x	y	z
C	0.965600	-2.867100	1.505200
C	1.766400	-2.250900	0.367600

C	1.379900	-1.149300	-0.414000
C	0.064500	-0.456300	-0.258400
C	-0.059800	0.872400	-0.085800
C	1.092300	1.799400	0.115700
C	2.141700	1.482300	0.985400
C	3.198500	2.364900	1.173100
C	3.226400	3.580600	0.495000
C	2.181100	3.913400	-0.360500
C	1.117700	3.035400	-0.537800
H	0.299000	3.310900	-1.193900
H	2.188600	4.861300	-0.887000
H	4.052100	4.267900	0.639900
H	4.001200	2.104100	1.853700
H	2.126100	0.537500	1.517900
C	-1.410500	1.512700	-0.005900
C	-2.001400	1.718400	1.242000
C	-3.253300	2.316100	1.341700
C	-3.923500	2.728700	0.193700
C	-3.335300	2.541000	-1.053300
C	-2.086000	1.935700	-1.151400
H	-1.631200	1.784700	-2.125600
H	-3.851100	2.860600	-1.951900
H	-4.899300	3.194700	0.271700
H	-3.705800	2.459600	2.316800
H	-1.474700	1.402200	2.137300
C	-1.126900	-1.353800	-0.320600
C	-1.990000	-1.370000	-1.414100
C	-3.081600	-2.232300	-1.437200
C	-3.311500	-3.086100	-0.363900
C	-2.444100	-3.080100	0.724800
C	-1.347500	-2.221000	0.757100
C	-0.363700	-2.214300	1.896400
H	-0.187700	-1.186700	2.231200
H	-0.775600	-2.761000	2.747600
H	-2.622800	-3.747700	1.562500
H	-4.163500	-3.756700	-0.373900
H	-3.749400	-2.235500	-2.291300
H	-1.802100	-0.706400	-2.250800
C	2.233400	-0.698200	-1.431500
C	3.455900	-1.301200	-1.675700
C	3.850100	-2.384100	-0.893200
C	3.008700	-2.842400	0.108000
H	3.314900	-3.688500	0.717200
H	4.804600	-2.868900	-1.065400
H	4.095500	-0.932800	-2.469600
H	1.917000	0.145700	-2.035600
H	0.769600	-3.913900	1.246200
H	1.611600	-2.894500	2.388700

Table S43. The optimized geometric structure of **5** in THF at the ground state (**S_0**) performed at M062X/6-311G(d) using the Gaussian 09 package.[33]

Atom	x	y	z
C	-0.788900	0.861300	3.040000
H	-0.760800	0.863000	4.132000
H	0.251100	0.884500	2.694800
C	-1.463100	-0.390900	2.546200

C	-1.344200	-0.722500	1.191200
C	-0.573000	0.189900	0.298100
C	-1.210900	1.523200	0.053900
C	-1.640800	2.379000	1.082000
C	-2.252500	3.588400	0.727600
H	-2.572900	4.255300	1.523300
C	-2.461800	3.953800	-0.591900
H	-2.940800	4.898000	-0.825800
C	-2.061500	3.090900	-1.608800
H	-2.230400	3.345500	-2.648900
C	-1.447600	1.894600	-1.279100
H	-1.145800	1.217100	-2.069700
C	-1.508300	2.129800	2.576300
H	-1.004000	2.995900	3.016500
H	-2.518500	2.124700	3.001200
C	0.573000	-0.189900	-0.298100
C	1.210900	-1.523200	-0.053900
C	1.640800	-2.379000	-1.082000
C	1.508300	-2.129800	-2.576300
C	0.788900	-0.861300	-3.040000
H	0.760800	-0.863000	-4.132000
H	-0.251100	-0.884500	-2.694800
C	1.463100	0.390900	-2.546200
C	1.344200	0.722500	-1.191200
C	1.946600	1.877800	-0.698300
H	1.836800	2.126000	0.352800
C	2.671700	2.707700	-1.547000
H	3.136700	3.606500	-1.157800
C	2.794400	2.382400	-2.893900
H	3.357200	3.026600	-3.560200
C	2.189000	1.230100	-3.388200
H	2.274300	0.983200	-4.442200
H	1.004000	-2.995900	-3.016500
H	2.518500	-2.124700	-3.001200
C	2.252500	-3.588400	-0.727600
H	2.572900	-4.255300	-1.523300
C	2.461800	-3.953800	0.591900
H	2.940800	-4.898000	0.825800
C	2.061500	-3.090900	1.608800
H	2.230400	-3.345500	2.648900
C	1.447600	-1.894600	1.279100
H	1.145800	-1.217100	2.069700
C	-1.946600	-1.877800	0.698300
H	-1.836800	-2.126000	-0.352800
C	-2.671700	-2.707700	1.547000
H	-3.136700	-3.606500	1.157800
C	-2.794400	-2.382400	2.893900
H	-3.357200	-3.026600	3.560200
C	-2.189000	-1.230100	3.388200
H	-2.274300	-0.983200	4.442200

Table S44. The optimized geometric structure of **5** in THF at the first excited state (**S_1**) performed at M062X/6-311G(d) using the Gaussian 09 package.[33]

Atom	x	y	z
C	3.043300	0.571800	1.576200
H	3.779700	1.062800	2.216600
H	2.152800	0.384200	2.187600

C	2.681900	1.461200	0.423500
C	1.514600	1.159100	-0.311700
C	0.730600	-0.046400	-0.045600
C	1.402300	-1.325300	-0.206200
C	2.737300	-1.627000	0.221900
C	3.296100	-2.854700	-0.114700
H	4.298500	-3.071000	0.245000
C	2.628700	-3.814500	-0.871100
H	3.109700	-4.756100	-1.109500
C	1.338200	-3.535300	-1.311000
H	0.796800	-4.245900	-1.925500
C	0.740100	-2.338000	-0.974700
H	-0.236400	-2.125700	-1.385500
C	3.620500	-0.753900	1.095700
H	3.918800	-1.346300	1.967700
H	4.545100	-0.549400	0.543900
C	-0.730600	0.046400	0.045700
C	-1.402300	1.325300	0.206300
C	-2.737300	1.627000	-0.221800
C	-3.620400	0.754000	-1.095700
C	-3.043300	-0.571800	-1.576200
H	-3.779700	-1.062700	-2.216600
H	-2.152800	-0.384100	-2.187500
C	-2.681900	-1.461100	-0.423500
C	-1.514600	-1.159100	0.311700
C	-1.104200	-2.056200	1.320600
H	-0.199000	-1.844600	1.878600
C	-1.866900	-3.169700	1.638500
H	-1.546000	-3.828800	2.437300
C	-3.031700	-3.444800	0.926900
H	-3.621600	-4.324100	1.159400
C	-3.420100	-2.594600	-0.108700
H	-4.302200	-2.830600	-0.696900
H	-3.918700	1.346400	-1.967700
H	-4.545100	0.549500	-0.544000
C	-3.296000	2.854800	0.114800
H	-4.298400	3.071100	-0.245000
C	-2.628700	3.814500	0.871100
H	-3.109600	4.756100	1.109500
C	-1.338200	3.535300	1.311000
H	-0.796700	4.245900	1.925500
C	-0.740100	2.338000	0.974700
H	0.236400	2.125700	1.385500
C	1.104200	2.056200	-1.320600
H	0.199000	1.844500	-1.878600
C	1.866800	3.169600	-1.638600
H	1.546000	3.828700	-2.437400
C	3.031600	3.444800	-0.927000
H	3.621500	4.324100	-1.159600
C	3.420100	2.594600	0.108600
H	4.302100	2.830700	0.696800

Table S45. The optimized geometric structure of **6** in THF at the ground state (**S_0**) performed at M062X/6-311G(d) using the Gaussian 09 package.[33]

Atom	x	y	z
C	4.876700	-0.639700	0.456600
H	5.808400	-1.184800	0.285900
H	4.861200	-0.319700	1.502000
C	4.876600	0.639600	-0.457000

H	5.808300	1.184700	-0.286600
H	4.860900	0.319400	-1.502300
C	3.681800	1.500600	-0.127300
C	2.423800	1.144700	-0.616500
C	1.261600	1.489000	0.081300
C	-0.000000	0.685500	0.000300
C	0.000000	-0.685300	0.000300
C	1.261600	-1.488900	-0.081100
C	2.423900	-1.144800	0.616500
H	2.359400	-0.431500	1.431800
C	3.681900	-1.500800	0.127100
C	3.753500	-2.444800	-0.900700
H	4.723400	-2.740800	-1.289400
C	2.592100	-2.973000	-1.454400
H	2.654900	-3.724100	-2.234000
C	1.352500	-2.461100	-1.081700
H	0.456300	-2.768100	-1.611100
C	-1.261600	-1.488900	0.081400
C	-2.423800	-1.144900	-0.616500
H	-2.359000	-0.431500	-1.431800
C	-3.681800	-1.500700	-0.127300
C	-4.876500	-0.639700	-0.457100
H	-5.808300	-1.184800	-0.286700
H	-4.860800	-0.319600	-1.502500
C	-4.876700	0.639700	0.456400
H	-5.808400	1.184700	0.285800
H	-4.861300	0.319500	1.501800
C	-3.681900	1.500700	0.127100
C	-2.424000	1.144700	0.616500
C	-1.261600	1.489000	-0.080900
C	-1.352500	2.461200	-1.081500
C	-2.592100	2.973000	-1.454200
C	-3.753500	2.444800	-0.900600
H	-4.723400	2.740700	-1.289400
H	-2.654900	3.724100	-2.233800
H	-0.456300	2.768300	-1.610800
H	-2.359400	0.431100	1.431600
C	-3.753700	-2.444600	0.900500
H	-4.723700	-2.740600	1.289000
C	-2.592400	-2.972800	1.454500
H	-2.655400	-3.723800	2.234200
C	-1.352700	-2.461000	1.082000
H	-0.456600	-2.768000	1.611700
C	1.352700	2.461100	1.081900
C	2.592400	2.972900	1.454400
C	3.753700	2.444600	0.900400
H	4.723700	2.740500	1.289000
H	2.655400	3.723900	2.234000
H	0.456600	2.768200	1.611500
H	2.359000	0.431000	-1.431500

Table S46. The optimized geometric structure of **6** in THF at the first excited state (**S_1**) performed at M062X/6-311G(d) using the Gaussian 09 package.[33]

Atom	x	y	z
C	-4.982800	-0.670600	-0.418600
H	-5.876100	-1.251900	-0.181100
H	-5.018000	-0.412200	-1.482000

C	-4.987000	0.666400	0.397800
H	-5.878700	1.247100	0.153000
H	-5.031100	0.407700	1.460800
C	-3.748300	1.431700	0.072400
C	-2.470400	0.763600	0.372800
C	-1.237100	1.425600	-0.116000
C	0.016400	0.697800	-0.062600
C	0.016800	-0.695500	0.071100
C	-1.238500	-1.423700	0.122200
C	-2.467800	-0.762700	-0.375200
H	-2.387000	-0.457600	-1.425600
C	-3.745700	-1.432900	-0.081400
C	-3.766800	-2.600900	0.631100
H	-4.722200	-3.058000	0.872600
C	-2.572700	-3.234100	1.044500
H	-2.618400	-4.173700	1.581400
C	-1.341700	-2.637400	0.795700
H	-0.438300	-3.103500	1.174800
C	1.277700	-1.497000	0.043400
C	2.442700	-1.092400	0.705000
H	2.378300	-0.318300	1.462900
C	3.702000	-1.483700	0.244500
C	4.896900	-0.599300	0.501600
H	5.828500	-1.157300	0.379300
H	4.880500	-0.190000	1.515300
C	4.894000	0.595400	-0.519100
H	5.827500	1.151700	-0.403800
H	4.869100	0.186200	-1.532800
C	3.702500	1.481600	-0.252800
C	2.439600	1.094500	-0.707700
C	1.279300	1.497500	-0.037100
C	1.388400	2.530500	0.902500
C	2.630500	3.064100	1.228400
C	3.786400	2.498100	0.701000
H	4.760300	2.816600	1.060700
H	2.700300	3.864000	1.957400
H	0.500500	2.871500	1.424000
H	2.369400	0.322800	-1.467600
C	3.778400	-2.504500	-0.705600
H	4.749700	-2.826300	-1.069600
C	2.618700	-3.071300	-1.223300
H	2.682800	-3.874800	-1.948700
C	1.379200	-2.534700	-0.892000
H	0.487700	-2.876700	-1.406300
C	-1.338400	2.648700	-0.775200
C	-2.569000	3.242400	-1.028700
C	-3.767000	2.603700	-0.633100
H	-4.720900	3.059900	-0.881600
H	-2.612300	4.185900	-1.559000
H	-0.433800	3.122300	-1.141800
H	-2.396400	0.460500	1.424200

Table S47. The optimized geometric structure of **1** in the QM region at the ground state (**S_0**) in solid performed at M062X/6-311G(d) using the Gaussian 09 package.[33]

Atom	x	y	z
C	2.706900	2.159500	2.925700
C	2.392100	1.739400	7.920700
C	1.698900	0.547400	7.701900

C	0.578600	0.234400	8.462900
C	3.604600	2.067400	7.117400
C	4.785600	2.343900	7.712400
C	5.255700	1.965600	11.964100
C	5.701500	3.104000	11.298500
C	5.563600	3.211500	9.920800
C	4.972600	2.183700	9.182800
C	4.559800	1.031400	9.854600
C	1.880600	2.786200	3.849900
C	4.686000	0.927100	11.233400
C	8.203300	3.925300	5.665200
C	6.930500	4.399900	5.371700
C	5.820200	3.898600	6.033200
C	5.960800	2.882100	6.980000
C	7.244700	2.422400	7.282700
C	8.357600	2.943600	6.637000
C	2.199000	2.753100	5.197700
C	3.355800	2.116000	5.650300
C	4.198300	1.521000	4.709200
C	3.875700	1.537400	3.356600
C	0.116900	1.132000	9.422800
C	0.787800	2.334800	9.623800
C	1.923200	2.632600	8.883900
H	2.423300	2.151100	1.882200
H	2.053700	-0.143800	6.945500
H	0.066400	-0.707100	8.305400
H	5.351300	1.878100	13.040000
H	6.148600	3.925300	11.845400
H	5.899000	4.106400	9.409000
H	4.126700	0.220600	9.281000
H	0.979400	3.290400	3.527300
H	4.353500	0.028900	11.737800
H	9.063000	4.339600	5.152800
H	6.798500	5.173700	4.633200
H	4.837700	4.307800	5.831700
H	7.377400	1.665200	8.047000
H	9.338200	2.592400	6.927000
H	1.557300	3.235900	5.923700
H	5.109000	1.043900	5.049400
H	4.537700	1.066200	2.638900
H	-0.770400	0.893700	9.996900
H	0.444300	3.055800	10.353900
H	2.457900	3.560000	9.058200

Table S48. The optimized geometric structure of **1** in the QM region at the first excited state (**S_1**) in solid performed at M062X/6-311G(d) using the Gaussian 09 package.[33]

Atom	x	y	z
C	3.62432700	1.64802600	2.92598700
C	2.40161200	1.93249300	7.87478600
C	1.65541100	0.76123000	7.61135700
C	0.56734100	0.41972500	8.39671700
C	3.63226100	2.16847400	7.13746600
C	4.84606000	2.50028600	7.84753800
C	5.12007500	1.89717400	12.06103800
C	5.66770600	3.02761700	11.44944200
C	5.61788900	3.17799000	10.07902700
C	4.98383800	2.21686300	9.26287000
C	4.41926400	1.08887100	9.89635100

C	2.49014100	2.15553700	3.57423000
C	4.50262300	0.92920500	11.27148500
C	8.18423200	3.91645900	5.62439200
C	6.91237200	4.38046800	5.32277000
C	5.80895900	3.96912000	6.05650300
C	5.96305500	3.09842300	7.16330100
C	7.27690700	2.65871800	7.47017000
C	8.35685600	3.04092000	6.70504000
C	2.48719900	2.30423200	4.94375900
C	3.63300000	1.97523800	5.71713300
C	4.79383600	1.55217200	5.01769500
C	4.77602600	1.36966100	3.64526900
C	0.16640300	1.25650500	9.44066300
C	0.88935200	2.41778400	9.70992900
C	2.00654000	2.73946700	8.95767800
H	3.60916100	1.46507100	1.86735800
H	1.99374100	0.08803600	6.83190100
H	0.03513800	-0.50524700	8.20601900
H	5.15707000	1.78042700	13.13683300
H	6.09587100	3.82982100	12.03662500
H	6.00068000	4.07881700	9.61701900
H	3.94043500	0.33436500	9.28336000
H	1.60747200	2.40928600	2.99829500
H	4.08361000	0.04534000	11.73584400
H	9.03067300	4.23149500	5.02600500
H	6.77484100	5.06131300	4.49823000
H	4.82341500	4.35344400	5.82447400
H	7.41541700	1.95955100	8.28583300
H	9.33538800	2.66077500	6.96523600
H	1.61753300	2.70780200	5.44823900
H	5.67161500	1.30483200	5.59388400
H	5.65920500	1.00269100	3.13389000
H	-0.70232900	0.98936600	10.03064300
H	0.60647500	3.08459300	10.51384100
H	2.57466900	3.63348300	9.18560600

Table S49. The optimized geometric structure of **2** in the QM region at the ground state (**S_0**) in solid performed at M062X/6-311G(d) using the Gaussian 09 package.[33]

Atom	x	y	z
C	3.421200	1.726900	3.338500
C	2.448300	1.774100	2.403000
C	3.305300	2.371600	4.679300
C	3.778300	1.687800	5.804600
H	4.213500	0.701800	5.675100
C	3.704000	2.246400	7.076500
C	3.170000	3.527900	7.210700
H	3.112600	3.967000	8.201900
C	2.701700	4.242500	6.110100
C	2.776500	3.653500	4.848300
H	2.431000	4.206700	3.980800
C	4.191700	1.500900	8.293400
H	5.027000	2.023900	8.767000
H	4.526200	0.494800	8.035700
H	3.400400	1.419600	9.042000
C	2.112100	5.619500	6.285000

H	2.135900	6.185000	5.352300
H	2.656200	6.190800	7.039000
H	1.071900	5.558800	6.616000
C	4.709200	1.021700	3.065900
C	5.923800	1.664900	3.326200
H	5.919300	2.647000	3.788100
C	7.137900	1.073600	2.996600
C	7.123500	-0.206400	2.436600
H	8.065700	-0.687300	2.199700
C	5.933400	-0.891300	2.210800
C	4.730700	-0.264900	2.532700
H	3.795200	-0.786500	2.356900
C	8.440400	1.791900	3.236000
H	9.067500	1.251300	3.946700
H	8.272700	2.792100	3.633600
H	9.015100	1.876900	2.311000
C	5.920900	-2.274900	1.615200
H	5.708500	-2.240700	0.543100
H	5.138400	-2.882000	2.071400
H	6.875500	-2.784100	1.754500
C	1.098000	2.371700	2.614200
C	0.228200	1.919600	3.608100
H	0.569400	1.155800	4.299400
C	-1.063800	2.431400	3.696900
H	-1.749100	2.068200	4.452400
C	-1.492300	3.406100	2.803900
H	-2.507400	3.781100	2.853800
C	-0.619200	3.891300	1.835500
H	-0.939800	4.665200	1.152900
C	0.660100	3.365900	1.733800
H	1.332700	3.724100	0.960700
C	2.619900	1.218000	1.024700
C	1.762400	0.205900	0.584200
H	0.997600	-0.172700	1.255900
C	1.894700	-0.331300	-0.689900
H	1.240300	-1.133100	-1.006200
C	2.861400	0.168200	-1.559100
H	2.952500	-0.231500	-2.562300
C	3.704400	1.191500	-1.138500
H	4.453000	1.585900	-1.815700
C	3.593200	1.703600	0.150100
H	4.267900	2.482100	0.487800

Table S50. The optimized geometric structure of **2** in the QM region at the first excited state (**S$_1$**) in solid performed at M062X/6-311G(d) using the Gaussian 09 package.[33]

Atom	x	y	z
C	3.41718800	1.73427000	3.36524800
C	2.37737200	1.79036200	2.36948800
C	3.31854300	2.45744500	4.60976700
C	3.85441700	1.86645400	5.78673000
H	4.32418300	0.89172400	5.70436600
C	3.72766000	2.46531500	7.02224600
C	3.10401900	3.72286000	7.10153700
H	3.02513100	4.19819700	8.07222600

C	2.56986300	4.34571500	5.97632400
C	2.64669400	3.69871300	4.74499600
H	2.25539700	4.18952500	3.86001000
C	4.21946800	1.79157100	8.27819600
H	4.96482400	2.40372800	8.79289100
H	4.67092200	0.82261400	8.05797200
H	3.39784200	1.63679000	8.98164200
C	1.90948900	5.69764900	6.08614200
H	2.03357900	6.27040100	5.16556000
H	2.33444900	6.28113400	6.90461900
H	0.83835200	5.59870900	6.27769600
C	4.63093900	0.97856800	3.09843000
C	5.88308900	1.56766300	3.40755900
H	5.89682200	2.53336500	3.90122900
C	7.07500100	0.98473200	3.02275700
C	7.03406900	-0.26178100	2.37451800
H	7.96690000	-0.74333000	2.10180800
C	5.82736900	-0.89185200	2.08477900
C	4.63525900	-0.25748100	2.42281900
H	3.69133700	-0.74400400	2.19688300
C	8.39090700	1.67825700	3.26342100
H	9.03014800	1.10597000	3.93705200
H	8.24373700	2.66535200	3.70000300
H	8.94605800	1.79204300	2.32957900
C	5.78184300	-2.23690200	1.40784400
H	5.32984700	-2.15688000	0.41527900
H	5.16806400	-2.93085600	1.98343900
H	6.77640900	-2.67243000	1.30272500
C	1.04331200	2.26996100	2.63525400
C	0.29530400	2.00804100	3.81018500
H	0.72125000	1.36711000	4.57459600
C	-1.00805100	2.47792200	3.92260100
H	-1.61513500	2.21592000	4.77873600
C	-1.56577200	3.29224000	2.94224600
H	-2.58479300	3.64657100	3.03793300
C	-0.79524600	3.67232500	1.84006500
H	-1.18248900	4.38403100	1.12472100
C	0.46804600	3.14949600	1.67719600
H	1.08202700	3.46827200	0.84460200
C	2.61457400	1.34292000	1.00021100
C	1.72144700	0.38836700	0.45712800
H	0.90805500	0.03062200	1.07809200
C	1.92192600	-0.14951400	-0.80206700
H	1.25902300	-0.92279300	-1.16941500
C	2.97337000	0.31208500	-1.59580700
H	3.11054100	-0.07129700	-2.60007500
C	3.85227200	1.26624300	-1.08640800
H	4.64825000	1.64520100	-1.71354200
C	3.71539200	1.73236900	0.21253600
H	4.42612400	2.44436900	0.61289300

Table S51. The optimized geometric structure of **3** in the QM region at the ground state ($\mathbf{S_0}$) in solid performed at M062X/6-311G(d) using the Gaussian 09 package.[33]

Atom	x	y	z
C	6.327500	2.620500	3.581400

C	5.153000	2.493000	2.914000
C	6.354300	2.719500	5.079900
C	7.104300	1.810500	5.856200
C	7.083400	1.913400	7.244100
H	7.651800	1.197700	7.827100
C	6.344700	2.902000	7.879200
H	6.331400	2.976900	8.959700
C	5.635000	3.809800	7.113800
H	5.066600	4.600700	7.589000
C	5.625400	3.741000	5.721700
C	7.891300	0.683300	5.237700
H	7.277300	0.088200	4.557700
H	8.268400	0.014300	6.011600
H	8.737600	1.058100	4.659600
C	4.816400	4.794200	5.006600
H	5.029400	4.871400	3.943300
H	4.996600	5.769100	5.461000
H	3.750800	4.571500	5.102800
C	7.640700	2.685300	2.861200
C	8.496000	3.794000	3.031700
C	9.715700	3.826700	2.362500
H	10.355400	4.691600	2.490300
C	10.105900	2.790800	1.524200
H	11.045300	2.847100	0.987700
C	9.266700	1.700600	1.361100
H	9.556400	0.890500	0.699400
C	8.036700	1.625800	2.018600
C	8.097300	4.994500	3.855700
H	7.188600	5.465000	3.467400
H	8.891700	5.742000	3.854500
H	7.882700	4.723000	4.888700
C	7.181100	0.410200	1.761200
H	6.502100	0.189000	2.583100
H	7.802500	-0.466300	1.570800
H	6.550200	0.573400	0.884700
C	3.888400	2.183500	3.642800
C	3.865200	1.208900	4.645700
H	4.762000	0.629300	4.833900
C	2.735300	1.007400	5.425200
H	2.751900	0.269000	6.218300
C	1.594600	1.773300	5.204200
H	0.729800	1.658300	5.846300
C	1.580900	2.703200	4.169800
H	0.692900	3.301300	3.995400
C	2.713000	2.895400	3.385500
H	2.713300	3.641400	2.598800
C	5.038000	2.704300	1.444100
C	5.707200	3.753500	0.803600
H	6.329300	4.419300	1.390000
C	5.560200	3.967800	-0.561200
H	6.063300	4.806200	-1.027200
C	4.748600	3.129800	-1.320000
H	4.625400	3.289100	-2.385200
C	4.072000	2.089100	-0.694500
H	3.429500	1.438700	-1.271500
C	4.207700	1.887300	0.672000
H	3.674800	1.080900	1.155600

Table S52. The optimized geometric structure of **3** in the QM region at the first excited state (S_1) in solid performed at M062X/6-311G(d) using the Gaussian 09 package.[33]

Atom	x	y	z
C	6.297600	2.624800	3.591700
C	5.023500	2.502300	2.907200
C	6.311700	2.884200	5.027000
C	7.082700	2.044700	5.891200
C	6.964500	2.202100	7.265800
H	7.522300	1.535100	7.914300
C	6.136600	3.173300	7.821100
H	6.070100	3.283300	8.898100
C	5.414300	4.012100	6.983200
H	4.792500	4.798800	7.397000
C	5.483400	3.885700	5.602100
C	7.915300	0.897400	5.374800
H	7.338400	0.271700	4.686700
H	8.249200	0.269900	6.201500
H	8.792600	1.234300	4.823100
C	4.743200	4.893500	4.764600
H	5.072500	4.908700	3.728100
H	4.893400	5.893600	5.174000
H	3.670200	4.687300	4.763300
C	7.553900	2.509300	2.869200
C	8.538700	3.540100	3.013100
C	9.692900	3.485300	2.244200
H	10.417800	4.288200	2.329000
C	9.913200	2.456800	1.330800
H	10.806400	2.457300	0.718300
C	8.958300	1.460100	1.182400
H	9.125700	0.658800	0.471200
C	7.786200	1.462700	1.931600
C	8.279900	4.797300	3.811000
H	7.422600	5.341600	3.396200
H	9.147500	5.458100	3.773800
H	8.031200	4.611400	4.853600
C	6.822900	0.315500	1.769500
H	6.239300	0.145500	2.675300
H	7.361400	-0.601800	1.521800
H	6.107800	0.512200	0.966000
C	3.864000	2.061300	3.662800
C	3.984200	1.156200	4.744900
H	4.938900	0.672200	4.915800
C	2.910100	0.877300	5.576000
H	3.023300	0.173000	6.391700
C	1.689500	1.515500	5.383500
H	0.877500	1.359100	6.083000
C	1.535500	2.395000	4.307700
H	0.591400	2.912100	4.170800
C	2.590900	2.646800	3.451500
H	2.483600	3.376000	2.656600
C	4.901500	2.856500	1.512100
C	5.780100	3.763800	0.865500
H	6.504500	4.313300	1.453200
C	5.686800	3.998300	-0.499700
H	6.338200	4.731600	-0.957800
C	4.760200	3.316800	-1.280200
H	4.704200	3.469800	-2.350600
C	3.886500	2.416400	-0.664000
H	3.159300	1.878500	-1.255600

C	3.943500	2.205000	0.695500
H	3.285900	1.481400	1.152100

Table S53. The optimized geometric structure of **4** in the QM region at the ground state (S_0) in solid performed at M062X/6-311G(d) using the Gaussian 09 package.[33]

Atom	x	y	z
C	13.765800	15.806300	-0.641400
C	13.974100	14.496700	-1.101000
H	13.219000	13.746400	-0.892700
C	15.108100	14.146200	-1.813400
H	15.243200	13.132700	-2.169100
C	16.065500	15.119700	-2.081100
H	16.944700	14.874500	-2.659400
C	15.889000	16.406500	-1.605200
H	16.640400	17.163200	-1.811200
C	14.752200	16.775200	-0.876000
C	14.710000	18.224600	-0.427000
H	14.790100	18.856100	-1.316700
H	15.620600	18.404600	0.146500
C	13.520100	18.705800	0.405000
H	12.617400	18.687500	-0.215700
H	13.700200	19.746800	0.683300
C	13.286700	17.886100	1.645900
C	13.556600	18.377400	2.920200
H	13.988800	19.367200	3.031400
C	13.282400	17.609300	4.048400
H	13.493000	18.009700	5.031700
C	12.731500	16.340200	3.905200
H	12.501300	15.742800	4.780300
C	12.472700	15.833900	2.635800
H	12.045000	14.845700	2.505500
C	12.748200	16.598800	1.507800
C	12.511000	16.086200	0.127300
C	11.290100	15.860500	-0.397800
C	10.010400	16.083200	0.345900
C	9.807500	17.191100	1.178200
H	10.593900	17.926000	1.301700
C	8.610300	17.361900	1.865900
H	8.485500	18.219500	2.517900
C	7.589900	16.425400	1.736100
H	6.666400	16.521800	2.289600
C	7.766500	15.334000	0.897400
H	6.979800	14.596100	0.795300
C	8.958200	15.171900	0.205700
H	9.074400	14.310800	-0.436500
C	11.170200	15.469700	-1.843400
C	10.773200	14.201300	-2.274900
H	10.555600	13.420500	-1.554000
C	10.679000	13.909700	-3.631500
H	10.336500	12.931000	-3.946600
C	11.011700	14.871200	-4.578800
H	10.940100	14.638000	-5.632200
C	11.428900	16.131400	-4.162400
H	11.689200	16.888300	-4.895200
C	11.491100	16.428900	-2.806700
H	11.804800	17.413200	-2.473800

Table S54. The optimized geometric structure of **5** in the QM region at the ground state ($\mathbf{S_0}$) in solid performed at M062X/6-311G(d) using the Gaussian 09 package.[33]

Atom	x	y	z
C	2.067000	2.722300	8.057100
H	1.706600	3.722900	8.307500
H	3.138700	2.693000	8.262800
C	1.856000	2.482100	6.566600
H	2.348800	3.275500	5.999800
H	0.789300	2.585400	6.346300
C	2.324300	1.156700	5.997100
C	2.107100	1.004200	4.625400
H	1.681500	1.838500	4.082200
C	2.426900	-0.160300	3.952300
H	2.229900	-0.229300	2.889600
C	2.999000	-1.217300	4.652900
H	3.233300	-2.149300	4.153900
C	3.272800	-1.061000	6.002500
H	3.751700	-1.868800	6.544800
C	2.946100	0.111600	6.700600
C	3.203500	0.139600	8.179300
C	1.993200	0.422300	9.007900
C	1.433800	-0.544000	9.838400
H	1.920900	-1.510300	9.917600
C	0.279300	-0.269500	10.562800
H	-0.141300	-1.020600	11.221100
C	-0.330500	0.974700	10.449900
H	-1.233100	1.189100	11.007400
C	0.236700	1.946900	9.633200
H	-0.213200	2.932100	9.560200
C	1.398200	1.685500	8.915300
C	5.542900	-2.723100	8.841100
H	5.903200	-3.723700	8.590700
H	4.471100	-2.693700	8.635500
C	5.754000	-2.482800	10.331500
H	5.261200	-3.276000	10.898400
H	6.820700	-2.586000	10.551800
C	5.285700	-1.157300	10.901000
C	5.503000	-1.004700	12.272700
H	5.928600	-1.839000	12.815800
C	5.183300	0.159900	12.945700
H	5.380400	0.229000	14.008300
C	4.611100	1.216800	12.245000
H	4.376900	2.148900	12.743900
C	4.337200	1.060400	10.895400
H	3.858300	1.868200	10.353100
C	4.663900	-0.112300	10.197400
C	4.406500	-0.140400	8.718700
C	5.616800	-0.423100	7.890100
C	6.176300	0.543100	7.059600
H	5.689400	1.509500	6.980400
C	7.330800	0.268400	6.335100
H	7.751400	1.019500	5.676800
C	7.940400	-0.975900	6.448000
H	8.842900	-1.190400	5.890400
C	7.373100	-1.948000	7.264800
H	7.822900	-2.933200	7.337900
C	6.211700	-1.686300	7.982700

Table S55. The optimized geometric structure of **5** in the QM region at the first excited state (**S_1**) in solid performed at M062X/6-311G(d) using the Gaussian 09 package.[33]

Atom	x	y	z
C	1.93685800	2.85974700	7.97568500
H	1.52328900	3.84334900	8.21020600
H	3.00738500	2.88767200	8.18301900
C	1.74679400	2.57787300	6.49376600
H	2.22117800	3.36959700	5.90901700
H	0.68161300	2.64448700	6.25722500
C	2.26171300	1.25131100	5.97492300
C	2.03556000	1.04500100	4.62267500
H	1.55886700	1.84343100	4.07248500
C	2.40510000	-0.11991500	3.96118700
H	2.18358700	-0.23032600	2.90737500
C	3.06320500	-1.11333900	4.67634100
H	3.32053000	-2.05270700	4.20364000
C	3.39279900	-0.89774900	6.00213500
H	3.90011200	-1.68683600	6.54144900
C	2.98395000	0.27171200	6.71670700
C	3.20692300	0.29465200	8.15810900
C	2.04697400	0.58837200	8.99270200
C	1.58654500	-0.34988600	9.94199700
H	2.14447900	-1.26919500	10.08117300
C	0.39525300	-0.14466200	10.62277300
H	0.03242900	-0.89632000	11.31547900
C	-0.32869600	1.02787700	10.42980800
H	-1.24967100	1.19561600	10.97217500
C	0.16730500	2.00090900	9.56174200
H	-0.34337300	2.95128800	9.45626900
C	1.33365800	1.79840600	8.84538700
C	5.67261100	-2.85995400	8.92201500
H	6.08626900	-3.84355600	8.68765400
H	4.60206900	-2.88801700	8.71479700
C	5.86287500	-2.57779500	10.40385200
H	5.38872300	-3.36949900	10.98884500
H	6.92811100	-2.64421900	10.64017500
C	5.34793900	-1.25116300	10.92252500
C	5.57448600	-1.04443900	12.27466400
H	6.05145700	-1.84265700	12.82496100
C	5.20516900	0.12067700	12.93590100
H	5.42707500	0.23146300	13.98958300
C	4.54680000	1.11388500	12.22066500
H	4.28968500	2.05343000	12.69314700
C	4.21679500	0.89791600	10.89501000
H	3.70938600	1.68686100	10.35561100
C	4.62550600	-0.27175000	10.18061200
C	4.40240300	-0.29497700	8.73921900
C	5.56231800	-0.58879000	7.90457700
C	6.02265500	0.34936200	6.95511800
H	5.46464700	1.26860900	6.81584400
C	7.21389900	0.14407000	6.27424100
H	7.57663200	0.89561300	5.58135600
C	7.93792200	-1.02838600	6.46739600
H	8.85885100	-1.19620600	5.92495700

C	7.44206100	-2.00128700	7.33573400
H	7.95284500	-2.95158000	7.44140400
C	6.27568500	-1.79874600	8.05207100

Table S56. The optimized geometric structure of **6** in the QM region at the ground state (**S_0**) in solid performed at M062X/6-311G(d) using the Gaussian 09 package.[33]

Atom	x	y	z
C	8.715800	12.418000	2.026400
C	7.851900	11.369400	1.845200
C	12.448400	9.251400	2.117500
H	13.473300	9.063000	2.442600
H	12.417500	9.071800	1.041300
C	11.496000	8.221500	2.824400
H	11.878800	7.215600	2.643500
H	11.522100	8.400300	3.901900
C	4.865300	15.373300	2.580500
H	4.487900	16.392400	2.521100
H	4.633700	14.997400	3.577700
C	4.087900	14.516800	1.523900
H	3.017700	14.654200	1.696000
H	4.328800	14.900400	0.533200
C	10.165300	12.226600	2.351300
C	10.961400	11.245900	1.749400
H	10.626300	10.787800	0.823300
C	12.016400	10.658400	2.451500
C	12.419900	11.239700	3.655500
H	13.211900	10.769200	4.229800
C	11.785800	12.375900	4.146100
H	12.124800	12.841300	5.065400
C	10.639400	12.846100	3.513200
H	10.054900	13.640200	3.966300
C	8.329400	9.949400	1.779100
C	9.309500	9.446100	2.640700
H	9.592400	10.023300	3.515300
C	10.101000	8.357300	2.270700
C	9.707500	7.596100	1.169500
H	10.293300	6.729000	0.878000
C	8.594900	7.963700	0.423900
H	8.271800	7.343800	-0.397900
C	7.944000	9.164100	0.689400
H	7.160400	9.514000	0.026900
C	8.238400	13.836400	2.001900
C	7.108400	14.238900	2.719300
H	6.715100	13.575700	3.482300
C	6.340000	15.318500	2.284900
C	6.868100	16.144400	1.294200
H	6.274300	16.968300	0.916300
C	8.105100	15.878700	0.728200
H	8.498300	16.554300	-0.019100
C	8.771200	14.699300	1.042500
H	9.663500	14.413000	0.496800
C	6.362900	11.541800	1.779100
C	5.726900	12.644000	1.195000
H	6.270200	13.280600	0.504900
C	4.476500	13.068300	1.651200
C	3.739400	12.224800	2.480600
H	2.763200	12.544500	2.828400

C	4.267500	11.003100	2.874800
H	3.668700	10.320600	3.464400
C	5.586400	10.689400	2.570500
H	6.047000	9.808200	2.997200

8 The photos of TPE derivatives

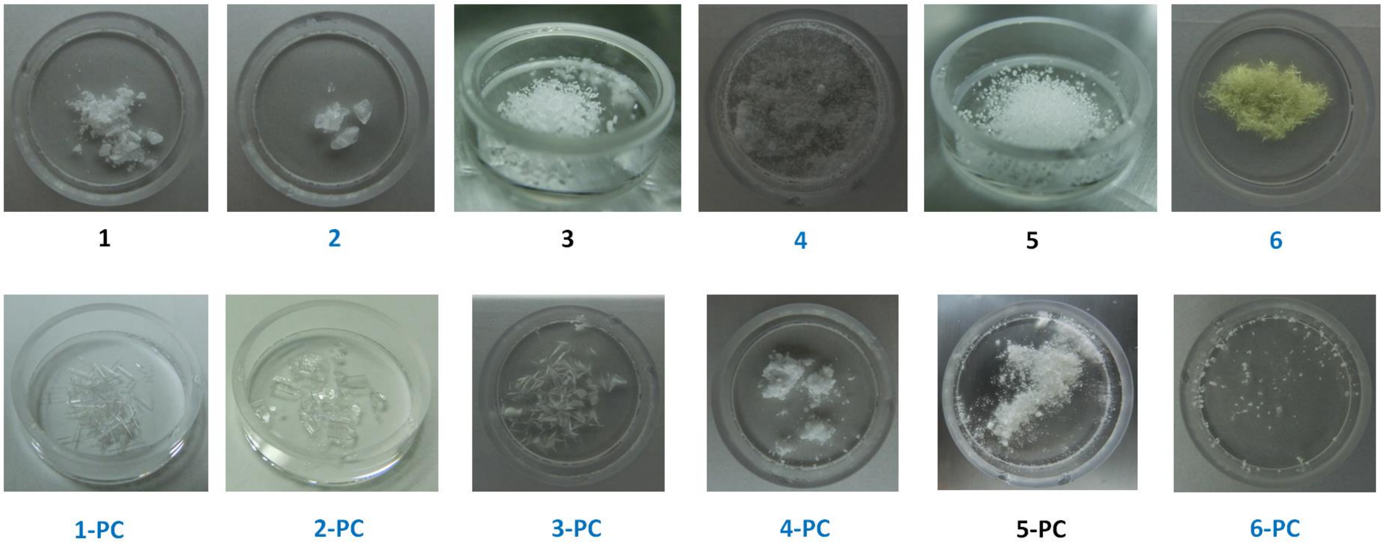

Figure S84. The photos of the TPE derivatives.

9 References

[1] Navale, T. S., Thakur, K. & Rathore, R. Sequential oxidative transformation of tetraarylethylenes to 9,10-diarylphenanthrenes and dibenzo[*g.p*]chrysenes using DDQ as an oxidant. *Org. Lett*. **13,** 1634-1637 (2011).
[2] Aldred, M. P., Li, C. & Zhu, M.-Q. Optical properties and photo-oxidation of tetraphenylethene-based fluorophores. *Chem. Eur. J*. **18,** 16037-16045 (2012).
[3] Zhan, G.-F. *et al*. Direct validation of the restriction of intramolecular rotation hypothesis *via* the synthesis of novel *ortho*-methyl substituted tetraphenylethenes and their application in cell imaging. *Chem. Commun*. **50,** 12058-12060 (2014).
[4] Schmitt, E. *et al*. A general approach towards NH-Pyrazoles that bear diverse fluoroalkyl groups by means of fluorinated iminium salts. *Eur. J. Org. Chem*. **2015**, 6052-6060 (2015).
[5] Luo, J., Song, K., Gu, F. L. & Miao, Q. Switching of non-helical overcrowded tetrabenzoheptafulvalene derivatives. *Chem. Sci*. **2,** 2029-2034 (2011).
[6] Javed, M.I. & Brewer, M. Diphenyldiazomethane. *Org. Synth*. **85,** 189-195 (2008).
[7] Mlostoń, G., Surya Prakash, G. K., Olah, G. A. & Heimgartner, H. Studies on reactions of thioketones with trimethyl(trifluoromethyl)silane catalyzed by fluoride ions. *Helv. Chim. Acta*. **85,** 1644-1658 (2002).

[8] Grützmacher, H.-F. & Husemann, W. [$0^{9,24}$][2.1.2.1] Metacyclophane-9-ene, a “twin cyclophane”, and its dehydrogenation products-synthesis, structure determination and ^{1}H-NMR-spectroscopy. *Tetrahedron Lett*. **26,** 2431-2434 (1985).
[9] Aloïse, S. *et al*. Bridged photochromic diarylethenes investigated by ultrafast absorption spectroscopy: evidence for two distinct photocyclization pathways. *J. Am. Chem. Soc*. **132,** 7379-7390 (2010).
[10] Shultz, D. A. & Fox, M. A. The effect of phenyl ring torsional rigidity on the photophysical behavior of tetraphenylethylenes. *J. Am. Chem. Soc*. **111,** 6311-6320 (1989).
[11] Takeshita, M. & Yamato, T. Synthesis and photochromic properties of 1,2-dicyano[2.n] metacyclophan-1-enes. *Tetrahedron Lett*. **42,** 4345-4347 (2001).
[12] Takeshita, M., Inoue. M, Hisasue, H., Maekawa, S. & Nakamura, T. Synthesis and photochromism of 1,2-dicyano[2.n]metacyclophan-1-enes. *J. Phys. Org. Chem*. **20,** 830-837 (2007).
[13] Dunn, J. P. *et al*. Dibenztroponeacetic and -propionic acids. Potent new antiinflammatory agents. *J. Med. Chem*. **20,** 1557-1562 (1977).
[14] Liu, L., Yang, B., Katz, T. J. & Poindexter, M. K. Improved methodology for photocyclization reactions. *J. Org. Chem.* **56,** 3769-3775 (1991).
[15] Dolomanov, O. V., Bourhis, L. J., Gildea, R. J., Howard, J. A. K. & Puschmann, H. OLEX2: a complete structure solution, refinement and analysis program. *J. Appl. Cryst.* **42,** 339-341 (2009).
[16] Sheldrick, G. M. A short history of SHELX. *Acta Cryst. A.* **64,** 112-122 (2008).
[17] Sheldrick, G. M. Crystal structure refinement with SHELXL. *Acta Cryst. C.* **71,** 3-8 (2015).
[18] Shustova, N. B. *et al*. Phenyl ring dynamics in a tetraphenylethylene-bridged metal-organic framework: implications for the mechanism of aggregation-induced emission. *J. Am. Chem. Soc*. **134,** 15061-15070 (2012).
[19] Leung, N. L. C. *et al.* Restriction of intramolecular motions: the general mechanism behind aggregation-induced emission. *Chem. Eur. J.* **20,** 15349-15353 (2014).
[20] Huang, G. *et al*. Dendron-containing tetraphenylethylene compounds: dependence of fluorescence and photocyclization reactivity on the dendron generation. *Chem. Eur. J.* **18,** 3886-3892 (2012).
[21] Schilling, C. L. & Hilinski, E. F. Dependence of the lifetime of the twisted excited singlet state of tetraphenylethylene on solvent polarity. *J. Am. Chem. Soc*. **110,** 2296-2298 (1988).
[22] Sun, Y. P. & Fox, M. A. Picosecond transient absorption study of the twisted excited singlet state of tetraphenylethylene in supercritical fluids. *J. Am. Chem. Soc.* **115,** 747-750 (1993).
[23] Greene, B. I. Observation of a long-lived twisted intermediate following picosecond uv excitation of tetraphenylethylene. *Chem. Phys. Lett*. **79,** 51-53 (1981).
[24] Zijlstra, R. W. J. *et al*. Excited-state dynamics of tetraphenylethylene: ultrafast stokes shift, isomerization, and charge separation. *J. Phys. Chem. A.* **101,** 9828-9836 (1997).
[25] Kayal, S.; Roy, K. & Umapathy, S. Femtosecond coherent nuclear dynamics of excited tetraphenylethylene: ultrafast transient absorption and ultrafast Raman loss spectroscopic studies. *J. Chem. Phys.* **148,** 024301 (2018).
[26] Lenderink, E., Duppen, K. & Wiersma, D. A. Femtosecond twisting and coherent vibrational motion in the excited state of tetraphenylethylene. *J. Phys. Chem.* **99,** 8972-8977 (1995).
[27] Zhao, G-J., Han, K.-L., Lei, Y.-B. & Dou, Y.-S. Ultrafast excited-state dynamics of tetraphenylethylene studied by semiclassical simulation. *J. Chem. Phys.* **127,** 094307 (2007).
[28] Du, L. *et al*. Direct detection of the open-shell singlet phenyloxenium ion: an atom-centered diradical reacts as an electrophile. *J. Am. Chem. Soc.* **139,** 15054-15059 (2017).
[29] Du, L. *et al*. Influence of water in the photogeneration and properties of a bifunctional quinone methide. *J. Phys. Chem. B.* **120,** 11132-11141 (2016).
[30] Chan, K. T. *et al*. The interplay between fluorescence and phosphorescence with luminescent gold(i) and gold(iii) complexes bearing heterocyclic arylacetylide ligands. *Chem. Sci.* **8,** 2352-2364 (2017).
[31] Zhao, Y. & Truhlar, D. G. The M06 suite of density functionals for main group thermochemistry, thermochemical kinetics, noncovalent interactions, excited states, and transition elements: two new functionals and systematic testing of four M06-class functionals and 12 other functionals. *Theor. Chem. Acc.*, **120,** 215-241(2008).

[32] Krishnan, R., Binkley, J. S., Seeger, R. & Pople, J. A. Self-consistent molecular orbital methods. XX. A basis set for correlated wave-functions. *J. Chem. Phys.*, **72,** 650-654 (1980).
[33] Frisch, M. J.; Trucks, G. W.; Schlegel, H. B.; Scuseria, G. E.; Robb, M. A.; Cheeseman, J. R.; Scalmani, G.; Barone, V.; Mennucci, B.; Petersson, G. A.; Nakatsuji, H.; Caricato, M.; Li, X.; Hratchian, H. P.; Izmaylov, A. F.; Bloino, J.; Zheng, G.; Sonnenberg, J. L.; Hada, M.; Ehara, M.; Toyota, K.; Fukuda, R.; Hasegawa, J.; Ishida, M.; Nakajima, T.; Honda, Y.; Kitao, O.; Nakai, H.; Vreven, T.; Montgomery, Jr., J. A.; Peralta, J. E.; Ogliaro, F.; Bearpark, M. ; Heyd, J. J.; Brothers, E.; Kudin, K. N.; Staroverov, V. N.; Keith, T.; Kobayashi, R.; Normand, J.; Raghavachari, K.; Rendell, A.; Burant, J. C.; Iyengar, S. S.; Tomasi, J.; Cossi, M.; Rega, N.; Millam, J. M.; Klene, M.; Knox, J. E.; Cross, J. B.; Bakken, V.; Adamo, C.; Jaramillo, J.; Gomperts, R.; Stratmann, R. E.; Yazyev, O.; Austin, A. J.; Cammi, R.; Pomelli, C.; Ochterski, J. W.; Martin, R. L.; Morokuma, K.; Zakrzewski, V. G.; Voth, G. A.; Salvador, P.; Dannenberg, J. J.; Dapprich, S.; Daniels, A. D.; Farkas, O.; Foresman, J. B.; Ortiz, J. V.; Cioslowski, J.; Fox, D. J. EM64L-Gaussian 09, Revision D.01. Gaussian, Inc., Wallingford CT, **2013**.
[34] Laarhoven, W. H. Photochemical cyclizations and intramolecular cycloadditions of conjugated arylolefins. Part I: Photocyclization with dehydrogenation. *Recl. Trav. Chim. Pays-Bas.* **102,** 185-204 (1983).
[35] Jørgensen, K. B. Photochemical oxidative cyclisation of stilbenes and stilbenoids-the mallory-reaction. *Molecules.* **15,** 4334-4358 (2010).
[36] Mallory, F. B. & Mallory, C. W. in *Photocyclization of Stilbenes and Related Molecules, Organic Reactions,* **30,** *Ch.1,* 1-456 (John Wiley & Sons, Inc., 2005).
[37] Irie, M. Diarylethenes for memories and switches. *Chem. Rev.* **100,** 1685-1716 (2000).
[38] Uchida, K., Ito, S., Nakano, M., Abe, M. & Kubo, T. Biphenalenylidene: isolation and characterization of the reactive intermediate on the decomposition pathway of phenalenyl radical. *J. Am. Chem. Soc.* **138,** 2399-2410 (2016).
[39] He, Z. *et al.* Aggregation-induced emission and aggregation-promoted photochromism of bis(diphenylmethylene)dihydroacenes. *Chem. Sci.* **6,** 3538-3543 (2015).
[40] Gu, X. *et al.* A luminescent nitrogen-containing polycyclic aromatic hydrocarbon synthesized by photocyclodehydrogenation with unprecedented regioselectivity. *Chem. Eur. J.* **21,** 17973-17980 (2015).
[41] Gao, Y.-J. *et al*. Excited-state decay paths in tetraphenylethene derivatives. *J. Phys. Chem. A.* **121,** 2572-2579 (2017).
[42] Van Stokkum, I. H. M., Larsen, D. S. & Van Grondelle, R. Global and target analysis of time-resolved spectra. *Biochim. Biophys. Acta-Bioenerg*. **1657,** 82-104 (2004).
[43] Fukui, K. The path of chemical reactions - The IRC approach. *Acc. Chem. Res.*, **14,** 363-368 (1981).
[44] Qi, F. *Light-driven molecular rotary motors*. PhD Thesis, Hong Kong Baptist University (2017).
[45] Conyard, J. *et al*. Ultrafast dynamics in the power stroke of a molecular rotary motor. *Nat. Chem.* **4,** 547-551 (2012).
[46] Laarhoven, W. H., Cuppen, Th. J. H. M. & Nivard, R. J. F. Photodehydrocyclizations in stilbene-like compounds, *Recl. Trav. Chim. Pays-Bas.* **87,** 687–698 (1968).
[47] Laarhoven, W. H., Cuppen, Th. J. H. M. & Nivard, R. J. F. Photodehydrocyclizations in stilbene-like compounds—II : Photochemistry of distyrylbenzenes. *Tetrahedron.* **26,** 1069–1083 (1970).
[48] Scholz, M., Mühlstädt, M. & Dietz, F. Chemie angeregter Zustände. I. Mitt. Die Richtung der Photocyclisierung naphthalinsubstituierter äthylene. *Tetrahedron Lett.* **7,** 665– 668 (1967).
[49] Blackburn, E. V., Loader, C. E. & Timmons, C. J. Studies in photochemistry. Part VIII. The ultra-violet, proton magnetic resonance, and mass spectra, and photocyclisation of some styrylnaphthalenes to some benzo[*c*]phenanthrenes and chrysenes. *J. Chem. Soc. C.* 163–171 (1970).
[50] Laarhoven, W. H. in *Photochromism: Molecules and Systems,* 270-313 (eds Duerr, H. & Bouas-Laurent, H. Elsevier: Amsterdam, 1990) and references therein.
[51] Streitweiser, A. in *Molecular orbital theory for organic chemists,* 56, (Wiley: New York, 1962).
[52] Mazzucato, U. & Spalletti, A. Competition between photoisomerization and photocyclization of the cis isomers of n-styrynaphthalenes and -phenanthrenes. *J. Phys. Chem. A.* **113,** 14521-14529 (2009).
[53] Young, D. C. in *Computational Chemistry: A Practical Guide for Applying Techniques to Real-World Problems, Ch.17,* 147-158 (John Wiley & Sons, Inc., 2001)

[54] Frisch, M. J.; Trucks, G. W.; Schlegel, H. B.; Scuseria, G. E.; Robb, M. A.; Cheeseman, J. R.; Scalmani, G.; Barone, V.; Petersson, G. A.; Nakatsuji, H.; Li, X.; Caricato, M.; Marenich, A. V.; Bloino, J.; Janesko, B. G.; Gomperts, R.; Mennucci, B.; Hratchian, H. P.; Ortiz, J. V.; Izmaylov, A. F.; Sonnenberg, J. L.; WilliamsYoung, D.; Ding, F.; Lipparini, F.; Egidi, F.; Goings, J.; Peng, B.; Petrone, A.; Henderson, T.; Ranasinghe, D.; Zakrzewski, V. G.; Gao, J.; Rega, N.; Zheng, G.; Liang, W.; Hada, M.; Ehara, M.; Toyota, K.; Fukuda, R.; Hasegawa, J.; Ishida, M.; Nakajima, T.; Honda, Y.; Kitao, O.; Nakai, H.; Vreven, T.; Throssell, K.; Montgomery, J. A., Jr.; Peralta, J. E.; Ogliaro, F.; Bearpark, M. J.; Heyd, J. J.; Brothers, E. N.; Kudin, K. N.; Staroverov, V. N.; Keith, T. A.; Kobayashi, R.; Normand, J.; Raghavachari, K.; Rendell, A. P.; Burant, J. C.; Iyengar, S. S.; Tomasi, J.; Cossi, M.; Millam, J. M.; Klene, M.; Adamo, C.; Cammi, R.; Ochterski, J. W.; Martin, R. L.; Morokuma, K.; Farkas, O.; Foresman, J. B.; Fox, D. J. Gaussan 16, Revision A.03, Gaussian, Inc., Wallingford CT, 2016.

[55] Hoekstra, A. & Vos, A. The crystal and molecular structures of tetraphenylhydrazine and related compounds at -160°C. II. The crystal structures of tetraphenylethylene (TPE) and diphenylaminotriphenylmethane (DTM). *Acta Cryst. B.* **31,** 1716-1721 (1975).

[56] Sun, G., Zhao, Y. & Liang, W. Aggregation-induced emission mechanism of dimethoxy-tetraphenylethylene in water solution: molecular dynamics and QM/MM investigations. *J. Chem. Theory Comput.* **11,** 2257-2267 (2015).

[57] Maseras, F. & Morokuma, K. IMOMM: A new integrated *ab initio* + molecular mechanics geometry optimization scheme of equilibrium structures and transition states. *J. Comput. Chem*. **16,** 1170-1179 (1995).

[58] Svensson, M. *et al*. ONIOM: A multilayered integrated MO + MM method for geometry optimizations and single point energy predictions. A test for Diels-Alder reactions and $Pt(P(^tBu)_3)_2$ + H_2 oxidative addition. *J. Phys. Chem.* **100,** 19357-19363 (1996).

[59] Dapprich, S., Komáromi, I., Byun, K. S., Morokuma, K. & Frisch, M. J. A new ONIOM implementation in Gaussian98. Part I. The calculation of energies, gradients, vibrational frequencies and electric field derivatives1. *J. Mol. Struct. (THEOCHEM)*. **461,** 1-23 (1999).

[60] Vreven, T. *et al*. Combining quantum mechanics methods with molecular mechanics methods in ONIOM. *J. Chem. Theory Comput*. **2,** 815-826 (2006).

[61] Rappe, A. K, Casewit, C. J., Colwell, K. S., Goddard III, W. A. & Skiff, W. A. UFF, a full periodic table force field for molecular mechanics and molecular dynamics simulations. *J. Am. Chem. Soc*. **114,** 10024-10035 (1992).

[62] Bakowies, D. & Thiel, W. Hybrid models for combined quantum mechanical and molecular mechanical approaches. *J. Phys. Chem*. **100,** 10580-10594 (1996).
